# Supplementary material for: Prevalence of chronic conditions in masters games athletes: predictors and comparison to the general population
Source: PeerJ. 2025 Feb 18;13:e18912. doi: 10.7717/peerj.18912 (PMC11844256; doi:10.7717/peerj.18912)
Supplement: Supplemental Information 1 [file peerj-13-18912-s001.pdf]

Survey to investigate the prevalence of nutrition-related chronic conditions in Australian Masters athletes

This is an online survey asking registered Australian Masters Games athletes about demographic information, health-related information including medical history and treatment strategies, history of engagement with physical activity, and sources of nutrition information. The physiological effects of the normal ageing process alongside age- or lifestyle-associated health conditions need to be taken into account when recommendations are provided for optimal athletic performance for Masters Athletes. This research will help to inform health professionals about education needs and advice for treatment and performance, and guide further research specific to Masters Athletes. Promotion of the health benefits Masters level activity may encourage others in this age-group to consider participation.

The study is being conducted by Dr Janelle Gifford, Mrs Wendy Stuart-Smith, Associate Professor Mike Climstein, Associate Professor Helen O'Connor and Xiaojing Wu (Masters of Nutrition and Dietetics Candidate) at University of Sydney.
Contact email: janelle.gifford@sydney.edu.au

By clicking the "Continue" button at the bottom of the screen, you are:
1. acknowledging that you have read the information sheet, which explains the aims of the study and have contacted the study researchers if you needed to clarify the details of the study;
2. acknowledging that you understand that you can withdraw from the study at any time without prejudice to your relationship to The University of Sydney;
3. agreeing that research data gathered from the results of the study may be published, provided that you cannot be identified;
4. acknowledging that you understand that if you have any questions relating to your participation in this research, and you may contact Dr Janelle Gifford at janelle.gifford@sydney.edu.au, who will be happy to answer them;
5. consenting to participate in the study described in the information sheet provided.

Please be assured that no information that can identify you will be kept, and that the information you enter will be securely held in confidence.

[Attachment: "Participant Information Sheet.pdf"]

Consent to participate

◯ Choose not to participate
◯ CONTINUE   (If you click continue, you are consenting to participate in the study described in the information sheet provided above.)

If you click continue, you are consenting to participate in the study described in the information sheet provided.

[Attachment: "Participant Information Sheet.pdf"]

Have you already participated in this survey?

◯ Yes
◯ No

What is your age in years?

- ◯ < 18
- ◯ 18
- ◯ 19
- ◯ 20
- ◯ 21
- ◯ 22
- ◯ 23
- ◯ 24
- ◯ 25
- ◯ 26
- ◯ 27
- ◯ 28
- ◯ 29
- ◯ 30
- ◯ 31
- ◯ 32
- ◯ 33
- ◯ 34
- ◯ 35
- ◯ 36
- ◯ 37
- ◯ 38
- ◯ 39
- ◯ 40
- ◯ 41
- ◯ 42
- ◯ 43
- ◯ 44
- ◯ 45
- ◯ 46
- ◯ 47
- ◯ 48
- ◯ 49
- ◯ 50
- ◯ 51
- ◯ 52
- ◯ 53
- ◯ 54
- ◯ 55
- ◯ 56
- ◯ 57
- ◯ 58
- ◯ 59
- ◯ 60
- ◯ 61
- ◯ 62
- ◯ 63
- ◯ 64
- ◯ 65
- ◯ 66
- ◯ 67
- ◯ 68
- ◯ 69
- ◯ 70
- ◯ 71
- ◯ 72
- ◯ 73
- ◯ 74
- ◯ 75
- ◯ 76
- ◯ 77
- ◯ 78
- ◯ 79
- ◯ 80
- ◯ 81
- ◯ 82
- ◯ 83
- ◯ 84
- ◯ 85

	◯ 86<br>◯ 87<br>◯ 88<br>◯ 89<br>◯ 90<br>◯ 91<br>◯ 92<br>◯ 93<br>◯ 94<br>◯ 95<br>◯ 96<br>◯ 97<br>◯ 98<br>◯ 99<br>◯ 100
Are you an athlete registered to participate in the Australian Masters Games?	◯ Yes<br>◯ No
What is your gender?	◯ Male<br>◯ Female

Do any of the following apply to you?
1. Currently pregnant
2. Currently breastfeeding
3. Currently experiencing menopause
4. Post menopause

◯ Yes ◯ No

Are you pregnant or breastfeeding?

◯ Yes ◯ No

Which of the following apply to you? (pregnant and breastfeeding can both be selected)
(Tick all that apply)

☐ Currently pregnant ☐ Currently breastfeeding

Which of the following apply to you?

◯ Currently experiencing menopause ◯ Post menopause

How many weeks pregnant are you?	◯ < 12 weeks<br>◯ 12 weeks<br>◯ 13 weeks<br>◯ 14 weeks<br>◯ 15 weeks<br>◯ 16 weeks<br>◯ 17 weeks<br>◯ 18 weeks<br>◯ 19 weeks<br>◯ 20 weeks<br>◯ 21 weeks<br>◯ 22 weeks<br>◯ 23 weeks<br>◯ 24 weeks<br>◯ 25 weeks<br>◯ 26 weeks<br>◯ 27 weeks<br>◯ 28 weeks<br>◯ 29 weeks<br>◯ 30 weeks<br>◯ 31 weeks<br>◯ 32 weeks<br>◯ 33 weeks<br>◯ 34 weeks<br>◯ 35 weeks<br>◯ 36 weeks<br>◯ 37 weeks<br>◯ 38 weeks<br>◯ 39 weeks<br>◯ 40 weeks<br>◯ Don't know
Do you speak English at home?	◯ Yes<br>◯ No
Are you Aboriginal or Torres Strait Islander origin?	◯ Yes<br>◯ No

In which country were you born?

◯ Afghanistan
◯ Albania
◯ Algeria
◯ Andorra
◯ Angola
◯ Antigua & Deps
◯ Argentina
◯ Armenia
◯ Australia
◯ Austria
◯ Azerbaijan
◯ Bahamas
◯ Bahrain
◯ Bangladesh
◯ Barbados
◯ Belarus
◯ Belgium
◯ Belize
◯ Benin
◯ Bhutan
◯ Bolivia
◯ Bosnia Herzegovina
◯ Botswana
◯ Brazil
◯ Brunei
◯ Bulgaria
◯ Burkina
◯ Burundi
◯ Cambodia
◯ Cameroon
◯ Canada
◯ Cape Verde
◯ Central African Rep
◯ Chad
◯ Chile
◯ China
◯ Colombia
◯ Comoros
◯ Congo
◯ Congo {Democratic Rep}
◯ Costa Rica
◯ Croatia
◯ Cuba
◯ Cyprus
◯ Czech Republic
◯ Denmark
◯ Djibouti
◯ Dominica
◯ Dominican Republic
◯ East Timor
◯ Ecuador
◯ Egypt
◯ El Salvador
◯ Equatorial Guinea
◯ Eritrea
◯ Estonia
◯ Ethiopia
◯ Fiji
◯ Finland
◯ France
◯ Gabon
◯ Gambia
◯ Georgia
◯ Germany
◯ Ghana
◯ Greece
◯ Grenada
◯ Guatemala
◯ Guinea

◯ Guinea-Bissau
◯ Guyana
◯ Haiti
◯ Honduras
◯ Hungary
◯ Iceland
◯ India
◯ Indonesia
◯ Iran
◯ Iraq
◯ Ireland {Republic}
◯ Israel
◯ Italy
◯ Ivory Coast
◯ Jamaica
◯ Japan
◯ Jordan
◯ Kazakhstan
◯ Kenya
◯ Kiribati
◯ Korea North
◯ Korea South
◯ Kosovo
◯ Kuwait
◯ Kyrgyzstan
◯ Laos
◯ Latvia
◯ Lebanon
◯ Lesotho
◯ Liberia
◯ Libya
◯ Liechtenstein
◯ Lithuania
◯ Luxembourg
◯ Macedonia
◯ Madagascar
◯ Malawi
◯ Malaysia
◯ Maldives
◯ Mali
◯ Malta
◯ Marshall Islands
◯ Mauritania
◯ Mauritius
◯ Mexico
◯ Micronesia
◯ Moldova
◯ Monaco
◯ Mongolia
◯ Montenegro
◯ Morocco
◯ Mozambique
◯ Myanmar {Burma}
◯ Namibia
◯ Nauru
◯ Nepal
◯ Netherlands
◯ New Zealand
◯ Nicaragua
◯ Niger
◯ Nigeria
◯ Norway
◯ Oman
◯ Pakistan
◯ Palau
◯ Panama
◯ Papua New Guinea
◯ Paraguay
◯ Peru
◯ Philippines
◯ Poland

◯ Portugal
◯ Qatar
◯ Romania
◯ Russian Federation
◯ Rwanda
◯ St Kitts & Nevis
◯ St Lucia
◯ Saint Vincent & the Grenadines
◯ Samoa
◯ San Marino
◯ Sao Tome & Principe
◯ Saudi Arabia
◯ Senegal
◯ Serbia
◯ Seychelles
◯ Sierra Leone
◯ Singapore
◯ Slovakia
◯ Slovenia
◯ Solomon Islands
◯ Somalia
◯ South Africa
◯ Spain
◯ Sri Lanka
◯ Sudan
◯ Suriname
◯ Swaziland
◯ Sweden
◯ Switzerland
◯ Syria
◯ Taiwan
◯ Tajikistan
◯ Tanzania
◯ Thailand
◯ Togo
◯ Tonga
◯ Trinidad & Tobago
◯ Tunisia
◯ Turkey
◯ Turkmenistan
◯ Tuvalu
◯ Uganda
◯ Ukraine
◯ United Arab Emirates
◯ United Kingdom
◯ United States
◯ Uruguay
◯ Uzbekistan
◯ Vanuatu
◯ Vatican City
◯ Venezuela
◯ Vietnam
◯ Yemen
◯ Zambia
◯ Zimbabwe

What is your current nationality?

- ◯ Afghanistan
- ◯ Albania
- ◯ Algeria
- ◯ Andorra
- ◯ Angola
- ◯ Antigua & Deps
- ◯ Argentina
- ◯ Armenia
- ◯ Australia
- ◯ Austria
- ◯ Azerbaijan
- ◯ Bahamas
- ◯ Bahrain
- ◯ Bangladesh
- ◯ Barbados
- ◯ Belarus
- ◯ Belgium
- ◯ Belize
- ◯ Benin
- ◯ Bhutan
- ◯ Bolivia
- ◯ Bosnia Herzegovina
- ◯ Botswana
- ◯ Brazil
- ◯ Brunei
- ◯ Bulgaria
- ◯ Burkina
- ◯ Burundi
- ◯ Cambodia
- ◯ Cameroon
- ◯ Canada
- ◯ Cape Verde
- ◯ Central African Rep
- ◯ Chad
- ◯ Chile
- ◯ China
- ◯ Colombia
- ◯ Comoros
- ◯ Congo
- ◯ Congo {Democratic Rep}
- ◯ Costa Rica
- ◯ Croatia
- ◯ Cuba
- ◯ Cyprus
- ◯ Czech Republic
- ◯ Denmark
- ◯ Djibouti
- ◯ Dominica
- ◯ Dominican Republic
- ◯ East Timor
- ◯ Ecuador
- ◯ Egypt
- ◯ El Salvador
- ◯ Equatorial Guinea
- ◯ Eritrea
- ◯ Estonia
- ◯ Ethiopia
- ◯ Fiji
- ◯ Finland
- ◯ France
- ◯ Gabon
- ◯ Gambia
- ◯ Georgia
- ◯ Germany
- ◯ Ghana
- ◯ Greece
- ◯ Grenada
- ◯ Guatemala
- ◯ Guinea

◯ Guinea-Bissau
◯ Guyana
◯ Haiti
◯ Honduras
◯ Hungary
◯ Iceland
◯ India
◯ Indonesia
◯ Iran
◯ Iraq
◯ Ireland {Republic}
◯ Israel
◯ Italy
◯ Ivory Coast
◯ Jamaica
◯ Japan
◯ Jordan
◯ Kazakhstan
◯ Kenya
◯ Kiribati
◯ Korea North
◯ Korea South
◯ Kosovo
◯ Kuwait
◯ Kyrgyzstan
◯ Laos
◯ Latvia
◯ Lebanon
◯ Lesotho
◯ Liberia
◯ Libya
◯ Liechtenstein
◯ Lithuania
◯ Luxembourg
◯ Macedonia
◯ Madagascar
◯ Malawi
◯ Malaysia
◯ Maldives
◯ Mali
◯ Malta
◯ Marshall Islands
◯ Mauritania
◯ Mauritius
◯ Mexico
◯ Micronesia
◯ Moldova
◯ Monaco
◯ Mongolia
◯ Montenegro
◯ Morocco
◯ Mozambique
◯ Myanmar {Burma}
◯ Namibia
◯ Nauru
◯ Nepal
◯ Netherlands
◯ New Zealand
◯ Nicaragua
◯ Niger
◯ Nigeria
◯ Norway
◯ Oman
◯ Pakistan
◯ Palau
◯ Panama
◯ Papua New Guinea
◯ Paraguay
◯ Peru
◯ Philippines
◯ Poland

◯ Portugal
◯ Qatar
◯ Romania
◯ Russian Federation
◯ Rwanda
◯ St Kitts & Nevis
◯ St Lucia
◯ Saint Vincent & the Grenadines
◯ Samoa
◯ San Marino
◯ Sao Tome & Principe
◯ Saudi Arabia
◯ Senegal
◯ Serbia
◯ Seychelles
◯ Sierra Leone
◯ Singapore
◯ Slovakia
◯ Slovenia
◯ Solomon Islands
◯ Somalia
◯ South Africa
◯ Spain
◯ Sri Lanka
◯ Sudan
◯ Suriname
◯ Swaziland
◯ Sweden
◯ Switzerland
◯ Syria
◯ Taiwan
◯ Tajikistan
◯ Tanzania
◯ Thailand
◯ Togo
◯ Tonga
◯ Trinidad & Tobago
◯ Tunisia
◯ Turkey
◯ Turkmenistan
◯ Tuvalu
◯ Uganda
◯ Ukraine
◯ United Arab Emirates
◯ United Kingdom
◯ United States
◯ Uruguay
◯ Uzbekistan
◯ Vanuatu
◯ Vatican City
◯ Venezuela
◯ Vietnam
◯ Yemen
◯ Zambia
◯ Zimbabwe

In which state or territory of Australia do you currently live?

◯ ACT
◯ NSW
◯ NT
◯ Queensland
◯ SA
◯ Tasmania
◯ Victoria
◯ Western Australia
◯ Not currently living in Australia

How would you describe where you live?	◯ Major urban centre (population of 100,000 or more)<br>◯ Other urban centre (population between 1000 and 99,999)<br>◯ Bounded locality (population between 200 and 999)<br>◯ Rural (population < 200)
What is the highest year of primary or secondary school that you have completed?	◯ Year 12 or equivalent<br>◯ Year 11 or equivalent<br>◯ Year 10 or equivalent<br>◯ Year 9 or equivalent<br>◯ Year 8 or below<br>◯ Never attended school
Have you completed a trade certificate, diploma, degree or any other educational qualification?	◯ Yes<br>◯ No<br>◯ Don't know
What is the highest qualification that you have completed?	◯ Trade certificate<br>◯ Diploma<br>◯ Bachelors degree<br>◯ Graduate diploma or certificate<br>◯ Masters degree<br>◯ PhD<br>◯ Other
please specify the highest qualification you have completed	______________________
This question is about your employment. Are you currently....<br>(More than one response is allowed)<br>(Tick all that apply)<br><br>☐ Employed casually for wages<br>☐ Employed part-time for wages<br>☐ Employed full-time for wages<br>☐ Self-employed<br>☐ Unemployed<br>☐ A homemaker<br>☐ A student<br>☐ Retired<br>☐ Unable to work<br>☐ Other (please specify)	
please specify your employment status	______________________
Before income tax, salary sacrifice or anything else is taken out, how much income in total do all members of your household (aged 15 years or over) usually receive from all sources? (amount is in Australian dollars)	◯ Prefer not to say<br>◯ Less than $20,000<br>◯ $20,001-$40,000<br>◯ $40,001-$60,000<br>◯ $60,001-$80,000<br>◯ $80,001-$100,000<br>◯ $100,001-$120,000<br>◯ $120,001-$140,000<br>◯ $140,001-$160,000<br>◯ $160,001-$180,000<br>◯ More than $180,000<br>◯ Don't Know

How long have you been competing in Masters level competition?

- ◯ Less than 1 year
- ◯ 1 year
- ◯ 2 years
- ◯ 3 years
- ◯ 4 years
- ◯ 5 years
- ◯ 6 years
- ◯ 7 years
- ◯ 8 years
- ◯ 9 years
- ◯ 10 years
- ◯ 11 years
- ◯ 12 years
- ◯ 13 years
- ◯ 14 years
- ◯ 15 years
- ◯ 16 years
- ◯ 17 years
- ◯ 18 years
- ◯ 19 years
- ◯ 20 years
- ◯ 21 years
- ◯ 22 years
- ◯ 23 years
- ◯ 24 years
- ◯ 25 years
- ◯ 26 years
- ◯ 27 years
- ◯ 28 years
- ◯ 29 years
- ◯ 30 years
- ◯ 31 years
- ◯ 32 years
- ◯ 33 years
- ◯ 34 years
- ◯ 35 years
- ◯ 36 years
- ◯ 37 years
- ◯ 38 years
- ◯ 39 years
- ◯ 40 years
- ◯ 41 years
- ◯ 42 years
- ◯ 43 years
- ◯ 44 years
- ◯ 45 years
- ◯ 46 years
- ◯ 47 years
- ◯ 48 years
- ◯ 49 years
- ◯ 50 years
- ◯ 51 years
- ◯ 52 years
- ◯ 53 years
- ◯ 54 years
- ◯ 55 years
- ◯ 56 years
- ◯ 57 years
- ◯ 58 years
- ◯ 59 years
- ◯ 60 years
- ◯ 61 years
- ◯ 62 years
- ◯ 63 years
- ◯ 64 years
- ◯ 65 years
- ◯ 66 years
- ◯ 67 years
- ◯ 68 years

◯ 69 years
◯ 70 years
◯ 71 years
◯ 72 years
◯ 73 years
◯ 74 years
◯ 75 years
◯ 76 years
◯ 77 years
◯ 78 years
◯ 79 years
◯ 80 years
◯ 81 years
◯ 82 years
◯ 83 years
◯ 84 years
◯ 85 years
◯ 86 years
◯ 87 years
◯ 88 years
◯ 89 years
◯ 90 years
◯ 91 years
◯ 92 years
◯ 93 years
◯ 94 years
◯ 95 years
◯ 96 years
◯ 97 years
◯ 98 years
◯ 99 years

What sport/s are you competing in at the Australian Masters Games?
(Tick all that apply)

☐ Archery
☐ Athletics
☐ Badminton
☐ Baseball
☐ Basketball
☐ Beach volleyball
☐ BMX
☐ Bocce
☐ Boxing
☐ Canoe/Kayak/SUP
☐ Canoe outrigger
☐ Cricket
☐ Croquet
☐ Cue sports
☐ Cycling - track
☐ Cycling - road
☐ Darts
☐ Dragon boat
☐ Duathlon
☐ Equestrian
☐ Fly fishing
☐ Football - AFL
☐ Football- soccer
☐ Futsal
☐ Golf
☐ Gymnastics
☐ Hockey
☐ Judo
☐ Karate
☐ Lawn bowls
☐ Motorsport - hill climb
☐ Mountain bike cross country
☐ Netball
☐ Pool lifesaving
☐ Powerlifting
☐ Racquetball
☐ Rowing
☐ Rowing - indoor
☐ Rugby union
☐ Sailing
☐ Shooting clay target
☐ Shooting smallbore & air rifle
☐ Shooting filed rifle
☐ Softball
☐ Squash
☐ Swimming
☐ Table tennis
☐ Taekwondo
☐ Tennis
☐ Tenpin Bowling
☐ Touch football
☐ Trail running
☐ Volleyball

Which categories will you compete in archery?
(Tick all that apply)

☐ Target
☐ Clout
☐ Filed round Marked

Which age group will you compete in archery?

- ◯ 30-39
- ◯ 40-49
- ◯ 50-59
- ◯ 60-69
- ◯ 70-79
- ◯ 80+

Which categories will you compete in athletics?
(Tick all that apply)

- ☐ 60m
- ☐ 100m
- ☐ 200m
- ☐ 400m
- ☐ 800m
- ☐ 1500m
- ☐ 5000m
- ☐ 80m Hurdles
- ☐ 100m Hurdles
- ☐ 110m Hurdles
- ☐ 200m Hurdles
- ☐ 300m Hurdles
- ☐ 400m Hurdles
- ☐ 1500m Walk
- ☐ 3000m Walk
- ☐ 5000m Walk
- ☐ 2000m Steeplechase
- ☐ 3000m Steeplechase
- ☐ Long Jump
- ☐ High Jump
- ☐ Triple Jump
- ☐ Pole Vault
- ☐ Shot Put
- ☐ Javelin
- ☐ Discus
- ☐ Weight Throw
- ☐ Hammer
- ☐ Throws Pentathlon
- ☐ Track and Field Pentathlon
- ☐ Road Race 10km
- ☐ Street Mile
- ☐ Half Marathon

Which age group will you compete in athletics?

- ◯ 30-34
- ◯ 35-39
- ◯ 40-44
- ◯ 45-49
- ◯ 50-54
- ◯ 55-59
- ◯ 60-64
- ◯ 65-69
- ◯ 70-74
- ◯ 75-79
- ◯ 80-84
- ◯ 85-89
- ◯ 90-94
- ◯ 95+

Which categories will you compete in badminton?
(Tick all that apply)

☐ Men's Singles
☐ Men's Doubles
☐ Mixed Doubles
☐ Teams

Which categories will you compete in badminton?
(Tick all that apply)

☐ Women's Singles
☐ Women's Doubles
☐ Mixed Doubles
☐ Teams

Which division will you compete in badminton?	◯ Elite,national and former state players<br>◯ A/B grade competition players<br>◯ B/C grade competition players and social players
Which age group will you compete in badminton?	◯ 35+<br>◯ 45+<br>◯ 55+<br>◯ 65+
Which age group will you compete in baseball?	◯ Open (mixed) 35+<br>◯ Open (mixed) 40+<br>◯ Open (mixed) 45+<br>◯ Open (mixed) 50+<br>◯ Open (mixed) social: 35+
Which grade will you compete in basketaball?	◯ A Grade<br>◯ B Grade
Which age group will you compete in basketball?	◯ 30+<br>◯ 35+<br>◯ 40+<br>◯ 45+<br>◯ 50+<br>◯ 55+<br>◯ 60+<br>◯ 65+<br>◯ 70+<br>◯ 75+<br>◯ 30+ Intellectual disability<br>◯ 30+ Wheelchair<br>◯ Mixed: W30+/M30+
Which age group will you compete in basketball?	◯ 30+<br>◯ 35+<br>◯ 40+<br>◯ 45+<br>◯ 50+<br>◯ 55+<br>◯ 60+<br>◯ 30+ Intellectual disability<br>◯ 30+ Wheelchair<br>◯ Mixed: W30+/M30+

Which categories will you compete in beach volleyball?
(Tick all that apply)

☐ Men's pairs
☐ Mixed pairs
☐ Men's fours
☐ Mixed fours

Which categories will you compete in beach volleyball?
(Tick all that apply)

☐ Women's pairs
☐ Mixed pairs
☐ Women's fours
☐ Mixed fours

Which age group will you compete in beach volleyball?

◯ 30+
◯ 35+
◯ 40+
◯ 45+
◯ 50+
◯ 55+
◯ 60+

Which categories will you compete in BMX?
(Tick all that apply)

☐ 20''
☐ Cruiser
☐ Retro

Which age group will you compete in BMX?

◯ 30-34
◯ 35-39
◯ 40-44
◯ 45-49
◯ 50-54
◯ 50+

Which categories will you compete in bocce?
(Tick all that apply)

☐ Beach Bocce
☐ OZ Bocce Mixed Doubles

Which categories will you compete in boxing?

◯ under 49kg
◯ 49-52kg
◯ 56-60kg
◯ 60-64kg
◯ 64-69kg
◯ 69-75kg
◯ 75-81kg
◯ 81-91kg
◯ 91kg+

Which categories will you compete in boxing?

- ◯ under 48kg
- ◯ 48-51kg
- ◯ 51-54kg
- ◯ 54-57kg
- ◯ 57-60kg
- ◯ 60-64kg
- ◯ 64-69kg
- ◯ 69-75kg
- ◯ 75-81kg
- ◯ 81kg+

Which experience level will you complete in boxing?

- ◯ Novice: 0-5 bouts
- ◯ Intermediate: 6-15 bouts
- ◯ Open: 16 bouts or more

Which age group will you compete in boxing?

◯ 36-40 ◯ 41-45 ◯ 46-50
◯ 51-55 ◯ 56-60 ◯ 61+

Which categories will you compete in canoe/kayak/SUP?
(Tick all that apply)

- ☐ Sprint 200 metres
- ☐ Sprint 1000 metres
- ☐ Sprint 5000 metres
- ☐ Marathon
- ☐ Ocean Race16km

Which classes will you compete in 200 meters?
(Tick all that apply)

- ☐ MK1 - Men's single kayak
- ☐ MK2 - Men's double kayak
- ☐ XK2 - Double kayak, mixed gender
- ☐ MSUP - Men's stand-up paddleboard

Which classes will you compete in 200 meters?
(Tick all that apply)

- ☐ WK1 - Women's single kayak
- ☐ WK2 - Women's double kayak
- ☐ XK2 - Double kayak, mixed gender
- ☐ WSUP - Women's stand-up paddleboard

Which classes will you compete in 1000 meters?
(Tick all that apply)

- ☐ MK1 - Men's single kayak
- ☐ MK2 - Men's double kayak
- ☐ XK2 - Double kayak, mixed gender
- ☐ MSUP - Men's stand-up paddleboard

Which classes will you compete in 200 meters?
(Tick all that apply)

- ☐ WK1 - Women's single kayak
- ☐ WK2 - Women's double kayak
- ☐ XK2 - Double kayak, mixed gender
- ☐ WSUP - Women's stand-up paddleboard

Which classes will you compete in 5000 meters?
(Tick all that apply)

☐ MK1 - Men's single kayak
☐ MSUP - Men's stand-up paddleboard

Which classes will you compete in 200 meters?
(Tick all that apply)

☐ WK1 - Women's single kayak
☐ WSUP - Women's stand-up paddleboard

What age group will you compete in canoe marathon?

◯ Under 65 years
◯ 65+ years

Which classes will you compete in ocean race?
(Tick all that apply)

☐ MSurf - ski/ocean ski - men's single
☐ MOC1 - Outrigger canoe, men's single
☐ MOC2 - Outrigger canoe, men's double
☐ XOC2 - Outrigger canoe, double, mixed gender
☐ MSUP - Stand-up paddleboard, men's single

Which classes will you compete in ocean race?
(Tick all that apply)

☐ WSurf - ski/ocean ski - women's single
☐ WOC1 - Outrigger canoe, women's single
☐ WOC2 - Outrigger canoe, women's double
☐ XOC2 - Outrigger canoe, double, mixed gender
☐ WSUP - Stand-up paddleboard, women's single

What age group will you compete in canoe/kayak/SUP? ?

◯ 30-34
◯ 35-39
◯ 40-44
◯ 45-49
◯ 50-54
◯ 55-59
◯ 60-64
◯ 65-69
◯ 70-74
◯ 75-79
◯ 80+

Which categories will you compete in canoe-outrigger?
(Tick all that apply)

☐ OC6
☐ V12

What combined age group will you compete in canoe-outrigger?

☐ Male 240+
☐ Mixed 240+
☐ Male 300+
☐ Mixed 300+
☐ Male 360+
☐ Mixed 360+

What combined age group will you compete in canoe-outrigger?

☐ Female 240+
☐ Mixed 240+
☐ Female 300+
☐ Mixed 300+
☐ Female 360+
☐ Mixed 360+

Which age group will you compete in cricket?
◯ 30+
◯ 40+
◯ 50+
◯ 60+

Which categories will you compete in croquet?
(Tick all that apply)

☐ Association Croquet singles
☐ Association Croquet handicap doubles
☐ Golf Croquet open singles
☐ Golf Croquet 8+ singles
☐ Golf Croquet doubles

Which categories will you compete in cue sports?
(Tick all that apply)

☐ Billiards
☐ Snooker

Which age group will you compete in Billiards?
◯ 40-59
◯ 60+

Which age group will you compete in Snooker?
◯ 40-54
◯ 55-65
◯ 66+

Which categories will you compete in track cycling?
(Tick all that apply)

☐ Time trial - men
☐ Sprint 200m
☐ Team sprints 2 member teams
☐ Team sprints 3 member teams
☐ Pursuits 2000m
☐ Scratch races men 10km
☐ Scratch races men 5km

Which categories will you compete in track cycling?
(Tick all that apply)

☐ Time trial - women
☐ Sprint 200m
☐ Team sprints 2 member teams
☐ Team sprints 3 member teams
☐ Pursuits 2000m
☐ Scratch races women 5km

Which age group will you compete in track cycling?

- ◯ 30-34
- ◯ 35-39
- ◯ 40-44
- ◯ 45-49
- ◯ 50-54
- ◯ 55-59
- ◯ 60-64
- ◯ 65-69
- ◯ 70-74
- ◯ 75-79
- ◯ 80+

Which categories will you compete in road cycling?
(Tick all that apply)

- ☐ Time trial
- ☐ Road race- 32.2km
- ☐ Road race- 48.3km
- ☐ Road race- 64.4km

Which age group will you compete in road cycling?

- ◯ 30-34
- ◯ 35-39
- ◯ 40-44
- ◯ 45-49
- ◯ 50-54
- ◯ 55-59
- ◯ 60-64
- ◯ 65-69
- ◯ 70-74
- ◯ 75-79
- ◯ 80+

Which categories will you compete in darts?
(Tick all that apply)

- ☐ Male Singles
- ☐ Male Doubles
- ☐ Mixed Doubles
- ☐ Teams of 4 any mix of gender

Which categories will you compete in darts?
(Tick all that apply)

- ☐ Female Singles
- ☐ Female Doubles
- ☐ Mixed Doubles
- ☐ Teams of 4 any mix of gender

Which age group will you compete in darts- singles?

- ◯ 30-49
- ◯ 50-64
- ◯ 65+

Which age group will you compete in darts- doubles?

- ◯ 60-99
- ◯ 100-129
- ◯ 130+

Which age group will you compete in darts- teams?

- ◯ 120-200
- ◯ 201+

Which categories will you compete in dragon boat?
(Tick all that apply)

- ☐ 10's Crews open
- ☐ 10's Crews mixed
- ☐ 10's Crews open- all cancer survivors
- ☐ 20's Crews open
- ☐ 20's Crews mixed

Which categories will you compete in dragon boat?
(Tick all that apply)

- ☐ 10's Crews open
- ☐ 10's Crews women
- ☐ 10's Crews mixed
- ☐ 10's Crews open- all cancer survivors
- ☐ 20's Crews open
- ☐ 20's Crews women
- ☐ 20's Crews mixed

Which age group will you compete in dragon boat?

- ◯ 40+
- ◯ 50+
- ◯ 60+

Which age group will you compete in duathlon?

- ◯ 30-34
- ◯ 35-39
- ◯ 40-44
- ◯ 45-49
- ◯ 50-54
- ◯ 55-59
- ◯ 60-64
- ◯ 65-69
- ◯ 70-74
- ◯ 75-79
- ◯ 80+

Which categories will you compete in equestrian?
(Tick all that apply)

- ☐ 45cm
- ☐ 65cm
- ☐ 85cm Accumulator
- ☐ 1m
- ☐ Six bar
- ☐ 50cm Super two phase
- ☐ 70cm Scurry
- ☐ 90cm Super two phase
- ☐ 1.04m Table

Which age group will you compete in equestrian?

- ◯ 35-44
- ◯ 45-54
- ◯ 55-64
- ◯ 65+

Which age group will you compete in AFL?

- ◯ 30-49
- ◯ 50+

Which age group will you compete in soccer?	◯ Men 35+<br>◯ Men 40+<br>◯ Men 45+<br>◯ Men 50+<br>◯ Men 55+
Which age group will you compete in soccer?	◯ Women 30+<br>◯ Women 40+<br>◯ Women 50+
Which categories will you compete in futsal?<br>(Tick all that apply)<br><br>☐ Men<br>☐ Mixed	
Which age group will you compete in futsal?	◯ 35+<br>◯ 45+
Which age group will you compete in golf?	◯ 30-39<br>◯ 40-49<br>◯ 50-59<br>◯ 60-69<br>◯ 70+
Which categories will you compete in gymnastics?<br>(Tick all that apply)<br><br>☐ Men floor<br>☐ Men parallel bars<br>☐ Men horizontal bar<br>☐ Men vaultinng<br>☐ Men rings<br>☐ Men mini tramps<br>☐ Men pommels	
Which categories will you compete in gymnastics?<br>(Tick all that apply)<br><br>☐ Women floor<br>☐ Women uneven bar<br>☐ Women beam<br>☐ Women vaulting<br>☐ Women mini tramp	
Which division will you compete in gymnastics?	◯ Open-beginner/general/recreational<br>◯ Masters
Which age group will you compete in gymnastics?	◯ 20-29<br>◯ 30-39<br>◯ 40-49<br>◯ 50-59<br>◯ 60-69<br>◯ 70+

Which age group will you compete in hockey?	◯ 30+<br>◯ 35+<br>◯ 40+<br>◯ 45+<br>◯ 50+<br>◯ 55+<br>◯ 60+

Which categories will you compete in judo?
(Tick all that apply)

☐ Shiai
☐ Nage no Kata
☐ Katame no Kata
☐ Kime no Kata
☐ Goshin Jutsu
☐ Ju no Kata

Which age group will you compete in judo?	◯ 30-34<br>◯ 35-39<br>◯ 40-44<br>◯ 45-49<br>◯ 50-54<br>◯ 55-59<br>◯ 60-64<br>◯ 65-69<br>◯ 70+

Which categories will you compete in karate?
(Tick all that apply)

☐ Kumite
☐ Kata

Which age group will you compete in karate?	◯ 35-45<br>◯ 45-55<br>◯ 55+

Which categories will you compete in lawn bowls?
(Tick all that apply)

☐ Men's pairs
☐ Mixed pairs
☐ Mixed triples

Which categories will you compete in lawn bowls?
(Tick all that apply)

☐ Women's pairs
☐ Mixed pairs
☐ Mixed triples

Which age group will you compete in lawn bowls?	◯ 35+<br>◯ 55+

Which categories will you compete in motorsport?
(Tick all that apply)

☐ 3J improved production
☐ Sports sedans
☐ Rally cars
☐ 2F production cars
☐ Historic group N
☐ Historic group C
☐ Non-log booked cars
☐ HQ Holden

Which classes will you compete in 3J improved production?
(Tick all that apply)

☐ 0-1600
☐ 1600-2000
☐ 2000-3000
☐ 3000 and above
☐ 4WD

Which classes will you compete in sport sedans?
(Tick all that apply)

☐ 0-1600
☐ 1600-2000
☐ 2000-3000
☐ 3000 and above
☐ 4WD

Which classes will you compete in rally cars?
(Tick all that apply)

☐ 0-1600
☐ 1600-2000
☐ 2000-3000
☐ 3000 and above
☐ 4WD

Which classes will you compete in 2F production cars?
(Tick all that apply)

☐ 0-2000
☐ 2000 and over
☐ 4WD

Which classes will you compete in historic group N?
(Tick all that apply)

☐ 0-2000
☐ 2000 and over

Which classes will you compete in historic group C?
(Tick all that apply)

☐ 0-2000
☐ 2000 and over

Which classes will you compete in non-log booked cars?
(Tick all that apply)

☐ 0-2000
☐ 2000 and over
☐ 4WD

Which age group will you compete in mountain bike?

◯ 30-39
◯ 40-49
◯ 50-59
◯ 60-69
◯ 70+

Which age group will you compete in netball?

◯ 30+
◯ 35+
◯ 40+
◯ 45+
◯ 50+
◯ 55+
◯ 60+
◯ 65+

Which division will you compete in netball?

◯ Division 1
◯ Division 2

Which categories will you compete in pool lifesaving?
(Tick all that apply)

☐ 25m Manikin Carry (No Fins)
☐ 100m Obstacle Swim
☐ 50m Manikin Tow with Fins (& Tube)
☐ 50m Manikin Carry (with Fins)
☐ 12.5m Line Throw
☐ 4 x 25m Obstacle Relay - Mixed
☐ 4 x 25m Medley Relay - Mixed
☐ 4 x 25m Manikin Relay - Mixed

Which age group will you compete in individual pool lifesaving?

◯ 25-45
◯ 35-44
◯ 45-54
◯ 55-64
◯ 65+

Which age group will you compete in relay pool lifesaving?

◯ 120+
◯ 160+
◯ 200+
◯ 240+

Which categories will you compete in power lifting?
(Tick all that apply)

☐ Raw powerlifting
☐ Equipped powerlifting
☐ Raw bench press only
☐ Equipped bench press only
☐ Disabled raw powerlifting
☐ Disabled raw bench press only

Which class will you compete in power lifting?	◯ Men 59kg<br>◯ Men 66kg<br>◯ Men 74kg<br>◯ Men 83kg<br>◯ Men 93kg<br>◯ Men 105kg<br>◯ Men 120kg<br>◯ Men 120+kg
Which class will you compete in power lifting?	◯ Women 47kg<br>◯ Women 52kg<br>◯ Women 57kg<br>◯ Women 63kg<br>◯ Women 72kg<br>◯ Women 84kg<br>◯ Women 84+kg
Which age group will you compete in power lifting?	◯ 40-49<br>◯ 50-59<br>◯ 60-69<br>◯ 70+

Which categories will you compete in racquetball?
(Tick all that apply)

☐ Men's Singles
☐ Men's doubles
☐ Mixed doubles

Which categories will you compete in racquetball?
(Tick all that apply)

☐ Singles
☐ Women's doubles
☐ Mixed doubles

Which age group will you compete in racquetball?	◯ 30-39<br>◯ 40-49<br>◯ 50-59<br>◯ 60-69<br>◯ 70+

Which categories will you compete in rowing?
(Tick all that apply)

☐ 1x 1000m
☐ 2x 1000m
☐ 4x 1000m
☐ 2- 1000m
☐ 4+ 1000m
☐ 4- 1000m
☐ 8+ 1000m
☐ 1x 500m
☐ 2x 500m
☐ Mixed 2x
☐ Mixed 4x
☐ Mixed 4+
☐ Mixed 8+

Which age group will you compete in rowing?

◯ 27-35
◯ 36-42
◯ 43-49
◯ 50-54
◯ 55-59
◯ 60-64
◯ 65-69
◯ 70-74
◯ 75-79
◯ 80+

Which categories will you compete in rowing-indoor?
(Tick all that apply)

☐ Individual 500m
☐ Individual 1000m
☐ Individual 2000m
☐ Team mixed 2000m
☐ Para 500m
☐ Para 1000m
☐ Para 2000m

Which age group will you compete in rowing- indoor?

◯ 27-35
◯ 36-42
◯ 43-49
◯ 50-54
◯ 55-59
◯ 60-64
◯ 65-69
◯ 70-74
◯ 75-79
◯ 80+

Which age group will you compete in rugby?

◯ 35+
◯ 45+
◯ 55+

Which categories will you compete in shooting- clay target?
(Tick all that apply)

☐ 50 Target skeet
☐ 25 Pair double rise
☐ 45 Target champion of champions off 18m
☐ 50 Target double barrel
☐ 50 Target single barrel
☐ 50 Target point score

Which age group will you compete in shooting- clay target?

◯ 30-39
◯ 40-49
◯ 50-59
◯ 60-69
◯ 70-79
◯ 80+

Which categories will you compete in shooting- smallbore?
(Tick all that apply)

☐ 20m x 60
☐ 50m x 60
☐ 90m x 60

Which age group will you compete in shooting- smallbore?	◯ 30-49<br>◯ 50-59<br>◯ 60+
Which age group will you compete in shooting- filed rifle?	◯ 30-59<br>◯ 60-74<br>◯ 75+
Which grade will you compete in softball?	◯ A Grade - National or State equivalent levels<br>◯ B Grade - All others
Which age group will you compete in softball?	◯ 35+<br>◯ 45+
Which age group will you compete in squash?	◯ 30-39<br>◯ 40-44<br>◯ 45-49<br>◯ 50-54<br>◯ 55-59<br>◯ 60-64<br>◯ 65-69<br>◯ 70-74<br>◯ 75+

Which categories will you compete in swimming?
(Tick all that apply)

☐ 50 Free style
☐ 100 Free style
☐ 200 Free style
☐ 50 Breast style
☐ 100 Breast style
☐ 200 Breast style
☐ 50 Back style
☐ 100 Back style
☐ 200 Back style
☐ 50 Fly style
☐ 100 Fly style
☐ 200 Fly style
☐ 100 Ind. Medley style
☐ 200 Ind. Medley style
☐ 4x50 Free style Men
☐ 4x50 Free style Mixed
☐ 4x50 Medley style Men
☐ 4x50 Medley style Mixed

Which categories will you compete in swimming?
(Tick all that apply)

- ☐ 50 Free style
- ☐ 100 Free style
- ☐ 200 Free style
- ☐ 50 Breast style
- ☐ 100 Breast style
- ☐ 200 Breast style
- ☐ 50 Back style
- ☐ 100 Back style
- ☐ 200 Back style
- ☐ 50 Fly style
- ☐ 100 Fly style
- ☐ 200 Fly style
- ☐ 100 Ind. Medley style
- ☐ 200 Ind. Medley style
- ☐ 4x50 Free style Women
- ☐ 4x50 Free style Mixed
- ☐ 4x50 Medley style Women
- ☐ 4x50 Medley style Mixed

Which age group will you compete in swimming?

- ◯ 18-24
- ◯ 25-29
- ◯ 30-34
- ◯ 35-39
- ◯ 40-44
- ◯ 45-49
- ◯ 50-54
- ◯ 55-59
- ◯ 60-64
- ◯ 65-69
- ◯ 70-74
- ◯ 75-79
- ◯ 80-84
- ◯ 85-89
- ◯ 90+

Which age group will your team compete in swimming?

- ◯ 72-119
- ◯ 120-159
- ◯ 160-199
- ◯ 200-239
- ◯ 240-279
- ◯ 280-319
- ◯ 320-359

Which categories will you compete in table tennis?
(Tick all that apply)

- ☐ Male singles
- ☐ Male doubles
- ☐ Mixed doubles
- ☐ Teams

Which categories will you compete in table tennis?
(Tick all that apply)

- ☐ Female singles
- ☐ Female doubles
- ☐ Mixed doubles
- ☐ Teams

Which division will you compete in table tennis?	◯ Division 1- Experienced and competition players<br>◯ Division 2- Social and first year players
Which age group will you compete in table tennis?	◯ 30-49<br>◯ 50-64<br>◯ 65-74<br>◯ 75-79<br>◯ 80+
Which age group will your team compete in tabletennis?	◯ 30-49<br>◯ 50-64<br>◯ 65-74<br>◯ 75+

Which categories will you compete in taekwondo?
(Tick all that apply)

☐ International Tul
☐ World Poomsae
☐ Sparring international
☐ Sparring world
☐ Team patterns/forms
☐ Individual demonstration
☐ Team demonstration

Which class will you compete in taekwondo?	◯ under 60kgs<br>◯ 60-69kgs<br>◯ 70-79kgs<br>◯ 80-89kgs<br>◯ 90kg+
Which class will you compete in taekwondo?	◯ under 55kgs<br>◯ 55-65kgs<br>◯ 66-74kgs<br>◯ 75kgs+
Which age group will you compete in taekwondo?	◯ 30-39<br>◯ 40-49<br>◯ 50-59<br>◯ 60+

Which categories will you compete in tennis?
(Tick all that apply)

☐ Male Singles
☐ Men Doubles
☐ Combined age mixed doubles
☐ Combined age doubles

Which categories will you compete in tennis?
(Tick all that apply)

☐ Female Singles
☐ Women Doubles
☐ Combined age mixed doubles
☐ Combined age doubles

Which age group will you compete in tennis?	◯ 35+<br>◯ 40+<br>◯ 45+<br>◯ 50+<br>◯ 55+<br>◯ 60+<br>◯ 65+<br>◯ 70+<br>◯ 75+<br>◯ 80+
Which age group will your team compete in combined age doubles/mixed doubles?	◯ 70+<br>◯ 90+<br>◯ 110+<br>◯ 130+
Which categories will you compete in Ten Pin bowling? (Tick all that apply)<br><br>☐ Singles<br>☐ Mixed doubles<br>☐ Mixed teams of three<br>☐ 9 No Tap	
Which age group will you compete in Ten Pin bowling singles, including 9 NO TAP?	◯ 30-39<br>◯ 40-49<br>◯ 50-59<br>◯ 60-69<br>◯ 70+<br>◯ 9 NO TAP (30-49)<br>◯ 9 NO TAP (50+)<br>◯ Disabled (30-49)<br>◯ Disabled (50+)
Which age group will you compete in Ten Pin bowling doubles?	◯ 60-79<br>◯ 80-99<br>◯ 100-119<br>◯ 120-139<br>◯ 140+
Which age group will you compete in Ten Pin bowling teams?	◯ 90-119<br>◯ 120-149<br>◯ 150-179<br>◯ 180-209<br>◯ 210+
Which categories will you compete in touch football? (Tick all that apply)<br><br>☐ Men<br>☐ Mixed	
Which age group will you compete in Men touch football?	◯ 30+<br>◯ 35+<br>◯ 40+<br>◯ 45+<br>◯ 50+<br>◯ 55+

Which age group will you compete in Mixed touch football?	◯ W27+/M30+<br>◯ W35+/M40+<br>◯ W45+/M50+<br>◯ W55+/M60+
Which age group will you compete in Mixed touch football?	◯ W27+/M30+<br>◯ W35+/M40+<br>◯ W45+/M50+<br>◯ W55+/M60+
Which categories will you compete in trial running? (Tick all that apply)<br><br>☐ Individual<br>☐ Team mixed	
Which age group will you compete in individual trial running?	◯ 30-39<br>◯ 40-49<br>◯ 50-59<br>◯ 60+
Which age group will your team compete in mixed team trial running?	◯ 120+<br>◯ 160+<br>◯ 200+
Which categories will you compete in volleyball? (Tick all that apply)<br><br>☐ Mens<br>☐ Mixed	
Which categories will you compete in volleyball? (Tick all that apply)<br><br>☐ Womens<br>☐ Mixed	
Which age group will you compete in volleyball?	◯ 30+<br>◯ 35+<br>◯ 40+<br>◯ 45+<br>◯ 50+<br>◯ 55+<br>◯ 60+
Have you ever been an elite athlete? (elite athletes compete in non-Masters adult competition at state, national or international level)	◯ Yes<br>◯ No<br>◯ Don't know

At which level did you compete in for each sport in elite competition? (select up to 5 sports) Please select the HIGHEST level you ever completed in your career.

	State	National	International	Don't Know
Archery	○	○	○	○
Athletics	○	○	○	○
Badminton	○	○	○	○
Baseball	○	○	○	○
Basketball	○	○	○	○
Beach volleyball	○	○	○	○
BMX	○	○	○	○
Bocce	○	○	○	○
Boxing	○	○	○	○
Canoe/Kayak/SUP	○	○	○	○
Canoe outrigger	○	○	○	○
Cricket	○	○	○	○
Croquet	○	○	○	○
Cue sports	○	○	○	○
Cycling-track	○	○	○	○
Cycling-road	○	○	○	○
Darts	○	○	○	○
Dragon boat	○	○	○	○
Duathlon	○	○	○	○
Equestrian	○	○	○	○
Fly fishing	○	○	○	○
Football-AFL	○	○	○	○
Football-soccer	○	○	○	○
Futsal	○	○	○	○
Golf	○	○	○	○
Gymnastics	○	○	○	○
Hockey	○	○	○	○
Judo	○	○	○	○
Karate	○	○	○	○
Lawn bowls	○	○	○	○
Motorsport-hill climb	○	○	○	○
Mountain bike cross country	○	○	○	○
Netball	○	○	○	○
Pool lifesaving	○	○	○	○
Powerlifting	○	○	○	○
Racquetball	○	○	○	○
Rowing	○	○	○	○

Rowing-indoor	◯	◯	◯	◯
Rugby union	◯	◯	◯	◯
Sailing	◯	◯	◯	◯
Shooting clay target	◯	◯	◯	◯
Shooting smallbore and air rifle	◯	◯	◯	◯
Shooting field rifle	◯	◯	◯	◯
Softball	◯	◯	◯	◯
Squash	◯	◯	◯	◯
Swimming	◯	◯	◯	◯
Table tennis	◯	◯	◯	◯
Taekwondo	◯	◯	◯	◯
Tennis	◯	◯	◯	◯
Tenpin bowling	◯	◯	◯	◯
Touch football	◯	◯	◯	◯
Trail running	◯	◯	◯	◯
Volleyball	◯	◯	◯	◯
Others	◯	◯	◯	◯

---

please specify the sport/s ______________________________

These following four questions are to get an idea of your regular participation in *organised, *leisure time, *occupational (work), or *transport (active transport) activity across your adult years.

Did you participate in *organised activity?

Please tick the box for each age group.
For example, if you know you regularly participated in organised activities during your 40s and 50s, but can't recall for your 20s and 30s, please tick the box "Yes" for 40-49 and 50-59. If you don't know for any of the age groups, please select "don't know"

	Yes	No	Don't know
18-29 years old	◯	◯	◯
30-39 years old	◯	◯	◯
40-49 years old	◯	◯	◯
50-59 years old	◯	◯	◯
60-69 years old	◯	◯	◯
70 79 years old	◯	◯	◯
80-89 years old	◯	◯	◯
90-99 years old	◯	◯	◯

*Organised activities are those that are governed by a set of rules and/or undertaken competitively at any level (e.g. competition tennis or golf).

Did you participate in *leisure-time activity?

	Yes	No	Don't know
18-29 years old	○	○	○
30-39 years old	○	○	○
40-49 years old	○	○	○
50-59 years old	○	○	○
60-69 years old	○	○	○
70-79 years old	○	○	○
80-89 years old	○	○	○
90-99 years old	○	○	○

*Leisure-time activities are other non-competitive activities (e.g. walking, gardening.)

Did you participate in *occupational activity?

	Yes	No	Don't know
18-29 years old	○	○	○
30-39 years old	○	○	○
40-49 years old	○	○	○
50-59 years old	○	○	○
60-69 years old	○	○	○
70-79 years old	○	○	○
80-89 years old	○	○	○
90-99 years old	○	○	○

*Occupational activities are non-sedentary activities undertaken at work (e.g. deliveries using cycling transport, housework such as vacuuming).

Did you participate in *transport activity?

	Yes	No	Don't know
18-29 years old	◯	◯	◯
30-39 years old	◯	◯	◯
40-49 years old	◯	◯	◯
50-59 years old	◯	◯	◯
60-69 years old	◯	◯	◯
70-79 years old	◯	◯	◯
80-89 years old	◯	◯	◯
90-99 years old	◯	◯	◯

*Transport activity is activity undertaken to get to/from somewhere (e.g. cycling to work).

In the last 12 months, how many times a week (on average) have you done the following training?

	Daily	5-6 times/week	3-4 times/week	1-2 times/week	0 times/week
Aerobic training	○	○	○	○	○
Anaerobic training	○	○	○	○	○
Resistance training	○	○	○	○	○

In the last 12 months, how much time per week (on average) did you spend in the following training?

	< 1hour	1-2 hours/week	2-3 hours/week	3-4 hours/week	>4 hours/week
Aerobic training	◯	◯	◯	◯	◯
Anaerobic training	◯	◯	◯	◯	◯
Resistance training	◯	◯	◯	◯	◯

Do you currently smoke tobacco?

◯ Yes. Daily
◯ Yes. Less than daily
◯ No. I am an ex-smoker
◯ No. I have never smoked

On average, how many cigarettes do you smoke each week?

◯ 1
◯ 2
◯ 3
◯ 4
◯ 5
◯ 6
◯ 7
◯ 8
◯ 9
◯ 10
◯ 11
◯ 12
◯ 13
◯ 14
◯ 15
◯ 16
◯ 17
◯ 18
◯ 19
◯ 20
◯ 21
◯ 22
◯ 23
◯ 24
◯ 25
◯ 26
◯ 27
◯ 28
◯ 29
◯ 30
◯ 31
◯ 32
◯ 33
◯ 34
◯ 35
◯ 36
◯ 37
◯ 38
◯ 39
◯ 40
◯ 41
◯ 42
◯ 43
◯ 44
◯ 45
◯ 46
◯ 47
◯ 48
◯ 49
◯ 50
◯ 51
◯ 52
◯ 53
◯ 54
◯ 55
◯ 56
◯ 57
◯ 58
◯ 59
◯ 60
◯ 61
◯ 62
◯ 63
◯ 64
◯ 65
◯ 66
◯ 67
◯ 68
◯ 69

	◯ 70<br>◯ 71<br>◯ 72<br>◯ 73<br>◯ 74<br>◯ 75<br>◯ 76<br>◯ 77<br>◯ 78<br>◯ 79<br>◯ 80<br>◯ 81<br>◯ 82<br>◯ 83<br>◯ 84<br>◯ 85<br>◯ 86<br>◯ 87<br>◯ 88<br>◯ 89<br>◯ 90<br>◯ 91<br>◯ 92<br>◯ 93<br>◯ 94<br>◯ 95<br>◯ 96<br>◯ 97<br>◯ 98<br>◯ 99<br>◯ 100<br>◯ >100<br>◯ Don't know
How long have you been smoking for?	◯ < 1 year<br>◯ 1-5 years<br>◯ 6-10 years<br>◯ >10 years<br>◯ Don't know
How long did you smoke for?	◯ < 1 year<br>◯ 1-5 years<br>◯ 6-10 years<br>◯ >10 years<br>◯ Don't know
Do you currently drink alcohol at least once per week?	◯ Yes<br>◯ No
On average how many *standard drinks do you have per week?	◯ < 1<br>◯ 1-4<br>◯ 5-7<br>◯ 7-10<br>◯ 10-14<br>◯ >14<br>◯ Prefer not to say<br>◯ Don't know<br>(List of common standard drinks is shown below)

*List of approximate number of common standard drinks:
Full strength 4.8% alc. vol:
285 ml glass - 1.1 standard drinks
375 ml bottle or can - 1.4 standard drinks
Mid strength 3.5% alc. vol:
285 ml glass - 0.8 standard drinks
375 ml bottle or can - 1 standard drink
Low strength 2.7% alc. vol:
285 ml glass - 0.6 standard drinks
375 ml bottle or can - 0.8 standard drinks
Red wine: 150 ml average restaurant serving - 1.5 standard drinks
White wine OR Champagne: 150 ml average restaurant serving - 1.4 standard drinks
Spirits: 30 ml nip - 1 standard drink
Full strength pre-mix spirits: 375 ml can - 1.5 standard drinks

---

Did you ever drink regularly, that is, at least once a week?

- ◯ Yes
- ◯ No

On average how many *standard drinks did you have per week?

- ◯ 1-4
- ◯ 5-7
- ◯ 7-10
- ◯ 10-14
- ◯ >14
- ◯ Prefer not to say
- ◯ Don't know

(List of common standard drinks is shown below)

---

*List of approximate number of common standard drinks:
Full strength 4.8% alc. vol:
285 ml glass - 1.1 standard drinks
375 ml bottle or can - 1.4 standard drinks
Mid strength 3.5% alc. vol:
285 ml glass - 0.8 standard drinks
375 ml bottle or can - 1 standard drink
Low strength 2.7% alc. vol:
285 ml glass - 0.6 standard drinks
375 ml bottle or can - 0.8 standard drinks
Red wine: 150 ml average restaurant serving - 1.5 standard drinks
White wine OR Champagne: 150 ml average restaurant serving - 1.4 standard drinks
Spirits: 30 ml nip - 1 standard drink
Full strength pre-mix spirits: 375 ml can - 1.5 standard drinks

What is your current height in cm? (an approximate height is OK if you don't know exactly)

◯ < 140
◯ 140
◯ 141
◯ 142
◯ 143
◯ 144
◯ 145
◯ 146
◯ 147
◯ 148
◯ 149
◯ 150
◯ 151
◯ 152
◯ 153
◯ 154
◯ 155
◯ 156
◯ 157
◯ 158
◯ 159
◯ 160
◯ 161
◯ 162
◯ 163
◯ 164
◯ 165
◯ 166
◯ 167
◯ 168
◯ 169
◯ 170
◯ 171
◯ 172
◯ 173
◯ 174
◯ 175
◯ 176
◯ 177
◯ 178
◯ 179
◯ 180
◯ 181
◯ 182
◯ 183
◯ 184
◯ 185
◯ 186
◯ 187
◯ 188
◯ 189
◯ 190
◯ 191
◯ 192
◯ 193
◯ 194
◯ 195
◯ 196
◯ 197
◯ 198
◯ 199
◯ 200
◯ 201
◯ 202
◯ 203
◯ 204
◯ 205
◯ 206
◯ 207

	◯ 208<br>◯ 209<br>◯ 210<br>◯ Don't know
Do you consider yourself to be an acceptable weight, underweight or overweight?	◯ Acceptable weight<br>◯ Underweight<br>◯ Overweight<br>◯ Don't Know

What is your current weight in kg? (an approximate weight is OK if you don't know exactly)

- ◯ < 40
- ◯ 40
- ◯ 41
- ◯ 42
- ◯ 43
- ◯ 44
- ◯ 45
- ◯ 46
- ◯ 47
- ◯ 48
- ◯ 49
- ◯ 50
- ◯ 51
- ◯ 52
- ◯ 53
- ◯ 54
- ◯ 55
- ◯ 56
- ◯ 57
- ◯ 58
- ◯ 59
- ◯ 60
- ◯ 61
- ◯ 62
- ◯ 63
- ◯ 64
- ◯ 65
- ◯ 66
- ◯ 67
- ◯ 68
- ◯ 69
- ◯ 70
- ◯ 71
- ◯ 72
- ◯ 73
- ◯ 74
- ◯ 75
- ◯ 76
- ◯ 77
- ◯ 78
- ◯ 79
- ◯ 80
- ◯ 81
- ◯ 82
- ◯ 83
- ◯ 84
- ◯ 85
- ◯ 86
- ◯ 87
- ◯ 88
- ◯ 89
- ◯ 90
- ◯ 91
- ◯ 92
- ◯ 93
- ◯ 94
- ◯ 95
- ◯ 96
- ◯ 97
- ◯ 98
- ◯ 99
- ◯ 100
- ◯ 101
- ◯ 102
- ◯ 103
- ◯ 104
- ◯ 105
- ◯ 106
- ◯ 107

- ◯ 108
- ◯ 109
- ◯ 110
- ◯ 111
- ◯ 112
- ◯ 113
- ◯ 114
- ◯ 115
- ◯ 116
- ◯ 117
- ◯ 118
- ◯ 119
- ◯ 120
- ◯ 121
- ◯ 122
- ◯ 123
- ◯ 124
- ◯ 125
- ◯ 126
- ◯ 127
- ◯ 128
- ◯ 129
- ◯ 130
- ◯ 131
- ◯ 132
- ◯ 133
- ◯ 134
- ◯ 135
- ◯ 136
- ◯ 137
- ◯ 138
- ◯ 139
- ◯ 140
- ◯ 141
- ◯ 142
- ◯ 143
- ◯ 144
- ◯ 145
- ◯ 146
- ◯ 147
- ◯ 148
- ◯ 149
- ◯ 150
- ◯ 151
- ◯ 152
- ◯ 153
- ◯ 154
- ◯ 155
- ◯ 156
- ◯ 157
- ◯ 158
- ◯ 159
- ◯ 160
- ◯ 161
- ◯ 162
- ◯ 163
- ◯ 164
- ◯ 165
- ◯ 166
- ◯ 167
- ◯ 168
- ◯ 169
- ◯ 170
- ◯ 171
- ◯ 172
- ◯ 173
- ◯ 174
- ◯ 175
- ◯ 176
- ◯ 177
- ◯ 178

	◯ 179<br>◯ 180<br>◯ 181<br>◯ 182<br>◯ 183<br>◯ 184<br>◯ 185<br>◯ 186<br>◯ 187<br>◯ 188<br>◯ 189<br>◯ 190<br>◯ 191<br>◯ 192<br>◯ 193<br>◯ 194<br>◯ 195<br>◯ 196<br>◯ 197<br>◯ 198<br>◯ 199<br>◯ 200<br>◯ Don't know
Are either of your parents, or any of brothers or sisters overweight or obese?	◯ Yes<br>◯ No<br>◯ Don't know
Including any conditions which can be controlled with medication, have you ever been told by a doctor (GP or specialist) or nurse that you have any heart or circulatory conditions?	◯ Yes<br>◯ No<br>◯ Don't know

What are the names of this/these condition/s?
(Tick all that apply)

☐ Angina
☐ Fluid problems/fluid retention/oedema
☐ Haemorrhoids
☐ Hardening of the arteries/atherosclerosis/arteriosclerosis
☐ Heart attack
☐ Heart failure
☐ Heart murmur/heart valve disorder
☐ High blood pressure/ hypertension
☐ High cholesterol
☐ Low blood pressure/hypotension
☐ Rapid or irregular heartbeats/tachycardia/palpitations
☐ Rheumatic heart disease
☐ Stroke (including after effects of stroke)
☐ Varicose veins
☐ Other (please specify)
☐ Don't know

please specify the other heart condition ____________________

Do you have another heart of circulatory condition need to be specified?

◯ Yes ◯ No

please specify the second heart condition ____________________

Do you have another heart of circulatory condition need to be specified?

◯ Yes ◯ No

please specify the third heart condition

____

At what age were you first told you had Rheumatic heart disease?

◯ 0
◯ 1
◯ 2
◯ 3
◯ 4
◯ 5
◯ 6
◯ 7
◯ 8
◯ 9
◯ 10
◯ 11
◯ 12
◯ 13
◯ 14
◯ 15
◯ 16
◯ 17
◯ 18
◯ 19
◯ 20
◯ 21
◯ 22
◯ 23
◯ 24
◯ 25
◯ 26
◯ 27
◯ 28
◯ 29
◯ 30
◯ 31
◯ 32
◯ 33
◯ 34
◯ 35
◯ 36
◯ 37
◯ 38
◯ 39
◯ 40
◯ 41
◯ 42
◯ 43
◯ 44
◯ 45
◯ 46
◯ 47
◯ 48
◯ 49
◯ 50
◯ 51
◯ 52
◯ 53
◯ 54
◯ 55
◯ 56
◯ 57
◯ 58
◯ 59
◯ 60
◯ 61
◯ 62
◯ 63
◯ 64
◯ 65
◯ 66
◯ 67
◯ 68

- 69
- 70
- 71
- 72
- 73
- 74
- 75
- 76
- 77
- 78
- 79
- 80
- 81
- 82
- 83
- 84
- 85
- 86
- 87
- 88
- 89
- 90
- 91
- 92
- 93
- 94
- 95
- 96
- 97
- 98
- 99
- Don't know

At what age were you first told you had heart attack?

◯ 0
◯ 1
◯ 2
◯ 3
◯ 4
◯ 5
◯ 6
◯ 7
◯ 8
◯ 9
◯ 10
◯ 11
◯ 12
◯ 13
◯ 14
◯ 15
◯ 16
◯ 17
◯ 18
◯ 19
◯ 20
◯ 21
◯ 22
◯ 23
◯ 24
◯ 25
◯ 26
◯ 27
◯ 28
◯ 29
◯ 30
◯ 31
◯ 32
◯ 33
◯ 34
◯ 35
◯ 36
◯ 37
◯ 38
◯ 39
◯ 40
◯ 41
◯ 42
◯ 43
◯ 44
◯ 45
◯ 46
◯ 47
◯ 48
◯ 49
◯ 50
◯ 51
◯ 52
◯ 53
◯ 54
◯ 55
◯ 56
◯ 57
◯ 58
◯ 59
◯ 60
◯ 61
◯ 62
◯ 63
◯ 64
◯ 65
◯ 66
◯ 67
◯ 68

◯ 69
◯ 70
◯ 71
◯ 72
◯ 73
◯ 74
◯ 75
◯ 76
◯ 77
◯ 78
◯ 79
◯ 80
◯ 81
◯ 82
◯ 83
◯ 84
◯ 85
◯ 86
◯ 87
◯ 88
◯ 89
◯ 90
◯ 91
◯ 92
◯ 93
◯ 94
◯ 95
◯ 96
◯ 97
◯ 98
◯ 99
◯ Don't know

At what age were you first told you had heart failure?

◯ 0
◯ 1
◯ 2
◯ 3
◯ 4
◯ 5
◯ 6
◯ 7
◯ 8
◯ 9
◯ 10
◯ 11
◯ 12
◯ 13
◯ 14
◯ 15
◯ 16
◯ 17
◯ 18
◯ 19
◯ 20
◯ 21
◯ 22
◯ 23
◯ 24
◯ 25
◯ 26
◯ 27
◯ 28
◯ 29
◯ 30
◯ 31
◯ 32
◯ 33
◯ 34
◯ 35
◯ 36
◯ 37
◯ 38
◯ 39
◯ 40
◯ 41
◯ 42
◯ 43
◯ 44
◯ 45
◯ 46
◯ 47
◯ 48
◯ 49
◯ 50
◯ 51
◯ 52
◯ 53
◯ 54
◯ 55
◯ 56
◯ 57
◯ 58
◯ 59
◯ 60
◯ 61
◯ 62
◯ 63
◯ 64
◯ 65
◯ 66
◯ 67
◯ 68

◯ 69
◯ 70
◯ 71
◯ 72
◯ 73
◯ 74
◯ 75
◯ 76
◯ 77
◯ 78
◯ 79
◯ 80
◯ 81
◯ 82
◯ 83
◯ 84
◯ 85
◯ 86
◯ 87
◯ 88
◯ 89
◯ 90
◯ 91
◯ 92
◯ 93
◯ 94
◯ 95
◯ 96
◯ 97
◯ 98
◯ 99
◯ Don't know

At what age were you first told you had stroke (including after effects of stoke)?

◯ 0
◯ 1
◯ 2
◯ 3
◯ 4
◯ 5
◯ 6
◯ 7
◯ 8
◯ 9
◯ 10
◯ 11
◯ 12
◯ 13
◯ 14
◯ 15
◯ 16
◯ 17
◯ 18
◯ 19
◯ 20
◯ 21
◯ 22
◯ 23
◯ 24
◯ 25
◯ 26
◯ 27
◯ 28
◯ 29
◯ 30
◯ 31
◯ 32
◯ 33
◯ 34
◯ 35
◯ 36
◯ 37
◯ 38
◯ 39
◯ 40
◯ 41
◯ 42
◯ 43
◯ 44
◯ 45
◯ 46
◯ 47
◯ 48
◯ 49
◯ 50
◯ 51
◯ 52
◯ 53
◯ 54
◯ 55
◯ 56
◯ 57
◯ 58
◯ 59
◯ 60
◯ 61
◯ 62
◯ 63
◯ 64
◯ 65
◯ 66
◯ 67
◯ 68

◯ 69
◯ 70
◯ 71
◯ 72
◯ 73
◯ 74
◯ 75
◯ 76
◯ 77
◯ 78
◯ 79
◯ 80
◯ 81
◯ 82
◯ 83
◯ 84
◯ 85
◯ 86
◯ 87
◯ 88
◯ 89
◯ 90
◯ 91
◯ 92
◯ 93
◯ 94
◯ 95
◯ 96
◯ 97
◯ 98
◯ 99
◯ Don't know

At what age were you first told you had angina?

◯ 0
◯ 1
◯ 2
◯ 3
◯ 4
◯ 5
◯ 6
◯ 7
◯ 8
◯ 9
◯ 10
◯ 11
◯ 12
◯ 13
◯ 14
◯ 15
◯ 16
◯ 17
◯ 18
◯ 19
◯ 20
◯ 21
◯ 22
◯ 23
◯ 24
◯ 25
◯ 26
◯ 27
◯ 28
◯ 29
◯ 30
◯ 31
◯ 32
◯ 33
◯ 34
◯ 35
◯ 36
◯ 37
◯ 38
◯ 39
◯ 40
◯ 41
◯ 42
◯ 43
◯ 44
◯ 45
◯ 46
◯ 47
◯ 48
◯ 49
◯ 50
◯ 51
◯ 52
◯ 53
◯ 54
◯ 55
◯ 56
◯ 57
◯ 58
◯ 59
◯ 60
◯ 61
◯ 62
◯ 63
◯ 64
◯ 65
◯ 66
◯ 67
◯ 68

- ◯ 69
- ◯ 70
- ◯ 71
- ◯ 72
- ◯ 73
- ◯ 74
- ◯ 75
- ◯ 76
- ◯ 77
- ◯ 78
- ◯ 79
- ◯ 80
- ◯ 81
- ◯ 82
- ◯ 83
- ◯ 84
- ◯ 85
- ◯ 86
- ◯ 87
- ◯ 88
- ◯ 89
- ◯ 90
- ◯ 91
- ◯ 92
- ◯ 93
- ◯ 94
- ◯ 95
- ◯ 96
- ◯ 97
- ◯ 98
- ◯ 99
- ◯ Don't know

At what age were you first told you had high blood pressure/hypertension?

◯ 0
◯ 1
◯ 2
◯ 3
◯ 4
◯ 5
◯ 6
◯ 7
◯ 8
◯ 9
◯ 10
◯ 11
◯ 12
◯ 13
◯ 14
◯ 15
◯ 16
◯ 17
◯ 18
◯ 19
◯ 20
◯ 21
◯ 22
◯ 23
◯ 24
◯ 25
◯ 26
◯ 27
◯ 28
◯ 29
◯ 30
◯ 31
◯ 32
◯ 33
◯ 34
◯ 35
◯ 36
◯ 37
◯ 38
◯ 39
◯ 40
◯ 41
◯ 42
◯ 43
◯ 44
◯ 45
◯ 46
◯ 47
◯ 48
◯ 49
◯ 50
◯ 51
◯ 52
◯ 53
◯ 54
◯ 55
◯ 56
◯ 57
◯ 58
◯ 59
◯ 60
◯ 61
◯ 62
◯ 63
◯ 64
◯ 65
◯ 66
◯ 67
◯ 68

◯ 69
◯ 70
◯ 71
◯ 72
◯ 73
◯ 74
◯ 75
◯ 76
◯ 77
◯ 78
◯ 79
◯ 80
◯ 81
◯ 82
◯ 83
◯ 84
◯ 85
◯ 86
◯ 87
◯ 88
◯ 89
◯ 90
◯ 91
◯ 92
◯ 93
◯ 94
◯ 95
◯ 96
◯ 97
◯ 98
◯ 99
◯ Don't know

At what age were you first told you had low blood pressure/hypotension

- ◯ 0
- ◯ 1
- ◯ 2
- ◯ 3
- ◯ 4
- ◯ 5
- ◯ 6
- ◯ 7
- ◯ 8
- ◯ 9
- ◯ 10
- ◯ 11
- ◯ 12
- ◯ 13
- ◯ 14
- ◯ 15
- ◯ 16
- ◯ 17
- ◯ 18
- ◯ 19
- ◯ 20
- ◯ 21
- ◯ 22
- ◯ 23
- ◯ 24
- ◯ 25
- ◯ 26
- ◯ 27
- ◯ 28
- ◯ 29
- ◯ 30
- ◯ 31
- ◯ 32
- ◯ 33
- ◯ 34
- ◯ 35
- ◯ 36
- ◯ 37
- ◯ 38
- ◯ 39
- ◯ 40
- ◯ 41
- ◯ 42
- ◯ 43
- ◯ 44
- ◯ 45
- ◯ 46
- ◯ 47
- ◯ 48
- ◯ 49
- ◯ 50
- ◯ 51
- ◯ 52
- ◯ 53
- ◯ 54
- ◯ 55
- ◯ 56
- ◯ 57
- ◯ 58
- ◯ 59
- ◯ 60
- ◯ 61
- ◯ 62
- ◯ 63
- ◯ 64
- ◯ 65
- ◯ 66
- ◯ 67
- ◯ 68

- ◯ 69
- ◯ 70
- ◯ 71
- ◯ 72
- ◯ 73
- ◯ 74
- ◯ 75
- ◯ 76
- ◯ 77
- ◯ 78
- ◯ 79
- ◯ 80
- ◯ 81
- ◯ 82
- ◯ 83
- ◯ 84
- ◯ 85
- ◯ 86
- ◯ 87
- ◯ 88
- ◯ 89
- ◯ 90
- ◯ 91
- ◯ 92
- ◯ 93
- ◯ 94
- ◯ 95
- ◯ 96
- ◯ 97
- ◯ 98
- ◯ 99
- ◯ Don't know

At what age were you first told you had hardening of the arteries/atherosclerosis/arteriosclerosis?

〇 0
〇 1
〇 2
〇 3
〇 4
〇 5
〇 6
〇 7
〇 8
〇 9
〇 10
〇 11
〇 12
〇 13
〇 14
〇 15
〇 16
〇 17
〇 18
〇 19
〇 20
〇 21
〇 22
〇 23
〇 24
〇 25
〇 26
〇 27
〇 28
〇 29
〇 30
〇 31
〇 32
〇 33
〇 34
〇 35
〇 36
〇 37
〇 38
〇 39
〇 40
〇 41
〇 42
〇 43
〇 44
〇 45
〇 46
〇 47
〇 48
〇 49
〇 50
〇 51
〇 52
〇 53
〇 54
〇 55
〇 56
〇 57
〇 58
〇 59
〇 60
〇 61
〇 62
〇 63
〇 64
〇 65
〇 66
〇 67
〇 68

◯ 69
◯ 70
◯ 71
◯ 72
◯ 73
◯ 74
◯ 75
◯ 76
◯ 77
◯ 78
◯ 79
◯ 80
◯ 81
◯ 82
◯ 83
◯ 84
◯ 85
◯ 86
◯ 87
◯ 88
◯ 89
◯ 90
◯ 91
◯ 92
◯ 93
◯ 94
◯ 95
◯ 96
◯ 97
◯ 98
◯ 99
◯ Don't know

At what age were you first told you had fluid problems/fluid retention/oedema?

◯ 0
◯ 1
◯ 2
◯ 3
◯ 4
◯ 5
◯ 6
◯ 7
◯ 8
◯ 9
◯ 10
◯ 11
◯ 12
◯ 13
◯ 14
◯ 15
◯ 16
◯ 17
◯ 18
◯ 19
◯ 20
◯ 21
◯ 22
◯ 23
◯ 24
◯ 25
◯ 26
◯ 27
◯ 28
◯ 29
◯ 30
◯ 31
◯ 32
◯ 33
◯ 34
◯ 35
◯ 36
◯ 37
◯ 38
◯ 39
◯ 40
◯ 41
◯ 42
◯ 43
◯ 44
◯ 45
◯ 46
◯ 47
◯ 48
◯ 49
◯ 50
◯ 51
◯ 52
◯ 53
◯ 54
◯ 55
◯ 56
◯ 57
◯ 58
◯ 59
◯ 60
◯ 61
◯ 62
◯ 63
◯ 64
◯ 65
◯ 66
◯ 67
◯ 68

◯ 69
◯ 70
◯ 71
◯ 72
◯ 73
◯ 74
◯ 75
◯ 76
◯ 77
◯ 78
◯ 79
◯ 80
◯ 81
◯ 82
◯ 83
◯ 84
◯ 85
◯ 86
◯ 87
◯ 88
◯ 89
◯ 90
◯ 91
◯ 92
◯ 93
◯ 94
◯ 95
◯ 96
◯ 97
◯ 98
◯ 99
◯ Don't know

At what age were you first told you had high cholesterol?

- ◯ 0
- ◯ 1
- ◯ 2
- ◯ 3
- ◯ 4
- ◯ 5
- ◯ 6
- ◯ 7
- ◯ 8
- ◯ 9
- ◯ 10
- ◯ 11
- ◯ 12
- ◯ 13
- ◯ 14
- ◯ 15
- ◯ 16
- ◯ 17
- ◯ 18
- ◯ 19
- ◯ 20
- ◯ 21
- ◯ 22
- ◯ 23
- ◯ 24
- ◯ 25
- ◯ 26
- ◯ 27
- ◯ 28
- ◯ 29
- ◯ 30
- ◯ 31
- ◯ 32
- ◯ 33
- ◯ 34
- ◯ 35
- ◯ 36
- ◯ 37
- ◯ 38
- ◯ 39
- ◯ 40
- ◯ 41
- ◯ 42
- ◯ 43
- ◯ 44
- ◯ 45
- ◯ 46
- ◯ 47
- ◯ 48
- ◯ 49
- ◯ 50
- ◯ 51
- ◯ 52
- ◯ 53
- ◯ 54
- ◯ 55
- ◯ 56
- ◯ 57
- ◯ 58
- ◯ 59
- ◯ 60
- ◯ 61
- ◯ 62
- ◯ 63
- ◯ 64
- ◯ 65
- ◯ 66
- ◯ 67
- ◯ 68

◯ 69
◯ 70
◯ 71
◯ 72
◯ 73
◯ 74
◯ 75
◯ 76
◯ 77
◯ 78
◯ 79
◯ 80
◯ 81
◯ 82
◯ 83
◯ 84
◯ 85
◯ 86
◯ 87
◯ 88
◯ 89
◯ 90
◯ 91
◯ 92
◯ 93
◯ 94
◯ 95
◯ 96
◯ 97
◯ 98
◯ 99
◯ Don't know

At what age were you first told you had rapid or irregular heartbeats/tachycardia/palpitations?

- ◯ 0
- ◯ 1
- ◯ 2
- ◯ 3
- ◯ 4
- ◯ 5
- ◯ 6
- ◯ 7
- ◯ 8
- ◯ 9
- ◯ 10
- ◯ 11
- ◯ 12
- ◯ 13
- ◯ 14
- ◯ 15
- ◯ 16
- ◯ 17
- ◯ 18
- ◯ 19
- ◯ 20
- ◯ 21
- ◯ 22
- ◯ 23
- ◯ 24
- ◯ 25
- ◯ 26
- ◯ 27
- ◯ 28
- ◯ 29
- ◯ 30
- ◯ 31
- ◯ 32
- ◯ 33
- ◯ 34
- ◯ 35
- ◯ 36
- ◯ 37
- ◯ 38
- ◯ 39
- ◯ 40
- ◯ 41
- ◯ 42
- ◯ 43
- ◯ 44
- ◯ 45
- ◯ 46
- ◯ 47
- ◯ 48
- ◯ 49
- ◯ 50
- ◯ 51
- ◯ 52
- ◯ 53
- ◯ 54
- ◯ 55
- ◯ 56
- ◯ 57
- ◯ 58
- ◯ 59
- ◯ 60
- ◯ 61
- ◯ 62
- ◯ 63
- ◯ 64
- ◯ 65
- ◯ 66
- ◯ 67
- ◯ 68

- ◯ 69
- ◯ 70
- ◯ 71
- ◯ 72
- ◯ 73
- ◯ 74
- ◯ 75
- ◯ 76
- ◯ 77
- ◯ 78
- ◯ 79
- ◯ 80
- ◯ 81
- ◯ 82
- ◯ 83
- ◯ 84
- ◯ 85
- ◯ 86
- ◯ 87
- ◯ 88
- ◯ 89
- ◯ 90
- ◯ 91
- ◯ 92
- ◯ 93
- ◯ 94
- ◯ 95
- ◯ 96
- ◯ 97
- ◯ 98
- ◯ 99
- ◯ Don't know

At what age were you first told you had heart murmur/heart valve disorder?

- ◯ 0
- ◯ 1
- ◯ 2
- ◯ 3
- ◯ 4
- ◯ 5
- ◯ 6
- ◯ 7
- ◯ 8
- ◯ 9
- ◯ 10
- ◯ 11
- ◯ 12
- ◯ 13
- ◯ 14
- ◯ 15
- ◯ 16
- ◯ 17
- ◯ 18
- ◯ 19
- ◯ 20
- ◯ 21
- ◯ 22
- ◯ 23
- ◯ 24
- ◯ 25
- ◯ 26
- ◯ 27
- ◯ 28
- ◯ 29
- ◯ 30
- ◯ 31
- ◯ 32
- ◯ 33
- ◯ 34
- ◯ 35
- ◯ 36
- ◯ 37
- ◯ 38
- ◯ 39
- ◯ 40
- ◯ 41
- ◯ 42
- ◯ 43
- ◯ 44
- ◯ 45
- ◯ 46
- ◯ 47
- ◯ 48
- ◯ 49
- ◯ 50
- ◯ 51
- ◯ 52
- ◯ 53
- ◯ 54
- ◯ 55
- ◯ 56
- ◯ 57
- ◯ 58
- ◯ 59
- ◯ 60
- ◯ 61
- ◯ 62
- ◯ 63
- ◯ 64
- ◯ 65
- ◯ 66
- ◯ 67
- ◯ 68

- ◯ 69
- ◯ 70
- ◯ 71
- ◯ 72
- ◯ 73
- ◯ 74
- ◯ 75
- ◯ 76
- ◯ 77
- ◯ 78
- ◯ 79
- ◯ 80
- ◯ 81
- ◯ 82
- ◯ 83
- ◯ 84
- ◯ 85
- ◯ 86
- ◯ 87
- ◯ 88
- ◯ 89
- ◯ 90
- ◯ 91
- ◯ 92
- ◯ 93
- ◯ 94
- ◯ 95
- ◯ 96
- ◯ 97
- ◯ 98
- ◯ 99
- ◯ Don't know

At what age were you first told you had haemorrhoids?

- ◯ 0
- ◯ 1
- ◯ 2
- ◯ 3
- ◯ 4
- ◯ 5
- ◯ 6
- ◯ 7
- ◯ 8
- ◯ 9
- ◯ 10
- ◯ 11
- ◯ 12
- ◯ 13
- ◯ 14
- ◯ 15
- ◯ 16
- ◯ 17
- ◯ 18
- ◯ 19
- ◯ 20
- ◯ 21
- ◯ 22
- ◯ 23
- ◯ 24
- ◯ 25
- ◯ 26
- ◯ 27
- ◯ 28
- ◯ 29
- ◯ 30
- ◯ 31
- ◯ 32
- ◯ 33
- ◯ 34
- ◯ 35
- ◯ 36
- ◯ 37
- ◯ 38
- ◯ 39
- ◯ 40
- ◯ 41
- ◯ 42
- ◯ 43
- ◯ 44
- ◯ 45
- ◯ 46
- ◯ 47
- ◯ 48
- ◯ 49
- ◯ 50
- ◯ 51
- ◯ 52
- ◯ 53
- ◯ 54
- ◯ 55
- ◯ 56
- ◯ 57
- ◯ 58
- ◯ 59
- ◯ 60
- ◯ 61
- ◯ 62
- ◯ 63
- ◯ 64
- ◯ 65
- ◯ 66
- ◯ 67
- ◯ 68

○ 69
○ 70
○ 71
○ 72
○ 73
○ 74
○ 75
○ 76
○ 77
○ 78
○ 79
○ 80
○ 81
○ 82
○ 83
○ 84
○ 85
○ 86
○ 87
○ 88
○ 89
○ 90
○ 91
○ 92
○ 93
○ 94
○ 95
○ 96
○ 97
○ 98
○ 99
○ Don't know

At what age were you first told you had varicose veins?

- 0
- 1
- 2
- 3
- 4
- 5
- 6
- 7
- 8
- 9
- 10
- 11
- 12
- 13
- 14
- 15
- 16
- 17
- 18
- 19
- 20
- 21
- 22
- 23
- 24
- 25
- 26
- 27
- 28
- 29
- 30
- 31
- 32
- 33
- 34
- 35
- 36
- 37
- 38
- 39
- 40
- 41
- 42
- 43
- 44
- 45
- 46
- 47
- 48
- 49
- 50
- 51
- 52
- 53
- 54
- 55
- 56
- 57
- 58
- 59
- 60
- 61
- 62
- 63
- 64
- 65
- 66
- 67
- 68

◯ 69
◯ 70
◯ 71
◯ 72
◯ 73
◯ 74
◯ 75
◯ 76
◯ 77
◯ 78
◯ 79
◯ 80
◯ 81
◯ 82
◯ 83
◯ 84
◯ 85
◯ 86
◯ 87
◯ 88
◯ 89
◯ 90
◯ 91
◯ 92
◯ 93
◯ 94
◯ 95
◯ 96
◯ 97
◯ 98
◯ 99
◯ Don't know

At what age were you first told you had [heart_others]?

◯ 0
◯ 1
◯ 2
◯ 3
◯ 4
◯ 5
◯ 6
◯ 7
◯ 8
◯ 9
◯ 10
◯ 11
◯ 12
◯ 13
◯ 14
◯ 15
◯ 16
◯ 17
◯ 18
◯ 19
◯ 20
◯ 21
◯ 22
◯ 23
◯ 24
◯ 25
◯ 26
◯ 27
◯ 28
◯ 29
◯ 30
◯ 31
◯ 32
◯ 33
◯ 34
◯ 35
◯ 36
◯ 37
◯ 38
◯ 39
◯ 40
◯ 41
◯ 42
◯ 43
◯ 44
◯ 45
◯ 46
◯ 47
◯ 48
◯ 49
◯ 50
◯ 51
◯ 52
◯ 53
◯ 54
◯ 55
◯ 56
◯ 57
◯ 58
◯ 59
◯ 60
◯ 61
◯ 62
◯ 63
◯ 64
◯ 65
◯ 66
◯ 67
◯ 68

◯ 69
◯ 70
◯ 71
◯ 72
◯ 73
◯ 74
◯ 75
◯ 76
◯ 77
◯ 78
◯ 79
◯ 80
◯ 81
◯ 82
◯ 83
◯ 84
◯ 85
◯ 86
◯ 87
◯ 88
◯ 89
◯ 90
◯ 91
◯ 92
◯ 93
◯ 94
◯ 95
◯ 96
◯ 97
◯ 98
◯ 99
◯ Don't know

At what age were you first told you had [heart_others_2]?

◯ 0
◯ 1
◯ 2
◯ 3
◯ 4
◯ 5
◯ 6
◯ 7
◯ 8
◯ 9
◯ 10
◯ 11
◯ 12
◯ 13
◯ 14
◯ 15
◯ 16
◯ 17
◯ 18
◯ 19
◯ 20
◯ 21
◯ 22
◯ 23
◯ 24
◯ 25
◯ 26
◯ 27
◯ 28
◯ 29
◯ 30
◯ 31
◯ 32
◯ 33
◯ 34
◯ 35
◯ 36
◯ 37
◯ 38
◯ 39
◯ 40
◯ 41
◯ 42
◯ 43
◯ 44
◯ 45
◯ 46
◯ 47
◯ 48
◯ 49
◯ 50
◯ 51
◯ 52
◯ 53
◯ 54
◯ 55
◯ 56
◯ 57
◯ 58
◯ 59
◯ 60
◯ 61
◯ 62
◯ 63
◯ 64
◯ 65
◯ 66
◯ 67
◯ 68

- ◯ 69
- ◯ 70
- ◯ 71
- ◯ 72
- ◯ 73
- ◯ 74
- ◯ 75
- ◯ 76
- ◯ 77
- ◯ 78
- ◯ 79
- ◯ 80
- ◯ 81
- ◯ 82
- ◯ 83
- ◯ 84
- ◯ 85
- ◯ 86
- ◯ 87
- ◯ 88
- ◯ 89
- ◯ 90
- ◯ 91
- ◯ 92
- ◯ 93
- ◯ 94
- ◯ 95
- ◯ 96
- ◯ 97
- ◯ 98
- ◯ 99
- ◯ Don't know

At what age were you first told you had [heart_others_3]?

- 0
- 1
- 2
- 3
- 4
- 5
- 6
- 7
- 8
- 9
- 10
- 11
- 12
- 13
- 14
- 15
- 16
- 17
- 18
- 19
- 20
- 21
- 22
- 23
- 24
- 25
- 26
- 27
- 28
- 29
- 30
- 31
- 32
- 33
- 34
- 35
- 36
- 37
- 38
- 39
- 40
- 41
- 42
- 43
- 44
- 45
- 46
- 47
- 48
- 49
- 50
- 51
- 52
- 53
- 54
- 55
- 56
- 57
- 58
- 59
- 60
- 61
- 62
- 63
- 64
- 65
- 66
- 67
- 68

- ◯ 69
- ◯ 70
- ◯ 71
- ◯ 72
- ◯ 73
- ◯ 74
- ◯ 75
- ◯ 76
- ◯ 77
- ◯ 78
- ◯ 79
- ◯ 80
- ◯ 81
- ◯ 82
- ◯ 83
- ◯ 84
- ◯ 85
- ◯ 86
- ◯ 87
- ◯ 88
- ◯ 89
- ◯ 90
- ◯ 91
- ◯ 92
- ◯ 93
- ◯ 94
- ◯ 95
- ◯ 96
- ◯ 97
- ◯ 98
- ◯ 99
- ◯ Don't know

At what age were you first told you had this condition?

◯ 0
◯ 1
◯ 2
◯ 3
◯ 4
◯ 5
◯ 6
◯ 7
◯ 8
◯ 9
◯ 10
◯ 11
◯ 12
◯ 13
◯ 14
◯ 15
◯ 16
◯ 17
◯ 18
◯ 19
◯ 20
◯ 21
◯ 22
◯ 23
◯ 24
◯ 25
◯ 26
◯ 27
◯ 28
◯ 29
◯ 30
◯ 31
◯ 32
◯ 33
◯ 34
◯ 35
◯ 36
◯ 37
◯ 38
◯ 39
◯ 40
◯ 41
◯ 42
◯ 43
◯ 44
◯ 45
◯ 46
◯ 47
◯ 48
◯ 49
◯ 50
◯ 51
◯ 52
◯ 53
◯ 54
◯ 55
◯ 56
◯ 57
◯ 58
◯ 59
◯ 60
◯ 61
◯ 62
◯ 63
◯ 64
◯ 65
◯ 66
◯ 67
◯ 68

	◯ 69<br>◯ 70<br>◯ 71<br>◯ 72<br>◯ 73<br>◯ 74<br>◯ 75<br>◯ 76<br>◯ 77<br>◯ 78<br>◯ 79<br>◯ 80<br>◯ 81<br>◯ 82<br>◯ 83<br>◯ 84<br>◯ 85<br>◯ 86<br>◯ 87<br>◯ 88<br>◯ 89<br>◯ 90<br>◯ 91<br>◯ 92<br>◯ 93<br>◯ 94<br>◯ 95<br>◯ 96<br>◯ 97<br>◯ 98<br>◯ 99<br>◯ Don't know
Do you still have rheumatic heart disease?	◯ Yes<br>◯ No<br>◯ Don't know
Do you still have heat attack?	◯ Yes<br>◯ No<br>◯ Don't know
Do you still have heat failure?	◯ Yes<br>◯ No<br>◯ Don't know
Do you still have stroke (including after effects of stroke)?	◯ Yes<br>◯ No<br>◯ Don't know
Do you still have angina?	◯ Yes<br>◯ No<br>◯ Don't know
Do you still have high blood pressure/hypertension?	◯ Yes<br>◯ No<br>◯ Don't know
Do you still have low blood pressure/hypotension?	◯ Yes<br>◯ No<br>◯ Don't know

Do you still have hardening of the arteries/atherosclerosis/arteriosclerosis?	◯ Yes<br>◯ No<br>◯ Don't know
Do you still have fluid problems/fluid retention/oedema?	◯ Yes<br>◯ No<br>◯ Don't know
Do you still have high cholesterol?	◯ Yes<br>◯ No<br>◯ Don't know
Do you still have rapid or irregular heartbeats/tachycardia/palpitations?	◯ Yes<br>◯ No<br>◯ Don't know
Do you still have heart murmur/heart valve disorder	◯ Yes<br>◯ No<br>◯ Don't know
Do you still have haemorrhoids?	◯ Yes<br>◯ No<br>◯ Don't know
Do you still have varicose veins?	◯ Yes<br>◯ No<br>◯ Don't know
Do you still have [heart_others]?	◯ Yes<br>◯ No<br>◯ Don't know
Do you still have [heart_others_2]?	◯ Yes<br>◯ No<br>◯ Don't know
Do you still have [heart_others_3]?	◯ Yes<br>◯ No<br>◯ Don't know
Do you still have this condition?	◯ Yes<br>◯ No<br>◯ Don't know

How is rheumatic heart disease treated?
(Tick all that apply)

☐ no treatment ☐ diet ☐ exercise ☐ medication (please specify) ☐ natural/herbal therapy
☐ weight management ☐ supplements (please specify) ☐ other (please specify) ☐ Don't know

please specify the medication

____

please specify the supplements

____

please specify the treatment

____

How is heart attack treated?
(Tick all that apply)

☐ no treatment ☐ diet ☐ exercise ☐ medication (please specify) ☐ natural/herbal therapy
☐ weight management ☐ supplements (please specify) ☐ other (please specify) ☐ Don't know

please specify the medication

____

please specify the supplements

____

please specify the treatment

____

How is heart failure treated?
(Tick all that apply)

☐ no treatment ☐ diet ☐ exercise ☐ medication (please specify) ☐ natural/herbal therapy
☐ weight management ☐ supplements (please specify) ☐ other (please specify) ☐ Don't know

please specify the medication

____

please specify the supplements

____

please specify the treatment

____

How is stroke (including after effects of stroke) treated?
(Tick all that apply)

☐ no treatment ☐ diet ☐ exercise ☐ medication (please specify) ☐ natural/herbal therapy
☐ weight management ☐ supplements (please specify) ☐ other (please specify) ☐ Don't know

please specify the medication

____

please specify the supplements

____

please specify the treatment

____

How is angina treated?
(Tick all that apply)

☐ no treatment ☐ diet ☐ exercise ☐ medication (please specify) ☐ natural/herbal therapy
☐ weight management ☐ supplements (please specify) ☐ other (please specify) ☐ Don't know

please specify the medication ______________________________

please specify the supplements ______________________________

please specify the treatment ______________________________

How is high blood pressure/hypertension treated?
(Tick all that apply)

☐ no treatment ☐ diet ☐ exercise ☐ medication (please specify) ☐ natural/herbal therapy
☐ weight management ☐ supplements (please specify) ☐ other (please specify) ☐ Don't know

please specify the medication ______________________________

please specify the supplements ______________________________

please specify the treatment ______________________________

How is low blood pressure/hypotension treated?
(Tick all that apply)

☐ no treatment ☐ diet ☐ exercise ☐ medication (please specify) ☐ natural/herbal therapy
☐ weight management ☐ supplements (please specify) ☐ other (please specify) ☐ Don't know

please specify the medication ______________________________

please specify the supplements ______________________________

please specify the treatment ______________________________

How is hardening of the arteries/atherosclerosis/arteriosclerosis treated?
(Tick all that apply)

☐ no treatment ☐ diet ☐ exercise ☐ medication (please specify) ☐ natural/herbal therapy
☐ weight management ☐ supplements (please specify) ☐ other (please specify) ☐ Don't know

please specify the medication ______________________________

please specify the supplements ______________________________

please specify the treatment

____

How is fluid problems/fluid retention/oedema treated?
(Tick all that apply)

☐ no treatment ☐ diet ☐ exercise ☐ medication (please specify) ☐ natural/herbal therapy
☐ weight management ☐ supplements (please specify) ☐ other (please specify) ☐ Don't know

please specify the medication

____

please specify the supplements

____

please specify the treatment

____

How is high cholesterol treated?
(Tick all that apply)

☐ no treatment ☐ diet ☐ exercise ☐ medication (please specify) ☐ natural/herbal therapy
☐ weight management ☐ supplements (please specify) ☐ other (please specify) ☐ Don't know

please specify the medication

____

please specify the supplements

____

please specify the treatment

____

How is rapid or irregular heartbeats/tachycardia/palpitations treated?
(Tick all that apply)

☐ no treatment ☐ diet ☐ exercise ☐ medication (please specify) ☐ natural/herbal therapy
☐ weight management ☐ supplements (please specify) ☐ other (please specify) ☐ Don't know

please specify the medication

____

please specify the supplements

____

please specify the treatment

____

How is heart murmur/heart valve disorder treated/
(Tick all that apply)

☐ no treatment ☐ diet ☐ exercise ☐ medication (please specify) ☐ natural/herbal therapy
☐ weight management ☐ supplements (please specify) ☐ other (please specify) ☐ Don't know

please specify the medication

____

please specify the supplements

____

please specify the treatment

____

How is haemorrohoids treataed?
(Tick all that apply)

☐ no treatment ☐ diet ☐ exercise ☐ medication (please specify) ☐ natural/herbal therapy
☐ weight management ☐ supplements (please specify) ☐ other (please specify) ☐ Don't know

please specify the medication

____

please specify the supplements

____

please specify the treatment

____

How is varicose veins treated?
(Tick all that apply)

☐ no treatment ☐ diet ☐ exercise ☐ medication (please specify) ☐ natural/herbal therapy
☐ weight management ☐ supplements (please specify) ☐ other (please specify) ☐ Don't know

please specify the medication

____

please specify the supplements

____

please specify the treatment

____

How is [heart_others] treated?
(Tick all that apply)

☐ no treatment ☐ diet ☐ exercise ☐ medication (please specify) ☐ natural/herbal therapy
☐ weight management ☐ supplements (please specify) ☐ other (please specify) ☐ Don't know

please specify the medication

____

please specify the supplements

____

please specify the treatment ______________________

How is [heart_others_2] treated?
(Tick all that apply)

☐ no treatment ☐ diet ☐ exercise ☐ medication (please specify) ☐ natural/herbal therapy
☐ weight management ☐ supplements (please specify) ☐ other (please specify) ☐ Don't know

please specify the medication ______________________

please specify the supplements ______________________

please specify the treatment ______________________

How is [heart_others_3] treated?
(Tick all that apply)

☐ no treatment ☐ diet ☐ exercise ☐ medication (please specify) ☐ natural/herbal therapy
☐ weight management ☐ supplements (please specify) ☐ other (please specify) ☐ Don't know

please specify the medication ______________________

please specify the supplements ______________________

please specify the treatment ______________________

How is this condition treated?
(Tick all that apply)

☐ no treatment ☐ diet ☐ exercise ☐ medication (please specify) ☐ natural/herbal therapy
☐ weight management ☐ supplements (please specify) ☐ other (please specify) ☐ Don't know

please specify the medication ______________________

please specify the supplements ______________________

please specify the treatment ______________________

Have either of your parents, or any of brothers or sisters ever been told by a doctor (GP or specialist) or nurse that they have any heart or circulatory conditions?

◯ Yes
◯ No
◯ Don't know

Including any conditions which can be controlled with medication, have you ever been told by a doctor (GP or specialist) or nurse that you have diabetes or pre-diabetes?

◯ Yes
◯ No
◯ Don't know

What are the names of this/these condition/s?
(Tick any that apply)

- ☐ Diabetes insipidus
- ☐ Gestational (pregnancy)
- ☐ Pre-diabetes (impaired fasting glucose, impaired glucose tolerance)
- ☐ Type 1 (Insulin Dependent Diabetes Mellitus/Juvenile onset Diabetes /Type A)
- ☐ Type 2 (Non-insulin Dependent Diabetes Mellitus/Adult onset Diabetes/Type B)
- ☐ Type unknown
- ☐ Other (please specify)

---

please specify the condition ______________________

Do you have another diebetes condition need to be specified?

○ Yes ○ No

---

please specify the second condition ______________________

Do you have another diebetes condition need to be specified?

○ Yes ○ No

---

please specify the third condition ______________________

At what age were you first told you had Type 1 diabetes (Insulin Dependent Diabetes Mellitus/Juvenile onset Diabetes /Type A)?

○ 0 ○ 1 ○ 2 ○ 3
○ 4 ○ 5 ○ 6 ○ 7
○ 8 ○ 9 ○ 10 ○ 11
○ 12 ○ 13 ○ 14 ○ 15
○ 16 ○ 17 ○ 18 ○ 19
○ 20 ○ 21 ○ 22 ○ 23
○ 24 ○ 25 ○ 26 ○ 27
○ 28 ○ 29 ○ 30 ○ 31
○ 32 ○ 33 ○ 34 ○ 35
○ 36 ○ 37 ○ 38 ○ 39
○ 40 ○ 41 ○ 42 ○ 43
○ 44 ○ 45 ○ 46 ○ 47
○ 48 ○ 49 ○ 50 ○ 51
○ 52 ○ 53 ○ 54 ○ 55
○ 56 ○ 57 ○ 58 ○ 59
○ 60 ○ 61 ○ 62 ○ 63
○ 64 ○ 65 ○ 66 ○ 67
○ 68 ○ 69 ○ 70 ○ 71
○ 72 ○ 73 ○ 74 ○ 75
○ 76 ○ 77 ○ 78 ○ 79
○ 80 ○ 81 ○ 82 ○ 83
○ 84 ○ 85 ○ 86 ○ 87
○ 88 ○ 89 ○ 90 ○ 91
○ 92 ○ 93 ○ 94 ○ 95
○ 96 ○ 97 ○ 98 ○ 99
○ Don't know

At what age were you first told you had Type 2 diabetes (Non-insulin Dependent Diabetes Mellitus/Adult Onset Diabetes/Type B)?

◯ 0
◯ 1
◯ 2
◯ 3
◯ 4
◯ 5
◯ 6
◯ 7
◯ 8
◯ 9
◯ 10
◯ 11
◯ 12
◯ 13
◯ 14
◯ 15
◯ 16
◯ 17
◯ 18
◯ 19
◯ 20
◯ 21
◯ 22
◯ 23
◯ 24
◯ 25
◯ 26
◯ 27
◯ 28
◯ 29
◯ 30
◯ 31
◯ 32
◯ 33
◯ 34
◯ 35
◯ 36
◯ 37
◯ 38
◯ 39
◯ 40
◯ 41
◯ 42
◯ 43
◯ 44
◯ 45
◯ 46
◯ 47
◯ 48
◯ 49
◯ 50
◯ 51
◯ 52
◯ 53
◯ 54
◯ 55
◯ 56
◯ 57
◯ 58
◯ 59
◯ 60
◯ 61
◯ 62
◯ 63
◯ 64
◯ 65
◯ 66
◯ 67
◯ 68

- ◯ 69
- ◯ 70
- ◯ 71
- ◯ 72
- ◯ 73
- ◯ 74
- ◯ 75
- ◯ 76
- ◯ 77
- ◯ 78
- ◯ 79
- ◯ 80
- ◯ 81
- ◯ 82
- ◯ 83
- ◯ 84
- ◯ 85
- ◯ 86
- ◯ 87
- ◯ 88
- ◯ 89
- ◯ 90
- ◯ 91
- ◯ 92
- ◯ 93
- ◯ 94
- ◯ 95
- ◯ 96
- ◯ 97
- ◯ 98
- ◯ 99
- ◯ Don't know

At what age were you first told you had gestational (pregnancy) diabetes?

◯ 0
◯ 1
◯ 2
◯ 3
◯ 4
◯ 5
◯ 6
◯ 7
◯ 8
◯ 9
◯ 10
◯ 11
◯ 12
◯ 13
◯ 14
◯ 15
◯ 16
◯ 17
◯ 18
◯ 19
◯ 20
◯ 21
◯ 22
◯ 23
◯ 24
◯ 25
◯ 26
◯ 27
◯ 28
◯ 29
◯ 30
◯ 31
◯ 32
◯ 33
◯ 34
◯ 35
◯ 36
◯ 37
◯ 38
◯ 39
◯ 40
◯ 41
◯ 42
◯ 43
◯ 44
◯ 45
◯ 46
◯ 47
◯ 48
◯ 49
◯ 50
◯ 51
◯ 52
◯ 53
◯ 54
◯ 55
◯ 56
◯ 57
◯ 58
◯ 59
◯ 60
◯ 61
◯ 62
◯ 63
◯ 64
◯ 65
◯ 66
◯ 67
◯ 68

- ◯ 69
- ◯ 70
- ◯ 71
- ◯ 72
- ◯ 73
- ◯ 74
- ◯ 75
- ◯ 76
- ◯ 77
- ◯ 78
- ◯ 79
- ◯ 80
- ◯ 81
- ◯ 82
- ◯ 83
- ◯ 84
- ◯ 85
- ◯ 86
- ◯ 87
- ◯ 88
- ◯ 89
- ◯ 90
- ◯ 91
- ◯ 92
- ◯ 93
- ◯ 94
- ◯ 95
- ◯ 96
- ◯ 97
- ◯ 98
- ◯ 99
- ◯ Don't know

At what age were you first told you had diabetes insipidus?

- ◯ 0
- ◯ 1
- ◯ 2
- ◯ 3
- ◯ 4
- ◯ 5
- ◯ 6
- ◯ 7
- ◯ 8
- ◯ 9
- ◯ 10
- ◯ 11
- ◯ 12
- ◯ 13
- ◯ 14
- ◯ 15
- ◯ 16
- ◯ 17
- ◯ 18
- ◯ 19
- ◯ 20
- ◯ 21
- ◯ 22
- ◯ 23
- ◯ 24
- ◯ 25
- ◯ 26
- ◯ 27
- ◯ 28
- ◯ 29
- ◯ 30
- ◯ 31
- ◯ 32
- ◯ 33
- ◯ 34
- ◯ 35
- ◯ 36
- ◯ 37
- ◯ 38
- ◯ 39
- ◯ 40
- ◯ 41
- ◯ 42
- ◯ 43
- ◯ 44
- ◯ 45
- ◯ 46
- ◯ 47
- ◯ 48
- ◯ 49
- ◯ 50
- ◯ 51
- ◯ 52
- ◯ 53
- ◯ 54
- ◯ 55
- ◯ 56
- ◯ 57
- ◯ 58
- ◯ 59
- ◯ 60
- ◯ 61
- ◯ 62
- ◯ 63
- ◯ 64
- ◯ 65
- ◯ 66
- ◯ 67
- ◯ 68

- ◯ 69
- ◯ 70
- ◯ 71
- ◯ 72
- ◯ 73
- ◯ 74
- ◯ 75
- ◯ 76
- ◯ 77
- ◯ 78
- ◯ 79
- ◯ 80
- ◯ 81
- ◯ 82
- ◯ 83
- ◯ 84
- ◯ 85
- ◯ 86
- ◯ 87
- ◯ 88
- ◯ 89
- ◯ 90
- ◯ 91
- ◯ 92
- ◯ 93
- ◯ 94
- ◯ 95
- ◯ 96
- ◯ 97
- ◯ 98
- ◯ 99
- ◯ Don't know

At what age were you first told you had pre-diabetes (impaired fasting glucose, impaired glucose intolerance)?

◯ 0
◯ 1
◯ 2
◯ 3
◯ 4
◯ 5
◯ 6
◯ 7
◯ 8
◯ 9
◯ 10
◯ 11
◯ 12
◯ 13
◯ 14
◯ 15
◯ 16
◯ 17
◯ 18
◯ 19
◯ 20
◯ 21
◯ 22
◯ 23
◯ 24
◯ 25
◯ 26
◯ 27
◯ 28
◯ 29
◯ 30
◯ 31
◯ 32
◯ 33
◯ 34
◯ 35
◯ 36
◯ 37
◯ 38
◯ 39
◯ 40
◯ 41
◯ 42
◯ 43
◯ 44
◯ 45
◯ 46
◯ 47
◯ 48
◯ 49
◯ 50
◯ 51
◯ 52
◯ 53
◯ 54
◯ 55
◯ 56
◯ 57
◯ 58
◯ 59
◯ 60
◯ 61
◯ 62
◯ 63
◯ 64
◯ 65
◯ 66
◯ 67
◯ 68

- ◯ 69
- ◯ 70
- ◯ 71
- ◯ 72
- ◯ 73
- ◯ 74
- ◯ 75
- ◯ 76
- ◯ 77
- ◯ 78
- ◯ 79
- ◯ 80
- ◯ 81
- ◯ 82
- ◯ 83
- ◯ 84
- ◯ 85
- ◯ 86
- ◯ 87
- ◯ 88
- ◯ 89
- ◯ 90
- ◯ 91
- ◯ 92
- ◯ 93
- ◯ 94
- ◯ 95
- ◯ 96
- ◯ 97
- ◯ 98
- ◯ 99
- ◯ Don't know

At what age were you first told you had this condition?

◯ 0
◯ 1
◯ 2
◯ 3
◯ 4
◯ 5
◯ 6
◯ 7
◯ 8
◯ 9
◯ 10
◯ 11
◯ 12
◯ 13
◯ 14
◯ 15
◯ 16
◯ 17
◯ 18
◯ 19
◯ 20
◯ 21
◯ 22
◯ 23
◯ 24
◯ 25
◯ 26
◯ 27
◯ 28
◯ 29
◯ 30
◯ 31
◯ 32
◯ 33
◯ 34
◯ 35
◯ 36
◯ 37
◯ 38
◯ 39
◯ 40
◯ 41
◯ 42
◯ 43
◯ 44
◯ 45
◯ 46
◯ 47
◯ 48
◯ 49
◯ 50
◯ 51
◯ 52
◯ 53
◯ 54
◯ 55
◯ 56
◯ 57
◯ 58
◯ 59
◯ 60
◯ 61
◯ 62
◯ 63
◯ 64
◯ 65
◯ 66
◯ 67
◯ 68

- ◯ 69
- ◯ 70
- ◯ 71
- ◯ 72
- ◯ 73
- ◯ 74
- ◯ 75
- ◯ 76
- ◯ 77
- ◯ 78
- ◯ 79
- ◯ 80
- ◯ 81
- ◯ 82
- ◯ 83
- ◯ 84
- ◯ 85
- ◯ 86
- ◯ 87
- ◯ 88
- ◯ 89
- ◯ 90
- ◯ 91
- ◯ 92
- ◯ 93
- ◯ 94
- ◯ 95
- ◯ 96
- ◯ 97
- ◯ 98
- ◯ 99
- ◯ Don't know

At what age were you first told you had [diabetes_others]?

◯ 0
◯ 1
◯ 2
◯ 3
◯ 4
◯ 5
◯ 6
◯ 7
◯ 8
◯ 9
◯ 10
◯ 11
◯ 12
◯ 13
◯ 14
◯ 15
◯ 16
◯ 17
◯ 18
◯ 19
◯ 20
◯ 21
◯ 22
◯ 23
◯ 24
◯ 25
◯ 26
◯ 27
◯ 28
◯ 29
◯ 30
◯ 31
◯ 32
◯ 33
◯ 34
◯ 35
◯ 36
◯ 37
◯ 38
◯ 39
◯ 40
◯ 41
◯ 42
◯ 43
◯ 44
◯ 45
◯ 46
◯ 47
◯ 48
◯ 49
◯ 50
◯ 51
◯ 52
◯ 53
◯ 54
◯ 55
◯ 56
◯ 57
◯ 58
◯ 59
◯ 60
◯ 61
◯ 62
◯ 63
◯ 64
◯ 65
◯ 66
◯ 67
◯ 68

- ◯ 69
- ◯ 70
- ◯ 71
- ◯ 72
- ◯ 73
- ◯ 74
- ◯ 75
- ◯ 76
- ◯ 77
- ◯ 78
- ◯ 79
- ◯ 80
- ◯ 81
- ◯ 82
- ◯ 83
- ◯ 84
- ◯ 85
- ◯ 86
- ◯ 87
- ◯ 88
- ◯ 89
- ◯ 90
- ◯ 91
- ◯ 92
- ◯ 93
- ◯ 94
- ◯ 95
- ◯ 96
- ◯ 97
- ◯ 98
- ◯ 99
- ◯ Don't know

At what age were you first told you had [diabetes_others_2]?

- ◯ 0
- ◯ 1
- ◯ 2
- ◯ 3
- ◯ 4
- ◯ 5
- ◯ 6
- ◯ 7
- ◯ 8
- ◯ 9
- ◯ 10
- ◯ 11
- ◯ 12
- ◯ 13
- ◯ 14
- ◯ 15
- ◯ 16
- ◯ 17
- ◯ 18
- ◯ 19
- ◯ 20
- ◯ 21
- ◯ 22
- ◯ 23
- ◯ 24
- ◯ 25
- ◯ 26
- ◯ 27
- ◯ 28
- ◯ 29
- ◯ 30
- ◯ 31
- ◯ 32
- ◯ 33
- ◯ 34
- ◯ 35
- ◯ 36
- ◯ 37
- ◯ 38
- ◯ 39
- ◯ 40
- ◯ 41
- ◯ 42
- ◯ 43
- ◯ 44
- ◯ 45
- ◯ 46
- ◯ 47
- ◯ 48
- ◯ 49
- ◯ 50
- ◯ 51
- ◯ 52
- ◯ 53
- ◯ 54
- ◯ 55
- ◯ 56
- ◯ 57
- ◯ 58
- ◯ 59
- ◯ 60
- ◯ 61
- ◯ 62
- ◯ 63
- ◯ 64
- ◯ 65
- ◯ 66
- ◯ 67
- ◯ 68

◯ 69
◯ 70
◯ 71
◯ 72
◯ 73
◯ 74
◯ 75
◯ 76
◯ 77
◯ 78
◯ 79
◯ 80
◯ 81
◯ 82
◯ 83
◯ 84
◯ 85
◯ 86
◯ 87
◯ 88
◯ 89
◯ 90
◯ 91
◯ 92
◯ 93
◯ 94
◯ 95
◯ 96
◯ 97
◯ 98
◯ 99
◯ Don't know

At what age were you first told you had [diabetes_others_3]?

- ◯ 0
- ◯ 1
- ◯ 2
- ◯ 3
- ◯ 4
- ◯ 5
- ◯ 6
- ◯ 7
- ◯ 8
- ◯ 9
- ◯ 10
- ◯ 11
- ◯ 12
- ◯ 13
- ◯ 14
- ◯ 15
- ◯ 16
- ◯ 17
- ◯ 18
- ◯ 19
- ◯ 20
- ◯ 21
- ◯ 22
- ◯ 23
- ◯ 24
- ◯ 25
- ◯ 26
- ◯ 27
- ◯ 28
- ◯ 29
- ◯ 30
- ◯ 31
- ◯ 32
- ◯ 33
- ◯ 34
- ◯ 35
- ◯ 36
- ◯ 37
- ◯ 38
- ◯ 39
- ◯ 40
- ◯ 41
- ◯ 42
- ◯ 43
- ◯ 44
- ◯ 45
- ◯ 46
- ◯ 47
- ◯ 48
- ◯ 49
- ◯ 50
- ◯ 51
- ◯ 52
- ◯ 53
- ◯ 54
- ◯ 55
- ◯ 56
- ◯ 57
- ◯ 58
- ◯ 59
- ◯ 60
- ◯ 61
- ◯ 62
- ◯ 63
- ◯ 64
- ◯ 65
- ◯ 66
- ◯ 67
- ◯ 68

	◯ 69<br>◯ 70<br>◯ 71<br>◯ 72<br>◯ 73<br>◯ 74<br>◯ 75<br>◯ 76<br>◯ 77<br>◯ 78<br>◯ 79<br>◯ 80<br>◯ 81<br>◯ 82<br>◯ 83<br>◯ 84<br>◯ 85<br>◯ 86<br>◯ 87<br>◯ 88<br>◯ 89<br>◯ 90<br>◯ 91<br>◯ 92<br>◯ 93<br>◯ 94<br>◯ 95<br>◯ 96<br>◯ 97<br>◯ 98<br>◯ 99<br>◯ Don't know
Do you still have Type 1/Type A diabetes?	◯ Yes<br>◯ No<br>◯ Don't know
Do you still have Type 2/Type B diabetes?	◯ Yes<br>◯ No<br>◯ Don't know
Do you still have gestational diabetes?	◯ Yes<br>◯ No<br>◯ Don't know
Do you still have diabetes insipidus?	◯ Yes<br>◯ No<br>◯ Don't know
Do you still have pre-diabetes?	◯ Yes<br>◯ No<br>◯ Don't know
Do you still have this condition?	◯ Yes<br>◯ No<br>◯ Don't know
Do you still have [diabetets_others]?	◯ Yes<br>◯ No<br>◯ Don't know

Do you still have [diabetets_others_2]?

◯ Yes
◯ No
◯ Don't know

Do you still have [diabetets_others_3]?

◯ Yes
◯ No
◯ Don't know

How is Type 1 diabetes (Insulin Dependent Diabetes Mellitus/Juvenile Onset Diabetes /Type A) treated?
(Tick all that apply)

☐ no treatment ☐ diet ☐ exercise ☐ medication (please specify) ☐ natural/herbal therapy
☐ weight management ☐ supplements (please specify) ☐ other (please specify) ☐ Don't know

please specify the medication

____

please specify the supplements

____

please specify the treatment

____

How is Type 2 diabetes (Non-insulin Dependent Diabetes Mellitus/Adult Onset Diabetes/Type B treated?
(Tick all that apply)

☐ no treatment ☐ diet ☐ exercise ☐ medication (please specify) ☐ natural/herbal therapy
☐ weight management ☐ supplements (please specify) ☐ other (please specify) ☐ Don't know

please specify the medication

____

please specify the supplements

____

please specify the treatment

____

How is gestational (pregnancy) diabetes treated?
(Tick all that apply)

☐ no treatment ☐ diet ☐ exercise ☐ medication (please specify) ☐ natural/herbal therapy
☐ weight management ☐ supplements (please specify) ☐ other (please specify) ☐ Don't know

please specify the medication

____

please specify the supplements

____

please specify the treatment

____

How is diabetes insipidus treated?
(Tick all that apply)

☐ no treatment ☐ diet ☐ exercise ☐ medication (please specify) ☐ natural/herbal therapy
☐ weight management ☐ supplements (please specify) ☐ other (please specify) ☐ Don't know

please specify the medication

____

please specify the supplements

____

please specify the treatment

____

How is pre-diabetes (impaired fasting glucose, impaired glucose tolerance) treated?
(Tick all that apply)

☐ no treatment ☐ diet ☐ exercise ☐ medication (please specify) ☐ natural/herbal therapy
☐ weight management ☐ supplements (please specify) ☐ other (please specify) ☐ Don't know

please specify the medication

____

please specify the supplements

____

please specify the treatment

____

How is this condition treated?
(Tick all that apply)

☐ no treatment ☐ diet ☐ exercise ☐ medication (please specify) ☐ natural/herbal therapy
☐ weight management ☐ supplements (please specify) ☐ other (please specify) ☐ Don't know

please specify the medication

____

please specify the supplements

____

please specify the treatment

____

How is [diabetes_others] treated?
(Tick all that apply)

☐ no treatment ☐ diet ☐ exercise ☐ medication (please specify) ☐ natural/herbal therapy
☐ weight management ☐ supplements (please specify) ☐ other (please specify) ☐ Don't know

please specify the medication

____

please specify the supplements

____

please specify the treatment

____

How is [diabetes_others_2] treated?
(Tick all that apply)

☐ no treatment ☐ diet ☐ exercise ☐ medication (please specify) ☐ natural/herbal therapy
☐ weight management ☐ supplements (please specify) ☐ other (please specify) ☐ Don't know

please specify the medication

____

please specify the supplements

____

please specify the treatment

____

How is [diabetes_others_3] treated?
(Tick all that apply)

☐ no treatment ☐ diet ☐ exercise ☐ medication (please specify) ☐ natural/herbal therapy
☐ weight management ☐ supplements (please specify) ☐ other (please specify) ☐ Don't know

please specify the medication

____

please specify the supplements

____

please specify the treatment

____

Have either of your parents, or any of brothers or sisters ever been told by a doctor (GP or specialist) or nurse that they have Diabetes?

◯ Yes
◯ No
◯ Don't know

Including any conditions which can be controlled with medication, have you ever been told by a doctor (GP or specialist) or nurse that you have osteoporosis or osteopenia?
(Osteopenia is a mild loss of bone density that may progress to osteoporosis.)

◯ Yes
◯ No
◯ Don't know

Which one were you told you have?

☐ Osteoporosis
☐ Osteopenia
☐ Don't Know
(Tick all that apply)

At what age were you first told you had osteoporosis?

- ◯ 0
- ◯ 1
- ◯ 2
- ◯ 3
- ◯ 4
- ◯ 5
- ◯ 6
- ◯ 7
- ◯ 8
- ◯ 9
- ◯ 10
- ◯ 11
- ◯ 12
- ◯ 13
- ◯ 14
- ◯ 15
- ◯ 16
- ◯ 17
- ◯ 18
- ◯ 19
- ◯ 20
- ◯ 21
- ◯ 22
- ◯ 23
- ◯ 24
- ◯ 25
- ◯ 26
- ◯ 27
- ◯ 28
- ◯ 29
- ◯ 30
- ◯ 31
- ◯ 32
- ◯ 33
- ◯ 34
- ◯ 35
- ◯ 36
- ◯ 37
- ◯ 38
- ◯ 39
- ◯ 40
- ◯ 41
- ◯ 42
- ◯ 43
- ◯ 44
- ◯ 45
- ◯ 46
- ◯ 47
- ◯ 48
- ◯ 49
- ◯ 50
- ◯ 51
- ◯ 52
- ◯ 53
- ◯ 54
- ◯ 55
- ◯ 56
- ◯ 57
- ◯ 58
- ◯ 59
- ◯ 60
- ◯ 61
- ◯ 62
- ◯ 63
- ◯ 64
- ◯ 65
- ◯ 66
- ◯ 67
- ◯ 68

- ◯ 69
- ◯ 70
- ◯ 71
- ◯ 72
- ◯ 73
- ◯ 74
- ◯ 75
- ◯ 76
- ◯ 77
- ◯ 78
- ◯ 79
- ◯ 80
- ◯ 81
- ◯ 82
- ◯ 83
- ◯ 84
- ◯ 85
- ◯ 86
- ◯ 87
- ◯ 88
- ◯ 89
- ◯ 90
- ◯ 91
- ◯ 92
- ◯ 93
- ◯ 94
- ◯ 95
- ◯ 96
- ◯ 97
- ◯ 98
- ◯ 99
- ◯ Don't know

At what age were you first told you had osteopenia?

- ◯ 0
- ◯ 1
- ◯ 2
- ◯ 3
- ◯ 4
- ◯ 5
- ◯ 6
- ◯ 7
- ◯ 8
- ◯ 9
- ◯ 10
- ◯ 11
- ◯ 12
- ◯ 13
- ◯ 14
- ◯ 15
- ◯ 16
- ◯ 17
- ◯ 18
- ◯ 19
- ◯ 20
- ◯ 21
- ◯ 22
- ◯ 23
- ◯ 24
- ◯ 25
- ◯ 26
- ◯ 27
- ◯ 28
- ◯ 29
- ◯ 30
- ◯ 31
- ◯ 32
- ◯ 33
- ◯ 34
- ◯ 35
- ◯ 36
- ◯ 37
- ◯ 38
- ◯ 39
- ◯ 40
- ◯ 41
- ◯ 42
- ◯ 43
- ◯ 44
- ◯ 45
- ◯ 46
- ◯ 47
- ◯ 48
- ◯ 49
- ◯ 50
- ◯ 51
- ◯ 52
- ◯ 53
- ◯ 54
- ◯ 55
- ◯ 56
- ◯ 57
- ◯ 58
- ◯ 59
- ◯ 60
- ◯ 61
- ◯ 62
- ◯ 63
- ◯ 64
- ◯ 65
- ◯ 66
- ◯ 67
- ◯ 68

◯ 69
◯ 70
◯ 71
◯ 72
◯ 73
◯ 74
◯ 75
◯ 76
◯ 77
◯ 78
◯ 79
◯ 80
◯ 81
◯ 82
◯ 83
◯ 84
◯ 85
◯ 86
◯ 87
◯ 88
◯ 89
◯ 90
◯ 91
◯ 92
◯ 93
◯ 94
◯ 95
◯ 96
◯ 97
◯ 98
◯ 99
◯ Don't know

At what age were you first told you had this condition?

〇 0
〇 1
〇 2
〇 3
〇 4
〇 5
〇 6
〇 7
〇 8
〇 9
〇 10
〇 11
〇 12
〇 13
〇 14
〇 15
〇 16
〇 17
〇 18
〇 19
〇 20
〇 21
〇 22
〇 23
〇 24
〇 25
〇 26
〇 27
〇 28
〇 29
〇 30
〇 31
〇 32
〇 33
〇 34
〇 35
〇 36
〇 37
〇 38
〇 39
〇 40
〇 41
〇 42
〇 43
〇 44
〇 45
〇 46
〇 47
〇 48
〇 49
〇 50
〇 51
〇 52
〇 53
〇 54
〇 55
〇 56
〇 57
〇 58
〇 59
〇 60
〇 61
〇 62
〇 63
〇 64
〇 65
〇 66
〇 67
〇 68

◯ 69
◯ 70
◯ 71
◯ 72
◯ 73
◯ 74
◯ 75
◯ 76
◯ 77
◯ 78
◯ 79
◯ 80
◯ 81
◯ 82
◯ 83
◯ 84
◯ 85
◯ 86
◯ 87
◯ 88
◯ 89
◯ 90
◯ 91
◯ 92
◯ 93
◯ 94
◯ 95
◯ 96
◯ 97
◯ 98
◯ 99
◯ Don't know

Do you still have osteoporosis?

◯ Yes
◯ No
◯ Don't know

Do you still have osteopenia?

◯ Yes
◯ No
◯ Don't know

How is osteoporosis treated?
(Tick all that apply)

☐ no treatment ☐ diet ☐ exercise ☐ medication (please specify) ☐ natural/herbal therapy
☐ weight management ☐ supplements (please specify) ☐ other (please specify) ☐ Don't know

---

please specify the medication

____

---

please specify the supplements

____

---

please specify the treatment

____

How is osteopenia treated?
(Tick all that apply)

☐ no treatment ☐ diet ☐ exercise ☐ medication (please specify) ☐ natural/herbal therapy
☐ weight management ☐ supplements (please specify) ☐ other (please specify) ☐ Don't know

please specify the medication ______________________

please specify the supplements ______________________

please specify the treatment ______________________

How is this condition treated?
(Tick all that apply)

☐ no treatment ☐ diet ☐ exercise ☐ medication (please specify) ☐ natural/herbal therapy
☐ weight management ☐ supplements (please specify) ☐ other (please specify) ☐ Don't know

please specify the medication ______________________

please specify the supplements ______________________

please specify the treatment ______________________

Have either of your parents, or any of brothers or sisters ever been told by a doctor (GP or specialist) that they have osteoporosis or osteopaenia?

◯ Yes
◯ No
◯ Don't know

Which best describes the *regularity of your usual menstrual periods between ages 20 and 35 when you were neither pregnant nor using oral contraceptives?

◯ Very regular
◯ Usually regular
◯ Usually irregular
◯ Very irregular

*Regular periods are between 26 and 34 days. Irregular periods are 35 days or more between periods

Between the ages of 20 and 35, did you ever experience amenorrhoea (no periods for more than 3 months) when you were neither pregnant nor using oral contraceptives?

◯ Yes
◯ No
◯ Don't know

Have you ever been told by a doctor (GP or specialist) or nurse that you have gout, osteoarthritis or rheumatoid arthritis?

◯ Yes
◯ No
◯ Don't know

Which one were you told you have?
(Tick any that apply)

☐ Gout ☐ Osteoarthritis ☐ Rheumatoid arthritis ☐ Don't know

At what age were you first told you had gout?

◯ 0
◯ 1
◯ 2
◯ 3
◯ 4
◯ 5
◯ 6
◯ 7
◯ 8
◯ 9
◯ 10
◯ 11
◯ 12
◯ 13
◯ 14
◯ 15
◯ 16
◯ 17
◯ 18
◯ 19
◯ 20
◯ 21
◯ 22
◯ 23
◯ 24
◯ 25
◯ 26
◯ 27
◯ 28
◯ 29
◯ 30
◯ 31
◯ 32
◯ 33
◯ 34
◯ 35
◯ 36
◯ 37
◯ 38
◯ 39
◯ 40
◯ 41
◯ 42
◯ 43
◯ 44
◯ 45
◯ 46
◯ 47
◯ 48
◯ 49
◯ 50
◯ 51
◯ 52
◯ 53
◯ 54
◯ 55
◯ 56
◯ 57
◯ 58
◯ 59
◯ 60
◯ 61
◯ 62
◯ 63
◯ 64
◯ 65
◯ 66
◯ 67
◯ 68

- ◯ 69
- ◯ 70
- ◯ 71
- ◯ 72
- ◯ 73
- ◯ 74
- ◯ 75
- ◯ 76
- ◯ 77
- ◯ 78
- ◯ 79
- ◯ 80
- ◯ 81
- ◯ 82
- ◯ 83
- ◯ 84
- ◯ 85
- ◯ 86
- ◯ 87
- ◯ 88
- ◯ 89
- ◯ 90
- ◯ 91
- ◯ 92
- ◯ 93
- ◯ 94
- ◯ 95
- ◯ 96
- ◯ 97
- ◯ 98
- ◯ 99
- ◯ Don't know

At what age were you first told you had osteoarthritis?

◯ 0
◯ 1
◯ 2
◯ 3
◯ 4
◯ 5
◯ 6
◯ 7
◯ 8
◯ 9
◯ 10
◯ 11
◯ 12
◯ 13
◯ 14
◯ 15
◯ 16
◯ 17
◯ 18
◯ 19
◯ 20
◯ 21
◯ 22
◯ 23
◯ 24
◯ 25
◯ 26
◯ 27
◯ 28
◯ 29
◯ 30
◯ 31
◯ 32
◯ 33
◯ 34
◯ 35
◯ 36
◯ 37
◯ 38
◯ 39
◯ 40
◯ 41
◯ 42
◯ 43
◯ 44
◯ 45
◯ 46
◯ 47
◯ 48
◯ 49
◯ 50
◯ 51
◯ 52
◯ 53
◯ 54
◯ 55
◯ 56
◯ 57
◯ 58
◯ 59
◯ 60
◯ 61
◯ 62
◯ 63
◯ 64
◯ 65
◯ 66
◯ 67
◯ 68

- ◯ 69
- ◯ 70
- ◯ 71
- ◯ 72
- ◯ 73
- ◯ 74
- ◯ 75
- ◯ 76
- ◯ 77
- ◯ 78
- ◯ 79
- ◯ 80
- ◯ 81
- ◯ 82
- ◯ 83
- ◯ 84
- ◯ 85
- ◯ 86
- ◯ 87
- ◯ 88
- ◯ 89
- ◯ 90
- ◯ 91
- ◯ 92
- ◯ 93
- ◯ 94
- ◯ 95
- ◯ 96
- ◯ 97
- ◯ 98
- ◯ 99
- ◯ Don't know

At what age were you first told you had rheumatoid arthritis?

◯ 0
◯ 1
◯ 2
◯ 3
◯ 4
◯ 5
◯ 6
◯ 7
◯ 8
◯ 9
◯ 10
◯ 11
◯ 12
◯ 13
◯ 14
◯ 15
◯ 16
◯ 17
◯ 18
◯ 19
◯ 20
◯ 21
◯ 22
◯ 23
◯ 24
◯ 25
◯ 26
◯ 27
◯ 28
◯ 29
◯ 30
◯ 31
◯ 32
◯ 33
◯ 34
◯ 35
◯ 36
◯ 37
◯ 38
◯ 39
◯ 40
◯ 41
◯ 42
◯ 43
◯ 44
◯ 45
◯ 46
◯ 47
◯ 48
◯ 49
◯ 50
◯ 51
◯ 52
◯ 53
◯ 54
◯ 55
◯ 56
◯ 57
◯ 58
◯ 59
◯ 60
◯ 61
◯ 62
◯ 63
◯ 64
◯ 65
◯ 66
◯ 67
◯ 68

- ◯ 69
- ◯ 70
- ◯ 71
- ◯ 72
- ◯ 73
- ◯ 74
- ◯ 75
- ◯ 76
- ◯ 77
- ◯ 78
- ◯ 79
- ◯ 80
- ◯ 81
- ◯ 82
- ◯ 83
- ◯ 84
- ◯ 85
- ◯ 86
- ◯ 87
- ◯ 88
- ◯ 89
- ◯ 90
- ◯ 91
- ◯ 92
- ◯ 93
- ◯ 94
- ◯ 95
- ◯ 96
- ◯ 97
- ◯ 98
- ◯ 99
- ◯ Don't know

At what age were you first told you had this condition?

◯ 0
◯ 1
◯ 2
◯ 3
◯ 4
◯ 5
◯ 6
◯ 7
◯ 8
◯ 9
◯ 10
◯ 11
◯ 12
◯ 13
◯ 14
◯ 15
◯ 16
◯ 17
◯ 18
◯ 19
◯ 20
◯ 21
◯ 22
◯ 23
◯ 24
◯ 25
◯ 26
◯ 27
◯ 28
◯ 29
◯ 30
◯ 31
◯ 32
◯ 33
◯ 34
◯ 35
◯ 36
◯ 37
◯ 38
◯ 39
◯ 40
◯ 41
◯ 42
◯ 43
◯ 44
◯ 45
◯ 46
◯ 47
◯ 48
◯ 49
◯ 50
◯ 51
◯ 52
◯ 53
◯ 54
◯ 55
◯ 56
◯ 57
◯ 58
◯ 59
◯ 60
◯ 61
◯ 62
◯ 63
◯ 64
◯ 65
◯ 66
◯ 67
◯ 68

◯ 69
◯ 70
◯ 71
◯ 72
◯ 73
◯ 74
◯ 75
◯ 76
◯ 77
◯ 78
◯ 79
◯ 80
◯ 81
◯ 82
◯ 83
◯ 84
◯ 85
◯ 86
◯ 87
◯ 88
◯ 89
◯ 90
◯ 91
◯ 92
◯ 93
◯ 94
◯ 95
◯ 96
◯ 97
◯ 98
◯ 99
◯ Don't know

Do you still have gout?

◯ Yes
◯ No
◯ Don't know

Do you still have osteoarthritis?

◯ Yes
◯ No
◯ Don't know

Do you still have rheumatoid arthritis?

◯ Yes
◯ No
◯ Don't know

Do you still have this condition?

◯ Yes
◯ No
◯ Don't know

How is gout treated?
(Tick all that apply)

☐ no treatment ☐ diet ☐ exercise ☐ medication (please specify) ☐ natural/herbal therapy
☐ weight management ☐ supplements (please specify) ☐ other (please specify) ☐ Don't know

---

please specify the medication

____

---

please specify the supplements

____

please specify the treatment

____

How is osteoarthritis treated?
(Tick all that apply)

☐ no treatment ☐ diet ☐ exercise ☐ medication (please specify) ☐ natural/herbal therapy
☐ weight management ☐ supplements (please specify) ☐ other (please specify) ☐ Don't know

please specify the medication

____

please specify the supplements

____

please specify the treatment

____

How is rheumatoid arthritis treated?
(Tick all that apply)

☐ no treatment ☐ diet ☐ exercise ☐ medication (please specify) ☐ natural/herbal therapy
☐ weight management ☐ supplements (please specify) ☐ other (please specify) ☐ Don't know

please specify the medication

____

please specify the supplements

____

please specify the treatment

____

How is this condition treated?
(Tick all that apply)

☐ no treatment ☐ diet ☐ exercise ☐ medication (please specify) ☐ natural/herbal therapy
☐ weight management ☐ supplements (please specify) ☐ other (please specify) ☐ Don't know

please specify the medication

____

please specify the supplements

____

please specify the treatment

____

Have either of your parents, or any of brothers or sisters ever been told by a doctor (GP or specialist) or nurse that they have gout, osteoarthritis or rheumatoid arthritis?

◯ Yes
◯ No
◯ Don't know

Have you ever been told by a doctor (GP or specialist) or nurse that you have any type of cancer?

◯ Yes
◯ No
◯ Don't know

What type/s of cancer were you told you had?
(Tick all that apply)

☐ Bladder/kidney cancer
☐ Breast cancer
☐ Cancer of unknown primary site
☐ Cervical cancer
☐ Cancer of other female reproductive organs (include uterus, ovary)
☐ Colon/rectum/bowel cancer (colorectal)
☐ Leukaemia
☐ Lung cancer (include trachea, pleura and bronchus)
☐ Non-Hodgkin lymphoma
☐ Other type of lymphoma
☐ Prostate cancer
☐ Skin cancer (include melanoma, basal cell carcinoma, squamous cell carcinoma)
☐ Stomach cancer
☐ Other cancer (please specify)
☐ Don't Know

please specify the type of cancer

____

Do you have another type of cancer need to be specified?

○ Yes ○ No

please specify the second type of cancer

____

Do you have another type of cancer need to be specified?

○ Yes ○ No

please specify the type of cancer

____

At what age were you first told you had skin cancer (include melanoma, basal cell carcinoma, squamous cell carcinoma)?

◯ 0
◯ 1
◯ 2
◯ 3
◯ 4
◯ 5
◯ 6
◯ 7
◯ 8
◯ 9
◯ 10
◯ 11
◯ 12
◯ 13
◯ 14
◯ 15
◯ 16
◯ 17
◯ 18
◯ 19
◯ 20
◯ 21
◯ 22
◯ 23
◯ 24
◯ 25
◯ 26
◯ 27
◯ 28
◯ 29
◯ 30
◯ 31
◯ 32
◯ 33
◯ 34
◯ 35
◯ 36
◯ 37
◯ 38
◯ 39
◯ 40
◯ 41
◯ 42
◯ 43
◯ 44
◯ 45
◯ 46
◯ 47
◯ 48
◯ 49
◯ 50
◯ 51
◯ 52
◯ 53
◯ 54
◯ 55
◯ 56
◯ 57
◯ 58
◯ 59
◯ 60
◯ 61
◯ 62
◯ 63
◯ 64
◯ 65
◯ 66
◯ 67
◯ 68

◯ 69
◯ 70
◯ 71
◯ 72
◯ 73
◯ 74
◯ 75
◯ 76
◯ 77
◯ 78
◯ 79
◯ 80
◯ 81
◯ 82
◯ 83
◯ 84
◯ 85
◯ 86
◯ 87
◯ 88
◯ 89
◯ 90
◯ 91
◯ 92
◯ 93
◯ 94
◯ 95
◯ 96
◯ 97
◯ 98
◯ 99
◯ Don't know

At what age were you first told you had colon/rectum/bowel cancer (colorectal)?

◯ 0
◯ 1
◯ 2
◯ 3
◯ 4
◯ 5
◯ 6
◯ 7
◯ 8
◯ 9
◯ 10
◯ 11
◯ 12
◯ 13
◯ 14
◯ 15
◯ 16
◯ 17
◯ 18
◯ 19
◯ 20
◯ 21
◯ 22
◯ 23
◯ 24
◯ 25
◯ 26
◯ 27
◯ 28
◯ 29
◯ 30
◯ 31
◯ 32
◯ 33
◯ 34
◯ 35
◯ 36
◯ 37
◯ 38
◯ 39
◯ 40
◯ 41
◯ 42
◯ 43
◯ 44
◯ 45
◯ 46
◯ 47
◯ 48
◯ 49
◯ 50
◯ 51
◯ 52
◯ 53
◯ 54
◯ 55
◯ 56
◯ 57
◯ 58
◯ 59
◯ 60
◯ 61
◯ 62
◯ 63
◯ 64
◯ 65
◯ 66
◯ 67
◯ 68

◯ 69
◯ 70
◯ 71
◯ 72
◯ 73
◯ 74
◯ 75
◯ 76
◯ 77
◯ 78
◯ 79
◯ 80
◯ 81
◯ 82
◯ 83
◯ 84
◯ 85
◯ 86
◯ 87
◯ 88
◯ 89
◯ 90
◯ 91
◯ 92
◯ 93
◯ 94
◯ 95
◯ 96
◯ 97
◯ 98
◯ 99
◯ Don't know

At what age were you first told you had breast cancer?

- ◯ 0
- ◯ 1
- ◯ 2
- ◯ 3
- ◯ 4
- ◯ 5
- ◯ 6
- ◯ 7
- ◯ 8
- ◯ 9
- ◯ 10
- ◯ 11
- ◯ 12
- ◯ 13
- ◯ 14
- ◯ 15
- ◯ 16
- ◯ 17
- ◯ 18
- ◯ 19
- ◯ 20
- ◯ 21
- ◯ 22
- ◯ 23
- ◯ 24
- ◯ 25
- ◯ 26
- ◯ 27
- ◯ 28
- ◯ 29
- ◯ 30
- ◯ 31
- ◯ 32
- ◯ 33
- ◯ 34
- ◯ 35
- ◯ 36
- ◯ 37
- ◯ 38
- ◯ 39
- ◯ 40
- ◯ 41
- ◯ 42
- ◯ 43
- ◯ 44
- ◯ 45
- ◯ 46
- ◯ 47
- ◯ 48
- ◯ 49
- ◯ 50
- ◯ 51
- ◯ 52
- ◯ 53
- ◯ 54
- ◯ 55
- ◯ 56
- ◯ 57
- ◯ 58
- ◯ 59
- ◯ 60
- ◯ 61
- ◯ 62
- ◯ 63
- ◯ 64
- ◯ 65
- ◯ 66
- ◯ 67
- ◯ 68

- ◯ 69
- ◯ 70
- ◯ 71
- ◯ 72
- ◯ 73
- ◯ 74
- ◯ 75
- ◯ 76
- ◯ 77
- ◯ 78
- ◯ 79
- ◯ 80
- ◯ 81
- ◯ 82
- ◯ 83
- ◯ 84
- ◯ 85
- ◯ 86
- ◯ 87
- ◯ 88
- ◯ 89
- ◯ 90
- ◯ 91
- ◯ 92
- ◯ 93
- ◯ 94
- ◯ 95
- ◯ 96
- ◯ 97
- ◯ 98
- ◯ 99
- ◯ Don't know

At what age were you first told you had prostate cancer?

- ◯ 0
- ◯ 1
- ◯ 2
- ◯ 3
- ◯ 4
- ◯ 5
- ◯ 6
- ◯ 7
- ◯ 8
- ◯ 9
- ◯ 10
- ◯ 11
- ◯ 12
- ◯ 13
- ◯ 14
- ◯ 15
- ◯ 16
- ◯ 17
- ◯ 18
- ◯ 19
- ◯ 20
- ◯ 21
- ◯ 22
- ◯ 23
- ◯ 24
- ◯ 25
- ◯ 26
- ◯ 27
- ◯ 28
- ◯ 29
- ◯ 30
- ◯ 31
- ◯ 32
- ◯ 33
- ◯ 34
- ◯ 35
- ◯ 36
- ◯ 37
- ◯ 38
- ◯ 39
- ◯ 40
- ◯ 41
- ◯ 42
- ◯ 43
- ◯ 44
- ◯ 45
- ◯ 46
- ◯ 47
- ◯ 48
- ◯ 49
- ◯ 50
- ◯ 51
- ◯ 52
- ◯ 53
- ◯ 54
- ◯ 55
- ◯ 56
- ◯ 57
- ◯ 58
- ◯ 59
- ◯ 60
- ◯ 61
- ◯ 62
- ◯ 63
- ◯ 64
- ◯ 65
- ◯ 66
- ◯ 67
- ◯ 68

- ◯ 69
- ◯ 70
- ◯ 71
- ◯ 72
- ◯ 73
- ◯ 74
- ◯ 75
- ◯ 76
- ◯ 77
- ◯ 78
- ◯ 79
- ◯ 80
- ◯ 81
- ◯ 82
- ◯ 83
- ◯ 84
- ◯ 85
- ◯ 86
- ◯ 87
- ◯ 88
- ◯ 89
- ◯ 90
- ◯ 91
- ◯ 92
- ◯ 93
- ◯ 94
- ◯ 95
- ◯ 96
- ◯ 97
- ◯ 98
- ◯ 99
- ◯ Don't know

At what age were you first told you had lung cancer (include trachea, pleura and bronchus)?

◯ 0
◯ 1
◯ 2
◯ 3
◯ 4
◯ 5
◯ 6
◯ 7
◯ 8
◯ 9
◯ 10
◯ 11
◯ 12
◯ 13
◯ 14
◯ 15
◯ 16
◯ 17
◯ 18
◯ 19
◯ 20
◯ 21
◯ 22
◯ 23
◯ 24
◯ 25
◯ 26
◯ 27
◯ 28
◯ 29
◯ 30
◯ 31
◯ 32
◯ 33
◯ 34
◯ 35
◯ 36
◯ 37
◯ 38
◯ 39
◯ 40
◯ 41
◯ 42
◯ 43
◯ 44
◯ 45
◯ 46
◯ 47
◯ 48
◯ 49
◯ 50
◯ 51
◯ 52
◯ 53
◯ 54
◯ 55
◯ 56
◯ 57
◯ 58
◯ 59
◯ 60
◯ 61
◯ 62
◯ 63
◯ 64
◯ 65
◯ 66
◯ 67
◯ 68

- ◯ 69
- ◯ 70
- ◯ 71
- ◯ 72
- ◯ 73
- ◯ 74
- ◯ 75
- ◯ 76
- ◯ 77
- ◯ 78
- ◯ 79
- ◯ 80
- ◯ 81
- ◯ 82
- ◯ 83
- ◯ 84
- ◯ 85
- ◯ 86
- ◯ 87
- ◯ 88
- ◯ 89
- ◯ 90
- ◯ 91
- ◯ 92
- ◯ 93
- ◯ 94
- ◯ 95
- ◯ 96
- ◯ 97
- ◯ 98
- ◯ 99
- ◯ Don't know

At what age were you first told you had cervical cancer?

- ◯ 0
- ◯ 1
- ◯ 2
- ◯ 3
- ◯ 4
- ◯ 5
- ◯ 6
- ◯ 7
- ◯ 8
- ◯ 9
- ◯ 10
- ◯ 11
- ◯ 12
- ◯ 13
- ◯ 14
- ◯ 15
- ◯ 16
- ◯ 17
- ◯ 18
- ◯ 19
- ◯ 20
- ◯ 21
- ◯ 22
- ◯ 23
- ◯ 24
- ◯ 25
- ◯ 26
- ◯ 27
- ◯ 28
- ◯ 29
- ◯ 30
- ◯ 31
- ◯ 32
- ◯ 33
- ◯ 34
- ◯ 35
- ◯ 36
- ◯ 37
- ◯ 38
- ◯ 39
- ◯ 40
- ◯ 41
- ◯ 42
- ◯ 43
- ◯ 44
- ◯ 45
- ◯ 46
- ◯ 47
- ◯ 48
- ◯ 49
- ◯ 50
- ◯ 51
- ◯ 52
- ◯ 53
- ◯ 54
- ◯ 55
- ◯ 56
- ◯ 57
- ◯ 58
- ◯ 59
- ◯ 60
- ◯ 61
- ◯ 62
- ◯ 63
- ◯ 64
- ◯ 65
- ◯ 66
- ◯ 67
- ◯ 68

- ◯ 69
- ◯ 70
- ◯ 71
- ◯ 72
- ◯ 73
- ◯ 74
- ◯ 75
- ◯ 76
- ◯ 77
- ◯ 78
- ◯ 79
- ◯ 80
- ◯ 81
- ◯ 82
- ◯ 83
- ◯ 84
- ◯ 85
- ◯ 86
- ◯ 87
- ◯ 88
- ◯ 89
- ◯ 90
- ◯ 91
- ◯ 92
- ◯ 93
- ◯ 94
- ◯ 95
- ◯ 96
- ◯ 97
- ◯ 98
- ◯ 99
- ◯ Don't know

At what age were you first told you had cancer of other female reproductive organs (include uterus, ovary) ?

◯ 0
◯ 1
◯ 2
◯ 3
◯ 4
◯ 5
◯ 6
◯ 7
◯ 8
◯ 9
◯ 10
◯ 11
◯ 12
◯ 13
◯ 14
◯ 15
◯ 16
◯ 17
◯ 18
◯ 19
◯ 20
◯ 21
◯ 22
◯ 23
◯ 24
◯ 25
◯ 26
◯ 27
◯ 28
◯ 29
◯ 30
◯ 31
◯ 32
◯ 33
◯ 34
◯ 35
◯ 36
◯ 37
◯ 38
◯ 39
◯ 40
◯ 41
◯ 42
◯ 43
◯ 44
◯ 45
◯ 46
◯ 47
◯ 48
◯ 49
◯ 50
◯ 51
◯ 52
◯ 53
◯ 54
◯ 55
◯ 56
◯ 57
◯ 58
◯ 59
◯ 60
◯ 61
◯ 62
◯ 63
◯ 64
◯ 65
◯ 66
◯ 67
◯ 68

- ◯ 69
- ◯ 70
- ◯ 71
- ◯ 72
- ◯ 73
- ◯ 74
- ◯ 75
- ◯ 76
- ◯ 77
- ◯ 78
- ◯ 79
- ◯ 80
- ◯ 81
- ◯ 82
- ◯ 83
- ◯ 84
- ◯ 85
- ◯ 86
- ◯ 87
- ◯ 88
- ◯ 89
- ◯ 90
- ◯ 91
- ◯ 92
- ◯ 93
- ◯ 94
- ◯ 95
- ◯ 96
- ◯ 97
- ◯ 98
- ◯ 99
- ◯ Don't know

At what age were you first told you had bladder/kidney cancer?

- ◯ 0
- ◯ 1
- ◯ 2
- ◯ 3
- ◯ 4
- ◯ 5
- ◯ 6
- ◯ 7
- ◯ 8
- ◯ 9
- ◯ 10
- ◯ 11
- ◯ 12
- ◯ 13
- ◯ 14
- ◯ 15
- ◯ 16
- ◯ 17
- ◯ 18
- ◯ 19
- ◯ 20
- ◯ 21
- ◯ 22
- ◯ 23
- ◯ 24
- ◯ 25
- ◯ 26
- ◯ 27
- ◯ 28
- ◯ 29
- ◯ 30
- ◯ 31
- ◯ 32
- ◯ 33
- ◯ 34
- ◯ 35
- ◯ 36
- ◯ 37
- ◯ 38
- ◯ 39
- ◯ 40
- ◯ 41
- ◯ 42
- ◯ 43
- ◯ 44
- ◯ 45
- ◯ 46
- ◯ 47
- ◯ 48
- ◯ 49
- ◯ 50
- ◯ 51
- ◯ 52
- ◯ 53
- ◯ 54
- ◯ 55
- ◯ 56
- ◯ 57
- ◯ 58
- ◯ 59
- ◯ 60
- ◯ 61
- ◯ 62
- ◯ 63
- ◯ 64
- ◯ 65
- ◯ 66
- ◯ 67
- ◯ 68

◯ 69
◯ 70
◯ 71
◯ 72
◯ 73
◯ 74
◯ 75
◯ 76
◯ 77
◯ 78
◯ 79
◯ 80
◯ 81
◯ 82
◯ 83
◯ 84
◯ 85
◯ 86
◯ 87
◯ 88
◯ 89
◯ 90
◯ 91
◯ 92
◯ 93
◯ 94
◯ 95
◯ 96
◯ 97
◯ 98
◯ 99
◯ Don't know

At what age were you first told you had stomach cancer?

◯ 0
◯ 1
◯ 2
◯ 3
◯ 4
◯ 5
◯ 6
◯ 7
◯ 8
◯ 9
◯ 10
◯ 11
◯ 12
◯ 13
◯ 14
◯ 15
◯ 16
◯ 17
◯ 18
◯ 19
◯ 20
◯ 21
◯ 22
◯ 23
◯ 24
◯ 25
◯ 26
◯ 27
◯ 28
◯ 29
◯ 30
◯ 31
◯ 32
◯ 33
◯ 34
◯ 35
◯ 36
◯ 37
◯ 38
◯ 39
◯ 40
◯ 41
◯ 42
◯ 43
◯ 44
◯ 45
◯ 46
◯ 47
◯ 48
◯ 49
◯ 50
◯ 51
◯ 52
◯ 53
◯ 54
◯ 55
◯ 56
◯ 57
◯ 58
◯ 59
◯ 60
◯ 61
◯ 62
◯ 63
◯ 64
◯ 65
◯ 66
◯ 67
◯ 68

◯ 69
◯ 70
◯ 71
◯ 72
◯ 73
◯ 74
◯ 75
◯ 76
◯ 77
◯ 78
◯ 79
◯ 80
◯ 81
◯ 82
◯ 83
◯ 84
◯ 85
◯ 86
◯ 87
◯ 88
◯ 89
◯ 90
◯ 91
◯ 92
◯ 93
◯ 94
◯ 95
◯ 96
◯ 97
◯ 98
◯ 99
◯ Don't know

At what age were you first told you had leukaemia?

- ◯ 0
- ◯ 1
- ◯ 2
- ◯ 3
- ◯ 4
- ◯ 5
- ◯ 6
- ◯ 7
- ◯ 8
- ◯ 9
- ◯ 10
- ◯ 11
- ◯ 12
- ◯ 13
- ◯ 14
- ◯ 15
- ◯ 16
- ◯ 17
- ◯ 18
- ◯ 19
- ◯ 20
- ◯ 21
- ◯ 22
- ◯ 23
- ◯ 24
- ◯ 25
- ◯ 26
- ◯ 27
- ◯ 28
- ◯ 29
- ◯ 30
- ◯ 31
- ◯ 32
- ◯ 33
- ◯ 34
- ◯ 35
- ◯ 36
- ◯ 37
- ◯ 38
- ◯ 39
- ◯ 40
- ◯ 41
- ◯ 42
- ◯ 43
- ◯ 44
- ◯ 45
- ◯ 46
- ◯ 47
- ◯ 48
- ◯ 49
- ◯ 50
- ◯ 51
- ◯ 52
- ◯ 53
- ◯ 54
- ◯ 55
- ◯ 56
- ◯ 57
- ◯ 58
- ◯ 59
- ◯ 60
- ◯ 61
- ◯ 62
- ◯ 63
- ◯ 64
- ◯ 65
- ◯ 66
- ◯ 67
- ◯ 68

○ 69
○ 70
○ 71
○ 72
○ 73
○ 74
○ 75
○ 76
○ 77
○ 78
○ 79
○ 80
○ 81
○ 82
○ 83
○ 84
○ 85
○ 86
○ 87
○ 88
○ 89
○ 90
○ 91
○ 92
○ 93
○ 94
○ 95
○ 96
○ 97
○ 98
○ 99
○ Don't know

At what age were you first told you had non-Hodgkin lymphoma

◯ 0
◯ 1
◯ 2
◯ 3
◯ 4
◯ 5
◯ 6
◯ 7
◯ 8
◯ 9
◯ 10
◯ 11
◯ 12
◯ 13
◯ 14
◯ 15
◯ 16
◯ 17
◯ 18
◯ 19
◯ 20
◯ 21
◯ 22
◯ 23
◯ 24
◯ 25
◯ 26
◯ 27
◯ 28
◯ 29
◯ 30
◯ 31
◯ 32
◯ 33
◯ 34
◯ 35
◯ 36
◯ 37
◯ 38
◯ 39
◯ 40
◯ 41
◯ 42
◯ 43
◯ 44
◯ 45
◯ 46
◯ 47
◯ 48
◯ 49
◯ 50
◯ 51
◯ 52
◯ 53
◯ 54
◯ 55
◯ 56
◯ 57
◯ 58
◯ 59
◯ 60
◯ 61
◯ 62
◯ 63
◯ 64
◯ 65
◯ 66
◯ 67
◯ 68

- ◯ 69
- ◯ 70
- ◯ 71
- ◯ 72
- ◯ 73
- ◯ 74
- ◯ 75
- ◯ 76
- ◯ 77
- ◯ 78
- ◯ 79
- ◯ 80
- ◯ 81
- ◯ 82
- ◯ 83
- ◯ 84
- ◯ 85
- ◯ 86
- ◯ 87
- ◯ 88
- ◯ 89
- ◯ 90
- ◯ 91
- ◯ 92
- ◯ 93
- ◯ 94
- ◯ 95
- ◯ 96
- ◯ 97
- ◯ 98
- ◯ 99
- ◯ Don't know

At what age were you first told you had other type of lymphoma?

- ◯ 0
- ◯ 1
- ◯ 2
- ◯ 3
- ◯ 4
- ◯ 5
- ◯ 6
- ◯ 7
- ◯ 8
- ◯ 9
- ◯ 10
- ◯ 11
- ◯ 12
- ◯ 13
- ◯ 14
- ◯ 15
- ◯ 16
- ◯ 17
- ◯ 18
- ◯ 19
- ◯ 20
- ◯ 21
- ◯ 22
- ◯ 23
- ◯ 24
- ◯ 25
- ◯ 26
- ◯ 27
- ◯ 28
- ◯ 29
- ◯ 30
- ◯ 31
- ◯ 32
- ◯ 33
- ◯ 34
- ◯ 35
- ◯ 36
- ◯ 37
- ◯ 38
- ◯ 39
- ◯ 40
- ◯ 41
- ◯ 42
- ◯ 43
- ◯ 44
- ◯ 45
- ◯ 46
- ◯ 47
- ◯ 48
- ◯ 49
- ◯ 50
- ◯ 51
- ◯ 52
- ◯ 53
- ◯ 54
- ◯ 55
- ◯ 56
- ◯ 57
- ◯ 58
- ◯ 59
- ◯ 60
- ◯ 61
- ◯ 62
- ◯ 63
- ◯ 64
- ◯ 65
- ◯ 66
- ◯ 67
- ◯ 68

◯ 69
◯ 70
◯ 71
◯ 72
◯ 73
◯ 74
◯ 75
◯ 76
◯ 77
◯ 78
◯ 79
◯ 80
◯ 81
◯ 82
◯ 83
◯ 84
◯ 85
◯ 86
◯ 87
◯ 88
◯ 89
◯ 90
◯ 91
◯ 92
◯ 93
◯ 94
◯ 95
◯ 96
◯ 97
◯ 98
◯ 99
◯ Don't know

At what age were you first told you had cancer of unknown primary site?

- ◯ 0
- ◯ 1
- ◯ 2
- ◯ 3
- ◯ 4
- ◯ 5
- ◯ 6
- ◯ 7
- ◯ 8
- ◯ 9
- ◯ 10
- ◯ 11
- ◯ 12
- ◯ 13
- ◯ 14
- ◯ 15
- ◯ 16
- ◯ 17
- ◯ 18
- ◯ 19
- ◯ 20
- ◯ 21
- ◯ 22
- ◯ 23
- ◯ 24
- ◯ 25
- ◯ 26
- ◯ 27
- ◯ 28
- ◯ 29
- ◯ 30
- ◯ 31
- ◯ 32
- ◯ 33
- ◯ 34
- ◯ 35
- ◯ 36
- ◯ 37
- ◯ 38
- ◯ 39
- ◯ 40
- ◯ 41
- ◯ 42
- ◯ 43
- ◯ 44
- ◯ 45
- ◯ 46
- ◯ 47
- ◯ 48
- ◯ 49
- ◯ 50
- ◯ 51
- ◯ 52
- ◯ 53
- ◯ 54
- ◯ 55
- ◯ 56
- ◯ 57
- ◯ 58
- ◯ 59
- ◯ 60
- ◯ 61
- ◯ 62
- ◯ 63
- ◯ 64
- ◯ 65
- ◯ 66
- ◯ 67
- ◯ 68

◯ 69
◯ 70
◯ 71
◯ 72
◯ 73
◯ 74
◯ 75
◯ 76
◯ 77
◯ 78
◯ 79
◯ 80
◯ 81
◯ 82
◯ 83
◯ 84
◯ 85
◯ 86
◯ 87
◯ 88
◯ 89
◯ 90
◯ 91
◯ 92
◯ 93
◯ 94
◯ 95
◯ 96
◯ 97
◯ 98
◯ 99
◯ Don't know

At what age were you first told you had [cancer_others]?

◯ 0
◯ 1
◯ 2
◯ 3
◯ 4
◯ 5
◯ 6
◯ 7
◯ 8
◯ 9
◯ 10
◯ 11
◯ 12
◯ 13
◯ 14
◯ 15
◯ 16
◯ 17
◯ 18
◯ 19
◯ 20
◯ 21
◯ 22
◯ 23
◯ 24
◯ 25
◯ 26
◯ 27
◯ 28
◯ 29
◯ 30
◯ 31
◯ 32
◯ 33
◯ 34
◯ 35
◯ 36
◯ 37
◯ 38
◯ 39
◯ 40
◯ 41
◯ 42
◯ 43
◯ 44
◯ 45
◯ 46
◯ 47
◯ 48
◯ 49
◯ 50
◯ 51
◯ 52
◯ 53
◯ 54
◯ 55
◯ 56
◯ 57
◯ 58
◯ 59
◯ 60
◯ 61
◯ 62
◯ 63
◯ 64
◯ 65
◯ 66
◯ 67
◯ 68

◯ 69
◯ 70
◯ 71
◯ 72
◯ 73
◯ 74
◯ 75
◯ 76
◯ 77
◯ 78
◯ 79
◯ 80
◯ 81
◯ 82
◯ 83
◯ 84
◯ 85
◯ 86
◯ 87
◯ 88
◯ 89
◯ 90
◯ 91
◯ 92
◯ 93
◯ 94
◯ 95
◯ 96
◯ 97
◯ 98
◯ 99
◯ Don't know

At what age were you first told you had this condition?

◯ 0
◯ 1
◯ 2
◯ 3
◯ 4
◯ 5
◯ 6
◯ 7
◯ 8
◯ 9
◯ 10
◯ 11
◯ 12
◯ 13
◯ 14
◯ 15
◯ 16
◯ 17
◯ 18
◯ 19
◯ 20
◯ 21
◯ 22
◯ 23
◯ 24
◯ 25
◯ 26
◯ 27
◯ 28
◯ 29
◯ 30
◯ 31
◯ 32
◯ 33
◯ 34
◯ 35
◯ 36
◯ 37
◯ 38
◯ 39
◯ 40
◯ 41
◯ 42
◯ 43
◯ 44
◯ 45
◯ 46
◯ 47
◯ 48
◯ 49
◯ 50
◯ 51
◯ 52
◯ 53
◯ 54
◯ 55
◯ 56
◯ 57
◯ 58
◯ 59
◯ 60
◯ 61
◯ 62
◯ 63
◯ 64
◯ 65
◯ 66
◯ 67
◯ 68

- ◯ 69
- ◯ 70
- ◯ 71
- ◯ 72
- ◯ 73
- ◯ 74
- ◯ 75
- ◯ 76
- ◯ 77
- ◯ 78
- ◯ 79
- ◯ 80
- ◯ 81
- ◯ 82
- ◯ 83
- ◯ 84
- ◯ 85
- ◯ 86
- ◯ 87
- ◯ 88
- ◯ 89
- ◯ 90
- ◯ 91
- ◯ 92
- ◯ 93
- ◯ 94
- ◯ 95
- ◯ 96
- ◯ 97
- ◯ 98
- ◯ 99
- ◯ Don't know

At what age were you first told you had [cancer_others_2]?

◯ 0
◯ 1
◯ 2
◯ 3
◯ 4
◯ 5
◯ 6
◯ 7
◯ 8
◯ 9
◯ 10
◯ 11
◯ 12
◯ 13
◯ 14
◯ 15
◯ 16
◯ 17
◯ 18
◯ 19
◯ 20
◯ 21
◯ 22
◯ 23
◯ 24
◯ 25
◯ 26
◯ 27
◯ 28
◯ 29
◯ 30
◯ 31
◯ 32
◯ 33
◯ 34
◯ 35
◯ 36
◯ 37
◯ 38
◯ 39
◯ 40
◯ 41
◯ 42
◯ 43
◯ 44
◯ 45
◯ 46
◯ 47
◯ 48
◯ 49
◯ 50
◯ 51
◯ 52
◯ 53
◯ 54
◯ 55
◯ 56
◯ 57
◯ 58
◯ 59
◯ 60
◯ 61
◯ 62
◯ 63
◯ 64
◯ 65
◯ 66
◯ 67
◯ 68

◯ 69
◯ 70
◯ 71
◯ 72
◯ 73
◯ 74
◯ 75
◯ 76
◯ 77
◯ 78
◯ 79
◯ 80
◯ 81
◯ 82
◯ 83
◯ 84
◯ 85
◯ 86
◯ 87
◯ 88
◯ 89
◯ 90
◯ 91
◯ 92
◯ 93
◯ 94
◯ 95
◯ 96
◯ 97
◯ 98
◯ 99
◯ Don't know

At what age were you first told you had [cancer_others_3]?

- ◯ 0
- ◯ 1
- ◯ 2
- ◯ 3
- ◯ 4
- ◯ 5
- ◯ 6
- ◯ 7
- ◯ 8
- ◯ 9
- ◯ 10
- ◯ 11
- ◯ 12
- ◯ 13
- ◯ 14
- ◯ 15
- ◯ 16
- ◯ 17
- ◯ 18
- ◯ 19
- ◯ 20
- ◯ 21
- ◯ 22
- ◯ 23
- ◯ 24
- ◯ 25
- ◯ 26
- ◯ 27
- ◯ 28
- ◯ 29
- ◯ 30
- ◯ 31
- ◯ 32
- ◯ 33
- ◯ 34
- ◯ 35
- ◯ 36
- ◯ 37
- ◯ 38
- ◯ 39
- ◯ 40
- ◯ 41
- ◯ 42
- ◯ 43
- ◯ 44
- ◯ 45
- ◯ 46
- ◯ 47
- ◯ 48
- ◯ 49
- ◯ 50
- ◯ 51
- ◯ 52
- ◯ 53
- ◯ 54
- ◯ 55
- ◯ 56
- ◯ 57
- ◯ 58
- ◯ 59
- ◯ 60
- ◯ 61
- ◯ 62
- ◯ 63
- ◯ 64
- ◯ 65
- ◯ 66
- ◯ 67
- ◯ 68

◯ 69
◯ 70
◯ 71
◯ 72
◯ 73
◯ 74
◯ 75
◯ 76
◯ 77
◯ 78
◯ 79
◯ 80
◯ 81
◯ 82
◯ 83
◯ 84
◯ 85
◯ 86
◯ 87
◯ 88
◯ 89
◯ 90
◯ 91
◯ 92
◯ 93
◯ 94
◯ 95
◯ 96
◯ 97
◯ 98
◯ 99
◯ Don't know

Do you still have Skin cancer (include melanoma, basal cell carcinoma, squamous cell carcinoma)?	◯ Yes<br>◯ No<br>◯ Don't know
Do you still have colon/rectum/bowel cancer (colorectal)?	◯ Yes<br>◯ No<br>◯ Don't know
Do you still have breast cancer?	◯ Yes<br>◯ No<br>◯ Don't know
Do you still have prostate cancer?	◯ Yes<br>◯ No<br>◯ Don't know
Do you still have lung cancer (include trachea, pleura and bronchus?	◯ Yes<br>◯ No<br>◯ Don't know
Do you still have cervical cancer?	◯ Yes<br>◯ No<br>◯ Don't know
Do you still have cancer of other female reproductive organs (include uterus, ovary)?	◯ Yes<br>◯ No<br>◯ Don't know

Do you still have bladder/kidney cancer?	◯ Yes<br>◯ No<br>◯ Don't know
Do you still have stomach cancer?	◯ Yes<br>◯ No<br>◯ Don't know
Do you still have leukaemia?	◯ Yes<br>◯ No<br>◯ Don't know
Do you still have non- Hodgkin leukaemia?	◯ Yes<br>◯ No<br>◯ Don't know
Do you still have other type of lymphoma?	◯ Yes<br>◯ No<br>◯ Don't know
Do you still have cancer of unknown primary site?	◯ Yes<br>◯ No<br>◯ Don't know
Do you still have [cancer_others]?	◯ Yes<br>◯ No<br>◯ Don't know
Do you still have this condition?	◯ Yes<br>◯ No<br>◯ Don't know
Do you still have [cancer_others_2]?	◯ Yes<br>◯ No<br>◯ Don't know
Do you still have [cancer_others_3]?	◯ Yes<br>◯ No<br>◯ Don't know

How was/is skin cancer (include melanoma, basal cell carcinoma, squamous cell carcinoma) treated?
(Tick all that apply)

- ☐ no treatment
- ☐ diet
- ☐ exercise
- ☐ medication/chemotherapy
- ☐ natural/herbal therapy
- ☐ radiotherapy
- ☐ supplements (please specify)
- ☐ surgery
- ☐ weight management
- ☐ other (please specify)
- ☐ Don't know

please specify the supplements ____________________

please specify the treatment

____

How was/is colon/rectum/bowel cancer (colorectal) treated?
(Tick all that apply)

- ☐ no treatment
- ☐ diet
- ☐ exercise
- ☐ medication/chemotherapy
- ☐ natural/herbal therapy
- ☐ radiotherapy
- ☐ supplements (please specify)
- ☐ surgery
- ☐ weight management
- ☐ other (please specify)
- ☐ Don't know

please specify the supplements

____

please specify the treatment

____

How was/is breast cancer treated?
(Tick all that apply)

- ☐ no treatment
- ☐ diet
- ☐ exercise
- ☐ medication/chemotherapy
- ☐ natural/herbal therapy
- ☐ radiotherapy
- ☐ supplements (please specify)
- ☐ surgery
- ☐ weight management
- ☐ other (please specify)
- ☐ Don't know

please specify the supplements

____

please specify the treatment

____

How was/is prostate cancer treated?
(Tick all that apply)

- ☐ no treatment
- ☐ diet
- ☐ exercise
- ☐ medication/chemotherapy
- ☐ natural/herbal therapy
- ☐ radiotherapy
- ☐ supplements (please specify)
- ☐ surgery
- ☐ weight management
- ☐ other (please specify)
- ☐ Don't know

please specify the supplements

____

please specify the treatment

____

How was/is lung cancer (include trachea, pleura and bronchus) treated?
(Tick all that apply)

- ☐ no treatment
- ☐ diet
- ☐ exercise
- ☐ medication/chemotherapy
- ☐ natural/herbal therapy
- ☐ radiotherapy
- ☐ supplements (please specify)
- ☐ surgery
- ☐ weight management
- ☐ other (please specify)
- ☐ Don't know

please specify the supplements

____

please specify the treatment

____

How was/is cervical cancer treated?
(Tick all that apply)

- ☐ no treatment
- ☐ diet
- ☐ exercise
- ☐ medication/chemotherapy
- ☐ natural/herbal therapy
- ☐ radiotherapy
- ☐ supplements (please specify)
- ☐ surgery
- ☐ weight management
- ☐ other (please specify)
- ☐ Don't know

please specify the supplements

____

please specify the treatment

____

How was/is cancer of other female reproductive organs (include uterus, ovary) treated?
(Tick all that apply)

- ☐ no treatment
- ☐ diet
- ☐ exercise
- ☐ medication/chemotherapy
- ☐ natural/herbal therapy
- ☐ radiotherapy
- ☐ supplements (please specify)
- ☐ surgery
- ☐ weight management
- ☐ other (please specify)
- ☐ Don't know

please specify the supplements ______________________

please specify the treatment ______________________

How was/is bladder/kidney
cancer treated?

- ☐ no treatment
- ☐ diet
- ☐ exercise
- ☐ medication/chemotherapy
- ☐ natural/herbal therapy
- ☐ radiotherapy
- ☐ supplements (please specify)
- ☐ surgery
- ☐ weight management
- ☐ other (please specify)
- ☐ Don't know

please specify the supplements ______________________

please specify the treatment ______________________

How was/is stomach cancer treated?
(Tick all that apply)

- ☐ no treatment
- ☐ diet
- ☐ exercise
- ☐ medication/chemotherapy
- ☐ natural/herbal therapy
- ☐ radiotherapy
- ☐ supplements (please specify)
- ☐ surgery
- ☐ weight management
- ☐ other (please specify)
- ☐ Don't know

please specify the supplements ______________________

please specify the treatment

____

How was/is leukaemia treated?
(Tick all that apply)

- ☐ no treatment
- ☐ diet
- ☐ exercise
- ☐ medication/chemotherapy
- ☐ natural/herbal therapy
- ☐ radiotherapy
- ☐ supplements (please specify)
- ☐ surgery
- ☐ weight management
- ☐ other (please specify)
- ☐ Don't know

please specify the supplements

____

please specify the treatment

____

How was/is non-Hodgkin lymphoma treated?
(Tick all that apply)

- ☐ no treatment
- ☐ diet
- ☐ exercise
- ☐ medication/chemotherapy
- ☐ natural/herbal therapy
- ☐ radiotherapy
- ☐ supplements (please specify)
- ☐ surgery
- ☐ weight management
- ☐ other (please specify)
- ☐ Don't know

please specify the supplements

____

please specify the treatment

____

How was/is other type of lymphoma treated?
(Tick all that apply)

- ☐ no treatment
- ☐ diet
- ☐ exercise
- ☐ medication/chemotherapy
- ☐ natural/herbal therapy
- ☐ radiotherapy
- ☐ supplements (please specify)
- ☐ surgery
- ☐ weight management
- ☐ other (please specify)
- ☐ Don't know

please specify the supplements

____

please specify the treatment

____

How was/is cancer of unknown primary site treated?
(Tick all that apply)

- ☐ no treatment
- ☐ diet
- ☐ exercise
- ☐ medication/chemotherapy
- ☐ natural/herbal therapy
- ☐ radiotherapy
- ☐ supplements (please specify)
- ☐ surgery
- ☐ weight management
- ☐ other (please specify)
- ☐ Don't know

please specify the supplements

____

please specify the treatment

____

How was/is [cancer_others] treated?
(Tick all that apply)

- ☐ no treatment
- ☐ diet
- ☐ exercise
- ☐ medication/chemotherapy
- ☐ natural/herbal therapy
- ☐ radiotherapy
- ☐ supplements (please specify)
- ☐ surgery
- ☐ weight management
- ☐ other (please specify)
- ☐ Don't know

please specify the supplements

____

please specify the treatment

____

How was/is this condition treated?
(Tick all that apply)

- ☐ no treatment
- ☐ diet
- ☐ exercise
- ☐ medication/chemotherapy
- ☐ natural/herbal therapy
- ☐ radiotherapy
- ☐ supplements (please specify)
- ☐ surgery
- ☐ weight management
- ☐ other (please specify)
- ☐ Don't know

please specify the supplements ________________________________

please specify the treatment ________________________________

please specify the treatment ________________________________

How was/is [cancer_others_2] treated?
(Tick all that apply)

- ☐ no treatment
- ☐ diet
- ☐ exercise
- ☐ medication/chemotherapy
- ☐ natural/herbal therapy
- ☐ radiotherapy
- ☐ supplements (please specify)
- ☐ surgery
- ☐ weight management
- ☐ other (please specify)
- ☐ Don't know

please specify the supplements ________________________________

please specify the treatment ________________________________

How was/is [cancer_others_3] treated?
(Tick all that apply)

- ☐ no treatment
- ☐ diet
- ☐ exercise
- ☐ medication/chemotherapy
- ☐ natural/herbal therapy
- ☐ radiotherapy
- ☐ supplements (please specify)
- ☐ surgery
- ☐ weight management
- ☐ other (please specify)
- ☐ Don't know

please specify the supplements

____

please specify the treatment

____

Have either of your parents, or any of brothers or sisters ever been told by a doctor (GP or specialist) or nurse that they have any type of cancer?

◯ Yes
◯ No
◯ Don't know

This question is about your mental and emotional well-being. Have you ever been told by a doctor (GP or specialist) or psychologist that you have depression, an anxiety condition, or memory loss?

◯ Yes
◯ No
◯ Don't know
◯ Prefer not to say

Which condition/s have been confirmed by a doctor (GP or specialist) or psychologist?
(Tick all that apply)

☐ Alzheimer's Disease
☐ Anxiety condition (such as post-traumatic stress disorder, panic attacks, obsessive compulsive disorder, phobias)
☐ Dementia
☐ Depression (including post-natal depression)
☐ Mild cognitive impairment (MCI)
☐ Other anxiety condition
☐ Other mood condition
☐ Other (please specify)
☐ Don't Know

please specify the condition

____

Do you have another mental condition need to be specified?

◯ Yes ◯ No

please specify the second condition

____

Do you have another mental condition need to be specified?

◯ Yes ◯ No

please specify the third condition

____

At what age were you first told you had depression (including post-natal depression)?

- ◯ 0
- ◯ 1
- ◯ 2
- ◯ 3
- ◯ 4
- ◯ 5
- ◯ 6
- ◯ 7
- ◯ 8
- ◯ 9
- ◯ 10
- ◯ 11
- ◯ 12
- ◯ 13
- ◯ 14
- ◯ 15
- ◯ 16
- ◯ 17
- ◯ 18
- ◯ 19
- ◯ 20
- ◯ 21
- ◯ 22
- ◯ 23
- ◯ 24
- ◯ 25
- ◯ 26
- ◯ 27
- ◯ 28
- ◯ 29
- ◯ 30
- ◯ 31
- ◯ 32
- ◯ 33
- ◯ 34
- ◯ 35
- ◯ 36
- ◯ 37
- ◯ 38
- ◯ 39
- ◯ 40
- ◯ 41
- ◯ 42
- ◯ 43
- ◯ 44
- ◯ 45
- ◯ 46
- ◯ 47
- ◯ 48
- ◯ 49
- ◯ 50
- ◯ 51
- ◯ 52
- ◯ 53
- ◯ 54
- ◯ 55
- ◯ 56
- ◯ 57
- ◯ 58
- ◯ 59
- ◯ 60
- ◯ 61
- ◯ 62
- ◯ 63
- ◯ 64
- ◯ 65
- ◯ 66
- ◯ 67
- ◯ 68

◯ 69
◯ 70
◯ 71
◯ 72
◯ 73
◯ 74
◯ 75
◯ 76
◯ 77
◯ 78
◯ 79
◯ 80
◯ 81
◯ 82
◯ 83
◯ 84
◯ 85
◯ 86
◯ 87
◯ 88
◯ 89
◯ 90
◯ 91
◯ 92
◯ 93
◯ 94
◯ 95
◯ 96
◯ 97
◯ 98
◯ 99
◯ Don't know

At what age were you first told you had this mood condition?

◯ 0
◯ 1
◯ 2
◯ 3
◯ 4
◯ 5
◯ 6
◯ 7
◯ 8
◯ 9
◯ 10
◯ 11
◯ 12
◯ 13
◯ 14
◯ 15
◯ 16
◯ 17
◯ 18
◯ 19
◯ 20
◯ 21
◯ 22
◯ 23
◯ 24
◯ 25
◯ 26
◯ 27
◯ 28
◯ 29
◯ 30
◯ 31
◯ 32
◯ 33
◯ 34
◯ 35
◯ 36
◯ 37
◯ 38
◯ 39
◯ 40
◯ 41
◯ 42
◯ 43
◯ 44
◯ 45
◯ 46
◯ 47
◯ 48
◯ 49
◯ 50
◯ 51
◯ 52
◯ 53
◯ 54
◯ 55
◯ 56
◯ 57
◯ 58
◯ 59
◯ 60
◯ 61
◯ 62
◯ 63
◯ 64
◯ 65
◯ 66
◯ 67
◯ 68

◯ 69
◯ 70
◯ 71
◯ 72
◯ 73
◯ 74
◯ 75
◯ 76
◯ 77
◯ 78
◯ 79
◯ 80
◯ 81
◯ 82
◯ 83
◯ 84
◯ 85
◯ 86
◯ 87
◯ 88
◯ 89
◯ 90
◯ 91
◯ 92
◯ 93
◯ 94
◯ 95
◯ 96
◯ 97
◯ 98
◯ 99
◯ Don't know

At what age were you first told you had anxiety (post-traumatic stress disorder, panic attacks, obsessive compulsive disorder, phobias)?

◯ 0
◯ 1
◯ 2
◯ 3
◯ 4
◯ 5
◯ 6
◯ 7
◯ 8
◯ 9
◯ 10
◯ 11
◯ 12
◯ 13
◯ 14
◯ 15
◯ 16
◯ 17
◯ 18
◯ 19
◯ 20
◯ 21
◯ 22
◯ 23
◯ 24
◯ 25
◯ 26
◯ 27
◯ 28
◯ 29
◯ 30
◯ 31
◯ 32
◯ 33
◯ 34
◯ 35
◯ 36
◯ 37
◯ 38
◯ 39
◯ 40
◯ 41
◯ 42
◯ 43
◯ 44
◯ 45
◯ 46
◯ 47
◯ 48
◯ 49
◯ 50
◯ 51
◯ 52
◯ 53
◯ 54
◯ 55
◯ 56
◯ 57
◯ 58
◯ 59
◯ 60
◯ 61
◯ 62
◯ 63
◯ 64
◯ 65
◯ 66
◯ 67
◯ 68

◯ 69
◯ 70
◯ 71
◯ 72
◯ 73
◯ 74
◯ 75
◯ 76
◯ 77
◯ 78
◯ 79
◯ 80
◯ 81
◯ 82
◯ 83
◯ 84
◯ 85
◯ 86
◯ 87
◯ 88
◯ 89
◯ 90
◯ 91
◯ 92
◯ 93
◯ 94
◯ 95
◯ 96
◯ 97
◯ 98
◯ 99
◯ Don't know

At what age were you first told you had this anxiety condition?

◯ 0
◯ 1
◯ 2
◯ 3
◯ 4
◯ 5
◯ 6
◯ 7
◯ 8
◯ 9
◯ 10
◯ 11
◯ 12
◯ 13
◯ 14
◯ 15
◯ 16
◯ 17
◯ 18
◯ 19
◯ 20
◯ 21
◯ 22
◯ 23
◯ 24
◯ 25
◯ 26
◯ 27
◯ 28
◯ 29
◯ 30
◯ 31
◯ 32
◯ 33
◯ 34
◯ 35
◯ 36
◯ 37
◯ 38
◯ 39
◯ 40
◯ 41
◯ 42
◯ 43
◯ 44
◯ 45
◯ 46
◯ 47
◯ 48
◯ 49
◯ 50
◯ 51
◯ 52
◯ 53
◯ 54
◯ 55
◯ 56
◯ 57
◯ 58
◯ 59
◯ 60
◯ 61
◯ 62
◯ 63
◯ 64
◯ 65
◯ 66
◯ 67
◯ 68

- ◯ 69
- ◯ 70
- ◯ 71
- ◯ 72
- ◯ 73
- ◯ 74
- ◯ 75
- ◯ 76
- ◯ 77
- ◯ 78
- ◯ 79
- ◯ 80
- ◯ 81
- ◯ 82
- ◯ 83
- ◯ 84
- ◯ 85
- ◯ 86
- ◯ 87
- ◯ 88
- ◯ 89
- ◯ 90
- ◯ 91
- ◯ 92
- ◯ 93
- ◯ 94
- ◯ 95
- ◯ 96
- ◯ 97
- ◯ 98
- ◯ 99
- ◯ Don't know

At what age were you first told you had Mild cognitive impairment (MCI)?

- ◯ 0
- ◯ 1
- ◯ 2
- ◯ 3
- ◯ 4
- ◯ 5
- ◯ 6
- ◯ 7
- ◯ 8
- ◯ 9
- ◯ 10
- ◯ 11
- ◯ 12
- ◯ 13
- ◯ 14
- ◯ 15
- ◯ 16
- ◯ 17
- ◯ 18
- ◯ 19
- ◯ 20
- ◯ 21
- ◯ 22
- ◯ 23
- ◯ 24
- ◯ 25
- ◯ 26
- ◯ 27
- ◯ 28
- ◯ 29
- ◯ 30
- ◯ 31
- ◯ 32
- ◯ 33
- ◯ 34
- ◯ 35
- ◯ 36
- ◯ 37
- ◯ 38
- ◯ 39
- ◯ 40
- ◯ 41
- ◯ 42
- ◯ 43
- ◯ 44
- ◯ 45
- ◯ 46
- ◯ 47
- ◯ 48
- ◯ 49
- ◯ 50
- ◯ 51
- ◯ 52
- ◯ 53
- ◯ 54
- ◯ 55
- ◯ 56
- ◯ 57
- ◯ 58
- ◯ 59
- ◯ 60
- ◯ 61
- ◯ 62
- ◯ 63
- ◯ 64
- ◯ 65
- ◯ 66
- ◯ 67
- ◯ 68

- ◯ 69
- ◯ 70
- ◯ 71
- ◯ 72
- ◯ 73
- ◯ 74
- ◯ 75
- ◯ 76
- ◯ 77
- ◯ 78
- ◯ 79
- ◯ 80
- ◯ 81
- ◯ 82
- ◯ 83
- ◯ 84
- ◯ 85
- ◯ 86
- ◯ 87
- ◯ 88
- ◯ 89
- ◯ 90
- ◯ 91
- ◯ 92
- ◯ 93
- ◯ 94
- ◯ 95
- ◯ 96
- ◯ 97
- ◯ 98
- ◯ 99
- ◯ Don't know

At what age were you first told you had dementia?

◯ 0
◯ 1
◯ 2
◯ 3
◯ 4
◯ 5
◯ 6
◯ 7
◯ 8
◯ 9
◯ 10
◯ 11
◯ 12
◯ 13
◯ 14
◯ 15
◯ 16
◯ 17
◯ 18
◯ 19
◯ 20
◯ 21
◯ 22
◯ 23
◯ 24
◯ 25
◯ 26
◯ 27
◯ 28
◯ 29
◯ 30
◯ 31
◯ 32
◯ 33
◯ 34
◯ 35
◯ 36
◯ 37
◯ 38
◯ 39
◯ 40
◯ 41
◯ 42
◯ 43
◯ 44
◯ 45
◯ 46
◯ 47
◯ 48
◯ 49
◯ 50
◯ 51
◯ 52
◯ 53
◯ 54
◯ 55
◯ 56
◯ 57
◯ 58
◯ 59
◯ 60
◯ 61
◯ 62
◯ 63
◯ 64
◯ 65
◯ 66
◯ 67
◯ 68

◯ 69
◯ 70
◯ 71
◯ 72
◯ 73
◯ 74
◯ 75
◯ 76
◯ 77
◯ 78
◯ 79
◯ 80
◯ 81
◯ 82
◯ 83
◯ 84
◯ 85
◯ 86
◯ 87
◯ 88
◯ 89
◯ 90
◯ 91
◯ 92
◯ 93
◯ 94
◯ 95
◯ 96
◯ 97
◯ 98
◯ 99
◯ Don't know

At what age were you first told you had Alzheimer's disease?

◯ 0
◯ 1
◯ 2
◯ 3
◯ 4
◯ 5
◯ 6
◯ 7
◯ 8
◯ 9
◯ 10
◯ 11
◯ 12
◯ 13
◯ 14
◯ 15
◯ 16
◯ 17
◯ 18
◯ 19
◯ 20
◯ 21
◯ 22
◯ 23
◯ 24
◯ 25
◯ 26
◯ 27
◯ 28
◯ 29
◯ 30
◯ 31
◯ 32
◯ 33
◯ 34
◯ 35
◯ 36
◯ 37
◯ 38
◯ 39
◯ 40
◯ 41
◯ 42
◯ 43
◯ 44
◯ 45
◯ 46
◯ 47
◯ 48
◯ 49
◯ 50
◯ 51
◯ 52
◯ 53
◯ 54
◯ 55
◯ 56
◯ 57
◯ 58
◯ 59
◯ 60
◯ 61
◯ 62
◯ 63
◯ 64
◯ 65
◯ 66
◯ 67
◯ 68

◯ 69
◯ 70
◯ 71
◯ 72
◯ 73
◯ 74
◯ 75
◯ 76
◯ 77
◯ 78
◯ 79
◯ 80
◯ 81
◯ 82
◯ 83
◯ 84
◯ 85
◯ 86
◯ 87
◯ 88
◯ 89
◯ 90
◯ 91
◯ 92
◯ 93
◯ 94
◯ 95
◯ 96
◯ 97
◯ 98
◯ 99
◯ Don't know

At what age were you first told you had [mental_others]?

◯ 0
◯ 1
◯ 2
◯ 3
◯ 4
◯ 5
◯ 6
◯ 7
◯ 8
◯ 9
◯ 10
◯ 11
◯ 12
◯ 13
◯ 14
◯ 15
◯ 16
◯ 17
◯ 18
◯ 19
◯ 20
◯ 21
◯ 22
◯ 23
◯ 24
◯ 25
◯ 26
◯ 27
◯ 28
◯ 29
◯ 30
◯ 31
◯ 32
◯ 33
◯ 34
◯ 35
◯ 36
◯ 37
◯ 38
◯ 39
◯ 40
◯ 41
◯ 42
◯ 43
◯ 44
◯ 45
◯ 46
◯ 47
◯ 48
◯ 49
◯ 50
◯ 51
◯ 52
◯ 53
◯ 54
◯ 55
◯ 56
◯ 57
◯ 58
◯ 59
◯ 60
◯ 61
◯ 62
◯ 63
◯ 64
◯ 65
◯ 66
◯ 67
◯ 68

- ◯ 69
- ◯ 70
- ◯ 71
- ◯ 72
- ◯ 73
- ◯ 74
- ◯ 75
- ◯ 76
- ◯ 77
- ◯ 78
- ◯ 79
- ◯ 80
- ◯ 81
- ◯ 82
- ◯ 83
- ◯ 84
- ◯ 85
- ◯ 86
- ◯ 87
- ◯ 88
- ◯ 89
- ◯ 90
- ◯ 91
- ◯ 92
- ◯ 93
- ◯ 94
- ◯ 95
- ◯ 96
- ◯ 97
- ◯ 98
- ◯ 99
- ◯ Don't know

At what age were you first told you [mental_others_2]?

- 0
- 1
- 2
- 3
- 4
- 5
- 6
- 7
- 8
- 9
- 10
- 11
- 12
- 13
- 14
- 15
- 16
- 17
- 18
- 19
- 20
- 21
- 22
- 23
- 24
- 25
- 26
- 27
- 28
- 29
- 30
- 31
- 32
- 33
- 34
- 35
- 36
- 37
- 38
- 39
- 40
- 41
- 42
- 43
- 44
- 45
- 46
- 47
- 48
- 49
- 50
- 51
- 52
- 53
- 54
- 55
- 56
- 57
- 58
- 59
- 60
- 61
- 62
- 63
- 64
- 65
- 66
- 67
- 68

- ◯ 69
- ◯ 70
- ◯ 71
- ◯ 72
- ◯ 73
- ◯ 74
- ◯ 75
- ◯ 76
- ◯ 77
- ◯ 78
- ◯ 79
- ◯ 80
- ◯ 81
- ◯ 82
- ◯ 83
- ◯ 84
- ◯ 85
- ◯ 86
- ◯ 87
- ◯ 88
- ◯ 89
- ◯ 90
- ◯ 91
- ◯ 92
- ◯ 93
- ◯ 94
- ◯ 95
- ◯ 96
- ◯ 97
- ◯ 98
- ◯ 99
- ◯ Don't know

At what age were you first told you [mental_others_3]?

◯ 0
◯ 1
◯ 2
◯ 3
◯ 4
◯ 5
◯ 6
◯ 7
◯ 8
◯ 9
◯ 10
◯ 11
◯ 12
◯ 13
◯ 14
◯ 15
◯ 16
◯ 17
◯ 18
◯ 19
◯ 20
◯ 21
◯ 22
◯ 23
◯ 24
◯ 25
◯ 26
◯ 27
◯ 28
◯ 29
◯ 30
◯ 31
◯ 32
◯ 33
◯ 34
◯ 35
◯ 36
◯ 37
◯ 38
◯ 39
◯ 40
◯ 41
◯ 42
◯ 43
◯ 44
◯ 45
◯ 46
◯ 47
◯ 48
◯ 49
◯ 50
◯ 51
◯ 52
◯ 53
◯ 54
◯ 55
◯ 56
◯ 57
◯ 58
◯ 59
◯ 60
◯ 61
◯ 62
◯ 63
◯ 64
◯ 65
◯ 66
◯ 67
◯ 68

- ◯ 69
- ◯ 70
- ◯ 71
- ◯ 72
- ◯ 73
- ◯ 74
- ◯ 75
- ◯ 76
- ◯ 77
- ◯ 78
- ◯ 79
- ◯ 80
- ◯ 81
- ◯ 82
- ◯ 83
- ◯ 84
- ◯ 85
- ◯ 86
- ◯ 87
- ◯ 88
- ◯ 89
- ◯ 90
- ◯ 91
- ◯ 92
- ◯ 93
- ◯ 94
- ◯ 95
- ◯ 96
- ◯ 97
- ◯ 98
- ◯ 99
- ◯ Don't know

At what age were you first told you had this condition?

◯ 0
◯ 1
◯ 2
◯ 3
◯ 4
◯ 5
◯ 6
◯ 7
◯ 8
◯ 9
◯ 10
◯ 11
◯ 12
◯ 13
◯ 14
◯ 15
◯ 16
◯ 17
◯ 18
◯ 19
◯ 20
◯ 21
◯ 22
◯ 23
◯ 24
◯ 25
◯ 26
◯ 27
◯ 28
◯ 29
◯ 30
◯ 31
◯ 32
◯ 33
◯ 34
◯ 35
◯ 36
◯ 37
◯ 38
◯ 39
◯ 40
◯ 41
◯ 42
◯ 43
◯ 44
◯ 45
◯ 46
◯ 47
◯ 48
◯ 49
◯ 50
◯ 51
◯ 52
◯ 53
◯ 54
◯ 55
◯ 56
◯ 57
◯ 58
◯ 59
◯ 60
◯ 61
◯ 62
◯ 63
◯ 64
◯ 65
◯ 66
◯ 67
◯ 68

	◯ 69<br>◯ 70<br>◯ 71<br>◯ 72<br>◯ 73<br>◯ 74<br>◯ 75<br>◯ 76<br>◯ 77<br>◯ 78<br>◯ 79<br>◯ 80<br>◯ 81<br>◯ 82<br>◯ 83<br>◯ 84<br>◯ 85<br>◯ 86<br>◯ 87<br>◯ 88<br>◯ 89<br>◯ 90<br>◯ 91<br>◯ 92<br>◯ 93<br>◯ 94<br>◯ 95<br>◯ 96<br>◯ 97<br>◯ 98<br>◯ 99<br>◯ Don't know
Do you still have depression (including post-natal depression)?	◯ Yes<br>◯ No<br>◯ Don't know
Do you still have this mood condition?	◯ Yes<br>◯ No<br>◯ Don't know
Do you still have anxiety (post-traumatic stress disorder, panic attacks, obsessive compulsive disorder, phobias)?	◯ Yes<br>◯ No<br>◯ Don't know
Do you still have this anxiety condition?	◯ Yes<br>◯ No<br>◯ Don't know
Do you still have Mild cognitive impairement (MCI)?	◯ Yes<br>◯ No<br>◯ Don't know
Do you still have dementia?	◯ Yes<br>◯ No<br>◯ Don't know
Do you still have Alzheimer's disease?	◯ Yes<br>◯ No<br>◯ Don't know

Do you still have [mental_others]?
◯ Yes
◯ No
◯ Don't know

Do you still have this condition?
◯ Yes
◯ No
◯ Don't know

Do you still have [mental_others_2]?
◯ Yes
◯ No
◯ Don't know

Do you still have [mental_others_3]?
◯ Yes
◯ No
◯ Don't know

How is depression (including post-natal depression) treated?
(Tick all that apply)

☐ no treatment ☐ diet ☐ exercise ☐ medication (please specify) ☐ natural/herbal therapy
☐ weight management ☐ supplements (please specify) ☐ other (please specify) ☐ Don't know

please specify the medication
____

please specify the supplements
____

please specify the treatment
____

How is this mood condition treated?
(Tick all that apply)

☐ no treatment ☐ diet ☐ exercise ☐ medication (please specify) ☐ natural/herbal therapy
☐ weight management ☐ supplements (please specify) ☐ other (please specify) ☐ Don't know

please specify the medication
____

please specify the supplements
____

please specify the treatment
____

How is anxiety condition (such as post-traumatic stress disorder, panic attacks, obsessive compulsive disorder, phobias) treated?
(Tick all that apply)

☐ no treatment ☐ diet ☐ exercise ☐ medication (please specify) ☐ natural/herbal therapy
☐ weight management ☐ supplements (please specify) ☐ other (please specify) ☐ Don't know

please specify the medication ______________________

please specify the supplements ______________________

please specify the treatment ______________________

How is this anxiety condition treated?
(Tick all that apply)

☐ no treatment ☐ diet ☐ exercise ☐ medication (please specify) ☐ natural/herbal therapy
☐ weight management ☐ supplements (please specify) ☐ other (please specify) ☐ Don't know

please specify the medication ______________________

please specify the supplements ______________________

please specify the treatment ______________________

How is mild cognitive impairment (MCI) treated?
(Tick all that apply)

☐ no treatment ☐ diet ☐ exercise ☐ medication (please specify) ☐ natural/herbal therapy
☐ weight management ☐ supplements (please specify) ☐ other (please specify) ☐ Don't know

please specify the medication ______________________

please specify the supplements ______________________

please specify the treatment ______________________

How is dimentia treated?
(Tick all that apply)

☐ no treatment ☐ diet ☐ exercise ☐ medication (please specify) ☐ natural/herbal therapy
☐ weight management ☐ supplements (please specify) ☐ other (please specify) ☐ Don't know

please specify the medication
____

please specify the supplements
____

please specify the treatment
____

How is Alzhiemer's Disease treated?
(Tick all that apply)

☐ no treatment ☐ diet ☐ exercise ☐ medication (please specify) ☐ natural/herbal therapy
☐ weight management ☐ supplements (please specify) ☐ other (please specify) ☐ Don't know

please specify the medication
____

please specify the supplements
____

please specify the treatment
____

How is [mental_others] treated?
(Tick all that apply)

☐ no treatment ☐ diet ☐ exercise ☐ medication (please specify) ☐ natural/herbal therapy
☐ weight management ☐ supplements (please specify) ☐ other (please specify) ☐ Don't know

please specify the medication
____

please specify the supplements
____

please specify the treatment
____

How is [mental_others_2] treated?
(Tick all that apply)

☐ no treatment ☐ diet ☐ exercise ☐ medication (please specify) ☐ natural/herbal therapy
☐ weight management ☐ supplements (please specify) ☐ other (please specify) ☐ Don't know

please specify the medication
____

please specify the supplements
____

please specify the treatment ____________________

How is [mental_others_2] treated?
(Tick all that apply)

☐ no treatment ☐ diet ☐ exercise ☐ medication (please specify) ☐ natural/herbal therapy
☐ weight management ☐ supplements (please specify) ☐ other (please specify) ☐ Don't know

please specify the medication ____________________

please specify the supplements ____________________

please specify the treatment ____________________

How is this condition treated?
(Tick all that apply)

☐ no treatment ☐ diet ☐ exercise ☐ medication (please specify) ☐ natural/herbal therapy
☐ weight management ☐ supplements (please specify) ☐ other (please specify) ☐ Don't know

please specify the medication ____________________

please specify the supplements ____________________

please specify the treatment ____________________

Have either of your parents, or any of brothers or sisters ever been told by a doctor (GP or specialist) or psychologist that they have any depression, an anxiety condition, or memory loss?

◯ Yes
◯ No
◯ Don't know

This question is about past or present eating disorders. Have you ever been told by a doctor (GP or specialist), dietitian, or psychologist that you have an eating disorder?

◯ Yes ◯ No ◯ Don't know
◯ Prefer not to say

Which condition/s have been confirmed by a doctor (GP or specialist), dietitian, or psychologist?
(Tick all that apply)

☐ Anorexia nervosa ☐ Binge eating disorder ☐ Bulemia nervosa ☐ Other (please specify)
☐ Don't Know

please specify the condition ____________________

Do you have another eating disorder condition need to be specified?

◯ Yes ◯ No

please specify the second condition

____

Do you have another eating disorder condition need to be specified?

◯ Yes ◯ No

please specify the third condition

____

At what age were you first told you had anorexia nervosa?

◯ 0
◯ 1
◯ 2
◯ 3
◯ 4
◯ 5
◯ 6
◯ 7
◯ 8
◯ 9
◯ 10
◯ 11
◯ 12
◯ 13
◯ 14
◯ 15
◯ 16
◯ 17
◯ 18
◯ 19
◯ 20
◯ 21
◯ 22
◯ 23
◯ 24
◯ 25
◯ 26
◯ 27
◯ 28
◯ 29
◯ 30
◯ 31
◯ 32
◯ 33
◯ 34
◯ 35
◯ 36
◯ 37
◯ 38
◯ 39
◯ 40
◯ 41
◯ 42
◯ 43
◯ 44
◯ 45
◯ 46
◯ 47
◯ 48
◯ 49
◯ 50
◯ 51
◯ 52
◯ 53
◯ 54
◯ 55
◯ 56
◯ 57
◯ 58
◯ 59
◯ 60
◯ 61
◯ 62
◯ 63
◯ 64
◯ 65
◯ 66
◯ 67
◯ 68

- ◯ 69
- ◯ 70
- ◯ 71
- ◯ 72
- ◯ 73
- ◯ 74
- ◯ 75
- ◯ 76
- ◯ 77
- ◯ 78
- ◯ 79
- ◯ 80
- ◯ 81
- ◯ 82
- ◯ 83
- ◯ 84
- ◯ 85
- ◯ 86
- ◯ 87
- ◯ 88
- ◯ 89
- ◯ 90
- ◯ 91
- ◯ 92
- ◯ 93
- ◯ 94
- ◯ 95
- ◯ 96
- ◯ 97
- ◯ 98
- ◯ 99
- ◯ Don't know

At what age were you first told you had bulemia nervosa?

- 0
- 1
- 2
- 3
- 4
- 5
- 6
- 7
- 8
- 9
- 10
- 11
- 12
- 13
- 14
- 15
- 16
- 17
- 18
- 19
- 20
- 21
- 22
- 23
- 24
- 25
- 26
- 27
- 28
- 29
- 30
- 31
- 32
- 33
- 34
- 35
- 36
- 37
- 38
- 39
- 40
- 41
- 42
- 43
- 44
- 45
- 46
- 47
- 48
- 49
- 50
- 51
- 52
- 53
- 54
- 55
- 56
- 57
- 58
- 59
- 60
- 61
- 62
- 63
- 64
- 65
- 66
- 67
- 68

- ◯ 69
- ◯ 70
- ◯ 71
- ◯ 72
- ◯ 73
- ◯ 74
- ◯ 75
- ◯ 76
- ◯ 77
- ◯ 78
- ◯ 79
- ◯ 80
- ◯ 81
- ◯ 82
- ◯ 83
- ◯ 84
- ◯ 85
- ◯ 86
- ◯ 87
- ◯ 88
- ◯ 89
- ◯ 90
- ◯ 91
- ◯ 92
- ◯ 93
- ◯ 94
- ◯ 95
- ◯ 96
- ◯ 97
- ◯ 98
- ◯ 99
- ◯ Don't know

At what age were you first told you had binge eating disorder?

◯ 0
◯ 1
◯ 2
◯ 3
◯ 4
◯ 5
◯ 6
◯ 7
◯ 8
◯ 9
◯ 10
◯ 11
◯ 12
◯ 13
◯ 14
◯ 15
◯ 16
◯ 17
◯ 18
◯ 19
◯ 20
◯ 21
◯ 22
◯ 23
◯ 24
◯ 25
◯ 26
◯ 27
◯ 28
◯ 29
◯ 30
◯ 31
◯ 32
◯ 33
◯ 34
◯ 35
◯ 36
◯ 37
◯ 38
◯ 39
◯ 40
◯ 41
◯ 42
◯ 43
◯ 44
◯ 45
◯ 46
◯ 47
◯ 48
◯ 49
◯ 50
◯ 51
◯ 52
◯ 53
◯ 54
◯ 55
◯ 56
◯ 57
◯ 58
◯ 59
◯ 60
◯ 61
◯ 62
◯ 63
◯ 64
◯ 65
◯ 66
◯ 67
◯ 68

- ◯ 69
- ◯ 70
- ◯ 71
- ◯ 72
- ◯ 73
- ◯ 74
- ◯ 75
- ◯ 76
- ◯ 77
- ◯ 78
- ◯ 79
- ◯ 80
- ◯ 81
- ◯ 82
- ◯ 83
- ◯ 84
- ◯ 85
- ◯ 86
- ◯ 87
- ◯ 88
- ◯ 89
- ◯ 90
- ◯ 91
- ◯ 92
- ◯ 93
- ◯ 94
- ◯ 95
- ◯ 96
- ◯ 97
- ◯ 98
- ◯ 99
- ◯ Don't know

At what age were you first told you had [ed_others]?

- ◯ 0
- ◯ 1
- ◯ 2
- ◯ 3
- ◯ 4
- ◯ 5
- ◯ 6
- ◯ 7
- ◯ 8
- ◯ 9
- ◯ 10
- ◯ 11
- ◯ 12
- ◯ 13
- ◯ 14
- ◯ 15
- ◯ 16
- ◯ 17
- ◯ 18
- ◯ 19
- ◯ 20
- ◯ 21
- ◯ 22
- ◯ 23
- ◯ 24
- ◯ 25
- ◯ 26
- ◯ 27
- ◯ 28
- ◯ 29
- ◯ 30
- ◯ 31
- ◯ 32
- ◯ 33
- ◯ 34
- ◯ 35
- ◯ 36
- ◯ 37
- ◯ 38
- ◯ 39
- ◯ 40
- ◯ 41
- ◯ 42
- ◯ 43
- ◯ 44
- ◯ 45
- ◯ 46
- ◯ 47
- ◯ 48
- ◯ 49
- ◯ 50
- ◯ 51
- ◯ 52
- ◯ 53
- ◯ 54
- ◯ 55
- ◯ 56
- ◯ 57
- ◯ 58
- ◯ 59
- ◯ 60
- ◯ 61
- ◯ 62
- ◯ 63
- ◯ 64
- ◯ 65
- ◯ 66
- ◯ 67
- ◯ 68

- ◯ 69
- ◯ 70
- ◯ 71
- ◯ 72
- ◯ 73
- ◯ 74
- ◯ 75
- ◯ 76
- ◯ 77
- ◯ 78
- ◯ 79
- ◯ 80
- ◯ 81
- ◯ 82
- ◯ 83
- ◯ 84
- ◯ 85
- ◯ 86
- ◯ 87
- ◯ 88
- ◯ 89
- ◯ 90
- ◯ 91
- ◯ 92
- ◯ 93
- ◯ 94
- ◯ 95
- ◯ 96
- ◯ 97
- ◯ 98
- ◯ 99
- ◯ Don't know

At what age were you first told you had [ed_others_2]?

◯ 0
◯ 1
◯ 2
◯ 3
◯ 4
◯ 5
◯ 6
◯ 7
◯ 8
◯ 9
◯ 10
◯ 11
◯ 12
◯ 13
◯ 14
◯ 15
◯ 16
◯ 17
◯ 18
◯ 19
◯ 20
◯ 21
◯ 22
◯ 23
◯ 24
◯ 25
◯ 26
◯ 27
◯ 28
◯ 29
◯ 30
◯ 31
◯ 32
◯ 33
◯ 34
◯ 35
◯ 36
◯ 37
◯ 38
◯ 39
◯ 40
◯ 41
◯ 42
◯ 43
◯ 44
◯ 45
◯ 46
◯ 47
◯ 48
◯ 49
◯ 50
◯ 51
◯ 52
◯ 53
◯ 54
◯ 55
◯ 56
◯ 57
◯ 58
◯ 59
◯ 60
◯ 61
◯ 62
◯ 63
◯ 64
◯ 65
◯ 66
◯ 67
◯ 68

◯ 69
◯ 70
◯ 71
◯ 72
◯ 73
◯ 74
◯ 75
◯ 76
◯ 77
◯ 78
◯ 79
◯ 80
◯ 81
◯ 82
◯ 83
◯ 84
◯ 85
◯ 86
◯ 87
◯ 88
◯ 89
◯ 90
◯ 91
◯ 92
◯ 93
◯ 94
◯ 95
◯ 96
◯ 97
◯ 98
◯ 99
◯ Don't know

At what age were you first told you had [ed_others_3]?

- ◯ 0
- ◯ 1
- ◯ 2
- ◯ 3
- ◯ 4
- ◯ 5
- ◯ 6
- ◯ 7
- ◯ 8
- ◯ 9
- ◯ 10
- ◯ 11
- ◯ 12
- ◯ 13
- ◯ 14
- ◯ 15
- ◯ 16
- ◯ 17
- ◯ 18
- ◯ 19
- ◯ 20
- ◯ 21
- ◯ 22
- ◯ 23
- ◯ 24
- ◯ 25
- ◯ 26
- ◯ 27
- ◯ 28
- ◯ 29
- ◯ 30
- ◯ 31
- ◯ 32
- ◯ 33
- ◯ 34
- ◯ 35
- ◯ 36
- ◯ 37
- ◯ 38
- ◯ 39
- ◯ 40
- ◯ 41
- ◯ 42
- ◯ 43
- ◯ 44
- ◯ 45
- ◯ 46
- ◯ 47
- ◯ 48
- ◯ 49
- ◯ 50
- ◯ 51
- ◯ 52
- ◯ 53
- ◯ 54
- ◯ 55
- ◯ 56
- ◯ 57
- ◯ 58
- ◯ 59
- ◯ 60
- ◯ 61
- ◯ 62
- ◯ 63
- ◯ 64
- ◯ 65
- ◯ 66
- ◯ 67
- ◯ 68

- ◯ 69
- ◯ 70
- ◯ 71
- ◯ 72
- ◯ 73
- ◯ 74
- ◯ 75
- ◯ 76
- ◯ 77
- ◯ 78
- ◯ 79
- ◯ 80
- ◯ 81
- ◯ 82
- ◯ 83
- ◯ 84
- ◯ 85
- ◯ 86
- ◯ 87
- ◯ 88
- ◯ 89
- ◯ 90
- ◯ 91
- ◯ 92
- ◯ 93
- ◯ 94
- ◯ 95
- ◯ 96
- ◯ 97
- ◯ 98
- ◯ 99
- ◯ Don't know

At what age were you first told you had this condition?

◯ 0
◯ 1
◯ 2
◯ 3
◯ 4
◯ 5
◯ 6
◯ 7
◯ 8
◯ 9
◯ 10
◯ 11
◯ 12
◯ 13
◯ 14
◯ 15
◯ 16
◯ 17
◯ 18
◯ 19
◯ 20
◯ 21
◯ 22
◯ 23
◯ 24
◯ 25
◯ 26
◯ 27
◯ 28
◯ 29
◯ 30
◯ 31
◯ 32
◯ 33
◯ 34
◯ 35
◯ 36
◯ 37
◯ 38
◯ 39
◯ 40
◯ 41
◯ 42
◯ 43
◯ 44
◯ 45
◯ 46
◯ 47
◯ 48
◯ 49
◯ 50
◯ 51
◯ 52
◯ 53
◯ 54
◯ 55
◯ 56
◯ 57
◯ 58
◯ 59
◯ 60
◯ 61
◯ 62
◯ 63
◯ 64
◯ 65
◯ 66
◯ 67
◯ 68

	◯ 69<br>◯ 70<br>◯ 71<br>◯ 72<br>◯ 73<br>◯ 74<br>◯ 75<br>◯ 76<br>◯ 77<br>◯ 78<br>◯ 79<br>◯ 80<br>◯ 81<br>◯ 82<br>◯ 83<br>◯ 84<br>◯ 85<br>◯ 86<br>◯ 87<br>◯ 88<br>◯ 89<br>◯ 90<br>◯ 91<br>◯ 92<br>◯ 93<br>◯ 94<br>◯ 95<br>◯ 96<br>◯ 97<br>◯ 98<br>◯ 99<br>◯ Don't know
Do you still have anorexia nervosa?	◯ Yes<br>◯ No<br>◯ Don't know
Do you still have bulemia nervosa?	◯ Yes<br>◯ No<br>◯ Don't know
Do you still have binge eating disorder?	◯ Yes<br>◯ No<br>◯ Don't know
Do you still have [ed_others]?	◯ Yes<br>◯ No<br>◯ Don't know
Do you still have this condition?	◯ Yes<br>◯ No<br>◯ Don't know
Do you still have [ed_others_2]?	◯ Yes<br>◯ No<br>◯ Don't know
Do you still have [ed_others_3]?	◯ Yes<br>◯ No<br>◯ Don't know

How is anorexia nervosa treated?
(Tick all that apply)

☐ no treatment
☐ medication (please specify)
☐ natural/herbal therapy
☐ supplements (please specify)
☐ specialist consultations (please specify)
☐ other (please specify)
☐ Don't Know

please specify the medication ______________________

please specify the supplements ______________________

please specify the specialist consultations ______________________

please specify the treatment ______________________

How is bulemia nervosa treated?
(Tick all that apply)

☐ no treatment
☐ medication (please specify)
☐ natural/herbal therapy
☐ supplements (please specify)
☐ specialist consultations (please specify)
☐ other (please specify)
☐ Don't Know

please specify the medication ______________________

please specify the supplements ______________________

please specify the specialist consultations ______________________

please specify the treatment ______________________

How is binge eating disorder treated?
(Tick all that apply)

☐ no treatment
☐ medication (please specify)
☐ natural/herbal therapy
☐ supplements (please specify)
☐ specialist consultations (please specify)
☐ other (please specify)
☐ Don't Know

please specify the medication ______________________

please specify the supplements

____

please specify the specialist consultations

____

please specify the treatment

____

How is [ed_others] treated?
(Tick all that apply)

- ☐ no treatment
- ☐ medication (please specify)
- ☐ natural/herbal therapy
- ☐ supplements (please specify)
- ☐ specialist consultations (please specify)
- ☐ other (please specify)
- ☐ Don't Know

please specify the medication

____

please specify the supplements

____

please specify the specialist consultations

____

please specify the treatment

____

How is [ed_others_2] treated?
(Tick all that apply)

- ☐ no treatment
- ☐ medication (please specify)
- ☐ natural/herbal therapy
- ☐ supplements (please specify)
- ☐ specialist consultations (please specify)
- ☐ other (please specify)
- ☐ Don't Know

please specify the medication

____

please specify the supplements

____

please specify the specialist consultations

____

please specify the treatment

____

How is [ed_others_3] treated?
(Tick all that apply)

- ☐ no treatment
- ☐ medication (please specify)
- ☐ natural/herbal therapy
- ☐ supplements (please specify)
- ☐ specialist consultations (please specify)
- ☐ other (please specify)
- ☐ Don't Know

please specify the medication ______________________________

please specify the supplements ______________________________

please specify the specialist consultations ______________________________

please specify the treatment ______________________________

How is this condition treated?
(Tick all that apply)

- ☐ no treatment
- ☐ medication (please specify)
- ☐ natural/herbal therapy
- ☐ supplements (please specify)
- ☐ specialist consultations (please specify)
- ☐ other (please specify)
- ☐ Don't Know

please specify the medication ______________________________

please specify the supplements ______________________________

please specify the specialist consultations ______________________________

please specify the treatment ______________________________

Have you ever been told by a doctor (GP or specialist) or nurse that you have asthma?

- ◯ Yes
- ◯ No
- ◯ Don't know

At what age were you first told you had asthma?

- ◯ 0
- ◯ 1
- ◯ 2
- ◯ 3
- ◯ 4
- ◯ 5
- ◯ 6
- ◯ 7
- ◯ 8
- ◯ 9
- ◯ 10
- ◯ 11
- ◯ 12
- ◯ 13
- ◯ 14
- ◯ 15
- ◯ 16
- ◯ 17
- ◯ 18
- ◯ 19
- ◯ 20
- ◯ 21
- ◯ 22
- ◯ 23
- ◯ 24
- ◯ 25
- ◯ 26
- ◯ 27
- ◯ 28
- ◯ 29
- ◯ 30
- ◯ 31
- ◯ 32
- ◯ 33
- ◯ 34
- ◯ 35
- ◯ 36
- ◯ 37
- ◯ 38
- ◯ 39
- ◯ 40
- ◯ 41
- ◯ 42
- ◯ 43
- ◯ 44
- ◯ 45
- ◯ 46
- ◯ 47
- ◯ 48
- ◯ 49
- ◯ 50
- ◯ 51
- ◯ 52
- ◯ 53
- ◯ 54
- ◯ 55
- ◯ 56
- ◯ 57
- ◯ 58
- ◯ 59
- ◯ 60
- ◯ 61
- ◯ 62
- ◯ 63
- ◯ 64
- ◯ 65
- ◯ 66
- ◯ 67
- ◯ 68

◯ 69
◯ 70
◯ 71
◯ 72
◯ 73
◯ 74
◯ 75
◯ 76
◯ 77
◯ 78
◯ 79
◯ 80
◯ 81
◯ 82
◯ 83
◯ 84
◯ 85
◯ 86
◯ 87
◯ 88
◯ 89
◯ 90
◯ 91
◯ 92
◯ 93
◯ 94
◯ 95
◯ 96
◯ 97
◯ 98
◯ 99
◯ Don't know

Do you still get asthma?

◯ Yes
◯ No
◯ Don't know

How is your asthma treated?
(Tick all that apply)

☐ no treatment ☐ diet ☐ exercise ☐ medication (please specify) ☐ natural/herbal therapy
☐ weight management ☐ supplements (please specify) ☐ other (please specify) ☐ Don't know

---

please specify the medication

____

---

please specify the supplements

____

---

please specify the treatment

____

Have either of your parents, or any of brothers or sisters ever been told by a doctor (GP or specialist), or nurse that they have asthma?

◯ Yes
◯ No
◯ Don't know

Do you currently have a food allergy or intolerance?

◯ Yes
◯ No
◯ Don't know

Which food allergy/allergies or intolerance/s have been confirmed by blood test, skin prick test, biopsy, or elimination diet prescribed by a dietitian?
(Tick all that apply)

☐ None have been confirmed
☐ amine intolerance
☐ cow's milk/dairy allergy
☐ cow's milk/dairy/lactose intolerance
☐ coeliac disease (confirmed by biopsy)
☐ colour/flavour/preservative (e.g. sulphite) intolerance
☐ egg allergy
☐ fish allergy
☐ FODMAPS intolerance (e.g. fructans, fructose)
☐ glutamate intolerance
☐ gluten/wheat sensitivity (non-coeliac disease)
☐ lupin allergy
☐ peanut allergy
☐ salicylate intolerance
☐ shellfish allergy
☐ soy allergy
☐ tree nut allergy (e.g. cashew, hazelnut, walnut)
☐ wheat allergy
☐ yeast allergy
☐ Other (please specify)
☐ Don't Know

---

please specify the allergy or intolerance

____

Do you have another food allergy or intolerance need to be specified?

◯ Yes ◯ No

---

please specify the second allergy or intolerance

____

Do you have another food allergy or intolerance need to be specified?

◯ Yes ◯ No

---

please specify the third allergy or intolerance

____

At what age were you first told you had egg allergy?

◯ 0
◯ 1
◯ 2
◯ 3
◯ 4
◯ 5
◯ 6
◯ 7
◯ 8
◯ 9
◯ 10
◯ 11
◯ 12
◯ 13
◯ 14
◯ 15
◯ 16
◯ 17
◯ 18
◯ 19
◯ 20
◯ 21
◯ 22
◯ 23
◯ 24
◯ 25
◯ 26
◯ 27
◯ 28
◯ 29
◯ 30
◯ 31
◯ 32
◯ 33
◯ 34
◯ 35
◯ 36
◯ 37
◯ 38
◯ 39
◯ 40
◯ 41
◯ 42
◯ 43
◯ 44
◯ 45
◯ 46
◯ 47
◯ 48
◯ 49
◯ 50
◯ 51
◯ 52
◯ 53
◯ 54
◯ 55
◯ 56
◯ 57
◯ 58
◯ 59
◯ 60
◯ 61
◯ 62
◯ 63
◯ 64
◯ 65
◯ 66
◯ 67
◯ 68

◯ 69
◯ 70
◯ 71
◯ 72
◯ 73
◯ 74
◯ 75
◯ 76
◯ 77
◯ 78
◯ 79
◯ 80
◯ 81
◯ 82
◯ 83
◯ 84
◯ 85
◯ 86
◯ 87
◯ 88
◯ 89
◯ 90
◯ 91
◯ 92
◯ 93
◯ 94
◯ 95
◯ 96
◯ 97
◯ 98
◯ 99
◯ Don't know

At what age were you first told you had cow's milk/dairy allergy?

- ◯ 0
- ◯ 1
- ◯ 2
- ◯ 3
- ◯ 4
- ◯ 5
- ◯ 6
- ◯ 7
- ◯ 8
- ◯ 9
- ◯ 10
- ◯ 11
- ◯ 12
- ◯ 13
- ◯ 14
- ◯ 15
- ◯ 16
- ◯ 17
- ◯ 18
- ◯ 19
- ◯ 20
- ◯ 21
- ◯ 22
- ◯ 23
- ◯ 24
- ◯ 25
- ◯ 26
- ◯ 27
- ◯ 28
- ◯ 29
- ◯ 30
- ◯ 31
- ◯ 32
- ◯ 33
- ◯ 34
- ◯ 35
- ◯ 36
- ◯ 37
- ◯ 38
- ◯ 39
- ◯ 40
- ◯ 41
- ◯ 42
- ◯ 43
- ◯ 44
- ◯ 45
- ◯ 46
- ◯ 47
- ◯ 48
- ◯ 49
- ◯ 50
- ◯ 51
- ◯ 52
- ◯ 53
- ◯ 54
- ◯ 55
- ◯ 56
- ◯ 57
- ◯ 58
- ◯ 59
- ◯ 60
- ◯ 61
- ◯ 62
- ◯ 63
- ◯ 64
- ◯ 65
- ◯ 66
- ◯ 67
- ◯ 68

- ◯ 69
- ◯ 70
- ◯ 71
- ◯ 72
- ◯ 73
- ◯ 74
- ◯ 75
- ◯ 76
- ◯ 77
- ◯ 78
- ◯ 79
- ◯ 80
- ◯ 81
- ◯ 82
- ◯ 83
- ◯ 84
- ◯ 85
- ◯ 86
- ◯ 87
- ◯ 88
- ◯ 89
- ◯ 90
- ◯ 91
- ◯ 92
- ◯ 93
- ◯ 94
- ◯ 95
- ◯ 96
- ◯ 97
- ◯ 98
- ◯ 99
- ◯ Don't know

At what age were you first told you had cow's milk/dairy/lactose intolerance?

◯ 0
◯ 1
◯ 2
◯ 3
◯ 4
◯ 5
◯ 6
◯ 7
◯ 8
◯ 9
◯ 10
◯ 11
◯ 12
◯ 13
◯ 14
◯ 15
◯ 16
◯ 17
◯ 18
◯ 19
◯ 20
◯ 21
◯ 22
◯ 23
◯ 24
◯ 25
◯ 26
◯ 27
◯ 28
◯ 29
◯ 30
◯ 31
◯ 32
◯ 33
◯ 34
◯ 35
◯ 36
◯ 37
◯ 38
◯ 39
◯ 40
◯ 41
◯ 42
◯ 43
◯ 44
◯ 45
◯ 46
◯ 47
◯ 48
◯ 49
◯ 50
◯ 51
◯ 52
◯ 53
◯ 54
◯ 55
◯ 56
◯ 57
◯ 58
◯ 59
◯ 60
◯ 61
◯ 62
◯ 63
◯ 64
◯ 65
◯ 66
◯ 67
◯ 68

- ◯ 69
- ◯ 70
- ◯ 71
- ◯ 72
- ◯ 73
- ◯ 74
- ◯ 75
- ◯ 76
- ◯ 77
- ◯ 78
- ◯ 79
- ◯ 80
- ◯ 81
- ◯ 82
- ◯ 83
- ◯ 84
- ◯ 85
- ◯ 86
- ◯ 87
- ◯ 88
- ◯ 89
- ◯ 90
- ◯ 91
- ◯ 92
- ◯ 93
- ◯ 94
- ◯ 95
- ◯ 96
- ◯ 97
- ◯ 98
- ◯ 99
- ◯ Don't know

At what age were you first told you had peanut allergy?

◯ 0
◯ 1
◯ 2
◯ 3
◯ 4
◯ 5
◯ 6
◯ 7
◯ 8
◯ 9
◯ 10
◯ 11
◯ 12
◯ 13
◯ 14
◯ 15
◯ 16
◯ 17
◯ 18
◯ 19
◯ 20
◯ 21
◯ 22
◯ 23
◯ 24
◯ 25
◯ 26
◯ 27
◯ 28
◯ 29
◯ 30
◯ 31
◯ 32
◯ 33
◯ 34
◯ 35
◯ 36
◯ 37
◯ 38
◯ 39
◯ 40
◯ 41
◯ 42
◯ 43
◯ 44
◯ 45
◯ 46
◯ 47
◯ 48
◯ 49
◯ 50
◯ 51
◯ 52
◯ 53
◯ 54
◯ 55
◯ 56
◯ 57
◯ 58
◯ 59
◯ 60
◯ 61
◯ 62
◯ 63
◯ 64
◯ 65
◯ 66
◯ 67
◯ 68

- ◯ 69
- ◯ 70
- ◯ 71
- ◯ 72
- ◯ 73
- ◯ 74
- ◯ 75
- ◯ 76
- ◯ 77
- ◯ 78
- ◯ 79
- ◯ 80
- ◯ 81
- ◯ 82
- ◯ 83
- ◯ 84
- ◯ 85
- ◯ 86
- ◯ 87
- ◯ 88
- ◯ 89
- ◯ 90
- ◯ 91
- ◯ 92
- ◯ 93
- ◯ 94
- ◯ 95
- ◯ 96
- ◯ 97
- ◯ 98
- ◯ 99
- ◯ Don't know

At what age were you first told you had tree nut allergy (e.g. cashew, hazelnut, walnut)?

- ◯ 0
- ◯ 1
- ◯ 2
- ◯ 3
- ◯ 4
- ◯ 5
- ◯ 6
- ◯ 7
- ◯ 8
- ◯ 9
- ◯ 10
- ◯ 11
- ◯ 12
- ◯ 13
- ◯ 14
- ◯ 15
- ◯ 16
- ◯ 17
- ◯ 18
- ◯ 19
- ◯ 20
- ◯ 21
- ◯ 22
- ◯ 23
- ◯ 24
- ◯ 25
- ◯ 26
- ◯ 27
- ◯ 28
- ◯ 29
- ◯ 30
- ◯ 31
- ◯ 32
- ◯ 33
- ◯ 34
- ◯ 35
- ◯ 36
- ◯ 37
- ◯ 38
- ◯ 39
- ◯ 40
- ◯ 41
- ◯ 42
- ◯ 43
- ◯ 44
- ◯ 45
- ◯ 46
- ◯ 47
- ◯ 48
- ◯ 49
- ◯ 50
- ◯ 51
- ◯ 52
- ◯ 53
- ◯ 54
- ◯ 55
- ◯ 56
- ◯ 57
- ◯ 58
- ◯ 59
- ◯ 60
- ◯ 61
- ◯ 62
- ◯ 63
- ◯ 64
- ◯ 65
- ◯ 66
- ◯ 67
- ◯ 68

◯ 69
◯ 70
◯ 71
◯ 72
◯ 73
◯ 74
◯ 75
◯ 76
◯ 77
◯ 78
◯ 79
◯ 80
◯ 81
◯ 82
◯ 83
◯ 84
◯ 85
◯ 86
◯ 87
◯ 88
◯ 89
◯ 90
◯ 91
◯ 92
◯ 93
◯ 94
◯ 95
◯ 96
◯ 97
◯ 98
◯ 99
◯ Don't know

At what age were you first told you had fish allergy?

- ◯ 0
- ◯ 1
- ◯ 2
- ◯ 3
- ◯ 4
- ◯ 5
- ◯ 6
- ◯ 7
- ◯ 8
- ◯ 9
- ◯ 10
- ◯ 11
- ◯ 12
- ◯ 13
- ◯ 14
- ◯ 15
- ◯ 16
- ◯ 17
- ◯ 18
- ◯ 19
- ◯ 20
- ◯ 21
- ◯ 22
- ◯ 23
- ◯ 24
- ◯ 25
- ◯ 26
- ◯ 27
- ◯ 28
- ◯ 29
- ◯ 30
- ◯ 31
- ◯ 32
- ◯ 33
- ◯ 34
- ◯ 35
- ◯ 36
- ◯ 37
- ◯ 38
- ◯ 39
- ◯ 40
- ◯ 41
- ◯ 42
- ◯ 43
- ◯ 44
- ◯ 45
- ◯ 46
- ◯ 47
- ◯ 48
- ◯ 49
- ◯ 50
- ◯ 51
- ◯ 52
- ◯ 53
- ◯ 54
- ◯ 55
- ◯ 56
- ◯ 57
- ◯ 58
- ◯ 59
- ◯ 60
- ◯ 61
- ◯ 62
- ◯ 63
- ◯ 64
- ◯ 65
- ◯ 66
- ◯ 67
- ◯ 68

- ◯ 69
- ◯ 70
- ◯ 71
- ◯ 72
- ◯ 73
- ◯ 74
- ◯ 75
- ◯ 76
- ◯ 77
- ◯ 78
- ◯ 79
- ◯ 80
- ◯ 81
- ◯ 82
- ◯ 83
- ◯ 84
- ◯ 85
- ◯ 86
- ◯ 87
- ◯ 88
- ◯ 89
- ◯ 90
- ◯ 91
- ◯ 92
- ◯ 93
- ◯ 94
- ◯ 95
- ◯ 96
- ◯ 97
- ◯ 98
- ◯ 99
- ◯ Don't know

At what age were you first told you had shellfish allergy?

- ◯ 0
- ◯ 1
- ◯ 2
- ◯ 3
- ◯ 4
- ◯ 5
- ◯ 6
- ◯ 7
- ◯ 8
- ◯ 9
- ◯ 10
- ◯ 11
- ◯ 12
- ◯ 13
- ◯ 14
- ◯ 15
- ◯ 16
- ◯ 17
- ◯ 18
- ◯ 19
- ◯ 20
- ◯ 21
- ◯ 22
- ◯ 23
- ◯ 24
- ◯ 25
- ◯ 26
- ◯ 27
- ◯ 28
- ◯ 29
- ◯ 30
- ◯ 31
- ◯ 32
- ◯ 33
- ◯ 34
- ◯ 35
- ◯ 36
- ◯ 37
- ◯ 38
- ◯ 39
- ◯ 40
- ◯ 41
- ◯ 42
- ◯ 43
- ◯ 44
- ◯ 45
- ◯ 46
- ◯ 47
- ◯ 48
- ◯ 49
- ◯ 50
- ◯ 51
- ◯ 52
- ◯ 53
- ◯ 54
- ◯ 55
- ◯ 56
- ◯ 57
- ◯ 58
- ◯ 59
- ◯ 60
- ◯ 61
- ◯ 62
- ◯ 63
- ◯ 64
- ◯ 65
- ◯ 66
- ◯ 67
- ◯ 68

- ◯ 69
- ◯ 70
- ◯ 71
- ◯ 72
- ◯ 73
- ◯ 74
- ◯ 75
- ◯ 76
- ◯ 77
- ◯ 78
- ◯ 79
- ◯ 80
- ◯ 81
- ◯ 82
- ◯ 83
- ◯ 84
- ◯ 85
- ◯ 86
- ◯ 87
- ◯ 88
- ◯ 89
- ◯ 90
- ◯ 91
- ◯ 92
- ◯ 93
- ◯ 94
- ◯ 95
- ◯ 96
- ◯ 97
- ◯ 98
- ◯ 99
- ◯ Don't know

At what age were you first told you had soy allergy?

◯ 0
◯ 1
◯ 2
◯ 3
◯ 4
◯ 5
◯ 6
◯ 7
◯ 8
◯ 9
◯ 10
◯ 11
◯ 12
◯ 13
◯ 14
◯ 15
◯ 16
◯ 17
◯ 18
◯ 19
◯ 20
◯ 21
◯ 22
◯ 23
◯ 24
◯ 25
◯ 26
◯ 27
◯ 28
◯ 29
◯ 30
◯ 31
◯ 32
◯ 33
◯ 34
◯ 35
◯ 36
◯ 37
◯ 38
◯ 39
◯ 40
◯ 41
◯ 42
◯ 43
◯ 44
◯ 45
◯ 46
◯ 47
◯ 48
◯ 49
◯ 50
◯ 51
◯ 52
◯ 53
◯ 54
◯ 55
◯ 56
◯ 57
◯ 58
◯ 59
◯ 60
◯ 61
◯ 62
◯ 63
◯ 64
◯ 65
◯ 66
◯ 67
◯ 68

- ◯ 69
- ◯ 70
- ◯ 71
- ◯ 72
- ◯ 73
- ◯ 74
- ◯ 75
- ◯ 76
- ◯ 77
- ◯ 78
- ◯ 79
- ◯ 80
- ◯ 81
- ◯ 82
- ◯ 83
- ◯ 84
- ◯ 85
- ◯ 86
- ◯ 87
- ◯ 88
- ◯ 89
- ◯ 90
- ◯ 91
- ◯ 92
- ◯ 93
- ◯ 94
- ◯ 95
- ◯ 96
- ◯ 97
- ◯ 98
- ◯ 99
- ◯ Don't know

At what age were you first told you had lupin allergy?

◯ 0
◯ 1
◯ 2
◯ 3
◯ 4
◯ 5
◯ 6
◯ 7
◯ 8
◯ 9
◯ 10
◯ 11
◯ 12
◯ 13
◯ 14
◯ 15
◯ 16
◯ 17
◯ 18
◯ 19
◯ 20
◯ 21
◯ 22
◯ 23
◯ 24
◯ 25
◯ 26
◯ 27
◯ 28
◯ 29
◯ 30
◯ 31
◯ 32
◯ 33
◯ 34
◯ 35
◯ 36
◯ 37
◯ 38
◯ 39
◯ 40
◯ 41
◯ 42
◯ 43
◯ 44
◯ 45
◯ 46
◯ 47
◯ 48
◯ 49
◯ 50
◯ 51
◯ 52
◯ 53
◯ 54
◯ 55
◯ 56
◯ 57
◯ 58
◯ 59
◯ 60
◯ 61
◯ 62
◯ 63
◯ 64
◯ 65
◯ 66
◯ 67
◯ 68

◯ 69
◯ 70
◯ 71
◯ 72
◯ 73
◯ 74
◯ 75
◯ 76
◯ 77
◯ 78
◯ 79
◯ 80
◯ 81
◯ 82
◯ 83
◯ 84
◯ 85
◯ 86
◯ 87
◯ 88
◯ 89
◯ 90
◯ 91
◯ 92
◯ 93
◯ 94
◯ 95
◯ 96
◯ 97
◯ 98
◯ 99
◯ Don't know

At what age were you first told you had coeliac disease (confirmed by biopsy)?

- ◯ 0
- ◯ 1
- ◯ 2
- ◯ 3
- ◯ 4
- ◯ 5
- ◯ 6
- ◯ 7
- ◯ 8
- ◯ 9
- ◯ 10
- ◯ 11
- ◯ 12
- ◯ 13
- ◯ 14
- ◯ 15
- ◯ 16
- ◯ 17
- ◯ 18
- ◯ 19
- ◯ 20
- ◯ 21
- ◯ 22
- ◯ 23
- ◯ 24
- ◯ 25
- ◯ 26
- ◯ 27
- ◯ 28
- ◯ 29
- ◯ 30
- ◯ 31
- ◯ 32
- ◯ 33
- ◯ 34
- ◯ 35
- ◯ 36
- ◯ 37
- ◯ 38
- ◯ 39
- ◯ 40
- ◯ 41
- ◯ 42
- ◯ 43
- ◯ 44
- ◯ 45
- ◯ 46
- ◯ 47
- ◯ 48
- ◯ 49
- ◯ 50
- ◯ 51
- ◯ 52
- ◯ 53
- ◯ 54
- ◯ 55
- ◯ 56
- ◯ 57
- ◯ 58
- ◯ 59
- ◯ 60
- ◯ 61
- ◯ 62
- ◯ 63
- ◯ 64
- ◯ 65
- ◯ 66
- ◯ 67
- ◯ 68

- ◯ 69
- ◯ 70
- ◯ 71
- ◯ 72
- ◯ 73
- ◯ 74
- ◯ 75
- ◯ 76
- ◯ 77
- ◯ 78
- ◯ 79
- ◯ 80
- ◯ 81
- ◯ 82
- ◯ 83
- ◯ 84
- ◯ 85
- ◯ 86
- ◯ 87
- ◯ 88
- ◯ 89
- ◯ 90
- ◯ 91
- ◯ 92
- ◯ 93
- ◯ 94
- ◯ 95
- ◯ 96
- ◯ 97
- ◯ 98
- ◯ 99
- ◯ Don't know

At what age were you first told you had gluten/wheat sensitivity (non-coeliac disease)?

◯ 0
◯ 1
◯ 2
◯ 3
◯ 4
◯ 5
◯ 6
◯ 7
◯ 8
◯ 9
◯ 10
◯ 11
◯ 12
◯ 13
◯ 14
◯ 15
◯ 16
◯ 17
◯ 18
◯ 19
◯ 20
◯ 21
◯ 22
◯ 23
◯ 24
◯ 25
◯ 26
◯ 27
◯ 28
◯ 29
◯ 30
◯ 31
◯ 32
◯ 33
◯ 34
◯ 35
◯ 36
◯ 37
◯ 38
◯ 39
◯ 40
◯ 41
◯ 42
◯ 43
◯ 44
◯ 45
◯ 46
◯ 47
◯ 48
◯ 49
◯ 50
◯ 51
◯ 52
◯ 53
◯ 54
◯ 55
◯ 56
◯ 57
◯ 58
◯ 59
◯ 60
◯ 61
◯ 62
◯ 63
◯ 64
◯ 65
◯ 66
◯ 67
◯ 68

○ 69
○ 70
○ 71
○ 72
○ 73
○ 74
○ 75
○ 76
○ 77
○ 78
○ 79
○ 80
○ 81
○ 82
○ 83
○ 84
○ 85
○ 86
○ 87
○ 88
○ 89
○ 90
○ 91
○ 92
○ 93
○ 94
○ 95
○ 96
○ 97
○ 98
○ 99
○ Don't know

At what age were you first told you had wheat allergy?

- ◯ 0
- ◯ 1
- ◯ 2
- ◯ 3
- ◯ 4
- ◯ 5
- ◯ 6
- ◯ 7
- ◯ 8
- ◯ 9
- ◯ 10
- ◯ 11
- ◯ 12
- ◯ 13
- ◯ 14
- ◯ 15
- ◯ 16
- ◯ 17
- ◯ 18
- ◯ 19
- ◯ 20
- ◯ 21
- ◯ 22
- ◯ 23
- ◯ 24
- ◯ 25
- ◯ 26
- ◯ 27
- ◯ 28
- ◯ 29
- ◯ 30
- ◯ 31
- ◯ 32
- ◯ 33
- ◯ 34
- ◯ 35
- ◯ 36
- ◯ 37
- ◯ 38
- ◯ 39
- ◯ 40
- ◯ 41
- ◯ 42
- ◯ 43
- ◯ 44
- ◯ 45
- ◯ 46
- ◯ 47
- ◯ 48
- ◯ 49
- ◯ 50
- ◯ 51
- ◯ 52
- ◯ 53
- ◯ 54
- ◯ 55
- ◯ 56
- ◯ 57
- ◯ 58
- ◯ 59
- ◯ 60
- ◯ 61
- ◯ 62
- ◯ 63
- ◯ 64
- ◯ 65
- ◯ 66
- ◯ 67
- ◯ 68

- ◯ 69
- ◯ 70
- ◯ 71
- ◯ 72
- ◯ 73
- ◯ 74
- ◯ 75
- ◯ 76
- ◯ 77
- ◯ 78
- ◯ 79
- ◯ 80
- ◯ 81
- ◯ 82
- ◯ 83
- ◯ 84
- ◯ 85
- ◯ 86
- ◯ 87
- ◯ 88
- ◯ 89
- ◯ 90
- ◯ 91
- ◯ 92
- ◯ 93
- ◯ 94
- ◯ 95
- ◯ 96
- ◯ 97
- ◯ 98
- ◯ 99
- ◯ Don't know

At what age were you first told you had yeast allergy?

- ◯ 0
- ◯ 1
- ◯ 2
- ◯ 3
- ◯ 4
- ◯ 5
- ◯ 6
- ◯ 7
- ◯ 8
- ◯ 9
- ◯ 10
- ◯ 11
- ◯ 12
- ◯ 13
- ◯ 14
- ◯ 15
- ◯ 16
- ◯ 17
- ◯ 18
- ◯ 19
- ◯ 20
- ◯ 21
- ◯ 22
- ◯ 23
- ◯ 24
- ◯ 25
- ◯ 26
- ◯ 27
- ◯ 28
- ◯ 29
- ◯ 30
- ◯ 31
- ◯ 32
- ◯ 33
- ◯ 34
- ◯ 35
- ◯ 36
- ◯ 37
- ◯ 38
- ◯ 39
- ◯ 40
- ◯ 41
- ◯ 42
- ◯ 43
- ◯ 44
- ◯ 45
- ◯ 46
- ◯ 47
- ◯ 48
- ◯ 49
- ◯ 50
- ◯ 51
- ◯ 52
- ◯ 53
- ◯ 54
- ◯ 55
- ◯ 56
- ◯ 57
- ◯ 58
- ◯ 59
- ◯ 60
- ◯ 61
- ◯ 62
- ◯ 63
- ◯ 64
- ◯ 65
- ◯ 66
- ◯ 67
- ◯ 68

- ◯ 69
- ◯ 70
- ◯ 71
- ◯ 72
- ◯ 73
- ◯ 74
- ◯ 75
- ◯ 76
- ◯ 77
- ◯ 78
- ◯ 79
- ◯ 80
- ◯ 81
- ◯ 82
- ◯ 83
- ◯ 84
- ◯ 85
- ◯ 86
- ◯ 87
- ◯ 88
- ◯ 89
- ◯ 90
- ◯ 91
- ◯ 92
- ◯ 93
- ◯ 94
- ◯ 95
- ◯ 96
- ◯ 97
- ◯ 98
- ◯ 99
- ◯ Don't know

At what age were you first told you had salicylate intolerance?

- ◯ 0
- ◯ 1
- ◯ 2
- ◯ 3
- ◯ 4
- ◯ 5
- ◯ 6
- ◯ 7
- ◯ 8
- ◯ 9
- ◯ 10
- ◯ 11
- ◯ 12
- ◯ 13
- ◯ 14
- ◯ 15
- ◯ 16
- ◯ 17
- ◯ 18
- ◯ 19
- ◯ 20
- ◯ 21
- ◯ 22
- ◯ 23
- ◯ 24
- ◯ 25
- ◯ 26
- ◯ 27
- ◯ 28
- ◯ 29
- ◯ 30
- ◯ 31
- ◯ 32
- ◯ 33
- ◯ 34
- ◯ 35
- ◯ 36
- ◯ 37
- ◯ 38
- ◯ 39
- ◯ 40
- ◯ 41
- ◯ 42
- ◯ 43
- ◯ 44
- ◯ 45
- ◯ 46
- ◯ 47
- ◯ 48
- ◯ 49
- ◯ 50
- ◯ 51
- ◯ 52
- ◯ 53
- ◯ 54
- ◯ 55
- ◯ 56
- ◯ 57
- ◯ 58
- ◯ 59
- ◯ 60
- ◯ 61
- ◯ 62
- ◯ 63
- ◯ 64
- ◯ 65
- ◯ 66
- ◯ 67
- ◯ 68

◯ 69
◯ 70
◯ 71
◯ 72
◯ 73
◯ 74
◯ 75
◯ 76
◯ 77
◯ 78
◯ 79
◯ 80
◯ 81
◯ 82
◯ 83
◯ 84
◯ 85
◯ 86
◯ 87
◯ 88
◯ 89
◯ 90
◯ 91
◯ 92
◯ 93
◯ 94
◯ 95
◯ 96
◯ 97
◯ 98
◯ 99
◯ Don't know

At what age were you first told you had amine intolerance?

◯ 0
◯ 1
◯ 2
◯ 3
◯ 4
◯ 5
◯ 6
◯ 7
◯ 8
◯ 9
◯ 10
◯ 11
◯ 12
◯ 13
◯ 14
◯ 15
◯ 16
◯ 17
◯ 18
◯ 19
◯ 20
◯ 21
◯ 22
◯ 23
◯ 24
◯ 25
◯ 26
◯ 27
◯ 28
◯ 29
◯ 30
◯ 31
◯ 32
◯ 33
◯ 34
◯ 35
◯ 36
◯ 37
◯ 38
◯ 39
◯ 40
◯ 41
◯ 42
◯ 43
◯ 44
◯ 45
◯ 46
◯ 47
◯ 48
◯ 49
◯ 50
◯ 51
◯ 52
◯ 53
◯ 54
◯ 55
◯ 56
◯ 57
◯ 58
◯ 59
◯ 60
◯ 61
◯ 62
◯ 63
◯ 64
◯ 65
◯ 66
◯ 67
◯ 68

◯ 69
◯ 70
◯ 71
◯ 72
◯ 73
◯ 74
◯ 75
◯ 76
◯ 77
◯ 78
◯ 79
◯ 80
◯ 81
◯ 82
◯ 83
◯ 84
◯ 85
◯ 86
◯ 87
◯ 88
◯ 89
◯ 90
◯ 91
◯ 92
◯ 93
◯ 94
◯ 95
◯ 96
◯ 97
◯ 98
◯ 99
◯ Don't know

At what age were you first told you had glutamate intolerance?

〇 0
〇 1
〇 2
〇 3
〇 4
〇 5
〇 6
〇 7
〇 8
〇 9
〇 10
〇 11
〇 12
〇 13
〇 14
〇 15
〇 16
〇 17
〇 18
〇 19
〇 20
〇 21
〇 22
〇 23
〇 24
〇 25
〇 26
〇 27
〇 28
〇 29
〇 30
〇 31
〇 32
〇 33
〇 34
〇 35
〇 36
〇 37
〇 38
〇 39
〇 40
〇 41
〇 42
〇 43
〇 44
〇 45
〇 46
〇 47
〇 48
〇 49
〇 50
〇 51
〇 52
〇 53
〇 54
〇 55
〇 56
〇 57
〇 58
〇 59
〇 60
〇 61
〇 62
〇 63
〇 64
〇 65
〇 66
〇 67
〇 68

- ◯ 69
- ◯ 70
- ◯ 71
- ◯ 72
- ◯ 73
- ◯ 74
- ◯ 75
- ◯ 76
- ◯ 77
- ◯ 78
- ◯ 79
- ◯ 80
- ◯ 81
- ◯ 82
- ◯ 83
- ◯ 84
- ◯ 85
- ◯ 86
- ◯ 87
- ◯ 88
- ◯ 89
- ◯ 90
- ◯ 91
- ◯ 92
- ◯ 93
- ◯ 94
- ◯ 95
- ◯ 96
- ◯ 97
- ◯ 98
- ◯ 99
- ◯ Don't know

At what age were you first told you had colour/flavour/preservative (e.g. sulphite) intolerance?

- ◯ 0
- ◯ 1
- ◯ 2
- ◯ 3
- ◯ 4
- ◯ 5
- ◯ 6
- ◯ 7
- ◯ 8
- ◯ 9
- ◯ 10
- ◯ 11
- ◯ 12
- ◯ 13
- ◯ 14
- ◯ 15
- ◯ 16
- ◯ 17
- ◯ 18
- ◯ 19
- ◯ 20
- ◯ 21
- ◯ 22
- ◯ 23
- ◯ 24
- ◯ 25
- ◯ 26
- ◯ 27
- ◯ 28
- ◯ 29
- ◯ 30
- ◯ 31
- ◯ 32
- ◯ 33
- ◯ 34
- ◯ 35
- ◯ 36
- ◯ 37
- ◯ 38
- ◯ 39
- ◯ 40
- ◯ 41
- ◯ 42
- ◯ 43
- ◯ 44
- ◯ 45
- ◯ 46
- ◯ 47
- ◯ 48
- ◯ 49
- ◯ 50
- ◯ 51
- ◯ 52
- ◯ 53
- ◯ 54
- ◯ 55
- ◯ 56
- ◯ 57
- ◯ 58
- ◯ 59
- ◯ 60
- ◯ 61
- ◯ 62
- ◯ 63
- ◯ 64
- ◯ 65
- ◯ 66
- ◯ 67
- ◯ 68

- ◯ 69
- ◯ 70
- ◯ 71
- ◯ 72
- ◯ 73
- ◯ 74
- ◯ 75
- ◯ 76
- ◯ 77
- ◯ 78
- ◯ 79
- ◯ 80
- ◯ 81
- ◯ 82
- ◯ 83
- ◯ 84
- ◯ 85
- ◯ 86
- ◯ 87
- ◯ 88
- ◯ 89
- ◯ 90
- ◯ 91
- ◯ 92
- ◯ 93
- ◯ 94
- ◯ 95
- ◯ 96
- ◯ 97
- ◯ 98
- ◯ 99
- ◯ Don't know

At what age were you first told you had FODMAPS intolerance (e.g. fructans, fructose)?

○ 0
○ 1
○ 2
○ 3
○ 4
○ 5
○ 6
○ 7
○ 8
○ 9
○ 10
○ 11
○ 12
○ 13
○ 14
○ 15
○ 16
○ 17
○ 18
○ 19
○ 20
○ 21
○ 22
○ 23
○ 24
○ 25
○ 26
○ 27
○ 28
○ 29
○ 30
○ 31
○ 32
○ 33
○ 34
○ 35
○ 36
○ 37
○ 38
○ 39
○ 40
○ 41
○ 42
○ 43
○ 44
○ 45
○ 46
○ 47
○ 48
○ 49
○ 50
○ 51
○ 52
○ 53
○ 54
○ 55
○ 56
○ 57
○ 58
○ 59
○ 60
○ 61
○ 62
○ 63
○ 64
○ 65
○ 66
○ 67
○ 68

- ◯ 69
- ◯ 70
- ◯ 71
- ◯ 72
- ◯ 73
- ◯ 74
- ◯ 75
- ◯ 76
- ◯ 77
- ◯ 78
- ◯ 79
- ◯ 80
- ◯ 81
- ◯ 82
- ◯ 83
- ◯ 84
- ◯ 85
- ◯ 86
- ◯ 87
- ◯ 88
- ◯ 89
- ◯ 90
- ◯ 91
- ◯ 92
- ◯ 93
- ◯ 94
- ◯ 95
- ◯ 96
- ◯ 97
- ◯ 98
- ◯ 99
- ◯ Don't know

At what age were you first told you had [allergy_others]?

◯ 0
◯ 1
◯ 2
◯ 3
◯ 4
◯ 5
◯ 6
◯ 7
◯ 8
◯ 9
◯ 10
◯ 11
◯ 12
◯ 13
◯ 14
◯ 15
◯ 16
◯ 17
◯ 18
◯ 19
◯ 20
◯ 21
◯ 22
◯ 23
◯ 24
◯ 25
◯ 26
◯ 27
◯ 28
◯ 29
◯ 30
◯ 31
◯ 32
◯ 33
◯ 34
◯ 35
◯ 36
◯ 37
◯ 38
◯ 39
◯ 40
◯ 41
◯ 42
◯ 43
◯ 44
◯ 45
◯ 46
◯ 47
◯ 48
◯ 49
◯ 50
◯ 51
◯ 52
◯ 53
◯ 54
◯ 55
◯ 56
◯ 57
◯ 58
◯ 59
◯ 60
◯ 61
◯ 62
◯ 63
◯ 64
◯ 65
◯ 66
◯ 67
◯ 68

- ◯ 69
- ◯ 70
- ◯ 71
- ◯ 72
- ◯ 73
- ◯ 74
- ◯ 75
- ◯ 76
- ◯ 77
- ◯ 78
- ◯ 79
- ◯ 80
- ◯ 81
- ◯ 82
- ◯ 83
- ◯ 84
- ◯ 85
- ◯ 86
- ◯ 87
- ◯ 88
- ◯ 89
- ◯ 90
- ◯ 91
- ◯ 92
- ◯ 93
- ◯ 94
- ◯ 95
- ◯ 96
- ◯ 97
- ◯ 98
- ◯ 99
- ◯ Don't know

At what age were you first told you had [allergy_others_2]?

◯ 0
◯ 1
◯ 2
◯ 3
◯ 4
◯ 5
◯ 6
◯ 7
◯ 8
◯ 9
◯ 10
◯ 11
◯ 12
◯ 13
◯ 14
◯ 15
◯ 16
◯ 17
◯ 18
◯ 19
◯ 20
◯ 21
◯ 22
◯ 23
◯ 24
◯ 25
◯ 26
◯ 27
◯ 28
◯ 29
◯ 30
◯ 31
◯ 32
◯ 33
◯ 34
◯ 35
◯ 36
◯ 37
◯ 38
◯ 39
◯ 40
◯ 41
◯ 42
◯ 43
◯ 44
◯ 45
◯ 46
◯ 47
◯ 48
◯ 49
◯ 50
◯ 51
◯ 52
◯ 53
◯ 54
◯ 55
◯ 56
◯ 57
◯ 58
◯ 59
◯ 60
◯ 61
◯ 62
◯ 63
◯ 64
◯ 65
◯ 66
◯ 67
◯ 68

- ◯ 69
- ◯ 70
- ◯ 71
- ◯ 72
- ◯ 73
- ◯ 74
- ◯ 75
- ◯ 76
- ◯ 77
- ◯ 78
- ◯ 79
- ◯ 80
- ◯ 81
- ◯ 82
- ◯ 83
- ◯ 84
- ◯ 85
- ◯ 86
- ◯ 87
- ◯ 88
- ◯ 89
- ◯ 90
- ◯ 91
- ◯ 92
- ◯ 93
- ◯ 94
- ◯ 95
- ◯ 96
- ◯ 97
- ◯ 98
- ◯ 99
- ◯ Don't know

At what age were you first told you had [allergy_others_3]?

- 0
- 1
- 2
- 3
- 4
- 5
- 6
- 7
- 8
- 9
- 10
- 11
- 12
- 13
- 14
- 15
- 16
- 17
- 18
- 19
- 20
- 21
- 22
- 23
- 24
- 25
- 26
- 27
- 28
- 29
- 30
- 31
- 32
- 33
- 34
- 35
- 36
- 37
- 38
- 39
- 40
- 41
- 42
- 43
- 44
- 45
- 46
- 47
- 48
- 49
- 50
- 51
- 52
- 53
- 54
- 55
- 56
- 57
- 58
- 59
- 60
- 61
- 62
- 63
- 64
- 65
- 66
- 67
- 68

◯ 69
◯ 70
◯ 71
◯ 72
◯ 73
◯ 74
◯ 75
◯ 76
◯ 77
◯ 78
◯ 79
◯ 80
◯ 81
◯ 82
◯ 83
◯ 84
◯ 85
◯ 86
◯ 87
◯ 88
◯ 89
◯ 90
◯ 91
◯ 92
◯ 93
◯ 94
◯ 95
◯ 96
◯ 97
◯ 98
◯ 99
◯ Don't know

At what age were you first told you had this condition?

- ◯ 0
- ◯ 1
- ◯ 2
- ◯ 3
- ◯ 4
- ◯ 5
- ◯ 6
- ◯ 7
- ◯ 8
- ◯ 9
- ◯ 10
- ◯ 11
- ◯ 12
- ◯ 13
- ◯ 14
- ◯ 15
- ◯ 16
- ◯ 17
- ◯ 18
- ◯ 19
- ◯ 20
- ◯ 21
- ◯ 22
- ◯ 23
- ◯ 24
- ◯ 25
- ◯ 26
- ◯ 27
- ◯ 28
- ◯ 29
- ◯ 30
- ◯ 31
- ◯ 32
- ◯ 33
- ◯ 34
- ◯ 35
- ◯ 36
- ◯ 37
- ◯ 38
- ◯ 39
- ◯ 40
- ◯ 41
- ◯ 42
- ◯ 43
- ◯ 44
- ◯ 45
- ◯ 46
- ◯ 47
- ◯ 48
- ◯ 49
- ◯ 50
- ◯ 51
- ◯ 52
- ◯ 53
- ◯ 54
- ◯ 55
- ◯ 56
- ◯ 57
- ◯ 58
- ◯ 59
- ◯ 60
- ◯ 61
- ◯ 62
- ◯ 63
- ◯ 64
- ◯ 65
- ◯ 66
- ◯ 67
- ◯ 68

	◯ 69<br>◯ 70<br>◯ 71<br>◯ 72<br>◯ 73<br>◯ 74<br>◯ 75<br>◯ 76<br>◯ 77<br>◯ 78<br>◯ 79<br>◯ 80<br>◯ 81<br>◯ 82<br>◯ 83<br>◯ 84<br>◯ 85<br>◯ 86<br>◯ 87<br>◯ 88<br>◯ 89<br>◯ 90<br>◯ 91<br>◯ 92<br>◯ 93<br>◯ 94<br>◯ 95<br>◯ 96<br>◯ 97<br>◯ 98<br>◯ 99<br>◯ Don't know
Do you still have egg allergy?	◯ Yes<br>◯ No<br>◯ Don't know
Do you still have milk/dairy allergy?	◯ Yes<br>◯ No<br>◯ Don't know
Do you still have milk/dairy/lactose intolerance?	◯ Yes<br>◯ No<br>◯ Don't know
Do you still have peanut allergy?	◯ Yes<br>◯ No<br>◯ Don't know
Do you still have nut allergy (e.g. cashew, hazelnut, walnut)?	◯ Yes<br>◯ No<br>◯ Don't know
Do you still have fish allergy?	◯ Yes<br>◯ No<br>◯ Don't know
Do you still have shellfish allergy?	◯ Yes<br>◯ No<br>◯ Don't know

Do you still have soy allergy?	◯ Yes<br>◯ No<br>◯ Don't know
Do you still have lupin allergy?	◯ Yes<br>◯ No<br>◯ Don't know
Do you still have coeliac disease?	◯ Yes<br>◯ No<br>◯ Don't know
Do you still have gluten/wheat sensitivity (non-coeliac disease)?	◯ Yes<br>◯ No<br>◯ Don't know
Do you still have wheat allergy?	◯ Yes<br>◯ No<br>◯ Don't know
Do you still have yeast allergy?	◯ Yes<br>◯ No<br>◯ Don't know
Do you still have salicylate intolerance?	◯ Yes<br>◯ No<br>◯ Don't know
Do you still have amine intolerance?	◯ Yes<br>◯ No<br>◯ Don't know
Do you still have glutamate intolerance?	◯ Yes<br>◯ No<br>◯ Don't know
Do you still have colour/flavour/preservative (e.g. sulphite) intolerance?	◯ Yes<br>◯ No<br>◯ Don't know
Do you still have FODMAPS intolerance (e.g. fructans, fructose)?	◯ Yes<br>◯ No<br>◯ Don't know
Do you still have [allergy_others]?	◯ Yes<br>◯ No<br>◯ Don't know
Do you still have [allergy_others_2]?	◯ Yes<br>◯ No<br>◯ Don't know
Do you still have [allergy_others_3]?	◯ Yes<br>◯ No<br>◯ Don't know

Do you still have this condition?	◯ Yes<br>◯ No<br>◯ Don't know

How is egg allergy treated?
(Tick all that apply)

☐ no treatment ☐ diet ☐ exercise ☐ medication (please specify) ☐ natural/herbal therapy
☐ weight management ☐ supplements (please specify) ☐ other (please specify) ☐ Don't know

please specify the medication ______________________

please specify the treatment ______________________

please specify the medication ______________________

How is cow's milk/dairy allergy treated?
(Tick all that apply)

☐ no treatment ☐ diet ☐ exercise ☐ medication (please specify) ☐ natural/herbal therapy
☐ weight management ☐ supplements (please specify) ☐ other (please specify) ☐ Don't know

please specify the medication ______________________

please specify the supplements ______________________

please specify the treatment ______________________

How is cow's milk/dairy/lactose intolerance treated?
(Tick all that apply)

☐ no treatment ☐ diet ☐ exercise ☐ medication (please specify) ☐ natural/herbal therapy
☐ weight management ☐ supplements (please specify) ☐ other (please specify) ☐ Don't know

please specify the medication ______________________

please specify the treatment ______________________

How is peanut allergy treated?
(Tick all that apply)

☐ no treatment ☐ diet ☐ exercise ☐ medication (please specify) ☐ natural/herbal therapy
☐ weight management ☐ supplements (please specify) ☐ other (please specify) ☐ Don't know

please specify the medication ______________________________

please specify the supplements ______________________________

please specify the supplements ______________________________

please specify the treatment ______________________________

How is tree nut allergy (e.g. cashew, hazelnut, walnut) treated?
(Tick all that apply)

☐ no treatment ☐ diet ☐ exercise ☐ medication (please specify) ☐ natural/herbal therapy
☐ weight management ☐ supplements (please specify) ☐ other (please specify) ☐ Don't know

please specify the medication ______________________________

please specify the supplements ______________________________

please specify the treatment ______________________________

How is fish allergy treated?
(Tick all that apply)

☐ no treatment ☐ diet ☐ exercise ☐ medication (please specify) ☐ natural/herbal therapy
☐ weight management ☐ supplements (please specify) ☐ other (please specify) ☐ Don't know

please specify the medication ______________________________

please specify the supplements ______________________________

please specify the treatment ______________________________

How is shellfish allergy treated?

☐ no treatment ☐ diet ☐ exercise ☐ medication (please specify) ☐ natural/herbal therapy
☐ weight management ☐ supplements (please specify) ☐ other (please specify) ☐ Don't know

please specify the medication
____

please specify the supplements
____

please specify the treatment
____

How is soy allergy treated?
(Tick all that apply)

☐ no treatment ☐ diet ☐ exercise ☐ medication (please specify) ☐ natural/herbal therapy
☐ weight management ☐ supplements (please specify) ☐ other (please specify) ☐ Don't know

please specify the medication
____

please specify the supplements
____

please specify the treatment
____

How is lupin allergy treated?
(Tick all that apply)

☐ no treatment ☐ diet ☐ exercise ☐ medication (please specify) ☐ natural/herbal therapy
☐ weight management ☐ supplements (please specify) ☐ other (please specify) ☐ Don't know

please specify the medication
____

please specify the supplements
____

please specify the treatment
____

How is coeliac disease (confirmed by biopsy) treated?
(Tick all that apply)

☐ no treatment ☐ diet ☐ exercise ☐ medication (please specify) ☐ natural/herbal therapy
☐ weight management ☐ supplements (please specify) ☐ other (please specify) ☐ Don't know

please specify the medication
____

please specify the supplements
____

please specify the treatment

____

How is gluten/wheat sensitivity (non-coeliac disease) treated?
(Tick all that apply)

☐ no treatment ☐ diet ☐ exercise ☐ medication (please specify) ☐ natural/herbal therapy
☐ weight management ☐ supplements (please specify) ☐ other (please specify) ☐ Don't know

please specify the medication

____

please specify the supplements

____

please specify the treatment

____

How is wheat allergy treated?
(Tick all that apply)

☐ no treatment ☐ diet ☐ exercise ☐ medication (please specify) ☐ natural/herbal therapy
☐ weight management ☐ supplements (please specify) ☐ other (please specify) ☐ Don't know

please specify the medication

____

please specify the supplements

____

please specify the treatment

____

How is yeast allergy treated?
(Tick all that apply)

☐ no treatment ☐ diet ☐ exercise ☐ medication (please specify) ☐ natural/herbal therapy
☐ weight management ☐ supplements (please specify) ☐ other (please specify) ☐ Don't know

please specify the medication

____

please specify the supplements

____

please specify the treatment

____

How is salicylate intolerance treated?
(Tick all that apply)

☐ no treatment ☐ diet ☐ exercise ☐ medication (please specify) ☐ natural/herbal therapy
☐ weight management ☐ supplements (please specify) ☐ other (please specify) ☐ Don't know

please specify the medication ______________________

please specify the supplements ______________________

please specify the treatment ______________________

How is amine intolerance treated?
(Tick all that apply)

☐ no treatment ☐ diet ☐ exercise ☐ medication (please specify) ☐ natural/herbal therapy
☐ weight management ☐ supplements (please specify) ☐ other (please specify) ☐ Don't know

please specify the medication ______________________

please specify the supplements ______________________

please specify the treatment ______________________

How is glutamate intolerance treated?
(Tick all that apply)

☐ no treatment ☐ diet ☐ exercise ☐ medication (please specify) ☐ natural/herbal therapy
☐ weight management ☐ supplements (please specify) ☐ other (please specify) ☐ Don't know

please specify the medication ______________________

please specify the supplements ______________________

please specify the treatment ______________________

How is colour/flavour/preservative (e.g. sulphite) intolerance treated?
(Tick all that apply)

☐ no treatment ☐ diet ☐ exercise ☐ medication (please specify) ☐ natural/herbal therapy
☐ weight management ☐ supplements (please specify) ☐ other (please specify) ☐ Don't know

please specify the medication ______________________

please specify the supplements ______________________

please specify the treatment

____

How is FODMAPS intolerance (e.g. fructans, fructose) treated?
(Tick all that apply)

☐ no treatment ☐ diet ☐ exercise ☐ medication (please specify) ☐ natural/herbal therapy
☐ weight management ☐ supplements (please specify) ☐ other (please specify) ☐ Don't know

please specify the medication

____

please specify the supplements

____

please specify the treatment

____

How is [allergy_others] treated?
(Tick all that apply)

☐ no treatment ☐ diet ☐ exercise ☐ medication (please specify) ☐ natural/herbal therapy
☐ weight management ☐ supplements (please specify) ☐ other (please specify) ☐ Don't know

please specify the medication

____

please specify the supplements

____

please specify the treatment

____

How is [allergy_others_2] treated?
(Tick all that apply)

☐ no treatment ☐ diet ☐ exercise ☐ medication (please specify) ☐ natural/herbal therapy
☐ weight management ☐ supplements (please specify) ☐ other (please specify) ☐ Don't know

please specify the medication

____

please specify the supplements

____

please specify the treatment

____

How is [allergy_others_3] treated?
(Tick all that apply)

☐ no treatment ☐ diet ☐ exercise ☐ medication (please specify) ☐ natural/herbal therapy
☐ weight management ☐ supplements (please specify) ☐ other (please specify) ☐ Don't know

please specify the medication

____

please specify the supplements

____

please specify the treatment

____

How is this condition treated?
(Tick all that apply)

☐ no treatment ☐ diet ☐ exercise ☐ medication (please specify) ☐ natural/herbal therapy
☐ weight management ☐ supplements (please specify) ☐ other (please specify) ☐ Don't know

please specify the medication

____

please specify the supplements

____

please specify the treatment

____

Have either of your parents, or any of brothers or sisters ever been told by a doctor (GP or specialist), or dietitian that they any food allergy or intolerance?

◯ Yes
◯ No
◯ Don't know

This question is about conditions that you may experience specifically related to training or competing in sport. Please tick any conditions that you regularly experience in training or competition.
(Tick all that apply)

☐ Cramps
☐ Dehydration/hypohydration
☐ Diahrroea
☐ Nausea/vomiting
☐ Stitches
☐ Other (please specify)
☐ Don't Know
☐ I don't experience any conditions related to training or competition

please specify the condition

____

Do you have another condition need to be specified?

◯ Yes ◯ No

please specify the second condition

____

Do you have another condition need to be specified?

◯ Yes ◯ No

please specify the third condition

____

Do you experience any vitamin or mineral deficiencies diagnosed by a blood test?

◯ Yes
◯ No
◯ Don't know

Which vitamin or mineral deficiencies have been diagnosed by a blood test? (Tick all that apply)

☐ Calcium deficiency
☐ Iron deficiency (e.g. low ferritin, low iron, low haemaglobin)
☐ Vitamin D deficiency
☐ Other (please specify)
☐ Don't Know

please specify the vitamin or mineral deficiency

____

Do you have another deficiency need to be specified?

◯ Yes ◯ No

please specify the second vitamin or mineral deficiency

____

Do you have another deficiency need to be specified?

◯ Yes ◯ No

please specify the third vitamin or mineral deficiency

____

Do you take *sports foods or dietary supplements?

◯ Yes
◯ No
◯ Don't know

*For example, sports drink, sports gel, sports bars, sports confectionary, liquid meals, electrolyte replacement (salt tablets), whey protein, creatine, carnitine etc..

Which sports foods or dietary supplements do you take?
(Tick all that apply)

- ☐ beta-alanine
- ☐ bicarbonate
- ☐ beetroot juice
- ☐ caffeine supplements (e.g. strips or tablets)
- ☐ caffeine containing foods taken to assist performance (e.g. coffee, chocolate milk)
- ☐ calcium supplement
- ☐ carnitine
- ☐ chondroitin sulphate
- ☐ creatine
- ☐ curcumin
- ☐ electrolyte replacement (e.g. salt tablets)
- ☐ Exotic berries (e.g. acai, goji)
- ☐ fish oil capsules
- ☐ glucosamine
- ☐ glutamine
- ☐ HMB
- ☐ iron supplement
- ☐ liquid meals
- ☐ multivitamin/mineral supplement
- ☐ probiotics
- ☐ quercetin
- ☐ sports bars
- ☐ sports confectionery (e.g. chews)
- ☐ sports drink
- ☐ sports gel
- ☐ tart (Montomorency) cherry
- ☐ Vitamin C
- ☐ vitamin D supplement
- ☐ Vitamin E
- ☐ whey protein
- ☐ Other anti-oxidants (e.g. vitamin A, selenium)
- ☐ Don't know

How do you ensure that your supplements are safe (that is that they are not contaminated or banned)?
(Tick all that apply)

- ☐ I check the ASADA/WADA prohibited substances list
- ☐ I check that third party testing has been done (e.g. by Informed Sport, HASTA, etc)
- ☐ I take the recommendation of a health or sport professional
- ☐ I take the recommendation of the supplement company (e.g. on the packet or website)
- ☐ I take the recommendation of other athlete/s
- ☐ I use an app (please specify)
- ☐ I don't take any measures to ensure they are safe.
- ☐ Don't Know

---

please specify the app you are using ________________________________

Do you have any other comments about the testing of your supplements? ________________________________

Which health or sport professional/s make recommendations to you about the safety of your supplements?
(Tick all that apply)

- ☐ General practitioner (GP)
- ☐ Medical specialist
- ☐ Nurse
- ☐ Dietitian/Nutritionist (general)
- ☐ Sports Dietitian/Nutritionist
- ☐ Naturopath
- ☐ Exercise Physiologist
- ☐ Physiotherapist
- ☐ Personal trainer
- ☐ Coach
- ☐ Other (please specify)
- ☐ Don't Know

please specify the health or sport professional

____

This is a list of sources of nutrition information.
Please list in order of importance where you look for nutrition information (1 is the most important and 5 is the least important). Up to 5 options can be selected.

	1	2	3	4	5
General practitioner (GP)	◯	◯	◯	◯	◯
Medical specialist	◯	◯	◯	◯	◯
Nurse	◯	◯	◯	◯	◯
Dietitian/Nutritionist (general)	◯	◯	◯	◯	◯
Sports Dietitian/Nutritionist	◯	◯	◯	◯	◯
Naturopath	◯	◯	◯	◯	◯
Exercise Physiologist	◯	◯	◯	◯	◯
Physiotherapist	◯	◯	◯	◯	◯
Personal trainer	◯	◯	◯	◯	◯
Coach	◯	◯	◯	◯	◯
Local health food/supplement store	◯	◯	◯	◯	◯
Internet	◯	◯	◯	◯	◯
Magazines	◯	◯	◯	◯	◯
Other	◯	◯	◯	◯	◯
Don't know	◯	◯	◯	◯	◯

please specify the source ______________________________

Are you interested in receiving education in nutrition, health, and performance?

◯ Yes
◯ No
◯ Don't know

This is a list of sources of nutrition, health and performance education sources. Please list in order of importance where you look for nutrition information (1 is the most important and 5 is the least important). Up to 5 options can be selected.

	1	2	3	4	5
Consultation with a dietitian	○	○	○	○	○
Group education session	○	○	○	○	○
Webinar	○	○	○	○	○
Face-to-face nutrition course	○	○	○	○	○
Online nutrition course	○	○	○	○	○
Written materials	○	○	○	○	○
Scientific journals	○	○	○	○	○
Website information	○	○	○	○	○
Nutrition Blog	○	○	○	○	○
Other	○	○	○	○	○

please specify the source

____
