# Supplementary material for: Prevalence of chronic conditions in masters games athletes: predictors and comparison to the general population
Source: PeerJ. 2025 Feb 18;13:e18912. doi: 10.7717/peerj.18912 (PMC11844256; doi:10.7717/peerj.18912)
Supplement: Supplemental Information 2 [file peerj-13-18912-s002.pdf]

Survey to investigate the prevalence of nutrition-related chronic conditions in Australian Masters athletes

This is an online survey asking registered Pan Pacific Masters Games athletes about demographic information, health-related information including medical history and treatment strategies, history of engagement with physical activity, and sources of nutrition information. The physiological effects of the normal ageing process alongside age- or lifestyle-associated health conditions need to be taken into account when recommendations are provided for optimal athletic performance for Masters Athletes. This research will help to inform health professionals about education needs and advice for treatment and performance, and guide further research specific to Masters Athletes. Promotion of the health benefits Masters level activity may encourage others in this age-group to consider participation.

The study is a University of Sydney project being conducted by Dr Janelle Gifford, Mrs Wendy Stuart-Smith, Associate Professor Mike Climstein, Associate Professor Helen O'Connor, Ms Xiaojing Wu, and Professor Peter Raeburn. Contact email: janelle.gifford@sydney.edu.au

---

By clicking the "Continue" button at the bottom of the screen, you are:
1. acknowledging that you have read the information sheet, which explains the aims of the study and have contacted the study researchers if you needed to clarify the details of the study;
2. acknowledging that you understand that you can withdraw from the study at any time without prejudice to your relationship to The University of Sydney;
3. agreeing that research data gathered from the results of the study may be published, provided that you cannot be identified;
4. acknowledging that you understand that if you have any questions relating to your participation in this research, and you may contact Dr Janelle Gifford at janelle.gifford@sydney.edu.au, who will be happy to answer them;
5. consenting to participate in the study described in the information sheet provided.

Please be assured that no information that can identify you will be kept, and that the information you enter will be securely held in confidence.

[Attachment: "Participant Information Sheet.pdf"]

Consent to participate

◯ Choose not to participate
◯ CONTINUE   (If you click continue, you are consenting to participate in the study described in the information sheet provided above.)

If you click continue, you are consenting to participate in the study described in the information sheet provided.

[Attachment: "Participant Information Sheet.pdf"]

Have you already participated in this survey?*

◯ Yes
◯ No
(*Participation includes completion of this survey at the Australian Masters Games (Tasmania) in 2017)

What is your age in years?

- ◯ < 18
- ◯ 18
- ◯ 19
- ◯ 20
- ◯ 21
- ◯ 22
- ◯ 23
- ◯ 24
- ◯ 25
- ◯ 26
- ◯ 27
- ◯ 28
- ◯ 29
- ◯ 30
- ◯ 31
- ◯ 32
- ◯ 33
- ◯ 34
- ◯ 35
- ◯ 36
- ◯ 37
- ◯ 38
- ◯ 39
- ◯ 40
- ◯ 41
- ◯ 42
- ◯ 43
- ◯ 44
- ◯ 45
- ◯ 46
- ◯ 47
- ◯ 48
- ◯ 49
- ◯ 50
- ◯ 51
- ◯ 52
- ◯ 53
- ◯ 54
- ◯ 55
- ◯ 56
- ◯ 57
- ◯ 58
- ◯ 59
- ◯ 60
- ◯ 61
- ◯ 62
- ◯ 63
- ◯ 64
- ◯ 65
- ◯ 66
- ◯ 67
- ◯ 68
- ◯ 69
- ◯ 70
- ◯ 71
- ◯ 72
- ◯ 73
- ◯ 74
- ◯ 75
- ◯ 76
- ◯ 77
- ◯ 78
- ◯ 79
- ◯ 80
- ◯ 81
- ◯ 82
- ◯ 83
- ◯ 84
- ◯ 85

	◯ 86<br>◯ 87<br>◯ 88<br>◯ 89<br>◯ 90<br>◯ 91<br>◯ 92<br>◯ 93<br>◯ 94<br>◯ 95<br>◯ 96<br>◯ 97<br>◯ 98<br>◯ 99<br>◯ 100
Are you an athlete registered to participate in the Pan Pacific Masters Games?	◯ Yes<br>◯ No
What is your gender?	◯ Male<br>◯ Female

Do any of the following apply to you?
1. Currently pregnant
2. Currently breastfeeding
3. Currently experiencing menopause
4. Post menopause

◯ Yes ◯ No

Are you pregnant or breastfeeding?

◯ Yes ◯ No

Which of the following apply to you? (pregnant and breastfeeding can both be selected)
(Tick all that apply)

☐ Currently pregnant ☐ Currently breastfeeding

Which of the following apply to you?

◯ Currently experiencing menopause ◯ Post menopause

How many weeks pregnant are you?

- ◯ < 12 weeks
- ◯ 12 weeks
- ◯ 13 weeks
- ◯ 14 weeks
- ◯ 15 weeks
- ◯ 16 weeks
- ◯ 17 weeks
- ◯ 18 weeks
- ◯ 19 weeks
- ◯ 20 weeks
- ◯ 21 weeks
- ◯ 22 weeks
- ◯ 23 weeks
- ◯ 24 weeks
- ◯ 25 weeks
- ◯ 26 weeks
- ◯ 27 weeks
- ◯ 28 weeks
- ◯ 29 weeks
- ◯ 30 weeks
- ◯ 31 weeks
- ◯ 32 weeks
- ◯ 33 weeks
- ◯ 34 weeks
- ◯ 35 weeks
- ◯ 36 weeks
- ◯ 37 weeks
- ◯ 38 weeks
- ◯ 39 weeks
- ◯ 40 weeks
- ◯ Don't know

Do you speak English at home?

- ◯ Yes
- ◯ No

Are you Aboriginal or Torres Strait Islander origin?

- ◯ Yes
- ◯ No

What is your ancestry?
(Select up to two (2) options)
(*Up to two may be specified if no other options are ticked. Examples of "Other": Greek, Vietnamese, Hmong, Kurdish, Maori, Lebanese, Australian South East Islander)

- ☐ English
- ☐ Irish
- ☐ Scottish
- ☐ Italian
- ☐ German
- ☐ Chinese
- ☐ Australian
- ☐ Other*

Please specify your ancestry

____

Do you have another ancestry you need to specify?

- ◯ Yes
- ◯ No

Please specify your ancestry

____

What is your current nationality?

- ◯ Afghanistan
- ◯ Albania
- ◯ Algeria
- ◯ Andorra
- ◯ Angola
- ◯ Antigua & Deps
- ◯ Argentina
- ◯ Armenia
- ◯ Australia
- ◯ Austria
- ◯ Azerbaijan
- ◯ Bahamas
- ◯ Bahrain
- ◯ Bangladesh
- ◯ Barbados
- ◯ Belarus
- ◯ Belgium
- ◯ Belize
- ◯ Benin
- ◯ Bhutan
- ◯ Bolivia
- ◯ Bosnia Herzegovina
- ◯ Botswana
- ◯ Brazil
- ◯ Brunei
- ◯ Bulgaria
- ◯ Burkina
- ◯ Burundi
- ◯ Cambodia
- ◯ Cameroon
- ◯ Canada
- ◯ Cape Verde
- ◯ Central African Rep
- ◯ Chad
- ◯ Chile
- ◯ China
- ◯ Colombia
- ◯ Comoros
- ◯ Congo
- ◯ Congo {Democratic Rep}
- ◯ Costa Rica
- ◯ Croatia
- ◯ Cuba
- ◯ Cyprus
- ◯ Czech Republic
- ◯ Denmark
- ◯ Djibouti
- ◯ Dominica
- ◯ Dominican Republic
- ◯ East Timor
- ◯ Ecuador
- ◯ Egypt
- ◯ El Salvador
- ◯ Equatorial Guinea
- ◯ Eritrea
- ◯ Estonia
- ◯ Ethiopia
- ◯ Fiji
- ◯ Finland
- ◯ France
- ◯ Gabon
- ◯ Gambia
- ◯ Georgia
- ◯ Germany
- ◯ Ghana
- ◯ Greece
- ◯ Grenada
- ◯ Guatemala
- ◯ Guinea

◯ Guinea-Bissau
◯ Guyana
◯ Haiti
◯ Honduras
◯ Hungary
◯ Iceland
◯ India
◯ Indonesia
◯ Iran
◯ Iraq
◯ Ireland {Republic}
◯ Israel
◯ Italy
◯ Ivory Coast
◯ Jamaica
◯ Japan
◯ Jordan
◯ Kazakhstan
◯ Kenya
◯ Kiribati
◯ Korea North
◯ Korea South
◯ Kosovo
◯ Kuwait
◯ Kyrgyzstan
◯ Laos
◯ Latvia
◯ Lebanon
◯ Lesotho
◯ Liberia
◯ Libya
◯ Liechtenstein
◯ Lithuania
◯ Luxembourg
◯ Macedonia
◯ Madagascar
◯ Malawi
◯ Malaysia
◯ Maldives
◯ Mali
◯ Malta
◯ Marshall Islands
◯ Mauritania
◯ Mauritius
◯ Mexico
◯ Micronesia
◯ Moldova
◯ Monaco
◯ Mongolia
◯ Montenegro
◯ Morocco
◯ Mozambique
◯ Myanmar {Burma}
◯ Namibia
◯ Nauru
◯ Nepal
◯ Netherlands
◯ New Zealand
◯ Nicaragua
◯ Niger
◯ Nigeria
◯ Norway
◯ Oman
◯ Pakistan
◯ Palau
◯ Panama
◯ Papua New Guinea
◯ Paraguay
◯ Peru
◯ Philippines
◯ Poland

◯ Portugal
◯ Qatar
◯ Romania
◯ Russian Federation
◯ Rwanda
◯ St Kitts & Nevis
◯ St Lucia
◯ Saint Vincent & the Grenadines
◯ Samoa
◯ San Marino
◯ Sao Tome & Principe
◯ Saudi Arabia
◯ Senegal
◯ Serbia
◯ Seychelles
◯ Sierra Leone
◯ Singapore
◯ Slovakia
◯ Slovenia
◯ Solomon Islands
◯ Somalia
◯ South Africa
◯ Spain
◯ Sri Lanka
◯ Sudan
◯ Suriname
◯ Swaziland
◯ Sweden
◯ Switzerland
◯ Syria
◯ Taiwan
◯ Tajikistan
◯ Tanzania
◯ Thailand
◯ Togo
◯ Tonga
◯ Trinidad & Tobago
◯ Tunisia
◯ Turkey
◯ Turkmenistan
◯ Tuvalu
◯ Uganda
◯ Ukraine
◯ United Arab Emirates
◯ United Kingdom
◯ United States
◯ Uruguay
◯ Uzbekistan
◯ Vanuatu
◯ Vatican City
◯ Venezuela
◯ Vietnam
◯ Yemen
◯ Zambia
◯ Zimbabwe

In which country do you live?

- ◯ Afghanistan
- ◯ Albania
- ◯ Algeria
- ◯ Andorra
- ◯ Angola
- ◯ Antigua & Deps
- ◯ Argentina
- ◯ Armenia
- ◯ Australia
- ◯ Austria
- ◯ Azerbaijan
- ◯ Bahamas
- ◯ Bahrain
- ◯ Bangladesh
- ◯ Barbados
- ◯ Belarus
- ◯ Belgium
- ◯ Belize
- ◯ Benin
- ◯ Bhutan
- ◯ Bolivia
- ◯ Bosnia Herzegovina
- ◯ Botswana
- ◯ Brazil
- ◯ Brunei
- ◯ Bulgaria
- ◯ Burkina
- ◯ Burundi
- ◯ Cambodia
- ◯ Cameroon
- ◯ Canada
- ◯ Cape Verde
- ◯ Central African Rep
- ◯ Chad
- ◯ Chile
- ◯ China
- ◯ Colombia
- ◯ Comoros
- ◯ Congo
- ◯ Congo {Democratic Rep}
- ◯ Costa Rica
- ◯ Croatia
- ◯ Cuba
- ◯ Cyprus
- ◯ Czech Republic
- ◯ Denmark
- ◯ Djibouti
- ◯ Dominica
- ◯ Dominican Republic
- ◯ East Timor
- ◯ Ecuador
- ◯ Egypt
- ◯ El Salvador
- ◯ Equatorial Guinea
- ◯ Eritrea
- ◯ Estonia
- ◯ Ethiopia
- ◯ Fiji
- ◯ Finland
- ◯ France
- ◯ Gabon
- ◯ Gambia
- ◯ Georgia
- ◯ Germany
- ◯ Ghana
- ◯ Greece
- ◯ Grenada
- ◯ Guatemala
- ◯ Guinea

◯ Guinea-Bissau
◯ Guyana
◯ Haiti
◯ Honduras
◯ Hungary
◯ Iceland
◯ India
◯ Indonesia
◯ Iran
◯ Iraq
◯ Ireland {Republic}
◯ Israel
◯ Italy
◯ Ivory Coast
◯ Jamaica
◯ Japan
◯ Jordan
◯ Kazakhstan
◯ Kenya
◯ Kiribati
◯ Korea North
◯ Korea South
◯ Kosovo
◯ Kuwait
◯ Kyrgyzstan
◯ Laos
◯ Latvia
◯ Lebanon
◯ Lesotho
◯ Liberia
◯ Libya
◯ Liechtenstein
◯ Lithuania
◯ Luxembourg
◯ Macedonia
◯ Madagascar
◯ Malawi
◯ Malaysia
◯ Maldives
◯ Mali
◯ Malta
◯ Marshall Islands
◯ Mauritania
◯ Mauritius
◯ Mexico
◯ Micronesia
◯ Moldova
◯ Monaco
◯ Mongolia
◯ Montenegro
◯ Morocco
◯ Mozambique
◯ Myanmar {Burma}
◯ Namibia
◯ Nauru
◯ Nepal
◯ Netherlands
◯ New Zealand
◯ Nicaragua
◯ Niger
◯ Nigeria
◯ Norway
◯ Oman
◯ Pakistan
◯ Palau
◯ Panama
◯ Papua New Guinea
◯ Paraguay
◯ Peru
◯ Philippines
◯ Poland

- ◯ Portugal
- ◯ Qatar
- ◯ Romania
- ◯ Russian Federation
- ◯ Rwanda
- ◯ St Kitts & Nevis
- ◯ St Lucia
- ◯ Saint Vincent & the Grenadines
- ◯ Samoa
- ◯ San Marino
- ◯ Sao Tome & Principe
- ◯ Saudi Arabia
- ◯ Senegal
- ◯ Serbia
- ◯ Seychelles
- ◯ Sierra Leone
- ◯ Singapore
- ◯ Slovakia
- ◯ Slovenia
- ◯ Solomon Islands
- ◯ Somalia
- ◯ South Africa
- ◯ Spain
- ◯ Sri Lanka
- ◯ Sudan
- ◯ Suriname
- ◯ Swaziland
- ◯ Sweden
- ◯ Switzerland
- ◯ Syria
- ◯ Taiwan
- ◯ Tajikistan
- ◯ Tanzania
- ◯ Thailand
- ◯ Togo
- ◯ Tonga
- ◯ Trinidad & Tobago
- ◯ Tunisia
- ◯ Turkey
- ◯ Turkmenistan
- ◯ Tuvalu
- ◯ Uganda
- ◯ Ukraine
- ◯ United Arab Emirates
- ◯ United Kingdom
- ◯ United States
- ◯ Uruguay
- ◯ Uzbekistan
- ◯ Vanuatu
- ◯ Vatican City
- ◯ Venezuela
- ◯ Vietnam
- ◯ Yemen
- ◯ Zambia
- ◯ Zimbabwe

How many years have you lived in [country_live]?

◯ 1
◯ 2
◯ 3
◯ 4
◯ 5
◯ 6
◯ 7
◯ 8
◯ 9
◯ 10
◯ 11
◯ 12
◯ 13
◯ 14
◯ 15
◯ 16
◯ 17
◯ 18
◯ 19
◯ 20
◯ 21
◯ 22
◯ 23
◯ 24
◯ 25
◯ 26
◯ 27
◯ 28
◯ 29
◯ 30
◯ 31
◯ 32
◯ 33
◯ 34
◯ 35
◯ 36
◯ 37
◯ 38
◯ 39
◯ 40
◯ 41
◯ 42
◯ 43
◯ 44
◯ 45
◯ 46
◯ 47
◯ 48
◯ 49
◯ 50
◯ 51
◯ 52
◯ 53
◯ 54
◯ 55
◯ 56
◯ 57
◯ 58
◯ 59
◯ 60
◯ 61
◯ 62
◯ 63
◯ 64
◯ 65
◯ 66
◯ 67
◯ 68
◯ 69

◯ 70
◯ 71
◯ 72
◯ 73
◯ 74
◯ 75
◯ 76
◯ 77
◯ 78
◯ 79
◯ 80
◯ 81
◯ 82
◯ 83
◯ 84
◯ 85
◯ 86
◯ 87
◯ 88
◯ 89
◯ 90
◯ 91
◯ 92
◯ 93
◯ 94
◯ 95
◯ 96
◯ 97
◯ 98
◯ 99
◯ 100
◯ >100
◯ Don't know

In which state or territory of Australia do you currently live?

◯ ACT
◯ NSW
◯ NT
◯ Queensland
◯ SA
◯ Tasmania
◯ Victoria
◯ Western Australia
◯ Not currently living in Australia

How would you describe where you live?

◯ Major urban centre (population of 100,000 or more)
◯ Other urban centre (population between 1000 and 99,999)
◯ Bounded locality (population between 200 and 999)
◯ Rural (population < 200)

What is the highest year of primary or secondary school that you have completed?

◯ Year 12 or equivalent
◯ Year 11 or equivalent
◯ Year 10 or equivalent
◯ Year 9 or equivalent
◯ Year 8 or below
◯ Never attended school

Have you completed a trade certificate, diploma, degree or any other educational qualification?

◯ Yes
◯ No
◯ Don't know

What is the highest qualification that you have completed?

- ◯ Trade certificate
- ◯ Diploma
- ◯ Bachelors degree
- ◯ Graduate diploma or certificate
- ◯ Masters degree
- ◯ PhD
- ◯ Other

---

please specify the highest qualification you have completed

____

This question is about your employment. Are you currently....
(More than one response is allowed)
(Tick all that apply)

- ☐ Employed casually for wages
- ☐ Employed part-time for wages
- ☐ Employed full-time for wages
- ☐ Self-employed
- ☐ Unemployed
- ☐ A homemaker
- ☐ A student
- ☐ Retired
- ☐ Unable to work
- ☐ Other (please specify)

---

please specify your employment status

____

Before income tax, salary sacrifice or anything else is taken out, how much income in total do all members of your household (aged 15 years or over) usually receive from all sources? (amount is in Australian dollars)

- ◯ Prefer not to say
- ◯ Less than $20,000
- ◯ $20,001-$40,000
- ◯ $40,001-$60,000
- ◯ $60,001-$80,000
- ◯ $80,001-$100,000
- ◯ $100,001-$120,000
- ◯ $120,001-$140,000
- ◯ $140,001-$160,000
- ◯ $160,001-$180,000
- ◯ More than $180,000
- ◯ Don't Know

How long have you been competing in Masters level competition?

◯ Less than 1 year
◯ 1 year
◯ 2 years
◯ 3 years
◯ 4 years
◯ 5 years
◯ 6 years
◯ 7 years
◯ 8 years
◯ 9 years
◯ 10 years
◯ 11 years
◯ 12 years
◯ 13 years
◯ 14 years
◯ 15 years
◯ 16 years
◯ 17 years
◯ 18 years
◯ 19 years
◯ 20 years
◯ 21 years
◯ 22 years
◯ 23 years
◯ 24 years
◯ 25 years
◯ 26 years
◯ 27 years
◯ 28 years
◯ 29 years
◯ 30 years
◯ 31 years
◯ 32 years
◯ 33 years
◯ 34 years
◯ 35 years
◯ 36 years
◯ 37 years
◯ 38 years
◯ 39 years
◯ 40 years
◯ 41 years
◯ 42 years
◯ 43 years
◯ 44 years
◯ 45 years
◯ 46 years
◯ 47 years
◯ 48 years
◯ 49 years
◯ 50 years
◯ 51 years
◯ 52 years
◯ 53 years
◯ 54 years
◯ 55 years
◯ 56 years
◯ 57 years
◯ 58 years
◯ 59 years
◯ 60 years
◯ 61 years
◯ 62 years
◯ 63 years
◯ 64 years
◯ 65 years
◯ 66 years
◯ 67 years
◯ 68 years

◯ 69 years
◯ 70 years
◯ 71 years
◯ 72 years
◯ 73 years
◯ 74 years
◯ 75 years
◯ 76 years
◯ 77 years
◯ 78 years
◯ 79 years
◯ 80 years
◯ 81 years
◯ 82 years
◯ 83 years
◯ 84 years
◯ 85 years
◯ 86 years
◯ 87 years
◯ 88 years
◯ 89 years
◯ 90 years
◯ 91 years
◯ 92 years
◯ 93 years
◯ 94 years
◯ 95 years
◯ 96 years
◯ 97 years
◯ 98 years
◯ 99 years

What sport/s are you competing in at the Pan Pacific Masters Games?
(Tick all that apply)

- ☐ Archery
- ☐ Athletics
- ☐ Baseball
- ☐ Basketball
- ☐ Beach volleyball
- ☐ Boxing
- ☐ Callisthenics
- ☐ Cricket - outdoor
- ☐ Cricket - indoor
- ☐ Diving
- ☐ Enduro/Vinduro
- ☐ Equestrian
- ☐ Fitness challenge
- ☐ Futsal
- ☐ Golf
- ☐ Hockey
- ☐ Judo
- ☐ Lawn bowls
- ☐ Mountain bike
- ☐ Netball - outdoor
- ☐ Netball - indoor
- ☐ Paddling - dragon boat
- ☐ Paddling - outrigger canoe
- ☐ Rowing - indoor
- ☐ Rugby league
- ☐ Rugby union
- ☐ Sailing
- ☐ Shooting clay target
- ☐ Soccer
- ☐ Softball
- ☐ Squash
- ☐ Swimming (including ocean swim)
- ☐ Table tennis
- ☐ Taekwondo
- ☐ Tennis
- ☐ Tenpin Bowling
- ☐ Touch football
- ☐ Volleyball

Which categories will you compete in athletics?
(Tick all that apply)

- ☐ Sprints (60m, 100m, 200m, 400m), Hurdles (80m, 100m, 110m, 200m, 300m, 400m), Throws (shot put, hammer, javelin, discus, weight throw, throws pentathalon), Jumps (long jump, triple jump, high jump, pole vault)
- ☐ Middle Distance and Distance events (800m, 1500m, 5000m, 2000m steeplechase, 3000m steeplechase, 3000m race walk, 5000m race walk, 10km road race walk, beach mile, 5km road race, 4km cross country, 10.5km trail run)

Which categories will you compete in swimming?
(Tick all that apply)

- ☐ 1500m or ocean swim
- ☐ other swimming events

Have you ever been an elite athlete? (elite athletes compete in non-Masters adult competition at state, national or international level)

- ◯ Yes
- ◯ No
- ◯ Don't know

At which level did you compete in for each sport in elite competition? (select up to 5 sports) Please select the HIGHEST level you ever completed in your career.

	State	National	International	Don't Know
AFL	○	○	○	○
Archery	○	○	○	○
Athletics	○	○	○	○
Badminton	○	○	○	○
Baseball	○	○	○	○
Basketball	○	○	○	○
Beach volleyball	○	○	○	○
BMX	○	○	○	○
Bocce	○	○	○	○
Boxing	○	○	○	○
Callisthenics	○	○	○	○
Canoe/Kayak/SUP	○	○	○	○
Canoe/Paddling - outrigger	○	○	○	○
Cricket - outdoor	○	○	○	○
Cricket - indoor	○	○	○	○
Croquet	○	○	○	○
Cue sports	○	○	○	○
Cycling-track	○	○	○	○
Cycling-road	○	○	○	○
Darts	○	○	○	○
Diving	○	○	○	○
Dragon boat	○	○	○	○
Duathlon	○	○	○	○
Enduro/Vinduro	○	○	○	○
Equestrian	○	○	○	○
Fitness challenge	○	○	○	○
Fly fishing	○	○	○	○
Futsal	○	○	○	○
Golf	○	○	○	○
Gymnastics	○	○	○	○
Hockey	○	○	○	○
Judo	○	○	○	○
Karate	○	○	○	○
Lawn bowls	○	○	○	○
Motorsport-hill climb	○	○	○	○
Mountain bike (including cross country)	○	○	○	○

Netball - outdoor	◯	◯	◯	◯
Netball - indoor	◯	◯	◯	◯
Pool lifesaving	◯	◯	◯	◯
Powerlifting	◯	◯	◯	◯
Racquetball	◯	◯	◯	◯
Rowing-outdoor	◯	◯	◯	◯
Rowing-indoor	◯	◯	◯	◯
Rugby league	◯	◯	◯	◯
Rugby union	◯	◯	◯	◯
Sailing	◯	◯	◯	◯
Shooting - clay target	◯	◯	◯	◯
Shooting - smallbore and air rifle	◯	◯	◯	◯
Shooting - field rifle	◯	◯	◯	◯
Soccer	◯	◯	◯	◯
Softball	◯	◯	◯	◯
Squash	◯	◯	◯	◯
Swimming	◯	◯	◯	◯
Table tennis	◯	◯	◯	◯
Taekwondo	◯	◯	◯	◯
Tennis	◯	◯	◯	◯
Tenpin bowling	◯	◯	◯	◯
Touch football	◯	◯	◯	◯
Trail running	◯	◯	◯	◯
Volleyball	◯	◯	◯	◯

Did you compete in a sport at an elite level that was not in the above list? ◯ Yes ◯ No

---

please specify the other sport ________________________________

Do you have a second other sport that was not listed? ◯ Yes ◯ No

---

please specify the other sport ________________________________

Do you have a third other sport that was not listed? ◯ Yes ◯ No

---

please specify the other sport ________________________________

Do you have a fourth other sport that was not listed? ◯ Yes ◯ No

---

please specify the other sport ________________________________

Do you have a fifth other sport that was not listed?	◯ Yes<br>◯ No
please specify the other sport	__________________________________

At which level did you compete in elite competition for each of the other sports specified. Please select the HIGHEST level you ever completed in your career.

	State	National	International	Don't Know
[others_specify]	○	○	○	○
[others_2_specify]	○	○	○	○
[others_3_specify]	○	○	○	○
[others_4_specify]	○	○	○	○
[others_5_specify]	○	○	○	○

These following four questions are to get an idea of your regular participation in *organised, *leisure time, *occupational (work), or *transport (active transport) activity across your adult years.

Did you participate in *organised activity?

Please tick the box for each age group.
For example, if you know you regularly participated in organised activities during your 40s and 50s, but can't recall for your 20s and 30s, please tick the box "Yes" for 40-49 and 50-59. If you don't know for any of the age groups, please select "don't know"

	Yes	No	Don't know
18-29 years old	○	○	○
30-39 years old	○	○	○
40-49 years old	○	○	○
50-59 years old	○	○	○
60-69 years old	○	○	○
70 79 years old	○	○	○
80-89 years old	○	○	○
90-99 years old	○	○	○

*Organised activities are those that are governed by a set of rules and/or undertaken competitively at any level (e.g. competition tennis or golf).

Did you participate in *leisure-time activity?

	Yes	No	Don't know
18-29 years old	○	○	○
30-39 years old	○	○	○
40-49 years old	○	○	○
50-59 years old	○	○	○
60-69 years old	○	○	○
70-79 years old	○	○	○
80-89 years old	○	○	○
90-99 years old	○	○	○

*Leisure-time activities are other non-competitive activities (e.g. walking, gardening.)

Did you participate in *occupational activity?

	Yes	No	Don't know
18-29 years old	◯	◯	◯
30-39 years old	◯	◯	◯
40-49 years old	◯	◯	◯
50-59 years old	◯	◯	◯
60-69 years old	◯	◯	◯
70-79 years old	◯	◯	◯
80-89 years old	◯	◯	◯
90-99 years old	◯	◯	◯

*Occupational activities are non-sedentary activities undertaken at work (e.g. deliveries using cycling transport, housework such as vacuuming).

Did you participate in *transport activity?

	Yes	No	Don't know
18-29 years old	◯	◯	◯
30-39 years old	◯	◯	◯
40-49 years old	◯	◯	◯
50-59 years old	◯	◯	◯
60-69 years old	◯	◯	◯
70-79 years old	◯	◯	◯
80-89 years old	◯	◯	◯
90-99 years old	◯	◯	◯

*Transport activity is activity undertaken to get to/from somewhere (e.g. cycling to work).

In the last 12 months, how many times a week (on average) have you done the following training?

	Daily	5-6 times/week	3-4 times/week	1-2 times/week	0 times/week
Aerobic training	○	○	○	○	○
Anaerobic training	○	○	○	○	○
Resistance training	○	○	○	○	○

*Aerobic training increases heart rate and breathing for a sustained period of time, for example middle and long distance running/swimming/cycling, continuous dancing, and race walking.
*Anaerobic exercise increases the heart rate and breathing for a short period of time, for example short distance running/swimming/cycling, throwing sports, jumping sports.
**Resistance training uses an external resistance to assist with increasing muscle strength, tone, and size, for example using weights, tubes, body weight (e.g. pull-ups, push-ups).

Definitions based on information at
*https://www.medicinenet.com/aerobic_exercise/article.htm#what_is_aerobic_exercise
**https://www.emedicinehealth.com/strength_training/article_em.htm#how_does_resistance_exercise_work
.

In the last 12 months, how much time per week (on average) did you spend in the following training?

	< 1hour	1-2 hours/week	2-3 hours/week	3-4 hours/week	>4 hours/week
Aerobic training	◯	◯	◯	◯	◯
Anaerobic training	◯	◯	◯	◯	◯
Resistance training	◯	◯	◯	◯	◯

*Aerobic training increases heart rate and breathing for a sustained period of time, for example middle and long distance running/swimming/cycling, continuous dancing, and race walking.
*Anaerobic exercise increases the heart rate and breathing for a short period of time, for example short distance running/swimming/cycling, throwing sports, jumping sports.
**Resistance training uses an external resistance to assist with increasing muscle strength, tone, and size, for example using weights, tubes, body weight (e.g. pull-ups, push-ups).

Definitions based on information at
*https://www.medicinenet.com/aerobic_exercise/article.htm#what_is_aerobic_exercise
**https://www.emedicinehealth.com/strength_training/article_em.htm#how_does_resistance_exercise_work
.

Do you currently smoke tobacco?

◯ Yes. Daily
◯ Yes. Less than daily
◯ No. I am an ex-smoker
◯ No. I have never smoked

On average, how many cigarettes do you smoke each week?

◯ 1
◯ 2
◯ 3
◯ 4
◯ 5
◯ 6
◯ 7
◯ 8
◯ 9
◯ 10
◯ 11
◯ 12
◯ 13
◯ 14
◯ 15
◯ 16
◯ 17
◯ 18
◯ 19
◯ 20
◯ 21
◯ 22
◯ 23
◯ 24
◯ 25
◯ 26
◯ 27
◯ 28
◯ 29
◯ 30
◯ 31
◯ 32
◯ 33
◯ 34
◯ 35
◯ 36
◯ 37
◯ 38
◯ 39
◯ 40
◯ 41
◯ 42
◯ 43
◯ 44
◯ 45
◯ 46
◯ 47
◯ 48
◯ 49
◯ 50
◯ 51
◯ 52
◯ 53
◯ 54
◯ 55
◯ 56
◯ 57
◯ 58
◯ 59
◯ 60
◯ 61
◯ 62
◯ 63
◯ 64
◯ 65
◯ 66
◯ 67
◯ 68
◯ 69

◯ 70
◯ 71
◯ 72
◯ 73
◯ 74
◯ 75
◯ 76
◯ 77
◯ 78
◯ 79
◯ 80
◯ 81
◯ 82
◯ 83
◯ 84
◯ 85
◯ 86
◯ 87
◯ 88
◯ 89
◯ 90
◯ 91
◯ 92
◯ 93
◯ 94
◯ 95
◯ 96
◯ 97
◯ 98
◯ 99
◯ 100
◯ >100
◯ Don't know

How long have you been smoking for?

◯ < 1 year
◯ 1-5 years
◯ 6-10 years
◯ >10 years
◯ Don't know

How long did you smoke for?

◯ < 1 year
◯ 1-5 years
◯ 6-10 years
◯ >10 years
◯ Don't know

Do you currently drink alcohol at least once per week?

◯ Yes
◯ No

On average how many *standard drinks do you have per week?

◯ < 1
◯ 1-4
◯ 5-7
◯ 7-10
◯ 10-14
◯ >14
◯ Prefer not to say
◯ Don't know
(List of common standard drinks is shown below)

*List of approximate number of common standard drinks:
Full strength 4.8% alc. vol:
285 ml glass - 1.1 standard drinks
375 ml bottle or can - 1.4 standard drinks
Mid strength 3.5% alc. vol:
285 ml glass - 0.8 standard drinks
375 ml bottle or can - 1 standard drink
Low strength 2.7% alc. vol:
285 ml glass - 0.6 standard drinks
375 ml bottle or can - 0.8 standard drinks
Red wine: 150 ml average restaurant serving - 1.5 standard drinks
White wine OR Champagne: 150 ml average restaurant serving - 1.4 standard drinks
Spirits: 30 ml nip - 1 standard drink
Full strength pre-mix spirits: 375 ml can - 1.5 standard drinks

---

Did you ever drink regularly, that is, at least once a week?

- ◯ Yes
- ◯ No

On average how many *standard drinks did you have per week?

- ◯ 1-4
- ◯ 5-7
- ◯ 7-10
- ◯ 10-14
- ◯ >14
- ◯ Prefer not to say
- ◯ Don't know

(List of common standard drinks is shown below)

---

*List of approximate number of common standard drinks:
Full strength 4.8% alc. vol:
285 ml glass - 1.1 standard drinks
375 ml bottle or can - 1.4 standard drinks
Mid strength 3.5% alc. vol:
285 ml glass - 0.8 standard drinks
375 ml bottle or can - 1 standard drink
Low strength 2.7% alc. vol:
285 ml glass - 0.6 standard drinks
375 ml bottle or can - 0.8 standard drinks
Red wine: 150 ml average restaurant serving - 1.5 standard drinks
White wine OR Champagne: 150 ml average restaurant serving - 1.4 standard drinks
Spirits: 30 ml nip - 1 standard drink
Full strength pre-mix spirits: 375 ml can - 1.5 standard drinks

What is your current height in cm? (an approximate height is OK if you don't know exactly)

- < 140
- 140
- 141
- 142
- 143
- 144
- 145
- 146
- 147
- 148
- 149
- 150
- 151
- 152
- 153
- 154
- 155
- 156
- 157
- 158
- 159
- 160
- 161
- 162
- 163
- 164
- 165
- 166
- 167
- 168
- 169
- 170
- 171
- 172
- 173
- 174
- 175
- 176
- 177
- 178
- 179
- 180
- 181
- 182
- 183
- 184
- 185
- 186
- 187
- 188
- 189
- 190
- 191
- 192
- 193
- 194
- 195
- 196
- 197
- 198
- 199
- 200
- 201
- 202
- 203
- 204
- 205
- 206
- 207

◯ 208
◯ 209
◯ 210
◯ Don't know

Do you consider yourself to be an acceptable weight, underweight or overweight?	◯ acceptable weight<br>◯ underweight<br>◯ overweight<br>◯ don't Know

How is your [weight] condition treated?
(Tick all that apply)

☐ no treatment ☐ diet ☐ exercise ☐ natural/herbal therapy (please specify) ☐ supplements (please specify) ☐ other (please specify) ☐ Don't know

Please specify the natural/herbal therapy ________________________________

Please specify the supplements ________________________________

Please specify the treatment ________________________________

What is your current weight in kg? (an approximate weight is OK if you don't know exactly)

◯ < 40
◯ 40
◯ 41
◯ 42
◯ 43
◯ 44
◯ 45
◯ 46
◯ 47
◯ 48
◯ 49
◯ 50
◯ 51
◯ 52
◯ 53
◯ 54
◯ 55
◯ 56
◯ 57
◯ 58
◯ 59
◯ 60
◯ 61
◯ 62
◯ 63
◯ 64
◯ 65
◯ 66
◯ 67
◯ 68
◯ 69
◯ 70
◯ 71
◯ 72
◯ 73
◯ 74
◯ 75
◯ 76
◯ 77
◯ 78
◯ 79
◯ 80
◯ 81
◯ 82
◯ 83
◯ 84
◯ 85
◯ 86
◯ 87
◯ 88
◯ 89
◯ 90
◯ 91
◯ 92
◯ 93
◯ 94
◯ 95
◯ 96
◯ 97
◯ 98
◯ 99
◯ 100
◯ 101
◯ 102
◯ 103
◯ 104
◯ 105
◯ 106
◯ 107

○ 108
○ 109
○ 110
○ 111
○ 112
○ 113
○ 114
○ 115
○ 116
○ 117
○ 118
○ 119
○ 120
○ 121
○ 122
○ 123
○ 124
○ 125
○ 126
○ 127
○ 128
○ 129
○ 130
○ 131
○ 132
○ 133
○ 134
○ 135
○ 136
○ 137
○ 138
○ 139
○ 140
○ 141
○ 142
○ 143
○ 144
○ 145
○ 146
○ 147
○ 148
○ 149
○ 150
○ 151
○ 152
○ 153
○ 154
○ 155
○ 156
○ 157
○ 158
○ 159
○ 160
○ 161
○ 162
○ 163
○ 164
○ 165
○ 166
○ 167
○ 168
○ 169
○ 170
○ 171
○ 172
○ 173
○ 174
○ 175
○ 176
○ 177
○ 178

◯ 179
◯ 180
◯ 181
◯ 182
◯ 183
◯ 184
◯ 185
◯ 186
◯ 187
◯ 188
◯ 189
◯ 190
◯ 191
◯ 192
◯ 193
◯ 194
◯ 195
◯ 196
◯ 197
◯ 198
◯ 199
◯ 200
◯ Don't know

Are either of your parents, or any of brothers or sisters overweight or obese?

◯ Yes
◯ No
◯ Don't know

Including any conditions which can be controlled with medication, have you ever been told by a doctor (GP or specialist) or nurse that you have any heart or circulatory conditions?

◯ Yes
◯ No
◯ Don't know

What are the names of this/these condition/s?
(Tick all that apply)

☐ Angina
☐ Fluid problems/fluid retention/oedema
☐ Haemorrhoids
☐ Hardening of the arteries/atherosclerosis/arteriosclerosis
☐ Heart attack
☐ Heart failure
☐ Heart murmur/heart valve disorder
☐ High blood pressure/ hypertension
☐ High cholesterol
☐ Low blood pressure/hypotension
☐ Rapid or irregular heartbeats/tachycardia/palpitations
☐ Rheumatic heart disease
☐ Stroke (including after effects of stroke)
☐ Varicose veins
☐ Other (please specify)
☐ Don't know

---

please specify the other heart condition

____

Do you have another heart of circulatory condition needs to be specified?

◯ Yes ◯ No

---

please specify the second heart condition

____

Do you have another heart of circulatory condition needs to be specified?

◯ Yes ◯ No

please specify the third heart condition

____

At what age were you first told you had Rheumatic heart disease?

- ◯ 0
- ◯ 1
- ◯ 2
- ◯ 3
- ◯ 4
- ◯ 5
- ◯ 6
- ◯ 7
- ◯ 8
- ◯ 9
- ◯ 10
- ◯ 11
- ◯ 12
- ◯ 13
- ◯ 14
- ◯ 15
- ◯ 16
- ◯ 17
- ◯ 18
- ◯ 19
- ◯ 20
- ◯ 21
- ◯ 22
- ◯ 23
- ◯ 24
- ◯ 25
- ◯ 26
- ◯ 27
- ◯ 28
- ◯ 29
- ◯ 30
- ◯ 31
- ◯ 32
- ◯ 33
- ◯ 34
- ◯ 35
- ◯ 36
- ◯ 37
- ◯ 38
- ◯ 39
- ◯ 40
- ◯ 41
- ◯ 42
- ◯ 43
- ◯ 44
- ◯ 45
- ◯ 46
- ◯ 47
- ◯ 48
- ◯ 49
- ◯ 50
- ◯ 51
- ◯ 52
- ◯ 53
- ◯ 54
- ◯ 55
- ◯ 56
- ◯ 57
- ◯ 58
- ◯ 59
- ◯ 60
- ◯ 61
- ◯ 62
- ◯ 63
- ◯ 64
- ◯ 65
- ◯ 66
- ◯ 67
- ◯ 68

- ◯ 69
- ◯ 70
- ◯ 71
- ◯ 72
- ◯ 73
- ◯ 74
- ◯ 75
- ◯ 76
- ◯ 77
- ◯ 78
- ◯ 79
- ◯ 80
- ◯ 81
- ◯ 82
- ◯ 83
- ◯ 84
- ◯ 85
- ◯ 86
- ◯ 87
- ◯ 88
- ◯ 89
- ◯ 90
- ◯ 91
- ◯ 92
- ◯ 93
- ◯ 94
- ◯ 95
- ◯ 96
- ◯ 97
- ◯ 98
- ◯ 99
- ◯ Don't know

At what age were you first told you had heart attack?

- ◯ 0
- ◯ 1
- ◯ 2
- ◯ 3
- ◯ 4
- ◯ 5
- ◯ 6
- ◯ 7
- ◯ 8
- ◯ 9
- ◯ 10
- ◯ 11
- ◯ 12
- ◯ 13
- ◯ 14
- ◯ 15
- ◯ 16
- ◯ 17
- ◯ 18
- ◯ 19
- ◯ 20
- ◯ 21
- ◯ 22
- ◯ 23
- ◯ 24
- ◯ 25
- ◯ 26
- ◯ 27
- ◯ 28
- ◯ 29
- ◯ 30
- ◯ 31
- ◯ 32
- ◯ 33
- ◯ 34
- ◯ 35
- ◯ 36
- ◯ 37
- ◯ 38
- ◯ 39
- ◯ 40
- ◯ 41
- ◯ 42
- ◯ 43
- ◯ 44
- ◯ 45
- ◯ 46
- ◯ 47
- ◯ 48
- ◯ 49
- ◯ 50
- ◯ 51
- ◯ 52
- ◯ 53
- ◯ 54
- ◯ 55
- ◯ 56
- ◯ 57
- ◯ 58
- ◯ 59
- ◯ 60
- ◯ 61
- ◯ 62
- ◯ 63
- ◯ 64
- ◯ 65
- ◯ 66
- ◯ 67
- ◯ 68

- ◯ 69
- ◯ 70
- ◯ 71
- ◯ 72
- ◯ 73
- ◯ 74
- ◯ 75
- ◯ 76
- ◯ 77
- ◯ 78
- ◯ 79
- ◯ 80
- ◯ 81
- ◯ 82
- ◯ 83
- ◯ 84
- ◯ 85
- ◯ 86
- ◯ 87
- ◯ 88
- ◯ 89
- ◯ 90
- ◯ 91
- ◯ 92
- ◯ 93
- ◯ 94
- ◯ 95
- ◯ 96
- ◯ 97
- ◯ 98
- ◯ 99
- ◯ Don't know

At what age were you first told you had heart failure?

- 0
- 1
- 2
- 3
- 4
- 5
- 6
- 7
- 8
- 9
- 10
- 11
- 12
- 13
- 14
- 15
- 16
- 17
- 18
- 19
- 20
- 21
- 22
- 23
- 24
- 25
- 26
- 27
- 28
- 29
- 30
- 31
- 32
- 33
- 34
- 35
- 36
- 37
- 38
- 39
- 40
- 41
- 42
- 43
- 44
- 45
- 46
- 47
- 48
- 49
- 50
- 51
- 52
- 53
- 54
- 55
- 56
- 57
- 58
- 59
- 60
- 61
- 62
- 63
- 64
- 65
- 66
- 67
- 68

- ◯ 69
- ◯ 70
- ◯ 71
- ◯ 72
- ◯ 73
- ◯ 74
- ◯ 75
- ◯ 76
- ◯ 77
- ◯ 78
- ◯ 79
- ◯ 80
- ◯ 81
- ◯ 82
- ◯ 83
- ◯ 84
- ◯ 85
- ◯ 86
- ◯ 87
- ◯ 88
- ◯ 89
- ◯ 90
- ◯ 91
- ◯ 92
- ◯ 93
- ◯ 94
- ◯ 95
- ◯ 96
- ◯ 97
- ◯ 98
- ◯ 99
- ◯ Don't know

At what age were you first told you had stroke (including after effects of stoke)?

◯ 0
◯ 1
◯ 2
◯ 3
◯ 4
◯ 5
◯ 6
◯ 7
◯ 8
◯ 9
◯ 10
◯ 11
◯ 12
◯ 13
◯ 14
◯ 15
◯ 16
◯ 17
◯ 18
◯ 19
◯ 20
◯ 21
◯ 22
◯ 23
◯ 24
◯ 25
◯ 26
◯ 27
◯ 28
◯ 29
◯ 30
◯ 31
◯ 32
◯ 33
◯ 34
◯ 35
◯ 36
◯ 37
◯ 38
◯ 39
◯ 40
◯ 41
◯ 42
◯ 43
◯ 44
◯ 45
◯ 46
◯ 47
◯ 48
◯ 49
◯ 50
◯ 51
◯ 52
◯ 53
◯ 54
◯ 55
◯ 56
◯ 57
◯ 58
◯ 59
◯ 60
◯ 61
◯ 62
◯ 63
◯ 64
◯ 65
◯ 66
◯ 67
◯ 68

- ◯ 69
- ◯ 70
- ◯ 71
- ◯ 72
- ◯ 73
- ◯ 74
- ◯ 75
- ◯ 76
- ◯ 77
- ◯ 78
- ◯ 79
- ◯ 80
- ◯ 81
- ◯ 82
- ◯ 83
- ◯ 84
- ◯ 85
- ◯ 86
- ◯ 87
- ◯ 88
- ◯ 89
- ◯ 90
- ◯ 91
- ◯ 92
- ◯ 93
- ◯ 94
- ◯ 95
- ◯ 96
- ◯ 97
- ◯ 98
- ◯ 99
- ◯ Don't know

At what age were you first told you had angina?

- ◯ 0
- ◯ 1
- ◯ 2
- ◯ 3
- ◯ 4
- ◯ 5
- ◯ 6
- ◯ 7
- ◯ 8
- ◯ 9
- ◯ 10
- ◯ 11
- ◯ 12
- ◯ 13
- ◯ 14
- ◯ 15
- ◯ 16
- ◯ 17
- ◯ 18
- ◯ 19
- ◯ 20
- ◯ 21
- ◯ 22
- ◯ 23
- ◯ 24
- ◯ 25
- ◯ 26
- ◯ 27
- ◯ 28
- ◯ 29
- ◯ 30
- ◯ 31
- ◯ 32
- ◯ 33
- ◯ 34
- ◯ 35
- ◯ 36
- ◯ 37
- ◯ 38
- ◯ 39
- ◯ 40
- ◯ 41
- ◯ 42
- ◯ 43
- ◯ 44
- ◯ 45
- ◯ 46
- ◯ 47
- ◯ 48
- ◯ 49
- ◯ 50
- ◯ 51
- ◯ 52
- ◯ 53
- ◯ 54
- ◯ 55
- ◯ 56
- ◯ 57
- ◯ 58
- ◯ 59
- ◯ 60
- ◯ 61
- ◯ 62
- ◯ 63
- ◯ 64
- ◯ 65
- ◯ 66
- ◯ 67
- ◯ 68

◯ 69
◯ 70
◯ 71
◯ 72
◯ 73
◯ 74
◯ 75
◯ 76
◯ 77
◯ 78
◯ 79
◯ 80
◯ 81
◯ 82
◯ 83
◯ 84
◯ 85
◯ 86
◯ 87
◯ 88
◯ 89
◯ 90
◯ 91
◯ 92
◯ 93
◯ 94
◯ 95
◯ 96
◯ 97
◯ 98
◯ 99
◯ Don't know

At what age were you first told you had high blood pressure/hypertension?

◯ 0
◯ 1
◯ 2
◯ 3
◯ 4
◯ 5
◯ 6
◯ 7
◯ 8
◯ 9
◯ 10
◯ 11
◯ 12
◯ 13
◯ 14
◯ 15
◯ 16
◯ 17
◯ 18
◯ 19
◯ 20
◯ 21
◯ 22
◯ 23
◯ 24
◯ 25
◯ 26
◯ 27
◯ 28
◯ 29
◯ 30
◯ 31
◯ 32
◯ 33
◯ 34
◯ 35
◯ 36
◯ 37
◯ 38
◯ 39
◯ 40
◯ 41
◯ 42
◯ 43
◯ 44
◯ 45
◯ 46
◯ 47
◯ 48
◯ 49
◯ 50
◯ 51
◯ 52
◯ 53
◯ 54
◯ 55
◯ 56
◯ 57
◯ 58
◯ 59
◯ 60
◯ 61
◯ 62
◯ 63
◯ 64
◯ 65
◯ 66
◯ 67
◯ 68

◯ 69
◯ 70
◯ 71
◯ 72
◯ 73
◯ 74
◯ 75
◯ 76
◯ 77
◯ 78
◯ 79
◯ 80
◯ 81
◯ 82
◯ 83
◯ 84
◯ 85
◯ 86
◯ 87
◯ 88
◯ 89
◯ 90
◯ 91
◯ 92
◯ 93
◯ 94
◯ 95
◯ 96
◯ 97
◯ 98
◯ 99
◯ Don't know

At what age were you first told you had low blood pressure/hypotension

○ 0
○ 1
○ 2
○ 3
○ 4
○ 5
○ 6
○ 7
○ 8
○ 9
○ 10
○ 11
○ 12
○ 13
○ 14
○ 15
○ 16
○ 17
○ 18
○ 19
○ 20
○ 21
○ 22
○ 23
○ 24
○ 25
○ 26
○ 27
○ 28
○ 29
○ 30
○ 31
○ 32
○ 33
○ 34
○ 35
○ 36
○ 37
○ 38
○ 39
○ 40
○ 41
○ 42
○ 43
○ 44
○ 45
○ 46
○ 47
○ 48
○ 49
○ 50
○ 51
○ 52
○ 53
○ 54
○ 55
○ 56
○ 57
○ 58
○ 59
○ 60
○ 61
○ 62
○ 63
○ 64
○ 65
○ 66
○ 67
○ 68

- ◯ 69
- ◯ 70
- ◯ 71
- ◯ 72
- ◯ 73
- ◯ 74
- ◯ 75
- ◯ 76
- ◯ 77
- ◯ 78
- ◯ 79
- ◯ 80
- ◯ 81
- ◯ 82
- ◯ 83
- ◯ 84
- ◯ 85
- ◯ 86
- ◯ 87
- ◯ 88
- ◯ 89
- ◯ 90
- ◯ 91
- ◯ 92
- ◯ 93
- ◯ 94
- ◯ 95
- ◯ 96
- ◯ 97
- ◯ 98
- ◯ 99
- ◯ Don't know

At what age were you first told you had hardening of the arteries/atherosclerosis/arteriosclerosis?

◯ 0
◯ 1
◯ 2
◯ 3
◯ 4
◯ 5
◯ 6
◯ 7
◯ 8
◯ 9
◯ 10
◯ 11
◯ 12
◯ 13
◯ 14
◯ 15
◯ 16
◯ 17
◯ 18
◯ 19
◯ 20
◯ 21
◯ 22
◯ 23
◯ 24
◯ 25
◯ 26
◯ 27
◯ 28
◯ 29
◯ 30
◯ 31
◯ 32
◯ 33
◯ 34
◯ 35
◯ 36
◯ 37
◯ 38
◯ 39
◯ 40
◯ 41
◯ 42
◯ 43
◯ 44
◯ 45
◯ 46
◯ 47
◯ 48
◯ 49
◯ 50
◯ 51
◯ 52
◯ 53
◯ 54
◯ 55
◯ 56
◯ 57
◯ 58
◯ 59
◯ 60
◯ 61
◯ 62
◯ 63
◯ 64
◯ 65
◯ 66
◯ 67
◯ 68

◯ 69
◯ 70
◯ 71
◯ 72
◯ 73
◯ 74
◯ 75
◯ 76
◯ 77
◯ 78
◯ 79
◯ 80
◯ 81
◯ 82
◯ 83
◯ 84
◯ 85
◯ 86
◯ 87
◯ 88
◯ 89
◯ 90
◯ 91
◯ 92
◯ 93
◯ 94
◯ 95
◯ 96
◯ 97
◯ 98
◯ 99
◯ Don't know

At what age were you first told you had fluid problems/fluid retention/oedema?

◯ 0
◯ 1
◯ 2
◯ 3
◯ 4
◯ 5
◯ 6
◯ 7
◯ 8
◯ 9
◯ 10
◯ 11
◯ 12
◯ 13
◯ 14
◯ 15
◯ 16
◯ 17
◯ 18
◯ 19
◯ 20
◯ 21
◯ 22
◯ 23
◯ 24
◯ 25
◯ 26
◯ 27
◯ 28
◯ 29
◯ 30
◯ 31
◯ 32
◯ 33
◯ 34
◯ 35
◯ 36
◯ 37
◯ 38
◯ 39
◯ 40
◯ 41
◯ 42
◯ 43
◯ 44
◯ 45
◯ 46
◯ 47
◯ 48
◯ 49
◯ 50
◯ 51
◯ 52
◯ 53
◯ 54
◯ 55
◯ 56
◯ 57
◯ 58
◯ 59
◯ 60
◯ 61
◯ 62
◯ 63
◯ 64
◯ 65
◯ 66
◯ 67
◯ 68

◯ 69
◯ 70
◯ 71
◯ 72
◯ 73
◯ 74
◯ 75
◯ 76
◯ 77
◯ 78
◯ 79
◯ 80
◯ 81
◯ 82
◯ 83
◯ 84
◯ 85
◯ 86
◯ 87
◯ 88
◯ 89
◯ 90
◯ 91
◯ 92
◯ 93
◯ 94
◯ 95
◯ 96
◯ 97
◯ 98
◯ 99
◯ Don't know

At what age were you first told you had high cholesterol?

- ◯ 0
- ◯ 1
- ◯ 2
- ◯ 3
- ◯ 4
- ◯ 5
- ◯ 6
- ◯ 7
- ◯ 8
- ◯ 9
- ◯ 10
- ◯ 11
- ◯ 12
- ◯ 13
- ◯ 14
- ◯ 15
- ◯ 16
- ◯ 17
- ◯ 18
- ◯ 19
- ◯ 20
- ◯ 21
- ◯ 22
- ◯ 23
- ◯ 24
- ◯ 25
- ◯ 26
- ◯ 27
- ◯ 28
- ◯ 29
- ◯ 30
- ◯ 31
- ◯ 32
- ◯ 33
- ◯ 34
- ◯ 35
- ◯ 36
- ◯ 37
- ◯ 38
- ◯ 39
- ◯ 40
- ◯ 41
- ◯ 42
- ◯ 43
- ◯ 44
- ◯ 45
- ◯ 46
- ◯ 47
- ◯ 48
- ◯ 49
- ◯ 50
- ◯ 51
- ◯ 52
- ◯ 53
- ◯ 54
- ◯ 55
- ◯ 56
- ◯ 57
- ◯ 58
- ◯ 59
- ◯ 60
- ◯ 61
- ◯ 62
- ◯ 63
- ◯ 64
- ◯ 65
- ◯ 66
- ◯ 67
- ◯ 68

◯ 69
◯ 70
◯ 71
◯ 72
◯ 73
◯ 74
◯ 75
◯ 76
◯ 77
◯ 78
◯ 79
◯ 80
◯ 81
◯ 82
◯ 83
◯ 84
◯ 85
◯ 86
◯ 87
◯ 88
◯ 89
◯ 90
◯ 91
◯ 92
◯ 93
◯ 94
◯ 95
◯ 96
◯ 97
◯ 98
◯ 99
◯ Don't know

At what age were you first told you had rapid or irregular heartbeats/tachycardia/palpitations?

◯ 0
◯ 1
◯ 2
◯ 3
◯ 4
◯ 5
◯ 6
◯ 7
◯ 8
◯ 9
◯ 10
◯ 11
◯ 12
◯ 13
◯ 14
◯ 15
◯ 16
◯ 17
◯ 18
◯ 19
◯ 20
◯ 21
◯ 22
◯ 23
◯ 24
◯ 25
◯ 26
◯ 27
◯ 28
◯ 29
◯ 30
◯ 31
◯ 32
◯ 33
◯ 34
◯ 35
◯ 36
◯ 37
◯ 38
◯ 39
◯ 40
◯ 41
◯ 42
◯ 43
◯ 44
◯ 45
◯ 46
◯ 47
◯ 48
◯ 49
◯ 50
◯ 51
◯ 52
◯ 53
◯ 54
◯ 55
◯ 56
◯ 57
◯ 58
◯ 59
◯ 60
◯ 61
◯ 62
◯ 63
◯ 64
◯ 65
◯ 66
◯ 67
◯ 68

- ◯ 69
- ◯ 70
- ◯ 71
- ◯ 72
- ◯ 73
- ◯ 74
- ◯ 75
- ◯ 76
- ◯ 77
- ◯ 78
- ◯ 79
- ◯ 80
- ◯ 81
- ◯ 82
- ◯ 83
- ◯ 84
- ◯ 85
- ◯ 86
- ◯ 87
- ◯ 88
- ◯ 89
- ◯ 90
- ◯ 91
- ◯ 92
- ◯ 93
- ◯ 94
- ◯ 95
- ◯ 96
- ◯ 97
- ◯ 98
- ◯ 99
- ◯ Don't know

At what age were you first told you had heart murmur/heart valve disorder?

◯ 0
◯ 1
◯ 2
◯ 3
◯ 4
◯ 5
◯ 6
◯ 7
◯ 8
◯ 9
◯ 10
◯ 11
◯ 12
◯ 13
◯ 14
◯ 15
◯ 16
◯ 17
◯ 18
◯ 19
◯ 20
◯ 21
◯ 22
◯ 23
◯ 24
◯ 25
◯ 26
◯ 27
◯ 28
◯ 29
◯ 30
◯ 31
◯ 32
◯ 33
◯ 34
◯ 35
◯ 36
◯ 37
◯ 38
◯ 39
◯ 40
◯ 41
◯ 42
◯ 43
◯ 44
◯ 45
◯ 46
◯ 47
◯ 48
◯ 49
◯ 50
◯ 51
◯ 52
◯ 53
◯ 54
◯ 55
◯ 56
◯ 57
◯ 58
◯ 59
◯ 60
◯ 61
◯ 62
◯ 63
◯ 64
◯ 65
◯ 66
◯ 67
◯ 68

◯ 69
◯ 70
◯ 71
◯ 72
◯ 73
◯ 74
◯ 75
◯ 76
◯ 77
◯ 78
◯ 79
◯ 80
◯ 81
◯ 82
◯ 83
◯ 84
◯ 85
◯ 86
◯ 87
◯ 88
◯ 89
◯ 90
◯ 91
◯ 92
◯ 93
◯ 94
◯ 95
◯ 96
◯ 97
◯ 98
◯ 99
◯ Don't know

At what age were you first told you had haemorrhoids?

◯ 0
◯ 1
◯ 2
◯ 3
◯ 4
◯ 5
◯ 6
◯ 7
◯ 8
◯ 9
◯ 10
◯ 11
◯ 12
◯ 13
◯ 14
◯ 15
◯ 16
◯ 17
◯ 18
◯ 19
◯ 20
◯ 21
◯ 22
◯ 23
◯ 24
◯ 25
◯ 26
◯ 27
◯ 28
◯ 29
◯ 30
◯ 31
◯ 32
◯ 33
◯ 34
◯ 35
◯ 36
◯ 37
◯ 38
◯ 39
◯ 40
◯ 41
◯ 42
◯ 43
◯ 44
◯ 45
◯ 46
◯ 47
◯ 48
◯ 49
◯ 50
◯ 51
◯ 52
◯ 53
◯ 54
◯ 55
◯ 56
◯ 57
◯ 58
◯ 59
◯ 60
◯ 61
◯ 62
◯ 63
◯ 64
◯ 65
◯ 66
◯ 67
◯ 68

- ◯ 69
- ◯ 70
- ◯ 71
- ◯ 72
- ◯ 73
- ◯ 74
- ◯ 75
- ◯ 76
- ◯ 77
- ◯ 78
- ◯ 79
- ◯ 80
- ◯ 81
- ◯ 82
- ◯ 83
- ◯ 84
- ◯ 85
- ◯ 86
- ◯ 87
- ◯ 88
- ◯ 89
- ◯ 90
- ◯ 91
- ◯ 92
- ◯ 93
- ◯ 94
- ◯ 95
- ◯ 96
- ◯ 97
- ◯ 98
- ◯ 99
- ◯ Don't know

At what age were you first told you had varicose veins?

◯ 0
◯ 1
◯ 2
◯ 3
◯ 4
◯ 5
◯ 6
◯ 7
◯ 8
◯ 9
◯ 10
◯ 11
◯ 12
◯ 13
◯ 14
◯ 15
◯ 16
◯ 17
◯ 18
◯ 19
◯ 20
◯ 21
◯ 22
◯ 23
◯ 24
◯ 25
◯ 26
◯ 27
◯ 28
◯ 29
◯ 30
◯ 31
◯ 32
◯ 33
◯ 34
◯ 35
◯ 36
◯ 37
◯ 38
◯ 39
◯ 40
◯ 41
◯ 42
◯ 43
◯ 44
◯ 45
◯ 46
◯ 47
◯ 48
◯ 49
◯ 50
◯ 51
◯ 52
◯ 53
◯ 54
◯ 55
◯ 56
◯ 57
◯ 58
◯ 59
◯ 60
◯ 61
◯ 62
◯ 63
◯ 64
◯ 65
◯ 66
◯ 67
◯ 68

- ◯ 69
- ◯ 70
- ◯ 71
- ◯ 72
- ◯ 73
- ◯ 74
- ◯ 75
- ◯ 76
- ◯ 77
- ◯ 78
- ◯ 79
- ◯ 80
- ◯ 81
- ◯ 82
- ◯ 83
- ◯ 84
- ◯ 85
- ◯ 86
- ◯ 87
- ◯ 88
- ◯ 89
- ◯ 90
- ◯ 91
- ◯ 92
- ◯ 93
- ◯ 94
- ◯ 95
- ◯ 96
- ◯ 97
- ◯ 98
- ◯ 99
- ◯ Don't know

At what age were you first told you had [heart_others]?

◯ 0
◯ 1
◯ 2
◯ 3
◯ 4
◯ 5
◯ 6
◯ 7
◯ 8
◯ 9
◯ 10
◯ 11
◯ 12
◯ 13
◯ 14
◯ 15
◯ 16
◯ 17
◯ 18
◯ 19
◯ 20
◯ 21
◯ 22
◯ 23
◯ 24
◯ 25
◯ 26
◯ 27
◯ 28
◯ 29
◯ 30
◯ 31
◯ 32
◯ 33
◯ 34
◯ 35
◯ 36
◯ 37
◯ 38
◯ 39
◯ 40
◯ 41
◯ 42
◯ 43
◯ 44
◯ 45
◯ 46
◯ 47
◯ 48
◯ 49
◯ 50
◯ 51
◯ 52
◯ 53
◯ 54
◯ 55
◯ 56
◯ 57
◯ 58
◯ 59
◯ 60
◯ 61
◯ 62
◯ 63
◯ 64
◯ 65
◯ 66
◯ 67
◯ 68

- ◯ 69
- ◯ 70
- ◯ 71
- ◯ 72
- ◯ 73
- ◯ 74
- ◯ 75
- ◯ 76
- ◯ 77
- ◯ 78
- ◯ 79
- ◯ 80
- ◯ 81
- ◯ 82
- ◯ 83
- ◯ 84
- ◯ 85
- ◯ 86
- ◯ 87
- ◯ 88
- ◯ 89
- ◯ 90
- ◯ 91
- ◯ 92
- ◯ 93
- ◯ 94
- ◯ 95
- ◯ 96
- ◯ 97
- ◯ 98
- ◯ 99
- ◯ Don't know

At what age were you first told you had [heart_others_2]?

◯ 0
◯ 1
◯ 2
◯ 3
◯ 4
◯ 5
◯ 6
◯ 7
◯ 8
◯ 9
◯ 10
◯ 11
◯ 12
◯ 13
◯ 14
◯ 15
◯ 16
◯ 17
◯ 18
◯ 19
◯ 20
◯ 21
◯ 22
◯ 23
◯ 24
◯ 25
◯ 26
◯ 27
◯ 28
◯ 29
◯ 30
◯ 31
◯ 32
◯ 33
◯ 34
◯ 35
◯ 36
◯ 37
◯ 38
◯ 39
◯ 40
◯ 41
◯ 42
◯ 43
◯ 44
◯ 45
◯ 46
◯ 47
◯ 48
◯ 49
◯ 50
◯ 51
◯ 52
◯ 53
◯ 54
◯ 55
◯ 56
◯ 57
◯ 58
◯ 59
◯ 60
◯ 61
◯ 62
◯ 63
◯ 64
◯ 65
◯ 66
◯ 67
◯ 68

- ◯ 69
- ◯ 70
- ◯ 71
- ◯ 72
- ◯ 73
- ◯ 74
- ◯ 75
- ◯ 76
- ◯ 77
- ◯ 78
- ◯ 79
- ◯ 80
- ◯ 81
- ◯ 82
- ◯ 83
- ◯ 84
- ◯ 85
- ◯ 86
- ◯ 87
- ◯ 88
- ◯ 89
- ◯ 90
- ◯ 91
- ◯ 92
- ◯ 93
- ◯ 94
- ◯ 95
- ◯ 96
- ◯ 97
- ◯ 98
- ◯ 99
- ◯ Don't know

At what age were you first told you had [heart_others_3]?

◯ 0
◯ 1
◯ 2
◯ 3
◯ 4
◯ 5
◯ 6
◯ 7
◯ 8
◯ 9
◯ 10
◯ 11
◯ 12
◯ 13
◯ 14
◯ 15
◯ 16
◯ 17
◯ 18
◯ 19
◯ 20
◯ 21
◯ 22
◯ 23
◯ 24
◯ 25
◯ 26
◯ 27
◯ 28
◯ 29
◯ 30
◯ 31
◯ 32
◯ 33
◯ 34
◯ 35
◯ 36
◯ 37
◯ 38
◯ 39
◯ 40
◯ 41
◯ 42
◯ 43
◯ 44
◯ 45
◯ 46
◯ 47
◯ 48
◯ 49
◯ 50
◯ 51
◯ 52
◯ 53
◯ 54
◯ 55
◯ 56
◯ 57
◯ 58
◯ 59
◯ 60
◯ 61
◯ 62
◯ 63
◯ 64
◯ 65
◯ 66
◯ 67
◯ 68

◯ 69
◯ 70
◯ 71
◯ 72
◯ 73
◯ 74
◯ 75
◯ 76
◯ 77
◯ 78
◯ 79
◯ 80
◯ 81
◯ 82
◯ 83
◯ 84
◯ 85
◯ 86
◯ 87
◯ 88
◯ 89
◯ 90
◯ 91
◯ 92
◯ 93
◯ 94
◯ 95
◯ 96
◯ 97
◯ 98
◯ 99
◯ Don't know

At what age were you first told you had this condition?

◯ 0
◯ 1
◯ 2
◯ 3
◯ 4
◯ 5
◯ 6
◯ 7
◯ 8
◯ 9
◯ 10
◯ 11
◯ 12
◯ 13
◯ 14
◯ 15
◯ 16
◯ 17
◯ 18
◯ 19
◯ 20
◯ 21
◯ 22
◯ 23
◯ 24
◯ 25
◯ 26
◯ 27
◯ 28
◯ 29
◯ 30
◯ 31
◯ 32
◯ 33
◯ 34
◯ 35
◯ 36
◯ 37
◯ 38
◯ 39
◯ 40
◯ 41
◯ 42
◯ 43
◯ 44
◯ 45
◯ 46
◯ 47
◯ 48
◯ 49
◯ 50
◯ 51
◯ 52
◯ 53
◯ 54
◯ 55
◯ 56
◯ 57
◯ 58
◯ 59
◯ 60
◯ 61
◯ 62
◯ 63
◯ 64
◯ 65
◯ 66
◯ 67
◯ 68

	◯ 69<br>◯ 70<br>◯ 71<br>◯ 72<br>◯ 73<br>◯ 74<br>◯ 75<br>◯ 76<br>◯ 77<br>◯ 78<br>◯ 79<br>◯ 80<br>◯ 81<br>◯ 82<br>◯ 83<br>◯ 84<br>◯ 85<br>◯ 86<br>◯ 87<br>◯ 88<br>◯ 89<br>◯ 90<br>◯ 91<br>◯ 92<br>◯ 93<br>◯ 94<br>◯ 95<br>◯ 96<br>◯ 97<br>◯ 98<br>◯ 99<br>◯ Don't know
Do you still have rheumatic heart disease?	◯ Yes<br>◯ No<br>◯ Don't know
Do you still have heat attack?	◯ Yes<br>◯ No<br>◯ Don't know
Do you still have heat failure?	◯ Yes<br>◯ No<br>◯ Don't know
Do you still have stroke (including after effects of stroke)?	◯ Yes<br>◯ No<br>◯ Don't know
Do you still have angina?	◯ Yes<br>◯ No<br>◯ Don't know
Do you still have high blood pressure/hypertension?	◯ Yes<br>◯ No<br>◯ Don't know
Do you still have low blood pressure/hypotension?	◯ Yes<br>◯ No<br>◯ Don't know

Do you still have hardening of the arteries/atherosclerosis/arteriosclerosis?	◯ Yes<br>◯ No<br>◯ Don't know
Do you still have fluid problems/fluid retention/oedema?	◯ Yes<br>◯ No<br>◯ Don't know
Do you still have high cholesterol?	◯ Yes<br>◯ No<br>◯ Don't know
Do you still have rapid or irregular heartbeats/tachycardia/palpitations?	◯ Yes<br>◯ No<br>◯ Don't know
Do you still have heart murmur/heart valve disorder	◯ Yes<br>◯ No<br>◯ Don't know
Do you still have haemorrhoids?	◯ Yes<br>◯ No<br>◯ Don't know
Do you still have varicose veins?	◯ Yes<br>◯ No<br>◯ Don't know
Do you still have [heart_others]?	◯ Yes<br>◯ No<br>◯ Don't know
Do you still have [heart_others_2]?	◯ Yes<br>◯ No<br>◯ Don't know
Do you still have [heart_others_3]?	◯ Yes<br>◯ No<br>◯ Don't know
Do you still have this condition?	◯ Yes<br>◯ No<br>◯ Don't know

How is rheumatic heart disease treated?
(Tick all that apply)

☐ no treatment ☐ diet ☐ exercise ☐ medication (please specify) ☐ natural/herbal therapy (please specify) ☐ weight management ☐ supplements (please specify) ☐ other (please specify)
☐ Don't know

please specify the medication	__________________________________
Please specify the natural/herbal therapy	__________________________________

please specify the supplements

____

please specify the treatment

____

How is heart attack treated?
(Tick all that apply)

☐ no treatment ☐ diet ☐ exercise ☐ medication (please specify) ☐ natural/herbal therapy (please specify) ☐ weight management ☐ supplements (please specify) ☐ other (please specify) ☐ Don't know

please specify the medication

____

please specify the natural/herbal therapy

____

please specify the supplements

____

please specify the treatment

____

How is heart failure treated?

☐ no treatment ☐ diet
☐ exercise ☐ medication (please specify)
☐ natural/herbal therapy (please specify)
☐ weight management
☐ supplements (please specify)
☐ other (please specify)
☐ Don't know
(Tick all that apply)

please specify the medication

____

please specify the natural/herbal therapy

____

please specify the supplements

____

please specify the treatment

____

How is stroke (including after effects of stroke) treated?
(Tick all that apply)

☐ no treatment ☐ diet ☐ exercise ☐ medication (please specify) ☐ natural/herbal therapy (please specify) ☐ weight management ☐ supplements (please specify) ☐ other (please specify)
☐ Don't know

please specify the medication ________________________________

please specify the natural/herbal therapy ________________________________

please specify the supplements ________________________________

please specify the treatment ________________________________

How is angina treated?
(Tick all that apply)

☐ no treatment ☐ diet ☐ exercise ☐ medication (please specify) ☐ natural/herbal therapy (please specify) ☐ weight management ☐ supplements (please specify) ☐ other (please specify)
☐ Don't know

please specify the medication ________________________________

please specify the natural/herbal therapy ________________________________

please specify the supplements ________________________________

please specify the treatment ________________________________

How is high blood pressure/hypertension treated?
(Tick all that apply)

☐ no treatment ☐ diet ☐ exercise ☐ medication (please specify) ☐ natural/herbal therapy (please specify) ☐ weight management ☐ supplements (please specify) ☐ other (please specify)
☐ Don't know

please specify the medication ________________________________

please specify the natural/herbal therapy ________________________________

please specify the supplements ________________________________

please specify the treatment ________________________________

How is low blood pressure/hypotension treated?
(Tick all that apply)

☐ no treatment ☐ diet ☐ exercise ☐ medication (please specify) ☐ natural/herbal therapy (please specify) ☐ weight management ☐ supplements (please specify) ☐ other (please specify)
☐ Don't know

please specify the medication

____

please specify the natural/herbal therapy

____

please specify the supplements

____

please specify the treatment

____

How is hardening of the arteries/atherosclerosis/arteriosclerosis treated?
(Tick all that apply)

☐ no treatment ☐ diet ☐ exercise ☐ medication (please specify) ☐ natural/herbal therapy (please specify) ☐ weight management ☐ supplements (please specify) ☐ other (please specify)
☐ Don't know

please specify the medication

____

please specify the natural/herbal therapy

____

please specify the supplements

____

please specify the treatment

____

How is fluid problems/fluid retention/oedema treated?
(Tick all that apply)

☐ no treatment ☐ diet ☐ exercise ☐ medication (please specify) ☐ natural/herbal therapy (please specify) ☐ weight management ☐ supplements (please specify) ☐ other (please specify)
☐ Don't know

please specify the medication

____

please specify the natural/herbal therapy

____

please specify the supplements

____

please specify the treatment

____

How is high cholesterol treated?
(Tick all that apply)

☐ no treatment ☐ diet ☐ exercise ☐ medication (please specify) ☐ natural/herbal therapy (please specify) ☐ weight management ☐ supplements (please specify) ☐ other (please specify)
☐ Don't know

please specify the medication ______________________

please specify the natural/herbal therapy ______________________

please specify the supplements ______________________

please specify the treatment ______________________

How is rapid or irregular heartbeats/tachycardia/palpitations treated?
(Tick all that apply)

☐ no treatment ☐ diet ☐ exercise ☐ medication (please specify) ☐ natural/herbal therapy (please specify) ☐ weight management ☐ supplements (please specify) ☐ other (please specify)
☐ Don't know

please specify the medication ______________________

please specify the natural/herbal therapy ______________________

please specify the supplements ______________________

please specify the treatment ______________________

How is heart murmur/heart valve disorder treated/
(Tick all that apply)

☐ no treatment ☐ diet ☐ exercise ☐ medication (please specify) ☐ natural/herbal therapy (please specify) ☐ weight management ☐ supplements (please specify) ☐ other (please specify)
☐ Don't know

please specify the medication ______________________

please specify the natural/herbal therapy ______________________

please specify the supplements ______________________

please specify the treatment ______________________

How is haemorrohoids treataed?
(Tick all that apply)

☐ no treatment ☐ diet ☐ exercise ☐ medication (please specify) ☐ natural/herbal therapy (please specify) ☐ weight management ☐ supplements (please specify) ☐ other (please specify)
☐ Don't know

please specify the medication

____

please specify the natural/herbal therapy

____

please specify the supplements

____

please specify the treatment

____

How is varicose veins treated?
(Tick all that apply)

☐ no treatment ☐ diet ☐ exercise ☐ medication (please specify) ☐ natural/herbal therapy (please specify) ☐ weight management ☐ supplements (please specify) ☐ other (please specify)
☐ Don't know

please specify the medication

____

please specify the natural/herbal therapy

____

please specify the supplements

____

please specify the treatment

____

How is [heart_others] treated?
(Tick all that apply)

☐ no treatment ☐ diet ☐ exercise ☐ medication (please specify) ☐ natural/herbal therapy (please specify) ☐ weight management ☐ supplements (please specify) ☐ other (please specify)
☐ Don't know

please specify the medication

____

please specify the natural/herbal therapy

____

please specify the supplements

____

please specify the treatment

____

How is [heart_others_2] treated?
(Tick all that apply)

☐ no treatment ☐ diet ☐ exercise ☐ medication (please specify) ☐ natural/herbal therapy (please specify) ☐ weight management ☐ supplements (please specify) ☐ other (please specify) ☐ Don't know

please specify the medication

____

please specify the natural/herbal therapy

____

please specify the supplements

____

please specify the treatment

____

How is [heart_others_3] treated?
(Tick all that apply)

☐ no treatment ☐ diet ☐ exercise ☐ medication (please specify) ☐ natural/herbal therapy (please specify) ☐ weight management ☐ supplements (please specify) ☐ other (please specify) ☐ Don't know

please specify the medication

____

please specify the natural/herbal therapy

____

please specify the supplements

____

please specify the treatment

____

How is this condition treated?
(Tick all that apply)

☐ no treatment ☐ diet ☐ exercise ☐ medication (please specify) ☐ natural/herbal therapy (please specify) ☐ weight management ☐ supplements (please specify) ☐ other (please specify) ☐ Don't know

please specify the medication

____

please specify the natural/herbal therapy

____

please specify the supplements

____

please specify the treatment

____

Have either of your parents, or any of brothers or sisters ever been told by a doctor (GP or specialist) or nurse that they have any heart or circulatory conditions?

◯ Yes
◯ No
◯ Don't know

Including any conditions which can be controlled with medication, have you ever been told by a doctor (GP or specialist) or nurse that you have diabetes or pre-diabetes?

◯ Yes
◯ No
◯ Don't know

What are the names of this/these condition/s?
(Tick any that apply)

☐ Diabetes insipidus
☐ Gestational (pregnancy)
☐ Pre-diabetes (impaired fasting glucose, impaired glucose tolerance)
☐ Type 1 (Insulin Dependent Diabetes Mellitus/Juvenile onset Diabetes /Type A)
☐ Type 2 (Non-insulin Dependent Diabetes Mellitus/Adult onset Diabetes/Type B)
☐ Type unknown
☐ Other (please specify)

please specify the condition

____

Do you have another diabetes condition need to be specified?

◯ Yes ◯ No

please specify the second condition

____

Do you have another diabetes condition need to be specified?

◯ Yes ◯ No

please specify the third condition

____

At what age were you first told you had Type 1 diabetes (Insulin Dependent Diabetes Mellitus/Juvenile onset Diabetes /Type A)?

◯ 0 ◯ 1 ◯ 2 ◯ 3
◯ 4 ◯ 5 ◯ 6 ◯ 7
◯ 8 ◯ 9 ◯ 10 ◯ 11
◯ 12 ◯ 13 ◯ 14 ◯ 15
◯ 16 ◯ 17 ◯ 18 ◯ 19
◯ 20 ◯ 21 ◯ 22 ◯ 23
◯ 24 ◯ 25 ◯ 26 ◯ 27
◯ 28 ◯ 29 ◯ 30 ◯ 31
◯ 32 ◯ 33 ◯ 34 ◯ 35
◯ 36 ◯ 37 ◯ 38 ◯ 39
◯ 40 ◯ 41 ◯ 42 ◯ 43
◯ 44 ◯ 45 ◯ 46 ◯ 47
◯ 48 ◯ 49 ◯ 50 ◯ 51
◯ 52 ◯ 53 ◯ 54 ◯ 55
◯ 56 ◯ 57 ◯ 58 ◯ 59
◯ 60 ◯ 61 ◯ 62 ◯ 63
◯ 64 ◯ 65 ◯ 66 ◯ 67
◯ 68 ◯ 69 ◯ 70 ◯ 71
◯ 72 ◯ 73 ◯ 74 ◯ 75
◯ 76 ◯ 77 ◯ 78 ◯ 79
◯ 80 ◯ 81 ◯ 82 ◯ 83
◯ 84 ◯ 85 ◯ 86 ◯ 87
◯ 88 ◯ 89 ◯ 90 ◯ 91
◯ 92 ◯ 93 ◯ 94 ◯ 95
◯ 96 ◯ 97 ◯ 98 ◯ 99
◯ Don't know

At what age were you first told you had Type 2 diabetes (Non-insulin Dependent Diabetes Mellitus/Adult Onset Diabetes/Type B)?

- ◯ 0
- ◯ 1
- ◯ 2
- ◯ 3
- ◯ 4
- ◯ 5
- ◯ 6
- ◯ 7
- ◯ 8
- ◯ 9
- ◯ 10
- ◯ 11
- ◯ 12
- ◯ 13
- ◯ 14
- ◯ 15
- ◯ 16
- ◯ 17
- ◯ 18
- ◯ 19
- ◯ 20
- ◯ 21
- ◯ 22
- ◯ 23
- ◯ 24
- ◯ 25
- ◯ 26
- ◯ 27
- ◯ 28
- ◯ 29
- ◯ 30
- ◯ 31
- ◯ 32
- ◯ 33
- ◯ 34
- ◯ 35
- ◯ 36
- ◯ 37
- ◯ 38
- ◯ 39
- ◯ 40
- ◯ 41
- ◯ 42
- ◯ 43
- ◯ 44
- ◯ 45
- ◯ 46
- ◯ 47
- ◯ 48
- ◯ 49
- ◯ 50
- ◯ 51
- ◯ 52
- ◯ 53
- ◯ 54
- ◯ 55
- ◯ 56
- ◯ 57
- ◯ 58
- ◯ 59
- ◯ 60
- ◯ 61
- ◯ 62
- ◯ 63
- ◯ 64
- ◯ 65
- ◯ 66
- ◯ 67
- ◯ 68

- ◯ 69
- ◯ 70
- ◯ 71
- ◯ 72
- ◯ 73
- ◯ 74
- ◯ 75
- ◯ 76
- ◯ 77
- ◯ 78
- ◯ 79
- ◯ 80
- ◯ 81
- ◯ 82
- ◯ 83
- ◯ 84
- ◯ 85
- ◯ 86
- ◯ 87
- ◯ 88
- ◯ 89
- ◯ 90
- ◯ 91
- ◯ 92
- ◯ 93
- ◯ 94
- ◯ 95
- ◯ 96
- ◯ 97
- ◯ 98
- ◯ 99
- ◯ Don't know

At what age were you first told you had gestational (pregnancy) diabetes?

◯ 0
◯ 1
◯ 2
◯ 3
◯ 4
◯ 5
◯ 6
◯ 7
◯ 8
◯ 9
◯ 10
◯ 11
◯ 12
◯ 13
◯ 14
◯ 15
◯ 16
◯ 17
◯ 18
◯ 19
◯ 20
◯ 21
◯ 22
◯ 23
◯ 24
◯ 25
◯ 26
◯ 27
◯ 28
◯ 29
◯ 30
◯ 31
◯ 32
◯ 33
◯ 34
◯ 35
◯ 36
◯ 37
◯ 38
◯ 39
◯ 40
◯ 41
◯ 42
◯ 43
◯ 44
◯ 45
◯ 46
◯ 47
◯ 48
◯ 49
◯ 50
◯ 51
◯ 52
◯ 53
◯ 54
◯ 55
◯ 56
◯ 57
◯ 58
◯ 59
◯ 60
◯ 61
◯ 62
◯ 63
◯ 64
◯ 65
◯ 66
◯ 67
◯ 68

◯ 69
◯ 70
◯ 71
◯ 72
◯ 73
◯ 74
◯ 75
◯ 76
◯ 77
◯ 78
◯ 79
◯ 80
◯ 81
◯ 82
◯ 83
◯ 84
◯ 85
◯ 86
◯ 87
◯ 88
◯ 89
◯ 90
◯ 91
◯ 92
◯ 93
◯ 94
◯ 95
◯ 96
◯ 97
◯ 98
◯ 99
◯ Don't know

At what age were you first told you had diabetes insipidus?

- ◯ 0
- ◯ 1
- ◯ 2
- ◯ 3
- ◯ 4
- ◯ 5
- ◯ 6
- ◯ 7
- ◯ 8
- ◯ 9
- ◯ 10
- ◯ 11
- ◯ 12
- ◯ 13
- ◯ 14
- ◯ 15
- ◯ 16
- ◯ 17
- ◯ 18
- ◯ 19
- ◯ 20
- ◯ 21
- ◯ 22
- ◯ 23
- ◯ 24
- ◯ 25
- ◯ 26
- ◯ 27
- ◯ 28
- ◯ 29
- ◯ 30
- ◯ 31
- ◯ 32
- ◯ 33
- ◯ 34
- ◯ 35
- ◯ 36
- ◯ 37
- ◯ 38
- ◯ 39
- ◯ 40
- ◯ 41
- ◯ 42
- ◯ 43
- ◯ 44
- ◯ 45
- ◯ 46
- ◯ 47
- ◯ 48
- ◯ 49
- ◯ 50
- ◯ 51
- ◯ 52
- ◯ 53
- ◯ 54
- ◯ 55
- ◯ 56
- ◯ 57
- ◯ 58
- ◯ 59
- ◯ 60
- ◯ 61
- ◯ 62
- ◯ 63
- ◯ 64
- ◯ 65
- ◯ 66
- ◯ 67
- ◯ 68

- ◯ 69
- ◯ 70
- ◯ 71
- ◯ 72
- ◯ 73
- ◯ 74
- ◯ 75
- ◯ 76
- ◯ 77
- ◯ 78
- ◯ 79
- ◯ 80
- ◯ 81
- ◯ 82
- ◯ 83
- ◯ 84
- ◯ 85
- ◯ 86
- ◯ 87
- ◯ 88
- ◯ 89
- ◯ 90
- ◯ 91
- ◯ 92
- ◯ 93
- ◯ 94
- ◯ 95
- ◯ 96
- ◯ 97
- ◯ 98
- ◯ 99
- ◯ Don't know

At what age were you first told you had pre-diabetes (impaired fasting glucose, impaired glucose intolerance)?

- 0
- 1
- 2
- 3
- 4
- 5
- 6
- 7
- 8
- 9
- 10
- 11
- 12
- 13
- 14
- 15
- 16
- 17
- 18
- 19
- 20
- 21
- 22
- 23
- 24
- 25
- 26
- 27
- 28
- 29
- 30
- 31
- 32
- 33
- 34
- 35
- 36
- 37
- 38
- 39
- 40
- 41
- 42
- 43
- 44
- 45
- 46
- 47
- 48
- 49
- 50
- 51
- 52
- 53
- 54
- 55
- 56
- 57
- 58
- 59
- 60
- 61
- 62
- 63
- 64
- 65
- 66
- 67
- 68

◯ 69
◯ 70
◯ 71
◯ 72
◯ 73
◯ 74
◯ 75
◯ 76
◯ 77
◯ 78
◯ 79
◯ 80
◯ 81
◯ 82
◯ 83
◯ 84
◯ 85
◯ 86
◯ 87
◯ 88
◯ 89
◯ 90
◯ 91
◯ 92
◯ 93
◯ 94
◯ 95
◯ 96
◯ 97
◯ 98
◯ 99
◯ Don't know

At what age were you first told you had this condition?

○ 0
○ 1
○ 2
○ 3
○ 4
○ 5
○ 6
○ 7
○ 8
○ 9
○ 10
○ 11
○ 12
○ 13
○ 14
○ 15
○ 16
○ 17
○ 18
○ 19
○ 20
○ 21
○ 22
○ 23
○ 24
○ 25
○ 26
○ 27
○ 28
○ 29
○ 30
○ 31
○ 32
○ 33
○ 34
○ 35
○ 36
○ 37
○ 38
○ 39
○ 40
○ 41
○ 42
○ 43
○ 44
○ 45
○ 46
○ 47
○ 48
○ 49
○ 50
○ 51
○ 52
○ 53
○ 54
○ 55
○ 56
○ 57
○ 58
○ 59
○ 60
○ 61
○ 62
○ 63
○ 64
○ 65
○ 66
○ 67
○ 68

- ◯ 69
- ◯ 70
- ◯ 71
- ◯ 72
- ◯ 73
- ◯ 74
- ◯ 75
- ◯ 76
- ◯ 77
- ◯ 78
- ◯ 79
- ◯ 80
- ◯ 81
- ◯ 82
- ◯ 83
- ◯ 84
- ◯ 85
- ◯ 86
- ◯ 87
- ◯ 88
- ◯ 89
- ◯ 90
- ◯ 91
- ◯ 92
- ◯ 93
- ◯ 94
- ◯ 95
- ◯ 96
- ◯ 97
- ◯ 98
- ◯ 99
- ◯ Don't know

At what age were you first told you had [diabetes_others]?

◯ 0
◯ 1
◯ 2
◯ 3
◯ 4
◯ 5
◯ 6
◯ 7
◯ 8
◯ 9
◯ 10
◯ 11
◯ 12
◯ 13
◯ 14
◯ 15
◯ 16
◯ 17
◯ 18
◯ 19
◯ 20
◯ 21
◯ 22
◯ 23
◯ 24
◯ 25
◯ 26
◯ 27
◯ 28
◯ 29
◯ 30
◯ 31
◯ 32
◯ 33
◯ 34
◯ 35
◯ 36
◯ 37
◯ 38
◯ 39
◯ 40
◯ 41
◯ 42
◯ 43
◯ 44
◯ 45
◯ 46
◯ 47
◯ 48
◯ 49
◯ 50
◯ 51
◯ 52
◯ 53
◯ 54
◯ 55
◯ 56
◯ 57
◯ 58
◯ 59
◯ 60
◯ 61
◯ 62
◯ 63
◯ 64
◯ 65
◯ 66
◯ 67
◯ 68

◯ 69
◯ 70
◯ 71
◯ 72
◯ 73
◯ 74
◯ 75
◯ 76
◯ 77
◯ 78
◯ 79
◯ 80
◯ 81
◯ 82
◯ 83
◯ 84
◯ 85
◯ 86
◯ 87
◯ 88
◯ 89
◯ 90
◯ 91
◯ 92
◯ 93
◯ 94
◯ 95
◯ 96
◯ 97
◯ 98
◯ 99
◯ Don't know

At what age were you first told you had [diabetes_others_2]?

- ◯ 0
- ◯ 1
- ◯ 2
- ◯ 3
- ◯ 4
- ◯ 5
- ◯ 6
- ◯ 7
- ◯ 8
- ◯ 9
- ◯ 10
- ◯ 11
- ◯ 12
- ◯ 13
- ◯ 14
- ◯ 15
- ◯ 16
- ◯ 17
- ◯ 18
- ◯ 19
- ◯ 20
- ◯ 21
- ◯ 22
- ◯ 23
- ◯ 24
- ◯ 25
- ◯ 26
- ◯ 27
- ◯ 28
- ◯ 29
- ◯ 30
- ◯ 31
- ◯ 32
- ◯ 33
- ◯ 34
- ◯ 35
- ◯ 36
- ◯ 37
- ◯ 38
- ◯ 39
- ◯ 40
- ◯ 41
- ◯ 42
- ◯ 43
- ◯ 44
- ◯ 45
- ◯ 46
- ◯ 47
- ◯ 48
- ◯ 49
- ◯ 50
- ◯ 51
- ◯ 52
- ◯ 53
- ◯ 54
- ◯ 55
- ◯ 56
- ◯ 57
- ◯ 58
- ◯ 59
- ◯ 60
- ◯ 61
- ◯ 62
- ◯ 63
- ◯ 64
- ◯ 65
- ◯ 66
- ◯ 67
- ◯ 68

- ◯ 69
- ◯ 70
- ◯ 71
- ◯ 72
- ◯ 73
- ◯ 74
- ◯ 75
- ◯ 76
- ◯ 77
- ◯ 78
- ◯ 79
- ◯ 80
- ◯ 81
- ◯ 82
- ◯ 83
- ◯ 84
- ◯ 85
- ◯ 86
- ◯ 87
- ◯ 88
- ◯ 89
- ◯ 90
- ◯ 91
- ◯ 92
- ◯ 93
- ◯ 94
- ◯ 95
- ◯ 96
- ◯ 97
- ◯ 98
- ◯ 99
- ◯ Don't know

At what age were you first told you had [diabetes_others_3]?

- 0
- 1
- 2
- 3
- 4
- 5
- 6
- 7
- 8
- 9
- 10
- 11
- 12
- 13
- 14
- 15
- 16
- 17
- 18
- 19
- 20
- 21
- 22
- 23
- 24
- 25
- 26
- 27
- 28
- 29
- 30
- 31
- 32
- 33
- 34
- 35
- 36
- 37
- 38
- 39
- 40
- 41
- 42
- 43
- 44
- 45
- 46
- 47
- 48
- 49
- 50
- 51
- 52
- 53
- 54
- 55
- 56
- 57
- 58
- 59
- 60
- 61
- 62
- 63
- 64
- 65
- 66
- 67
- 68

◯ 69
◯ 70
◯ 71
◯ 72
◯ 73
◯ 74
◯ 75
◯ 76
◯ 77
◯ 78
◯ 79
◯ 80
◯ 81
◯ 82
◯ 83
◯ 84
◯ 85
◯ 86
◯ 87
◯ 88
◯ 89
◯ 90
◯ 91
◯ 92
◯ 93
◯ 94
◯ 95
◯ 96
◯ 97
◯ 98
◯ 99
◯ Don't know

Do you still have Type 1/Type A diabetes?	◯ Yes<br>◯ No<br>◯ Don't know
Do you still have Type 2/Type B diabetes?	◯ Yes<br>◯ No<br>◯ Don't know
Do you still have gestational diabetes?	◯ Yes<br>◯ No<br>◯ Don't know
Do you still have diabetes insipidus?	◯ Yes<br>◯ No<br>◯ Don't know
Do you still have pre-diabetes?	◯ Yes<br>◯ No<br>◯ Don't know
Do you still have this condition?	◯ Yes<br>◯ No<br>◯ Don't know
Do you still have [diabetes_others]?	◯ Yes<br>◯ No<br>◯ Don't know

Do you still have [diabetes_others_2]?

◯ Yes
◯ No
◯ Don't know

Do you still have [diabetes_others_3]?

◯ Yes
◯ No
◯ Don't know

How is Type 1 diabetes (Insulin Dependent Diabetes Mellitus/Juvenile Onset Diabetes /Type A) treated? (Tick all that apply)

☐ no treatment ☐ diet ☐ exercise ☐ medication (please specify) ☐ natural/herbal therapy (please specify) ☐ weight management ☐ supplements (please specify) ☐ other (please specify) ☐ Don't know

please specify the medication ______________________

please specify the natural/herbal therapy ______________________

please specify the supplements ______________________

please specify the treatment ______________________

How is Type 2 diabetes (Non-insulin Dependent Diabetes Mellitus/Adult Onset Diabetes/Type B treated? (Tick all that apply)

☐ no treatment ☐ diet ☐ exercise ☐ medication (please specify) ☐ natural/herbal therapy (please specify) ☐ weight management ☐ supplements (please specify) ☐ other (please specify) ☐ Don't know

please specify the medication ______________________

please specify the natural/herbal therapy ______________________

please specify the supplements ______________________

please specify the treatment ______________________

How is gestational (pregnancy) diabetes treated?
(Tick all that apply)

☐ no treatment ☐ diet ☐ exercise ☐ medication (please specify) ☐ natural/herbal therapy (please specify) ☐ weight management ☐ supplements (please specify) ☐ other (please specify)
☐ Don't know

please specify the medication ________________________

please specify the natural/herbal therapy ________________________

please specify the supplements ________________________

please specify the treatment ________________________

How is diabetes insipidus treated?
(Tick all that apply)

☐ no treatment ☐ diet ☐ exercise ☐ medication (please specify) ☐ natural/herbal therapy (please specify) ☐ weight management ☐ supplements (please specify) ☐ other (please specify)
☐ Don't know

please specify the medication ________________________

please specify the natural/herbal therapy ________________________

please specify the supplements ________________________

please specify the treatment ________________________

How is pre-diabetes (impaired fasting glucose, impaired glucose tolerance) treated?
(Tick all that apply)

☐ no treatment ☐ diet ☐ exercise ☐ medication (please specify) ☐ natural/herbal therapy (please specify) ☐ weight management ☐ supplements (please specify) ☐ other (please specify)
☐ Don't know

please specify the medication ________________________

please specify the natural/herbal therapy ________________________

please specify the supplements ________________________

please specify the treatment ________________________

How is this condition treated?
(Tick all that apply)

☐ no treatment ☐ diet ☐ exercise ☐ medication (please specify) ☐ natural/herbal therapy (please specify) ☐ weight management ☐ supplements (please specify) ☐ other (please specify)
☐ Don't know

please specify the medication
____

please specify the natural/herbal therapy
____

please specify the supplements
____

please specify the treatment
____

How is [diabetes_others] treated?
(Tick all that apply)

☐ no treatment ☐ diet ☐ exercise ☐ medication (please specify) ☐ natural/herbal therapy (please specify) ☐ weight management ☐ supplements (please specify) ☐ other (please specify)
☐ Don't know

please specify the medication
____

please specify the natural/herbal therapy
____

please specify the supplements
____

please specify the treatment
____

How is [diabetes_others_2] treated?
(Tick all that apply)

☐ no treatment ☐ diet ☐ exercise ☐ medication (please specify) ☐ natural/herbal therapy (please specify) ☐ weight management ☐ supplements (please specify) ☐ other (please specify)
☐ Don't know

please specify the medication
____

please specify the natural/herbal therapy
____

please specify the supplements
____

please specify the treatment
____

How is [diabetes_others_3] treated?
(Tick all that apply)

☐ no treatment ☐ diet ☐ exercise ☐ medication (please specify) ☐ natural/herbal therapy (please specify) ☐ weight management ☐ supplements (please specify) ☐ other (please specify)
☐ Don't know

please specify the medication ____________________

please specify the natural/herbal therapy ____________________

please specify the supplements ____________________

please specify the treatment ____________________

Have either of your parents, or any of brothers or sisters ever been told by a doctor (GP or specialist) or nurse that they have Diabetes?
○ Yes
○ No
○ Don't know

Including any conditions which can be controlled with medication, have you ever been told by a doctor (GP or specialist) or nurse that you have osteoporosis or osteopenia?
(Osteopenia is a mild loss of bone density that may progress to osteoporosis.)
○ Yes
○ No
○ Don't know

Which one were you told you have?
☐ Osteoporosis
☐ Osteopenia
☐ Don't Know
(Tick all that apply)

At what age were you first told you had osteoporosis?

- ◯ 0
- ◯ 1
- ◯ 2
- ◯ 3
- ◯ 4
- ◯ 5
- ◯ 6
- ◯ 7
- ◯ 8
- ◯ 9
- ◯ 10
- ◯ 11
- ◯ 12
- ◯ 13
- ◯ 14
- ◯ 15
- ◯ 16
- ◯ 17
- ◯ 18
- ◯ 19
- ◯ 20
- ◯ 21
- ◯ 22
- ◯ 23
- ◯ 24
- ◯ 25
- ◯ 26
- ◯ 27
- ◯ 28
- ◯ 29
- ◯ 30
- ◯ 31
- ◯ 32
- ◯ 33
- ◯ 34
- ◯ 35
- ◯ 36
- ◯ 37
- ◯ 38
- ◯ 39
- ◯ 40
- ◯ 41
- ◯ 42
- ◯ 43
- ◯ 44
- ◯ 45
- ◯ 46
- ◯ 47
- ◯ 48
- ◯ 49
- ◯ 50
- ◯ 51
- ◯ 52
- ◯ 53
- ◯ 54
- ◯ 55
- ◯ 56
- ◯ 57
- ◯ 58
- ◯ 59
- ◯ 60
- ◯ 61
- ◯ 62
- ◯ 63
- ◯ 64
- ◯ 65
- ◯ 66
- ◯ 67
- ◯ 68

- ◯ 69
- ◯ 70
- ◯ 71
- ◯ 72
- ◯ 73
- ◯ 74
- ◯ 75
- ◯ 76
- ◯ 77
- ◯ 78
- ◯ 79
- ◯ 80
- ◯ 81
- ◯ 82
- ◯ 83
- ◯ 84
- ◯ 85
- ◯ 86
- ◯ 87
- ◯ 88
- ◯ 89
- ◯ 90
- ◯ 91
- ◯ 92
- ◯ 93
- ◯ 94
- ◯ 95
- ◯ 96
- ◯ 97
- ◯ 98
- ◯ 99
- ◯ Don't know

At what age were you first told you had osteopenia?

- ◯ 0
- ◯ 1
- ◯ 2
- ◯ 3
- ◯ 4
- ◯ 5
- ◯ 6
- ◯ 7
- ◯ 8
- ◯ 9
- ◯ 10
- ◯ 11
- ◯ 12
- ◯ 13
- ◯ 14
- ◯ 15
- ◯ 16
- ◯ 17
- ◯ 18
- ◯ 19
- ◯ 20
- ◯ 21
- ◯ 22
- ◯ 23
- ◯ 24
- ◯ 25
- ◯ 26
- ◯ 27
- ◯ 28
- ◯ 29
- ◯ 30
- ◯ 31
- ◯ 32
- ◯ 33
- ◯ 34
- ◯ 35
- ◯ 36
- ◯ 37
- ◯ 38
- ◯ 39
- ◯ 40
- ◯ 41
- ◯ 42
- ◯ 43
- ◯ 44
- ◯ 45
- ◯ 46
- ◯ 47
- ◯ 48
- ◯ 49
- ◯ 50
- ◯ 51
- ◯ 52
- ◯ 53
- ◯ 54
- ◯ 55
- ◯ 56
- ◯ 57
- ◯ 58
- ◯ 59
- ◯ 60
- ◯ 61
- ◯ 62
- ◯ 63
- ◯ 64
- ◯ 65
- ◯ 66
- ◯ 67
- ◯ 68

- ◯ 69
- ◯ 70
- ◯ 71
- ◯ 72
- ◯ 73
- ◯ 74
- ◯ 75
- ◯ 76
- ◯ 77
- ◯ 78
- ◯ 79
- ◯ 80
- ◯ 81
- ◯ 82
- ◯ 83
- ◯ 84
- ◯ 85
- ◯ 86
- ◯ 87
- ◯ 88
- ◯ 89
- ◯ 90
- ◯ 91
- ◯ 92
- ◯ 93
- ◯ 94
- ◯ 95
- ◯ 96
- ◯ 97
- ◯ 98
- ◯ 99
- ◯ Don't know

At what age were you first told you had this condition?

◯ 0
◯ 1
◯ 2
◯ 3
◯ 4
◯ 5
◯ 6
◯ 7
◯ 8
◯ 9
◯ 10
◯ 11
◯ 12
◯ 13
◯ 14
◯ 15
◯ 16
◯ 17
◯ 18
◯ 19
◯ 20
◯ 21
◯ 22
◯ 23
◯ 24
◯ 25
◯ 26
◯ 27
◯ 28
◯ 29
◯ 30
◯ 31
◯ 32
◯ 33
◯ 34
◯ 35
◯ 36
◯ 37
◯ 38
◯ 39
◯ 40
◯ 41
◯ 42
◯ 43
◯ 44
◯ 45
◯ 46
◯ 47
◯ 48
◯ 49
◯ 50
◯ 51
◯ 52
◯ 53
◯ 54
◯ 55
◯ 56
◯ 57
◯ 58
◯ 59
◯ 60
◯ 61
◯ 62
◯ 63
◯ 64
◯ 65
◯ 66
◯ 67
◯ 68

	◯ 69<br>◯ 70<br>◯ 71<br>◯ 72<br>◯ 73<br>◯ 74<br>◯ 75<br>◯ 76<br>◯ 77<br>◯ 78<br>◯ 79<br>◯ 80<br>◯ 81<br>◯ 82<br>◯ 83<br>◯ 84<br>◯ 85<br>◯ 86<br>◯ 87<br>◯ 88<br>◯ 89<br>◯ 90<br>◯ 91<br>◯ 92<br>◯ 93<br>◯ 94<br>◯ 95<br>◯ 96<br>◯ 97<br>◯ 98<br>◯ 99<br>◯ Don't know
Do you still have osteoporosis?	◯ Yes<br>◯ No<br>◯ Don't know
Do you still have osteopenia?	◯ Yes<br>◯ No<br>◯ Don't know
Do you still have this condition?	◯ Yes<br>◯ No<br>◯ Don't know

How is osteoporosis treated?
(Tick all that apply)

☐ no treatment ☐ diet ☐ exercise ☐ medication (please specify) ☐ natural/herbal therapy (please specify) ☐ weight management ☐ supplements (please specify) ☐ other (please specify) ☐ Don't know

please specify the medication	________________________________
please specify the natural/herbal therapy	________________________________
please specify the supplements	________________________________

please specify the treatment ______________________

How is osteopenia treated?
(Tick all that apply)

☐ no treatment ☐ diet ☐ exercise ☐ medication (please specify) ☐ natural/herbal therapy (please specify) ☐ weight management ☐ supplements (please specify) ☐ other (please specify)
☐ Don't know

please specify the medication ______________________

please specify the natural/herbal therapy ______________________

please specify the supplements ______________________

please specify the treatment ______________________

How is this condition treated?
(Tick all that apply)

☐ no treatment ☐ diet ☐ exercise ☐ medication (please specify) ☐ natural/herbal therapy (please specify) ☐ weight management ☐ supplements (please specify) ☐ other (please specify)
☐ Don't know

please specify the medication ______________________

please specify the natural/herbal therapy ______________________

please specify the supplements ______________________

please specify the treatment ______________________

Have either of your parents, or any of brothers or sisters ever been told by a doctor (GP or specialist) that they have osteoporosis or osteopaenia?

◯ Yes
◯ No
◯ Don't know

Which best describes the *regularity of your usual menstrual periods between ages 20 and 35 when you were neither pregnant nor using oral contraceptives?

◯ Very regular
◯ Usually regular
◯ Usually irregular
◯ Very irregular

*Regular periods are between 26 and 34 days. Irregular periods are 35 days or more between periods

Between the ages of 20 and 35, did you ever experience amenorrhoea (no periods for more than 3 months) when you were neither pregnant nor using oral contraceptives?

◯ Yes
◯ No
◯ Don't know

Have you ever been told by a doctor (GP or specialist) or nurse that you have gout, osteoarthritis or rheumatoid arthritis?	◯ Yes<br>◯ No<br>◯ Don't know

Which one were you told you have?
(Tick any that apply)

☐ Gout ☐ Osteoarthritis ☐ Rheumatoid arthritis ☐ Don't know

At what age were you first told you had gout?

- ◯ 0
- ◯ 1
- ◯ 2
- ◯ 3
- ◯ 4
- ◯ 5
- ◯ 6
- ◯ 7
- ◯ 8
- ◯ 9
- ◯ 10
- ◯ 11
- ◯ 12
- ◯ 13
- ◯ 14
- ◯ 15
- ◯ 16
- ◯ 17
- ◯ 18
- ◯ 19
- ◯ 20
- ◯ 21
- ◯ 22
- ◯ 23
- ◯ 24
- ◯ 25
- ◯ 26
- ◯ 27
- ◯ 28
- ◯ 29
- ◯ 30
- ◯ 31
- ◯ 32
- ◯ 33
- ◯ 34
- ◯ 35
- ◯ 36
- ◯ 37
- ◯ 38
- ◯ 39
- ◯ 40
- ◯ 41
- ◯ 42
- ◯ 43
- ◯ 44
- ◯ 45
- ◯ 46
- ◯ 47
- ◯ 48
- ◯ 49
- ◯ 50
- ◯ 51
- ◯ 52
- ◯ 53
- ◯ 54
- ◯ 55
- ◯ 56
- ◯ 57
- ◯ 58
- ◯ 59
- ◯ 60
- ◯ 61
- ◯ 62
- ◯ 63
- ◯ 64
- ◯ 65
- ◯ 66
- ◯ 67
- ◯ 68

◯ 69
◯ 70
◯ 71
◯ 72
◯ 73
◯ 74
◯ 75
◯ 76
◯ 77
◯ 78
◯ 79
◯ 80
◯ 81
◯ 82
◯ 83
◯ 84
◯ 85
◯ 86
◯ 87
◯ 88
◯ 89
◯ 90
◯ 91
◯ 92
◯ 93
◯ 94
◯ 95
◯ 96
◯ 97
◯ 98
◯ 99
◯ Don't know

At what age were you first told you had osteoarthritis?

- ◯ 0
- ◯ 1
- ◯ 2
- ◯ 3
- ◯ 4
- ◯ 5
- ◯ 6
- ◯ 7
- ◯ 8
- ◯ 9
- ◯ 10
- ◯ 11
- ◯ 12
- ◯ 13
- ◯ 14
- ◯ 15
- ◯ 16
- ◯ 17
- ◯ 18
- ◯ 19
- ◯ 20
- ◯ 21
- ◯ 22
- ◯ 23
- ◯ 24
- ◯ 25
- ◯ 26
- ◯ 27
- ◯ 28
- ◯ 29
- ◯ 30
- ◯ 31
- ◯ 32
- ◯ 33
- ◯ 34
- ◯ 35
- ◯ 36
- ◯ 37
- ◯ 38
- ◯ 39
- ◯ 40
- ◯ 41
- ◯ 42
- ◯ 43
- ◯ 44
- ◯ 45
- ◯ 46
- ◯ 47
- ◯ 48
- ◯ 49
- ◯ 50
- ◯ 51
- ◯ 52
- ◯ 53
- ◯ 54
- ◯ 55
- ◯ 56
- ◯ 57
- ◯ 58
- ◯ 59
- ◯ 60
- ◯ 61
- ◯ 62
- ◯ 63
- ◯ 64
- ◯ 65
- ◯ 66
- ◯ 67
- ◯ 68

- ◯ 69
- ◯ 70
- ◯ 71
- ◯ 72
- ◯ 73
- ◯ 74
- ◯ 75
- ◯ 76
- ◯ 77
- ◯ 78
- ◯ 79
- ◯ 80
- ◯ 81
- ◯ 82
- ◯ 83
- ◯ 84
- ◯ 85
- ◯ 86
- ◯ 87
- ◯ 88
- ◯ 89
- ◯ 90
- ◯ 91
- ◯ 92
- ◯ 93
- ◯ 94
- ◯ 95
- ◯ 96
- ◯ 97
- ◯ 98
- ◯ 99
- ◯ Don't know

At what age were you first told you had rheumatoid arthritis?

◯ 0
◯ 1
◯ 2
◯ 3
◯ 4
◯ 5
◯ 6
◯ 7
◯ 8
◯ 9
◯ 10
◯ 11
◯ 12
◯ 13
◯ 14
◯ 15
◯ 16
◯ 17
◯ 18
◯ 19
◯ 20
◯ 21
◯ 22
◯ 23
◯ 24
◯ 25
◯ 26
◯ 27
◯ 28
◯ 29
◯ 30
◯ 31
◯ 32
◯ 33
◯ 34
◯ 35
◯ 36
◯ 37
◯ 38
◯ 39
◯ 40
◯ 41
◯ 42
◯ 43
◯ 44
◯ 45
◯ 46
◯ 47
◯ 48
◯ 49
◯ 50
◯ 51
◯ 52
◯ 53
◯ 54
◯ 55
◯ 56
◯ 57
◯ 58
◯ 59
◯ 60
◯ 61
◯ 62
◯ 63
◯ 64
◯ 65
◯ 66
◯ 67
◯ 68

- ◯ 69
- ◯ 70
- ◯ 71
- ◯ 72
- ◯ 73
- ◯ 74
- ◯ 75
- ◯ 76
- ◯ 77
- ◯ 78
- ◯ 79
- ◯ 80
- ◯ 81
- ◯ 82
- ◯ 83
- ◯ 84
- ◯ 85
- ◯ 86
- ◯ 87
- ◯ 88
- ◯ 89
- ◯ 90
- ◯ 91
- ◯ 92
- ◯ 93
- ◯ 94
- ◯ 95
- ◯ 96
- ◯ 97
- ◯ 98
- ◯ 99
- ◯ Don't know

At what age were you first told you had this condition?

◯ 0
◯ 1
◯ 2
◯ 3
◯ 4
◯ 5
◯ 6
◯ 7
◯ 8
◯ 9
◯ 10
◯ 11
◯ 12
◯ 13
◯ 14
◯ 15
◯ 16
◯ 17
◯ 18
◯ 19
◯ 20
◯ 21
◯ 22
◯ 23
◯ 24
◯ 25
◯ 26
◯ 27
◯ 28
◯ 29
◯ 30
◯ 31
◯ 32
◯ 33
◯ 34
◯ 35
◯ 36
◯ 37
◯ 38
◯ 39
◯ 40
◯ 41
◯ 42
◯ 43
◯ 44
◯ 45
◯ 46
◯ 47
◯ 48
◯ 49
◯ 50
◯ 51
◯ 52
◯ 53
◯ 54
◯ 55
◯ 56
◯ 57
◯ 58
◯ 59
◯ 60
◯ 61
◯ 62
◯ 63
◯ 64
◯ 65
◯ 66
◯ 67
◯ 68

◯ 69
◯ 70
◯ 71
◯ 72
◯ 73
◯ 74
◯ 75
◯ 76
◯ 77
◯ 78
◯ 79
◯ 80
◯ 81
◯ 82
◯ 83
◯ 84
◯ 85
◯ 86
◯ 87
◯ 88
◯ 89
◯ 90
◯ 91
◯ 92
◯ 93
◯ 94
◯ 95
◯ 96
◯ 97
◯ 98
◯ 99
◯ Don't know

Do you still have gout?

◯ Yes
◯ No
◯ Don't know

Do you still have osteoarthritis?

◯ Yes
◯ No
◯ Don't know

Do you still have rheumatoid arthritis?

◯ Yes
◯ No
◯ Don't know

Do you still have this condition?

◯ Yes
◯ No
◯ Don't know

How is gout treated?
(Tick all that apply)

☐ no treatment ☐ diet ☐ exercise ☐ medication (please specify) ☐ natural/herbal therapy (please specify) ☐ weight management ☐ supplements (please specify) ☐ other (please specify)
☐ Don't know

---

please specify the medication

____

---

please specify the natural/herbal therapy

____

please specify the supplements

____

please specify the treatment

____

How is osteoarthritis treated?
(Tick all that apply)

☐ no treatment ☐ diet ☐ exercise ☐ medication (please specify) ☐ natural/herbal therapy (please specify) ☐ weight management ☐ supplements (please specify) ☐ other (please specify)
☐ Don't know

please specify the medication

____

please specify the natural/herbal therapy

____

please specify the supplements

____

please specify the treatment

____

How is rheumatoid arthritis treated?
(Tick all that apply)

☐ no treatment ☐ diet ☐ exercise ☐ medication (please specify) ☐ natural/herbal therapy (please specify) ☐ weight management ☐ supplements (please specify) ☐ other (please specify)
☐ Don't know

please specify the medication

____

please specify the natural/herbal therapy

____

please specify the supplements

____

please specify the treatment

____

How is this condition treated?
(Tick all that apply)

☐ no treatment ☐ diet ☐ exercise ☐ medication (please specify) ☐ natural/herbal therapy (please specify) ☐ weight management ☐ supplements (please specify) ☐ other (please specify)
☐ Don't know

please specify the medication

____

please specify the natural/herbal therapy

____

please specify the supplements

____

please specify the treatment

____

Have either of your parents, or any of brothers or sisters ever been told by a doctor (GP or specialist) or nurse that they have gout, osteoarthritis or rheumatoid arthritis?

◯ Yes
◯ No
◯ Don't know

Have you ever been told by a doctor (GP or specialist) or nurse that you have any type of cancer?

◯ Yes
◯ No
◯ Don't know

What type/s of cancer were you told you had?
(Tick all that apply)

☐ Bladder/kidney cancer
☐ Breast cancer
☐ Cancer of unknown primary site
☐ Cervical cancer
☐ Cancer of other female reproductive organs (include uterus, ovary)
☐ Colon/rectum/bowel cancer (colorectal)
☐ Leukaemia
☐ Lung cancer (include trachea, pleura and bronchus)
☐ Non-Hodgkin lymphoma
☐ Other type of lymphoma
☐ Prostate cancer
☐ Skin cancer (include melanoma, basal cell carcinoma, squamous cell carcinoma)
☐ Stomach cancer
☐ Other cancer (please specify)
☐ Don't Know

please specify the type of cancer

____

Do you have another type of cancer need to be specified?

◯ Yes ◯ No

please specify the second type of cancer

____

Do you have another type of cancer need to be specified?

◯ Yes ◯ No

please specify the type of cancer

____

At what age were you first told you had skin cancer (include melanoma, basal cell carcinoma, squamous cell carcinoma)?

- ◯ 0
- ◯ 1
- ◯ 2
- ◯ 3
- ◯ 4
- ◯ 5
- ◯ 6
- ◯ 7
- ◯ 8
- ◯ 9
- ◯ 10
- ◯ 11
- ◯ 12
- ◯ 13
- ◯ 14
- ◯ 15
- ◯ 16
- ◯ 17
- ◯ 18
- ◯ 19
- ◯ 20
- ◯ 21
- ◯ 22
- ◯ 23
- ◯ 24
- ◯ 25
- ◯ 26
- ◯ 27
- ◯ 28
- ◯ 29
- ◯ 30
- ◯ 31
- ◯ 32
- ◯ 33
- ◯ 34
- ◯ 35
- ◯ 36
- ◯ 37
- ◯ 38
- ◯ 39
- ◯ 40
- ◯ 41
- ◯ 42
- ◯ 43
- ◯ 44
- ◯ 45
- ◯ 46
- ◯ 47
- ◯ 48
- ◯ 49
- ◯ 50
- ◯ 51
- ◯ 52
- ◯ 53
- ◯ 54
- ◯ 55
- ◯ 56
- ◯ 57
- ◯ 58
- ◯ 59
- ◯ 60
- ◯ 61
- ◯ 62
- ◯ 63
- ◯ 64
- ◯ 65
- ◯ 66
- ◯ 67
- ◯ 68

- ◯ 69
- ◯ 70
- ◯ 71
- ◯ 72
- ◯ 73
- ◯ 74
- ◯ 75
- ◯ 76
- ◯ 77
- ◯ 78
- ◯ 79
- ◯ 80
- ◯ 81
- ◯ 82
- ◯ 83
- ◯ 84
- ◯ 85
- ◯ 86
- ◯ 87
- ◯ 88
- ◯ 89
- ◯ 90
- ◯ 91
- ◯ 92
- ◯ 93
- ◯ 94
- ◯ 95
- ◯ 96
- ◯ 97
- ◯ 98
- ◯ 99
- ◯ Don't know

At what age were you first told you had colon/rectum/bowel cancer (colorectal)?

◯ 0
◯ 1
◯ 2
◯ 3
◯ 4
◯ 5
◯ 6
◯ 7
◯ 8
◯ 9
◯ 10
◯ 11
◯ 12
◯ 13
◯ 14
◯ 15
◯ 16
◯ 17
◯ 18
◯ 19
◯ 20
◯ 21
◯ 22
◯ 23
◯ 24
◯ 25
◯ 26
◯ 27
◯ 28
◯ 29
◯ 30
◯ 31
◯ 32
◯ 33
◯ 34
◯ 35
◯ 36
◯ 37
◯ 38
◯ 39
◯ 40
◯ 41
◯ 42
◯ 43
◯ 44
◯ 45
◯ 46
◯ 47
◯ 48
◯ 49
◯ 50
◯ 51
◯ 52
◯ 53
◯ 54
◯ 55
◯ 56
◯ 57
◯ 58
◯ 59
◯ 60
◯ 61
◯ 62
◯ 63
◯ 64
◯ 65
◯ 66
◯ 67
◯ 68

◯ 69
◯ 70
◯ 71
◯ 72
◯ 73
◯ 74
◯ 75
◯ 76
◯ 77
◯ 78
◯ 79
◯ 80
◯ 81
◯ 82
◯ 83
◯ 84
◯ 85
◯ 86
◯ 87
◯ 88
◯ 89
◯ 90
◯ 91
◯ 92
◯ 93
◯ 94
◯ 95
◯ 96
◯ 97
◯ 98
◯ 99
◯ Don't know

At what age were you first told you had breast cancer?

◯ 0
◯ 1
◯ 2
◯ 3
◯ 4
◯ 5
◯ 6
◯ 7
◯ 8
◯ 9
◯ 10
◯ 11
◯ 12
◯ 13
◯ 14
◯ 15
◯ 16
◯ 17
◯ 18
◯ 19
◯ 20
◯ 21
◯ 22
◯ 23
◯ 24
◯ 25
◯ 26
◯ 27
◯ 28
◯ 29
◯ 30
◯ 31
◯ 32
◯ 33
◯ 34
◯ 35
◯ 36
◯ 37
◯ 38
◯ 39
◯ 40
◯ 41
◯ 42
◯ 43
◯ 44
◯ 45
◯ 46
◯ 47
◯ 48
◯ 49
◯ 50
◯ 51
◯ 52
◯ 53
◯ 54
◯ 55
◯ 56
◯ 57
◯ 58
◯ 59
◯ 60
◯ 61
◯ 62
◯ 63
◯ 64
◯ 65
◯ 66
◯ 67
◯ 68

- ◯ 69
- ◯ 70
- ◯ 71
- ◯ 72
- ◯ 73
- ◯ 74
- ◯ 75
- ◯ 76
- ◯ 77
- ◯ 78
- ◯ 79
- ◯ 80
- ◯ 81
- ◯ 82
- ◯ 83
- ◯ 84
- ◯ 85
- ◯ 86
- ◯ 87
- ◯ 88
- ◯ 89
- ◯ 90
- ◯ 91
- ◯ 92
- ◯ 93
- ◯ 94
- ◯ 95
- ◯ 96
- ◯ 97
- ◯ 98
- ◯ 99
- ◯ Don't know

At what age were you first told you had prostate cancer?

- ◯ 0
- ◯ 1
- ◯ 2
- ◯ 3
- ◯ 4
- ◯ 5
- ◯ 6
- ◯ 7
- ◯ 8
- ◯ 9
- ◯ 10
- ◯ 11
- ◯ 12
- ◯ 13
- ◯ 14
- ◯ 15
- ◯ 16
- ◯ 17
- ◯ 18
- ◯ 19
- ◯ 20
- ◯ 21
- ◯ 22
- ◯ 23
- ◯ 24
- ◯ 25
- ◯ 26
- ◯ 27
- ◯ 28
- ◯ 29
- ◯ 30
- ◯ 31
- ◯ 32
- ◯ 33
- ◯ 34
- ◯ 35
- ◯ 36
- ◯ 37
- ◯ 38
- ◯ 39
- ◯ 40
- ◯ 41
- ◯ 42
- ◯ 43
- ◯ 44
- ◯ 45
- ◯ 46
- ◯ 47
- ◯ 48
- ◯ 49
- ◯ 50
- ◯ 51
- ◯ 52
- ◯ 53
- ◯ 54
- ◯ 55
- ◯ 56
- ◯ 57
- ◯ 58
- ◯ 59
- ◯ 60
- ◯ 61
- ◯ 62
- ◯ 63
- ◯ 64
- ◯ 65
- ◯ 66
- ◯ 67
- ◯ 68

- ◯ 69
- ◯ 70
- ◯ 71
- ◯ 72
- ◯ 73
- ◯ 74
- ◯ 75
- ◯ 76
- ◯ 77
- ◯ 78
- ◯ 79
- ◯ 80
- ◯ 81
- ◯ 82
- ◯ 83
- ◯ 84
- ◯ 85
- ◯ 86
- ◯ 87
- ◯ 88
- ◯ 89
- ◯ 90
- ◯ 91
- ◯ 92
- ◯ 93
- ◯ 94
- ◯ 95
- ◯ 96
- ◯ 97
- ◯ 98
- ◯ 99
- ◯ Don't know

At what age were you first told you had lung cancer (include trachea, pleura and bronchus)?

◯ 0
◯ 1
◯ 2
◯ 3
◯ 4
◯ 5
◯ 6
◯ 7
◯ 8
◯ 9
◯ 10
◯ 11
◯ 12
◯ 13
◯ 14
◯ 15
◯ 16
◯ 17
◯ 18
◯ 19
◯ 20
◯ 21
◯ 22
◯ 23
◯ 24
◯ 25
◯ 26
◯ 27
◯ 28
◯ 29
◯ 30
◯ 31
◯ 32
◯ 33
◯ 34
◯ 35
◯ 36
◯ 37
◯ 38
◯ 39
◯ 40
◯ 41
◯ 42
◯ 43
◯ 44
◯ 45
◯ 46
◯ 47
◯ 48
◯ 49
◯ 50
◯ 51
◯ 52
◯ 53
◯ 54
◯ 55
◯ 56
◯ 57
◯ 58
◯ 59
◯ 60
◯ 61
◯ 62
◯ 63
◯ 64
◯ 65
◯ 66
◯ 67
◯ 68

◯ 69
◯ 70
◯ 71
◯ 72
◯ 73
◯ 74
◯ 75
◯ 76
◯ 77
◯ 78
◯ 79
◯ 80
◯ 81
◯ 82
◯ 83
◯ 84
◯ 85
◯ 86
◯ 87
◯ 88
◯ 89
◯ 90
◯ 91
◯ 92
◯ 93
◯ 94
◯ 95
◯ 96
◯ 97
◯ 98
◯ 99
◯ Don't know

At what age were you first told you had cervical cancer?

◯ 0
◯ 1
◯ 2
◯ 3
◯ 4
◯ 5
◯ 6
◯ 7
◯ 8
◯ 9
◯ 10
◯ 11
◯ 12
◯ 13
◯ 14
◯ 15
◯ 16
◯ 17
◯ 18
◯ 19
◯ 20
◯ 21
◯ 22
◯ 23
◯ 24
◯ 25
◯ 26
◯ 27
◯ 28
◯ 29
◯ 30
◯ 31
◯ 32
◯ 33
◯ 34
◯ 35
◯ 36
◯ 37
◯ 38
◯ 39
◯ 40
◯ 41
◯ 42
◯ 43
◯ 44
◯ 45
◯ 46
◯ 47
◯ 48
◯ 49
◯ 50
◯ 51
◯ 52
◯ 53
◯ 54
◯ 55
◯ 56
◯ 57
◯ 58
◯ 59
◯ 60
◯ 61
◯ 62
◯ 63
◯ 64
◯ 65
◯ 66
◯ 67
◯ 68

- ◯ 69
- ◯ 70
- ◯ 71
- ◯ 72
- ◯ 73
- ◯ 74
- ◯ 75
- ◯ 76
- ◯ 77
- ◯ 78
- ◯ 79
- ◯ 80
- ◯ 81
- ◯ 82
- ◯ 83
- ◯ 84
- ◯ 85
- ◯ 86
- ◯ 87
- ◯ 88
- ◯ 89
- ◯ 90
- ◯ 91
- ◯ 92
- ◯ 93
- ◯ 94
- ◯ 95
- ◯ 96
- ◯ 97
- ◯ 98
- ◯ 99
- ◯ Don't know

At what age were you first told you had cancer of other female reproductive organs (include uterus, ovary) ?

◯ 0
◯ 1
◯ 2
◯ 3
◯ 4
◯ 5
◯ 6
◯ 7
◯ 8
◯ 9
◯ 10
◯ 11
◯ 12
◯ 13
◯ 14
◯ 15
◯ 16
◯ 17
◯ 18
◯ 19
◯ 20
◯ 21
◯ 22
◯ 23
◯ 24
◯ 25
◯ 26
◯ 27
◯ 28
◯ 29
◯ 30
◯ 31
◯ 32
◯ 33
◯ 34
◯ 35
◯ 36
◯ 37
◯ 38
◯ 39
◯ 40
◯ 41
◯ 42
◯ 43
◯ 44
◯ 45
◯ 46
◯ 47
◯ 48
◯ 49
◯ 50
◯ 51
◯ 52
◯ 53
◯ 54
◯ 55
◯ 56
◯ 57
◯ 58
◯ 59
◯ 60
◯ 61
◯ 62
◯ 63
◯ 64
◯ 65
◯ 66
◯ 67
◯ 68

- ◯ 69
- ◯ 70
- ◯ 71
- ◯ 72
- ◯ 73
- ◯ 74
- ◯ 75
- ◯ 76
- ◯ 77
- ◯ 78
- ◯ 79
- ◯ 80
- ◯ 81
- ◯ 82
- ◯ 83
- ◯ 84
- ◯ 85
- ◯ 86
- ◯ 87
- ◯ 88
- ◯ 89
- ◯ 90
- ◯ 91
- ◯ 92
- ◯ 93
- ◯ 94
- ◯ 95
- ◯ 96
- ◯ 97
- ◯ 98
- ◯ 99
- ◯ Don't know

At what age were you first told you had bladder/kidney cancer?

◯ 0
◯ 1
◯ 2
◯ 3
◯ 4
◯ 5
◯ 6
◯ 7
◯ 8
◯ 9
◯ 10
◯ 11
◯ 12
◯ 13
◯ 14
◯ 15
◯ 16
◯ 17
◯ 18
◯ 19
◯ 20
◯ 21
◯ 22
◯ 23
◯ 24
◯ 25
◯ 26
◯ 27
◯ 28
◯ 29
◯ 30
◯ 31
◯ 32
◯ 33
◯ 34
◯ 35
◯ 36
◯ 37
◯ 38
◯ 39
◯ 40
◯ 41
◯ 42
◯ 43
◯ 44
◯ 45
◯ 46
◯ 47
◯ 48
◯ 49
◯ 50
◯ 51
◯ 52
◯ 53
◯ 54
◯ 55
◯ 56
◯ 57
◯ 58
◯ 59
◯ 60
◯ 61
◯ 62
◯ 63
◯ 64
◯ 65
◯ 66
◯ 67
◯ 68

◯ 69
◯ 70
◯ 71
◯ 72
◯ 73
◯ 74
◯ 75
◯ 76
◯ 77
◯ 78
◯ 79
◯ 80
◯ 81
◯ 82
◯ 83
◯ 84
◯ 85
◯ 86
◯ 87
◯ 88
◯ 89
◯ 90
◯ 91
◯ 92
◯ 93
◯ 94
◯ 95
◯ 96
◯ 97
◯ 98
◯ 99
◯ Don't know

At what age were you first told you had stomach cancer?

- ◯ 0
- ◯ 1
- ◯ 2
- ◯ 3
- ◯ 4
- ◯ 5
- ◯ 6
- ◯ 7
- ◯ 8
- ◯ 9
- ◯ 10
- ◯ 11
- ◯ 12
- ◯ 13
- ◯ 14
- ◯ 15
- ◯ 16
- ◯ 17
- ◯ 18
- ◯ 19
- ◯ 20
- ◯ 21
- ◯ 22
- ◯ 23
- ◯ 24
- ◯ 25
- ◯ 26
- ◯ 27
- ◯ 28
- ◯ 29
- ◯ 30
- ◯ 31
- ◯ 32
- ◯ 33
- ◯ 34
- ◯ 35
- ◯ 36
- ◯ 37
- ◯ 38
- ◯ 39
- ◯ 40
- ◯ 41
- ◯ 42
- ◯ 43
- ◯ 44
- ◯ 45
- ◯ 46
- ◯ 47
- ◯ 48
- ◯ 49
- ◯ 50
- ◯ 51
- ◯ 52
- ◯ 53
- ◯ 54
- ◯ 55
- ◯ 56
- ◯ 57
- ◯ 58
- ◯ 59
- ◯ 60
- ◯ 61
- ◯ 62
- ◯ 63
- ◯ 64
- ◯ 65
- ◯ 66
- ◯ 67
- ◯ 68

- ◯ 69
- ◯ 70
- ◯ 71
- ◯ 72
- ◯ 73
- ◯ 74
- ◯ 75
- ◯ 76
- ◯ 77
- ◯ 78
- ◯ 79
- ◯ 80
- ◯ 81
- ◯ 82
- ◯ 83
- ◯ 84
- ◯ 85
- ◯ 86
- ◯ 87
- ◯ 88
- ◯ 89
- ◯ 90
- ◯ 91
- ◯ 92
- ◯ 93
- ◯ 94
- ◯ 95
- ◯ 96
- ◯ 97
- ◯ 98
- ◯ 99
- ◯ Don't know

At what age were you first told you had leukaemia?

- ◯ 0
- ◯ 1
- ◯ 2
- ◯ 3
- ◯ 4
- ◯ 5
- ◯ 6
- ◯ 7
- ◯ 8
- ◯ 9
- ◯ 10
- ◯ 11
- ◯ 12
- ◯ 13
- ◯ 14
- ◯ 15
- ◯ 16
- ◯ 17
- ◯ 18
- ◯ 19
- ◯ 20
- ◯ 21
- ◯ 22
- ◯ 23
- ◯ 24
- ◯ 25
- ◯ 26
- ◯ 27
- ◯ 28
- ◯ 29
- ◯ 30
- ◯ 31
- ◯ 32
- ◯ 33
- ◯ 34
- ◯ 35
- ◯ 36
- ◯ 37
- ◯ 38
- ◯ 39
- ◯ 40
- ◯ 41
- ◯ 42
- ◯ 43
- ◯ 44
- ◯ 45
- ◯ 46
- ◯ 47
- ◯ 48
- ◯ 49
- ◯ 50
- ◯ 51
- ◯ 52
- ◯ 53
- ◯ 54
- ◯ 55
- ◯ 56
- ◯ 57
- ◯ 58
- ◯ 59
- ◯ 60
- ◯ 61
- ◯ 62
- ◯ 63
- ◯ 64
- ◯ 65
- ◯ 66
- ◯ 67
- ◯ 68

◯ 69
◯ 70
◯ 71
◯ 72
◯ 73
◯ 74
◯ 75
◯ 76
◯ 77
◯ 78
◯ 79
◯ 80
◯ 81
◯ 82
◯ 83
◯ 84
◯ 85
◯ 86
◯ 87
◯ 88
◯ 89
◯ 90
◯ 91
◯ 92
◯ 93
◯ 94
◯ 95
◯ 96
◯ 97
◯ 98
◯ 99
◯ Don't know

At what age were you first told you had non-Hodgkin lymphoma

◯ 0
◯ 1
◯ 2
◯ 3
◯ 4
◯ 5
◯ 6
◯ 7
◯ 8
◯ 9
◯ 10
◯ 11
◯ 12
◯ 13
◯ 14
◯ 15
◯ 16
◯ 17
◯ 18
◯ 19
◯ 20
◯ 21
◯ 22
◯ 23
◯ 24
◯ 25
◯ 26
◯ 27
◯ 28
◯ 29
◯ 30
◯ 31
◯ 32
◯ 33
◯ 34
◯ 35
◯ 36
◯ 37
◯ 38
◯ 39
◯ 40
◯ 41
◯ 42
◯ 43
◯ 44
◯ 45
◯ 46
◯ 47
◯ 48
◯ 49
◯ 50
◯ 51
◯ 52
◯ 53
◯ 54
◯ 55
◯ 56
◯ 57
◯ 58
◯ 59
◯ 60
◯ 61
◯ 62
◯ 63
◯ 64
◯ 65
◯ 66
◯ 67
◯ 68

- ◯ 69
- ◯ 70
- ◯ 71
- ◯ 72
- ◯ 73
- ◯ 74
- ◯ 75
- ◯ 76
- ◯ 77
- ◯ 78
- ◯ 79
- ◯ 80
- ◯ 81
- ◯ 82
- ◯ 83
- ◯ 84
- ◯ 85
- ◯ 86
- ◯ 87
- ◯ 88
- ◯ 89
- ◯ 90
- ◯ 91
- ◯ 92
- ◯ 93
- ◯ 94
- ◯ 95
- ◯ 96
- ◯ 97
- ◯ 98
- ◯ 99
- ◯ Don't know

At what age were you first told you had other type of lymphoma?

○ 0
○ 1
○ 2
○ 3
○ 4
○ 5
○ 6
○ 7
○ 8
○ 9
○ 10
○ 11
○ 12
○ 13
○ 14
○ 15
○ 16
○ 17
○ 18
○ 19
○ 20
○ 21
○ 22
○ 23
○ 24
○ 25
○ 26
○ 27
○ 28
○ 29
○ 30
○ 31
○ 32
○ 33
○ 34
○ 35
○ 36
○ 37
○ 38
○ 39
○ 40
○ 41
○ 42
○ 43
○ 44
○ 45
○ 46
○ 47
○ 48
○ 49
○ 50
○ 51
○ 52
○ 53
○ 54
○ 55
○ 56
○ 57
○ 58
○ 59
○ 60
○ 61
○ 62
○ 63
○ 64
○ 65
○ 66
○ 67
○ 68

- ◯ 69
- ◯ 70
- ◯ 71
- ◯ 72
- ◯ 73
- ◯ 74
- ◯ 75
- ◯ 76
- ◯ 77
- ◯ 78
- ◯ 79
- ◯ 80
- ◯ 81
- ◯ 82
- ◯ 83
- ◯ 84
- ◯ 85
- ◯ 86
- ◯ 87
- ◯ 88
- ◯ 89
- ◯ 90
- ◯ 91
- ◯ 92
- ◯ 93
- ◯ 94
- ◯ 95
- ◯ 96
- ◯ 97
- ◯ 98
- ◯ 99
- ◯ Don't know

At what age were you first told you had cancer of unknown primary site?

- ◯ 0
- ◯ 1
- ◯ 2
- ◯ 3
- ◯ 4
- ◯ 5
- ◯ 6
- ◯ 7
- ◯ 8
- ◯ 9
- ◯ 10
- ◯ 11
- ◯ 12
- ◯ 13
- ◯ 14
- ◯ 15
- ◯ 16
- ◯ 17
- ◯ 18
- ◯ 19
- ◯ 20
- ◯ 21
- ◯ 22
- ◯ 23
- ◯ 24
- ◯ 25
- ◯ 26
- ◯ 27
- ◯ 28
- ◯ 29
- ◯ 30
- ◯ 31
- ◯ 32
- ◯ 33
- ◯ 34
- ◯ 35
- ◯ 36
- ◯ 37
- ◯ 38
- ◯ 39
- ◯ 40
- ◯ 41
- ◯ 42
- ◯ 43
- ◯ 44
- ◯ 45
- ◯ 46
- ◯ 47
- ◯ 48
- ◯ 49
- ◯ 50
- ◯ 51
- ◯ 52
- ◯ 53
- ◯ 54
- ◯ 55
- ◯ 56
- ◯ 57
- ◯ 58
- ◯ 59
- ◯ 60
- ◯ 61
- ◯ 62
- ◯ 63
- ◯ 64
- ◯ 65
- ◯ 66
- ◯ 67
- ◯ 68

◯ 69
◯ 70
◯ 71
◯ 72
◯ 73
◯ 74
◯ 75
◯ 76
◯ 77
◯ 78
◯ 79
◯ 80
◯ 81
◯ 82
◯ 83
◯ 84
◯ 85
◯ 86
◯ 87
◯ 88
◯ 89
◯ 90
◯ 91
◯ 92
◯ 93
◯ 94
◯ 95
◯ 96
◯ 97
◯ 98
◯ 99
◯ Don't know

At what age were you first told you had [cancer_others]?

- ◯ 0
- ◯ 1
- ◯ 2
- ◯ 3
- ◯ 4
- ◯ 5
- ◯ 6
- ◯ 7
- ◯ 8
- ◯ 9
- ◯ 10
- ◯ 11
- ◯ 12
- ◯ 13
- ◯ 14
- ◯ 15
- ◯ 16
- ◯ 17
- ◯ 18
- ◯ 19
- ◯ 20
- ◯ 21
- ◯ 22
- ◯ 23
- ◯ 24
- ◯ 25
- ◯ 26
- ◯ 27
- ◯ 28
- ◯ 29
- ◯ 30
- ◯ 31
- ◯ 32
- ◯ 33
- ◯ 34
- ◯ 35
- ◯ 36
- ◯ 37
- ◯ 38
- ◯ 39
- ◯ 40
- ◯ 41
- ◯ 42
- ◯ 43
- ◯ 44
- ◯ 45
- ◯ 46
- ◯ 47
- ◯ 48
- ◯ 49
- ◯ 50
- ◯ 51
- ◯ 52
- ◯ 53
- ◯ 54
- ◯ 55
- ◯ 56
- ◯ 57
- ◯ 58
- ◯ 59
- ◯ 60
- ◯ 61
- ◯ 62
- ◯ 63
- ◯ 64
- ◯ 65
- ◯ 66
- ◯ 67
- ◯ 68

- ◯ 69
- ◯ 70
- ◯ 71
- ◯ 72
- ◯ 73
- ◯ 74
- ◯ 75
- ◯ 76
- ◯ 77
- ◯ 78
- ◯ 79
- ◯ 80
- ◯ 81
- ◯ 82
- ◯ 83
- ◯ 84
- ◯ 85
- ◯ 86
- ◯ 87
- ◯ 88
- ◯ 89
- ◯ 90
- ◯ 91
- ◯ 92
- ◯ 93
- ◯ 94
- ◯ 95
- ◯ 96
- ◯ 97
- ◯ 98
- ◯ 99
- ◯ Don't know

At what age were you first told you had this condition?

◯ 0
◯ 1
◯ 2
◯ 3
◯ 4
◯ 5
◯ 6
◯ 7
◯ 8
◯ 9
◯ 10
◯ 11
◯ 12
◯ 13
◯ 14
◯ 15
◯ 16
◯ 17
◯ 18
◯ 19
◯ 20
◯ 21
◯ 22
◯ 23
◯ 24
◯ 25
◯ 26
◯ 27
◯ 28
◯ 29
◯ 30
◯ 31
◯ 32
◯ 33
◯ 34
◯ 35
◯ 36
◯ 37
◯ 38
◯ 39
◯ 40
◯ 41
◯ 42
◯ 43
◯ 44
◯ 45
◯ 46
◯ 47
◯ 48
◯ 49
◯ 50
◯ 51
◯ 52
◯ 53
◯ 54
◯ 55
◯ 56
◯ 57
◯ 58
◯ 59
◯ 60
◯ 61
◯ 62
◯ 63
◯ 64
◯ 65
◯ 66
◯ 67
◯ 68

◯ 69
◯ 70
◯ 71
◯ 72
◯ 73
◯ 74
◯ 75
◯ 76
◯ 77
◯ 78
◯ 79
◯ 80
◯ 81
◯ 82
◯ 83
◯ 84
◯ 85
◯ 86
◯ 87
◯ 88
◯ 89
◯ 90
◯ 91
◯ 92
◯ 93
◯ 94
◯ 95
◯ 96
◯ 97
◯ 98
◯ 99
◯ Don't know

At what age were you first told you had [cancer_others_2]?

- ◯ 0
- ◯ 1
- ◯ 2
- ◯ 3
- ◯ 4
- ◯ 5
- ◯ 6
- ◯ 7
- ◯ 8
- ◯ 9
- ◯ 10
- ◯ 11
- ◯ 12
- ◯ 13
- ◯ 14
- ◯ 15
- ◯ 16
- ◯ 17
- ◯ 18
- ◯ 19
- ◯ 20
- ◯ 21
- ◯ 22
- ◯ 23
- ◯ 24
- ◯ 25
- ◯ 26
- ◯ 27
- ◯ 28
- ◯ 29
- ◯ 30
- ◯ 31
- ◯ 32
- ◯ 33
- ◯ 34
- ◯ 35
- ◯ 36
- ◯ 37
- ◯ 38
- ◯ 39
- ◯ 40
- ◯ 41
- ◯ 42
- ◯ 43
- ◯ 44
- ◯ 45
- ◯ 46
- ◯ 47
- ◯ 48
- ◯ 49
- ◯ 50
- ◯ 51
- ◯ 52
- ◯ 53
- ◯ 54
- ◯ 55
- ◯ 56
- ◯ 57
- ◯ 58
- ◯ 59
- ◯ 60
- ◯ 61
- ◯ 62
- ◯ 63
- ◯ 64
- ◯ 65
- ◯ 66
- ◯ 67
- ◯ 68

- ◯ 69
- ◯ 70
- ◯ 71
- ◯ 72
- ◯ 73
- ◯ 74
- ◯ 75
- ◯ 76
- ◯ 77
- ◯ 78
- ◯ 79
- ◯ 80
- ◯ 81
- ◯ 82
- ◯ 83
- ◯ 84
- ◯ 85
- ◯ 86
- ◯ 87
- ◯ 88
- ◯ 89
- ◯ 90
- ◯ 91
- ◯ 92
- ◯ 93
- ◯ 94
- ◯ 95
- ◯ 96
- ◯ 97
- ◯ 98
- ◯ 99
- ◯ Don't know

At what age were you first told you had [cancer_others_3]?

〇 0
〇 1
〇 2
〇 3
〇 4
〇 5
〇 6
〇 7
〇 8
〇 9
〇 10
〇 11
〇 12
〇 13
〇 14
〇 15
〇 16
〇 17
〇 18
〇 19
〇 20
〇 21
〇 22
〇 23
〇 24
〇 25
〇 26
〇 27
〇 28
〇 29
〇 30
〇 31
〇 32
〇 33
〇 34
〇 35
〇 36
〇 37
〇 38
〇 39
〇 40
〇 41
〇 42
〇 43
〇 44
〇 45
〇 46
〇 47
〇 48
〇 49
〇 50
〇 51
〇 52
〇 53
〇 54
〇 55
〇 56
〇 57
〇 58
〇 59
〇 60
〇 61
〇 62
〇 63
〇 64
〇 65
〇 66
〇 67
〇 68

	◯ 69<br>◯ 70<br>◯ 71<br>◯ 72<br>◯ 73<br>◯ 74<br>◯ 75<br>◯ 76<br>◯ 77<br>◯ 78<br>◯ 79<br>◯ 80<br>◯ 81<br>◯ 82<br>◯ 83<br>◯ 84<br>◯ 85<br>◯ 86<br>◯ 87<br>◯ 88<br>◯ 89<br>◯ 90<br>◯ 91<br>◯ 92<br>◯ 93<br>◯ 94<br>◯ 95<br>◯ 96<br>◯ 97<br>◯ 98<br>◯ 99<br>◯ Don't know
Do you still have Skin cancer (include melanoma, basal cell carcinoma, squamous cell carcinoma)?	◯ Yes<br>◯ No<br>◯ Don't know
Do you still have colon/rectum/bowel cancer (colorectal)?	◯ Yes<br>◯ No<br>◯ Don't know
Do you still have breast cancer?	◯ Yes<br>◯ No<br>◯ Don't know
Do you still have prostate cancer?	◯ Yes<br>◯ No<br>◯ Don't know
Do you still have lung cancer (include trachea, pleura and bronchus?	◯ Yes<br>◯ No<br>◯ Don't know
Do you still have cervical cancer?	◯ Yes<br>◯ No<br>◯ Don't know
Do you still have cancer of other female reproductive organs (include uterus, ovary)?	◯ Yes<br>◯ No<br>◯ Don't know

Do you still have bladder/kidney cancer?
- ◯ Yes
- ◯ No
- ◯ Don't know

Do you still have stomach cancer?
- ◯ Yes
- ◯ No
- ◯ Don't know

Do you still have leukaemia?
- ◯ Yes
- ◯ No
- ◯ Don't know

Do you still have non- Hodgkin leukaemia?
- ◯ Yes
- ◯ No
- ◯ Don't know

Do you still have other type of lymphoma?
- ◯ Yes
- ◯ No
- ◯ Don't know

Do you still have cancer of unknown primary site?
- ◯ Yes
- ◯ No
- ◯ Don't know

Do you still have [cancer_others]?
- ◯ Yes
- ◯ No
- ◯ Don't know

Do you still have this condition?
- ◯ Yes
- ◯ No
- ◯ Don't know

Do you still have [cancer_others_2]?
- ◯ Yes
- ◯ No
- ◯ Don't know

Do you still have [cancer_others_3]?
- ◯ Yes
- ◯ No
- ◯ Don't know

How was/is skin cancer (include melanoma, basal cell carcinoma, squamous cell carcinoma) treated? (Tick all that apply)

- ☐ no treatment
- ☐ diet
- ☐ exercise
- ☐ medication/chemotherapy
- ☐ natural/herbal therapy (please specify)
- ☐ radiotherapy
- ☐ supplements (please specify)
- ☐ surgery
- ☐ weight management
- ☐ other (please specify)
- ☐ Don't know

please specify the natural/herbal therapy

____

please specify the supplements

____

please specify the treatment

____

How was/is colon/rectum/bowel cancer (colorectal) treated?
(Tick all that apply)

☐ no treatment
☐ diet
☐ exercise
☐ medication/chemotherapy
☐ natural/herbal therapy (please specify)
☐ radiotherapy
☐ supplements (please specify)
☐ surgery
☐ weight management
☐ other (please specify)
☐ Don't know

please specify the natural/herbal therapy

____

please specify the supplements

____

please specify the treatment

____

How was/is breast cancer treated?
(Tick all that apply)

☐ no treatment
☐ diet
☐ exercise
☐ medication/chemotherapy
☐ natural/herbal therapy (please specify)
☐ radiotherapy
☐ supplements (please specify)
☐ surgery
☐ weight management
☐ other (please specify)
☐ Don't know

please specify the natural/herbal therapy

____

please specify the supplements

____

please specify the treatment

____

How was/is prostate cancer treated?
(Tick all that apply)

- ☐ no treatment
- ☐ diet
- ☐ exercise
- ☐ medication/chemotherapy
- ☐ natural/herbal therapy (please specify)
- ☐ radiotherapy
- ☐ supplements (please specify)
- ☐ surgery
- ☐ weight management
- ☐ other (please specify)
- ☐ Don't know

please specify the natural/herbal therapy ______________________

please specify the supplements ______________________

please specify the treatment ______________________

How was/is lung cancer (include trachea, pleura and bronchus) treated?
(Tick all that apply)

- ☐ no treatment
- ☐ diet
- ☐ exercise
- ☐ medication/chemotherapy
- ☐ natural/herbal therapy (please specify)
- ☐ radiotherapy
- ☐ supplements (please specify)
- ☐ surgery
- ☐ weight management
- ☐ other (please specify)
- ☐ Don't know

please specify the natural/herbal therapy ______________________

please specify the supplements ______________________

please specify the treatment ______________________

How was/is cervical cancer treated?
(Tick all that apply)

- ☐ no treatment
- ☐ diet
- ☐ exercise
- ☐ medication/chemotherapy
- ☐ natural/herbal therapy (please specify)
- ☐ radiotherapy
- ☐ supplements (please specify)
- ☐ surgery
- ☐ weight management
- ☐ other (please specify)
- ☐ Don't know

please specify the natural/herbal therapy ______________________________

please specify the supplements ______________________________

please specify the treatment ______________________________

How was/is cancer of other female reproductive organs (include uterus, ovary) treated?
(Tick all that apply)

- ☐ no treatment
- ☐ diet
- ☐ exercise
- ☐ medication/chemotherapy
- ☐ natural/herbal therapy (please specify)
- ☐ radiotherapy
- ☐ supplements (please specify)
- ☐ surgery
- ☐ weight management
- ☐ other (please specify)
- ☐ Don't know

please specify the natural/herbal therapy ______________________________

please specify the supplements ______________________________

please specify the treatment ______________________________

How was/is bladder/kidney cancer treated?

- [ ] no treatment
- [ ] diet
- [ ] exercise
- [ ] medication/chemotherapy
- [ ] natural/herbal therapy (please specify)
- [ ] radiotherapy
- [ ] supplements (please specify)
- [ ] surgery
- [ ] weight management
- [ ] other (please specify)
- [ ] Don't know

please specify the natural/herbal therapy

____

please specify the supplements

____

please specify the treatment

____

How was/is stomach cancer treated?
(Tick all that apply)

- [ ] no treatment
- [ ] diet
- [ ] exercise
- [ ] medication/chemotherapy
- [ ] natural/herbal therapy (please specify)
- [ ] radiotherapy
- [ ] supplements (please specify)
- [ ] surgery
- [ ] weight management
- [ ] other (please specify)
- [ ] Don't know

please specify the natural/herbal therapy

____

please specify the supplements

____

please specify the treatment

____

How was/is leukaemia treated?
(Tick all that apply)

- ☐ no treatment
- ☐ diet
- ☐ exercise
- ☐ medication/chemotherapy
- ☐ natural/herbal therapy (please specify)
- ☐ radiotherapy
- ☐ supplements (please specify)
- ☐ surgery
- ☐ weight management
- ☐ other (please specify)
- ☐ Don't know

please specify the natural/herbal therapy

____

please specify the supplements

____

please specify the treatment

____

How was/is non-Hodgkin lymphoma treated?
(Tick all that apply)

- ☐ no treatment
- ☐ diet
- ☐ exercise
- ☐ medication/chemotherapy
- ☐ natural/herbal therapy (please specify)
- ☐ radiotherapy
- ☐ supplements (please specify)
- ☐ surgery
- ☐ weight management
- ☐ other (please specify)
- ☐ Don't know

please specify the natural/herbal therapy

____

please specify the supplements

____

please specify the treatment

____

How was/is other type of lymphoma treated?
(Tick all that apply)

- ☐ no treatment
- ☐ diet
- ☐ exercise
- ☐ medication/chemotherapy
- ☐ natural/herbal therapy (please specify)
- ☐ radiotherapy
- ☐ supplements (please specify)
- ☐ surgery
- ☐ weight management
- ☐ other (please specify)
- ☐ Don't know

please specify the natural/herbal therapy

____

please specify the supplements

____

please specify the treatment

____

How was/is cancer of unknown primary site treated?
(Tick all that apply)

- ☐ no treatment
- ☐ diet
- ☐ exercise
- ☐ medication/chemotherapy
- ☐ natural/herbal therapy (please specify)
- ☐ radiotherapy
- ☐ supplements (please specify)
- ☐ surgery
- ☐ weight management
- ☐ other (please specify)
- ☐ Don't know

please specify the natural/herbal therapy

____

please specify the supplements

____

please specify the treatment

____

How was/is [cancer_others] treated?
(Tick all that apply)

☐ no treatment
☐ diet
☐ exercise
☐ medication/chemotherapy
☐ natural/herbal therapy (please specify)
☐ radiotherapy
☐ supplements (please specify)
☐ surgery
☐ weight management
☐ other (please specify)
☐ Don't know

please specify the natural/herbal therapy ____________________

please specify the supplements ____________________

please specify the treatment ____________________

How was/is this condition treated?
(Tick all that apply)

☐ no treatment
☐ diet
☐ exercise
☐ medication/chemotherapy
☐ natural/herbal therapy (please specify)
☐ radiotherapy
☐ supplements (please specify)
☐ surgery
☐ weight management
☐ other (please specify)
☐ Don't know

please specify the natural/herbal therapy ____________________

please specify the supplements ____________________

please specify the treatment ____________________

please specify the treatment ____________________

How was/is [cancer_others_2] treated?
(Tick all that apply)

- ☐ no treatment
- ☐ diet
- ☐ exercise
- ☐ medication/chemotherapy
- ☐ natural/herbal therapy (please specify)
- ☐ radiotherapy
- ☐ supplements (please specify)
- ☐ surgery
- ☐ weight management
- ☐ other (please specify)
- ☐ Don't know

please specify the natural/herbal therapy ______________________

please specify the supplements ______________________

please specify the treatment ______________________

How was/is [cancer_others_3] treated?
(Tick all that apply)

- ☐ no treatment
- ☐ diet
- ☐ exercise
- ☐ medication/chemotherapy
- ☐ natural/herbal therapy (please specify)
- ☐ radiotherapy
- ☐ supplements (please specify)
- ☐ surgery
- ☐ weight management
- ☐ other (please specify)
- ☐ Don't know

please specify the natural/herbal therapy ______________________

please specify the supplements ______________________

please specify the treatment ______________________

Have either of your parents, or any of brothers or sisters ever been told by a doctor (GP or specialist) or nurse that they have any type of cancer?

- ◯ Yes
- ◯ No
- ◯ Don't know

This question is about your mental and emotional well-being. Have you ever been told by a doctor (GP or specialist) or psychologist that you have depression, an anxiety condition, or memory loss?

- ◯ Yes
- ◯ No
- ◯ Don't know
- ◯ Prefer not to say

Which condition/s have been confirmed by a doctor (GP or specialist) or psychologist?
(Tick all that apply)

- ☐ Alzheimer's Disease
- ☐ Anxiety condition (such as post-traumatic stress disorder, panic attacks, obsessive compulsive disorder, phobias)
- ☐ Dementia
- ☐ Depression (including post-natal depression)
- ☐ Mild cognitive impairment (MCI)
- ☐ Other anxiety condition
- ☐ Other mood condition
- ☐ Other (please specify)
- ☐ Don't Know

please specify the condition

____

Do you have another mental health or emotional health condition that needs to be specified?

◯ Yes ◯ No

please specify the second condition

____

Do you have another mental health or emotional health condition that needs to be specified?

◯ Yes ◯ No

please specify the third condition

____

At what age were you first told you had Alzheimer's disease?

◯ 0
◯ 1
◯ 2
◯ 3
◯ 4
◯ 5
◯ 6
◯ 7
◯ 8
◯ 9
◯ 10
◯ 11
◯ 12
◯ 13
◯ 14
◯ 15
◯ 16
◯ 17
◯ 18
◯ 19
◯ 20
◯ 21
◯ 22
◯ 23
◯ 24
◯ 25
◯ 26
◯ 27
◯ 28
◯ 29
◯ 30
◯ 31
◯ 32
◯ 33
◯ 34
◯ 35
◯ 36
◯ 37
◯ 38
◯ 39
◯ 40
◯ 41
◯ 42
◯ 43
◯ 44
◯ 45
◯ 46
◯ 47
◯ 48
◯ 49
◯ 50
◯ 51
◯ 52
◯ 53
◯ 54
◯ 55
◯ 56
◯ 57
◯ 58
◯ 59
◯ 60
◯ 61
◯ 62
◯ 63
◯ 64
◯ 65
◯ 66
◯ 67
◯ 68

- ◯ 69
- ◯ 70
- ◯ 71
- ◯ 72
- ◯ 73
- ◯ 74
- ◯ 75
- ◯ 76
- ◯ 77
- ◯ 78
- ◯ 79
- ◯ 80
- ◯ 81
- ◯ 82
- ◯ 83
- ◯ 84
- ◯ 85
- ◯ 86
- ◯ 87
- ◯ 88
- ◯ 89
- ◯ 90
- ◯ 91
- ◯ 92
- ◯ 93
- ◯ 94
- ◯ 95
- ◯ 96
- ◯ 97
- ◯ 98
- ◯ 99
- ◯ Don't know

At what age were you first told you had anxiety (post-traumatic stress disorder, panic attacks, obsessive compulsive disorder, phobias)?

- ◯ 0
- ◯ 1
- ◯ 2
- ◯ 3
- ◯ 4
- ◯ 5
- ◯ 6
- ◯ 7
- ◯ 8
- ◯ 9
- ◯ 10
- ◯ 11
- ◯ 12
- ◯ 13
- ◯ 14
- ◯ 15
- ◯ 16
- ◯ 17
- ◯ 18
- ◯ 19
- ◯ 20
- ◯ 21
- ◯ 22
- ◯ 23
- ◯ 24
- ◯ 25
- ◯ 26
- ◯ 27
- ◯ 28
- ◯ 29
- ◯ 30
- ◯ 31
- ◯ 32
- ◯ 33
- ◯ 34
- ◯ 35
- ◯ 36
- ◯ 37
- ◯ 38
- ◯ 39
- ◯ 40
- ◯ 41
- ◯ 42
- ◯ 43
- ◯ 44
- ◯ 45
- ◯ 46
- ◯ 47
- ◯ 48
- ◯ 49
- ◯ 50
- ◯ 51
- ◯ 52
- ◯ 53
- ◯ 54
- ◯ 55
- ◯ 56
- ◯ 57
- ◯ 58
- ◯ 59
- ◯ 60
- ◯ 61
- ◯ 62
- ◯ 63
- ◯ 64
- ◯ 65
- ◯ 66
- ◯ 67
- ◯ 68

◯ 69
◯ 70
◯ 71
◯ 72
◯ 73
◯ 74
◯ 75
◯ 76
◯ 77
◯ 78
◯ 79
◯ 80
◯ 81
◯ 82
◯ 83
◯ 84
◯ 85
◯ 86
◯ 87
◯ 88
◯ 89
◯ 90
◯ 91
◯ 92
◯ 93
◯ 94
◯ 95
◯ 96
◯ 97
◯ 98
◯ 99
◯ Don't know

At what age were you first told you had dementia?

- ◯ 0
- ◯ 1
- ◯ 2
- ◯ 3
- ◯ 4
- ◯ 5
- ◯ 6
- ◯ 7
- ◯ 8
- ◯ 9
- ◯ 10
- ◯ 11
- ◯ 12
- ◯ 13
- ◯ 14
- ◯ 15
- ◯ 16
- ◯ 17
- ◯ 18
- ◯ 19
- ◯ 20
- ◯ 21
- ◯ 22
- ◯ 23
- ◯ 24
- ◯ 25
- ◯ 26
- ◯ 27
- ◯ 28
- ◯ 29
- ◯ 30
- ◯ 31
- ◯ 32
- ◯ 33
- ◯ 34
- ◯ 35
- ◯ 36
- ◯ 37
- ◯ 38
- ◯ 39
- ◯ 40
- ◯ 41
- ◯ 42
- ◯ 43
- ◯ 44
- ◯ 45
- ◯ 46
- ◯ 47
- ◯ 48
- ◯ 49
- ◯ 50
- ◯ 51
- ◯ 52
- ◯ 53
- ◯ 54
- ◯ 55
- ◯ 56
- ◯ 57
- ◯ 58
- ◯ 59
- ◯ 60
- ◯ 61
- ◯ 62
- ◯ 63
- ◯ 64
- ◯ 65
- ◯ 66
- ◯ 67
- ◯ 68

◯ 69
◯ 70
◯ 71
◯ 72
◯ 73
◯ 74
◯ 75
◯ 76
◯ 77
◯ 78
◯ 79
◯ 80
◯ 81
◯ 82
◯ 83
◯ 84
◯ 85
◯ 86
◯ 87
◯ 88
◯ 89
◯ 90
◯ 91
◯ 92
◯ 93
◯ 94
◯ 95
◯ 96
◯ 97
◯ 98
◯ 99
◯ Don't know

At what age were you first told you had depression (including post-natal depression)?

- ◯ 0
- ◯ 1
- ◯ 2
- ◯ 3
- ◯ 4
- ◯ 5
- ◯ 6
- ◯ 7
- ◯ 8
- ◯ 9
- ◯ 10
- ◯ 11
- ◯ 12
- ◯ 13
- ◯ 14
- ◯ 15
- ◯ 16
- ◯ 17
- ◯ 18
- ◯ 19
- ◯ 20
- ◯ 21
- ◯ 22
- ◯ 23
- ◯ 24
- ◯ 25
- ◯ 26
- ◯ 27
- ◯ 28
- ◯ 29
- ◯ 30
- ◯ 31
- ◯ 32
- ◯ 33
- ◯ 34
- ◯ 35
- ◯ 36
- ◯ 37
- ◯ 38
- ◯ 39
- ◯ 40
- ◯ 41
- ◯ 42
- ◯ 43
- ◯ 44
- ◯ 45
- ◯ 46
- ◯ 47
- ◯ 48
- ◯ 49
- ◯ 50
- ◯ 51
- ◯ 52
- ◯ 53
- ◯ 54
- ◯ 55
- ◯ 56
- ◯ 57
- ◯ 58
- ◯ 59
- ◯ 60
- ◯ 61
- ◯ 62
- ◯ 63
- ◯ 64
- ◯ 65
- ◯ 66
- ◯ 67
- ◯ 68

- ◯ 69
- ◯ 70
- ◯ 71
- ◯ 72
- ◯ 73
- ◯ 74
- ◯ 75
- ◯ 76
- ◯ 77
- ◯ 78
- ◯ 79
- ◯ 80
- ◯ 81
- ◯ 82
- ◯ 83
- ◯ 84
- ◯ 85
- ◯ 86
- ◯ 87
- ◯ 88
- ◯ 89
- ◯ 90
- ◯ 91
- ◯ 92
- ◯ 93
- ◯ 94
- ◯ 95
- ◯ 96
- ◯ 97
- ◯ 98
- ◯ 99
- ◯ Don't know

At what age were you first told you had Mild cognitive impairment (MCI)?

- ◯ 0
- ◯ 1
- ◯ 2
- ◯ 3
- ◯ 4
- ◯ 5
- ◯ 6
- ◯ 7
- ◯ 8
- ◯ 9
- ◯ 10
- ◯ 11
- ◯ 12
- ◯ 13
- ◯ 14
- ◯ 15
- ◯ 16
- ◯ 17
- ◯ 18
- ◯ 19
- ◯ 20
- ◯ 21
- ◯ 22
- ◯ 23
- ◯ 24
- ◯ 25
- ◯ 26
- ◯ 27
- ◯ 28
- ◯ 29
- ◯ 30
- ◯ 31
- ◯ 32
- ◯ 33
- ◯ 34
- ◯ 35
- ◯ 36
- ◯ 37
- ◯ 38
- ◯ 39
- ◯ 40
- ◯ 41
- ◯ 42
- ◯ 43
- ◯ 44
- ◯ 45
- ◯ 46
- ◯ 47
- ◯ 48
- ◯ 49
- ◯ 50
- ◯ 51
- ◯ 52
- ◯ 53
- ◯ 54
- ◯ 55
- ◯ 56
- ◯ 57
- ◯ 58
- ◯ 59
- ◯ 60
- ◯ 61
- ◯ 62
- ◯ 63
- ◯ 64
- ◯ 65
- ◯ 66
- ◯ 67
- ◯ 68

- ◯ 69
- ◯ 70
- ◯ 71
- ◯ 72
- ◯ 73
- ◯ 74
- ◯ 75
- ◯ 76
- ◯ 77
- ◯ 78
- ◯ 79
- ◯ 80
- ◯ 81
- ◯ 82
- ◯ 83
- ◯ 84
- ◯ 85
- ◯ 86
- ◯ 87
- ◯ 88
- ◯ 89
- ◯ 90
- ◯ 91
- ◯ 92
- ◯ 93
- ◯ 94
- ◯ 95
- ◯ 96
- ◯ 97
- ◯ 98
- ◯ 99
- ◯ Don't know

At what age were you first told you had the anxiety condition?

- ◯ 0
- ◯ 1
- ◯ 2
- ◯ 3
- ◯ 4
- ◯ 5
- ◯ 6
- ◯ 7
- ◯ 8
- ◯ 9
- ◯ 10
- ◯ 11
- ◯ 12
- ◯ 13
- ◯ 14
- ◯ 15
- ◯ 16
- ◯ 17
- ◯ 18
- ◯ 19
- ◯ 20
- ◯ 21
- ◯ 22
- ◯ 23
- ◯ 24
- ◯ 25
- ◯ 26
- ◯ 27
- ◯ 28
- ◯ 29
- ◯ 30
- ◯ 31
- ◯ 32
- ◯ 33
- ◯ 34
- ◯ 35
- ◯ 36
- ◯ 37
- ◯ 38
- ◯ 39
- ◯ 40
- ◯ 41
- ◯ 42
- ◯ 43
- ◯ 44
- ◯ 45
- ◯ 46
- ◯ 47
- ◯ 48
- ◯ 49
- ◯ 50
- ◯ 51
- ◯ 52
- ◯ 53
- ◯ 54
- ◯ 55
- ◯ 56
- ◯ 57
- ◯ 58
- ◯ 59
- ◯ 60
- ◯ 61
- ◯ 62
- ◯ 63
- ◯ 64
- ◯ 65
- ◯ 66
- ◯ 67
- ◯ 68

◯ 69
◯ 70
◯ 71
◯ 72
◯ 73
◯ 74
◯ 75
◯ 76
◯ 77
◯ 78
◯ 79
◯ 80
◯ 81
◯ 82
◯ 83
◯ 84
◯ 85
◯ 86
◯ 87
◯ 88
◯ 89
◯ 90
◯ 91
◯ 92
◯ 93
◯ 94
◯ 95
◯ 96
◯ 97
◯ 98
◯ 99
◯ Don't know

At what age were you first told you had the mood condition?

- ◯ 0
- ◯ 1
- ◯ 2
- ◯ 3
- ◯ 4
- ◯ 5
- ◯ 6
- ◯ 7
- ◯ 8
- ◯ 9
- ◯ 10
- ◯ 11
- ◯ 12
- ◯ 13
- ◯ 14
- ◯ 15
- ◯ 16
- ◯ 17
- ◯ 18
- ◯ 19
- ◯ 20
- ◯ 21
- ◯ 22
- ◯ 23
- ◯ 24
- ◯ 25
- ◯ 26
- ◯ 27
- ◯ 28
- ◯ 29
- ◯ 30
- ◯ 31
- ◯ 32
- ◯ 33
- ◯ 34
- ◯ 35
- ◯ 36
- ◯ 37
- ◯ 38
- ◯ 39
- ◯ 40
- ◯ 41
- ◯ 42
- ◯ 43
- ◯ 44
- ◯ 45
- ◯ 46
- ◯ 47
- ◯ 48
- ◯ 49
- ◯ 50
- ◯ 51
- ◯ 52
- ◯ 53
- ◯ 54
- ◯ 55
- ◯ 56
- ◯ 57
- ◯ 58
- ◯ 59
- ◯ 60
- ◯ 61
- ◯ 62
- ◯ 63
- ◯ 64
- ◯ 65
- ◯ 66
- ◯ 67
- ◯ 68

- ◯ 69
- ◯ 70
- ◯ 71
- ◯ 72
- ◯ 73
- ◯ 74
- ◯ 75
- ◯ 76
- ◯ 77
- ◯ 78
- ◯ 79
- ◯ 80
- ◯ 81
- ◯ 82
- ◯ 83
- ◯ 84
- ◯ 85
- ◯ 86
- ◯ 87
- ◯ 88
- ◯ 89
- ◯ 90
- ◯ 91
- ◯ 92
- ◯ 93
- ◯ 94
- ◯ 95
- ◯ 96
- ◯ 97
- ◯ 98
- ◯ 99
- ◯ Don't know

At what age were you first told you had [mental_others]?

〇 0
〇 1
〇 2
〇 3
〇 4
〇 5
〇 6
〇 7
〇 8
〇 9
〇 10
〇 11
〇 12
〇 13
〇 14
〇 15
〇 16
〇 17
〇 18
〇 19
〇 20
〇 21
〇 22
〇 23
〇 24
〇 25
〇 26
〇 27
〇 28
〇 29
〇 30
〇 31
〇 32
〇 33
〇 34
〇 35
〇 36
〇 37
〇 38
〇 39
〇 40
〇 41
〇 42
〇 43
〇 44
〇 45
〇 46
〇 47
〇 48
〇 49
〇 50
〇 51
〇 52
〇 53
〇 54
〇 55
〇 56
〇 57
〇 58
〇 59
〇 60
〇 61
〇 62
〇 63
〇 64
〇 65
〇 66
〇 67
〇 68

- ◯ 69
- ◯ 70
- ◯ 71
- ◯ 72
- ◯ 73
- ◯ 74
- ◯ 75
- ◯ 76
- ◯ 77
- ◯ 78
- ◯ 79
- ◯ 80
- ◯ 81
- ◯ 82
- ◯ 83
- ◯ 84
- ◯ 85
- ◯ 86
- ◯ 87
- ◯ 88
- ◯ 89
- ◯ 90
- ◯ 91
- ◯ 92
- ◯ 93
- ◯ 94
- ◯ 95
- ◯ 96
- ◯ 97
- ◯ 98
- ◯ 99
- ◯ Don't know

At what age were you first told you [mental_others_2]?

◯ 0
◯ 1
◯ 2
◯ 3
◯ 4
◯ 5
◯ 6
◯ 7
◯ 8
◯ 9
◯ 10
◯ 11
◯ 12
◯ 13
◯ 14
◯ 15
◯ 16
◯ 17
◯ 18
◯ 19
◯ 20
◯ 21
◯ 22
◯ 23
◯ 24
◯ 25
◯ 26
◯ 27
◯ 28
◯ 29
◯ 30
◯ 31
◯ 32
◯ 33
◯ 34
◯ 35
◯ 36
◯ 37
◯ 38
◯ 39
◯ 40
◯ 41
◯ 42
◯ 43
◯ 44
◯ 45
◯ 46
◯ 47
◯ 48
◯ 49
◯ 50
◯ 51
◯ 52
◯ 53
◯ 54
◯ 55
◯ 56
◯ 57
◯ 58
◯ 59
◯ 60
◯ 61
◯ 62
◯ 63
◯ 64
◯ 65
◯ 66
◯ 67
◯ 68

◯ 69
◯ 70
◯ 71
◯ 72
◯ 73
◯ 74
◯ 75
◯ 76
◯ 77
◯ 78
◯ 79
◯ 80
◯ 81
◯ 82
◯ 83
◯ 84
◯ 85
◯ 86
◯ 87
◯ 88
◯ 89
◯ 90
◯ 91
◯ 92
◯ 93
◯ 94
◯ 95
◯ 96
◯ 97
◯ 98
◯ 99
◯ Don't know

At what age were you first told you [mental_others_3]?

〇 0
〇 1
〇 2
〇 3
〇 4
〇 5
〇 6
〇 7
〇 8
〇 9
〇 10
〇 11
〇 12
〇 13
〇 14
〇 15
〇 16
〇 17
〇 18
〇 19
〇 20
〇 21
〇 22
〇 23
〇 24
〇 25
〇 26
〇 27
〇 28
〇 29
〇 30
〇 31
〇 32
〇 33
〇 34
〇 35
〇 36
〇 37
〇 38
〇 39
〇 40
〇 41
〇 42
〇 43
〇 44
〇 45
〇 46
〇 47
〇 48
〇 49
〇 50
〇 51
〇 52
〇 53
〇 54
〇 55
〇 56
〇 57
〇 58
〇 59
〇 60
〇 61
〇 62
〇 63
〇 64
〇 65
〇 66
〇 67
〇 68

- ◯ 69
- ◯ 70
- ◯ 71
- ◯ 72
- ◯ 73
- ◯ 74
- ◯ 75
- ◯ 76
- ◯ 77
- ◯ 78
- ◯ 79
- ◯ 80
- ◯ 81
- ◯ 82
- ◯ 83
- ◯ 84
- ◯ 85
- ◯ 86
- ◯ 87
- ◯ 88
- ◯ 89
- ◯ 90
- ◯ 91
- ◯ 92
- ◯ 93
- ◯ 94
- ◯ 95
- ◯ 96
- ◯ 97
- ◯ 98
- ◯ 99
- ◯ Don't know

At what age were you first told you had this condition?

◯ 0
◯ 1
◯ 2
◯ 3
◯ 4
◯ 5
◯ 6
◯ 7
◯ 8
◯ 9
◯ 10
◯ 11
◯ 12
◯ 13
◯ 14
◯ 15
◯ 16
◯ 17
◯ 18
◯ 19
◯ 20
◯ 21
◯ 22
◯ 23
◯ 24
◯ 25
◯ 26
◯ 27
◯ 28
◯ 29
◯ 30
◯ 31
◯ 32
◯ 33
◯ 34
◯ 35
◯ 36
◯ 37
◯ 38
◯ 39
◯ 40
◯ 41
◯ 42
◯ 43
◯ 44
◯ 45
◯ 46
◯ 47
◯ 48
◯ 49
◯ 50
◯ 51
◯ 52
◯ 53
◯ 54
◯ 55
◯ 56
◯ 57
◯ 58
◯ 59
◯ 60
◯ 61
◯ 62
◯ 63
◯ 64
◯ 65
◯ 66
◯ 67
◯ 68

	◯ 69<br>◯ 70<br>◯ 71<br>◯ 72<br>◯ 73<br>◯ 74<br>◯ 75<br>◯ 76<br>◯ 77<br>◯ 78<br>◯ 79<br>◯ 80<br>◯ 81<br>◯ 82<br>◯ 83<br>◯ 84<br>◯ 85<br>◯ 86<br>◯ 87<br>◯ 88<br>◯ 89<br>◯ 90<br>◯ 91<br>◯ 92<br>◯ 93<br>◯ 94<br>◯ 95<br>◯ 96<br>◯ 97<br>◯ 98<br>◯ 99<br>◯ Don't know
Do you still have depression (including post-natal depression)?	◯ Yes<br>◯ No<br>◯ Don't know
Do you still have this mood condition?	◯ Yes<br>◯ No<br>◯ Don't know
Do you still have anxiety (post-traumatic stress disorder, panic attacks, obsessive compulsive disorder, phobias)?	◯ Yes<br>◯ No<br>◯ Don't know
Do you still have this anxiety condition?	◯ Yes<br>◯ No<br>◯ Don't know
Do you still have Mild cognitive impairement (MCI)?	◯ Yes<br>◯ No<br>◯ Don't know
Do you still have dementia?	◯ Yes<br>◯ No<br>◯ Don't know
Do you still have Alzheimer's disease?	◯ Yes<br>◯ No<br>◯ Don't know

Do you still have [mental_others]?
◯ Yes
◯ No
◯ Don't know

Do you still have this condition?
◯ Yes
◯ No
◯ Don't know

Do you still have [mental_others_2]?
◯ Yes
◯ No
◯ Don't know

Do you still have [mental_others_3]?
◯ Yes
◯ No
◯ Don't know

How is depression (including post-natal depression) treated?
(Tick all that apply)

☐ no treatment ☐ diet ☐ exercise ☐ medication (please specify) ☐ natural/herbal therapy (please specify) ☐ weight management ☐ supplements (please specify) ☐ other (please specify)
☐ Don't know

please specify the medication
____

please specify the natural/herbal therapy
____

please specify the supplements
____

please specify the treatment
____

How is this mood condition treated?
(Tick all that apply)

☐ no treatment ☐ diet ☐ exercise ☐ medication (please specify) ☐ natural/herbal therapy (please specify) ☐ weight management ☐ supplements (please specify) ☐ other (please specify)
☐ Don't know

please specify the medication
____

please specify the natural/herbal therapy
____

please specify the supplements
____

please specify the treatment
____

How is anxiety condition (such as post-traumatic stress disorder, panic attacks, obsessive compulsive disorder, phobias) treated?
(Tick all that apply)

☐ no treatment ☐ diet ☐ exercise ☐ medication (please specify) ☐ natural/herbal therapy (please specify) ☐ weight management ☐ supplements (please specify) ☐ other (please specify)
☐ Don't know

please specify the medication ____________________

please specify the natural/herbal therapy ____________________

please specify the supplements ____________________

please specify the treatment ____________________

How is this anxiety condition treated?
(Tick all that apply)

☐ no treatment ☐ diet ☐ exercise ☐ medication (please specify) ☐ natural/herbal therapy (please specify) ☐ weight management ☐ supplements (please specify) ☐ other (please specify)
☐ Don't know

please specify the medication ____________________

please specify the natural/herbal therapy ____________________

please specify the supplements ____________________

please specify the treatment ____________________

How is mild cognitive impairment (MCI) treated?
(Tick all that apply)

☐ no treatment ☐ diet ☐ exercise ☐ medication (please specify) ☐ natural/herbal therapy (please specify) ☐ weight management ☐ supplements (please specify) ☐ other (please specify)
☐ Don't know

please specify the medication ____________________

please specify the natural/herbal therapy ____________________

please specify the supplements ____________________

please specify the treatment ____________________

How is dimentia treated?
(Tick all that apply)

☐ no treatment ☐ diet ☐ exercise ☐ medication (please specify) ☐ natural/herbal therapy (please specify) ☐ weight management ☐ supplements (please specify) ☐ other (please specify)
☐ Don't know

please specify the medication

____

please specify the natural/herbal therapy

____

please specify the supplements

____

please specify the treatment

____

How is Alzhiemer's Disease treated?
(Tick all that apply)

☐ no treatment ☐ diet ☐ exercise ☐ medication (please specify) ☐ natural/herbal therapy (please specify) ☐ weight management ☐ supplements (please specify) ☐ other (please specify)
☐ Don't know

please specify the medication

____

please specify the natural/herbal therapy

____

please specify the supplements

____

please specify the treatment

____

How is [mental_others] treated?
(Tick all that apply)

☐ no treatment ☐ diet ☐ exercise ☐ medication (please specify) ☐ natural/herbal therapy (please specify) ☐ weight management ☐ supplements (please specify) ☐ other (please specify)
☐ Don't know

please specify the medication

____

please specify the natural/herbal therapy

____

please specify the supplements

____

please specify the treatment

____

How is [mental_others_2] treated?
(Tick all that apply)

☐ no treatment ☐ diet ☐ exercise ☐ medication (please specify) ☐ natural/herbal therapy (please specify) ☐ weight management ☐ supplements (please specify) ☐ other (please specify)
☐ Don't know

please specify the medication

____

please specify the natural/herbal therapy

____

please specify the supplements

____

please specify the treatment

____

How is [mental_others_2] treated?
(Tick all that apply)

☐ no treatment ☐ diet ☐ exercise ☐ medication (please specify) ☐ natural/herbal therapy (please specify) ☐ weight management ☐ supplements (please specify) ☐ other (please specify)
☐ Don't know

please specify the medication

____

please specify the natural/herbal therapy

____

please specify the supplements

____

please specify the treatment

____

How is this condition treated?
(Tick all that apply)

☐ no treatment ☐ diet ☐ exercise ☐ medication (please specify) ☐ natural/herbal therapy (please specify) ☐ weight management ☐ supplements (please specify) ☐ other (please specify)
☐ Don't know

please specify the medication

____

please specify the natural/herbal therapy

____

please specify the supplements

____

please specify the treatment

____

Have either of your parents, or any of brothers or sisters ever been told by a doctor (GP or specialist) or psychologist that they have any depression, an anxiety condition, or memory loss?

◯ Yes
◯ No
◯ Don't know

This question is about past or present eating disorders. Have you ever been told by a doctor (GP or specialist), dietitian, or psychologist that you have an eating disorder?

◯ Yes ◯ No ◯ Don't know
◯ Prefer not to say

Which condition/s have been confirmed by a doctor (GP or specialist), dietitian, or psychologist?
(Tick all that apply)

☐ Anorexia nervosa ☐ Binge eating disorder ☐ Bulemia nervosa ☐ Other (please specify)
☐ Don't Know

please specify the condition

____

Do you have another eating disorder condition need to be specified?

◯ Yes ◯ No

please specify the second condition

____

Do you have another eating disorder condition need to be specified?

◯ Yes ◯ No

please specify the third condition

____

At what age were you first told you had anorexia nervosa?

◯ 0
◯ 1
◯ 2
◯ 3
◯ 4
◯ 5
◯ 6
◯ 7
◯ 8
◯ 9
◯ 10
◯ 11
◯ 12
◯ 13
◯ 14
◯ 15
◯ 16
◯ 17
◯ 18
◯ 19
◯ 20
◯ 21
◯ 22
◯ 23
◯ 24
◯ 25
◯ 26
◯ 27
◯ 28
◯ 29
◯ 30
◯ 31
◯ 32
◯ 33
◯ 34
◯ 35
◯ 36
◯ 37
◯ 38
◯ 39
◯ 40
◯ 41
◯ 42
◯ 43
◯ 44
◯ 45
◯ 46
◯ 47
◯ 48
◯ 49
◯ 50
◯ 51
◯ 52
◯ 53
◯ 54
◯ 55
◯ 56
◯ 57
◯ 58
◯ 59
◯ 60
◯ 61
◯ 62
◯ 63
◯ 64
◯ 65
◯ 66
◯ 67
◯ 68

- ◯ 69
- ◯ 70
- ◯ 71
- ◯ 72
- ◯ 73
- ◯ 74
- ◯ 75
- ◯ 76
- ◯ 77
- ◯ 78
- ◯ 79
- ◯ 80
- ◯ 81
- ◯ 82
- ◯ 83
- ◯ 84
- ◯ 85
- ◯ 86
- ◯ 87
- ◯ 88
- ◯ 89
- ◯ 90
- ◯ 91
- ◯ 92
- ◯ 93
- ◯ 94
- ◯ 95
- ◯ 96
- ◯ 97
- ◯ 98
- ◯ 99
- ◯ Don't know

At what age were you first told you had binge eating disorder?

- ◯ 0
- ◯ 1
- ◯ 2
- ◯ 3
- ◯ 4
- ◯ 5
- ◯ 6
- ◯ 7
- ◯ 8
- ◯ 9
- ◯ 10
- ◯ 11
- ◯ 12
- ◯ 13
- ◯ 14
- ◯ 15
- ◯ 16
- ◯ 17
- ◯ 18
- ◯ 19
- ◯ 20
- ◯ 21
- ◯ 22
- ◯ 23
- ◯ 24
- ◯ 25
- ◯ 26
- ◯ 27
- ◯ 28
- ◯ 29
- ◯ 30
- ◯ 31
- ◯ 32
- ◯ 33
- ◯ 34
- ◯ 35
- ◯ 36
- ◯ 37
- ◯ 38
- ◯ 39
- ◯ 40
- ◯ 41
- ◯ 42
- ◯ 43
- ◯ 44
- ◯ 45
- ◯ 46
- ◯ 47
- ◯ 48
- ◯ 49
- ◯ 50
- ◯ 51
- ◯ 52
- ◯ 53
- ◯ 54
- ◯ 55
- ◯ 56
- ◯ 57
- ◯ 58
- ◯ 59
- ◯ 60
- ◯ 61
- ◯ 62
- ◯ 63
- ◯ 64
- ◯ 65
- ◯ 66
- ◯ 67
- ◯ 68

- ◯ 69
- ◯ 70
- ◯ 71
- ◯ 72
- ◯ 73
- ◯ 74
- ◯ 75
- ◯ 76
- ◯ 77
- ◯ 78
- ◯ 79
- ◯ 80
- ◯ 81
- ◯ 82
- ◯ 83
- ◯ 84
- ◯ 85
- ◯ 86
- ◯ 87
- ◯ 88
- ◯ 89
- ◯ 90
- ◯ 91
- ◯ 92
- ◯ 93
- ◯ 94
- ◯ 95
- ◯ 96
- ◯ 97
- ◯ 98
- ◯ 99
- ◯ Don't know

At what age were you first told you had bulemia nervosa?

- ◯ 0
- ◯ 1
- ◯ 2
- ◯ 3
- ◯ 4
- ◯ 5
- ◯ 6
- ◯ 7
- ◯ 8
- ◯ 9
- ◯ 10
- ◯ 11
- ◯ 12
- ◯ 13
- ◯ 14
- ◯ 15
- ◯ 16
- ◯ 17
- ◯ 18
- ◯ 19
- ◯ 20
- ◯ 21
- ◯ 22
- ◯ 23
- ◯ 24
- ◯ 25
- ◯ 26
- ◯ 27
- ◯ 28
- ◯ 29
- ◯ 30
- ◯ 31
- ◯ 32
- ◯ 33
- ◯ 34
- ◯ 35
- ◯ 36
- ◯ 37
- ◯ 38
- ◯ 39
- ◯ 40
- ◯ 41
- ◯ 42
- ◯ 43
- ◯ 44
- ◯ 45
- ◯ 46
- ◯ 47
- ◯ 48
- ◯ 49
- ◯ 50
- ◯ 51
- ◯ 52
- ◯ 53
- ◯ 54
- ◯ 55
- ◯ 56
- ◯ 57
- ◯ 58
- ◯ 59
- ◯ 60
- ◯ 61
- ◯ 62
- ◯ 63
- ◯ 64
- ◯ 65
- ◯ 66
- ◯ 67
- ◯ 68

- ◯ 69
- ◯ 70
- ◯ 71
- ◯ 72
- ◯ 73
- ◯ 74
- ◯ 75
- ◯ 76
- ◯ 77
- ◯ 78
- ◯ 79
- ◯ 80
- ◯ 81
- ◯ 82
- ◯ 83
- ◯ 84
- ◯ 85
- ◯ 86
- ◯ 87
- ◯ 88
- ◯ 89
- ◯ 90
- ◯ 91
- ◯ 92
- ◯ 93
- ◯ 94
- ◯ 95
- ◯ 96
- ◯ 97
- ◯ 98
- ◯ 99
- ◯ Don't know

At what age were you first told you had [ed_others]?

- ◯ 0
- ◯ 1
- ◯ 2
- ◯ 3
- ◯ 4
- ◯ 5
- ◯ 6
- ◯ 7
- ◯ 8
- ◯ 9
- ◯ 10
- ◯ 11
- ◯ 12
- ◯ 13
- ◯ 14
- ◯ 15
- ◯ 16
- ◯ 17
- ◯ 18
- ◯ 19
- ◯ 20
- ◯ 21
- ◯ 22
- ◯ 23
- ◯ 24
- ◯ 25
- ◯ 26
- ◯ 27
- ◯ 28
- ◯ 29
- ◯ 30
- ◯ 31
- ◯ 32
- ◯ 33
- ◯ 34
- ◯ 35
- ◯ 36
- ◯ 37
- ◯ 38
- ◯ 39
- ◯ 40
- ◯ 41
- ◯ 42
- ◯ 43
- ◯ 44
- ◯ 45
- ◯ 46
- ◯ 47
- ◯ 48
- ◯ 49
- ◯ 50
- ◯ 51
- ◯ 52
- ◯ 53
- ◯ 54
- ◯ 55
- ◯ 56
- ◯ 57
- ◯ 58
- ◯ 59
- ◯ 60
- ◯ 61
- ◯ 62
- ◯ 63
- ◯ 64
- ◯ 65
- ◯ 66
- ◯ 67
- ◯ 68

◯ 69
◯ 70
◯ 71
◯ 72
◯ 73
◯ 74
◯ 75
◯ 76
◯ 77
◯ 78
◯ 79
◯ 80
◯ 81
◯ 82
◯ 83
◯ 84
◯ 85
◯ 86
◯ 87
◯ 88
◯ 89
◯ 90
◯ 91
◯ 92
◯ 93
◯ 94
◯ 95
◯ 96
◯ 97
◯ 98
◯ 99
◯ Don't know

At what age were you first told you had [ed_others_2]?

◯ 0
◯ 1
◯ 2
◯ 3
◯ 4
◯ 5
◯ 6
◯ 7
◯ 8
◯ 9
◯ 10
◯ 11
◯ 12
◯ 13
◯ 14
◯ 15
◯ 16
◯ 17
◯ 18
◯ 19
◯ 20
◯ 21
◯ 22
◯ 23
◯ 24
◯ 25
◯ 26
◯ 27
◯ 28
◯ 29
◯ 30
◯ 31
◯ 32
◯ 33
◯ 34
◯ 35
◯ 36
◯ 37
◯ 38
◯ 39
◯ 40
◯ 41
◯ 42
◯ 43
◯ 44
◯ 45
◯ 46
◯ 47
◯ 48
◯ 49
◯ 50
◯ 51
◯ 52
◯ 53
◯ 54
◯ 55
◯ 56
◯ 57
◯ 58
◯ 59
◯ 60
◯ 61
◯ 62
◯ 63
◯ 64
◯ 65
◯ 66
◯ 67
◯ 68

- ◯ 69
- ◯ 70
- ◯ 71
- ◯ 72
- ◯ 73
- ◯ 74
- ◯ 75
- ◯ 76
- ◯ 77
- ◯ 78
- ◯ 79
- ◯ 80
- ◯ 81
- ◯ 82
- ◯ 83
- ◯ 84
- ◯ 85
- ◯ 86
- ◯ 87
- ◯ 88
- ◯ 89
- ◯ 90
- ◯ 91
- ◯ 92
- ◯ 93
- ◯ 94
- ◯ 95
- ◯ 96
- ◯ 97
- ◯ 98
- ◯ 99
- ◯ Don't know

At what age were you first told you had [ed_others_3]?

- ◯ 0
- ◯ 1
- ◯ 2
- ◯ 3
- ◯ 4
- ◯ 5
- ◯ 6
- ◯ 7
- ◯ 8
- ◯ 9
- ◯ 10
- ◯ 11
- ◯ 12
- ◯ 13
- ◯ 14
- ◯ 15
- ◯ 16
- ◯ 17
- ◯ 18
- ◯ 19
- ◯ 20
- ◯ 21
- ◯ 22
- ◯ 23
- ◯ 24
- ◯ 25
- ◯ 26
- ◯ 27
- ◯ 28
- ◯ 29
- ◯ 30
- ◯ 31
- ◯ 32
- ◯ 33
- ◯ 34
- ◯ 35
- ◯ 36
- ◯ 37
- ◯ 38
- ◯ 39
- ◯ 40
- ◯ 41
- ◯ 42
- ◯ 43
- ◯ 44
- ◯ 45
- ◯ 46
- ◯ 47
- ◯ 48
- ◯ 49
- ◯ 50
- ◯ 51
- ◯ 52
- ◯ 53
- ◯ 54
- ◯ 55
- ◯ 56
- ◯ 57
- ◯ 58
- ◯ 59
- ◯ 60
- ◯ 61
- ◯ 62
- ◯ 63
- ◯ 64
- ◯ 65
- ◯ 66
- ◯ 67
- ◯ 68

◯ 69
◯ 70
◯ 71
◯ 72
◯ 73
◯ 74
◯ 75
◯ 76
◯ 77
◯ 78
◯ 79
◯ 80
◯ 81
◯ 82
◯ 83
◯ 84
◯ 85
◯ 86
◯ 87
◯ 88
◯ 89
◯ 90
◯ 91
◯ 92
◯ 93
◯ 94
◯ 95
◯ 96
◯ 97
◯ 98
◯ 99
◯ Don't know

At what age were you first told you had the condition?

- ◯ 0
- ◯ 1
- ◯ 2
- ◯ 3
- ◯ 4
- ◯ 5
- ◯ 6
- ◯ 7
- ◯ 8
- ◯ 9
- ◯ 10
- ◯ 11
- ◯ 12
- ◯ 13
- ◯ 14
- ◯ 15
- ◯ 16
- ◯ 17
- ◯ 18
- ◯ 19
- ◯ 20
- ◯ 21
- ◯ 22
- ◯ 23
- ◯ 24
- ◯ 25
- ◯ 26
- ◯ 27
- ◯ 28
- ◯ 29
- ◯ 30
- ◯ 31
- ◯ 32
- ◯ 33
- ◯ 34
- ◯ 35
- ◯ 36
- ◯ 37
- ◯ 38
- ◯ 39
- ◯ 40
- ◯ 41
- ◯ 42
- ◯ 43
- ◯ 44
- ◯ 45
- ◯ 46
- ◯ 47
- ◯ 48
- ◯ 49
- ◯ 50
- ◯ 51
- ◯ 52
- ◯ 53
- ◯ 54
- ◯ 55
- ◯ 56
- ◯ 57
- ◯ 58
- ◯ 59
- ◯ 60
- ◯ 61
- ◯ 62
- ◯ 63
- ◯ 64
- ◯ 65
- ◯ 66
- ◯ 67
- ◯ 68

	◯ 69<br>◯ 70<br>◯ 71<br>◯ 72<br>◯ 73<br>◯ 74<br>◯ 75<br>◯ 76<br>◯ 77<br>◯ 78<br>◯ 79<br>◯ 80<br>◯ 81<br>◯ 82<br>◯ 83<br>◯ 84<br>◯ 85<br>◯ 86<br>◯ 87<br>◯ 88<br>◯ 89<br>◯ 90<br>◯ 91<br>◯ 92<br>◯ 93<br>◯ 94<br>◯ 95<br>◯ 96<br>◯ 97<br>◯ 98<br>◯ 99<br>◯ Don't know
Do you still have anorexia nervosa?	◯ Yes<br>◯ No<br>◯ Don't know
Do you still have binge eating disorder?	◯ Yes<br>◯ No<br>◯ Don't know
Do you still have bulemia nervosa?	◯ Yes<br>◯ No<br>◯ Don't know
Do you still have [ed_others]?	◯ Yes<br>◯ No<br>◯ Don't know
Do you still have [ed_others_2]?	◯ Yes<br>◯ No<br>◯ Don't know
Do you still have [ed_others_3]?	◯ Yes<br>◯ No<br>◯ Don't know
Do you still have the condition?	◯ Yes<br>◯ No<br>◯ Don't know

How is anorexia nervosa treated?
(Tick all that apply)

☐ no treatment
☐ medication (please specify)
☐ natural/herbal therapy (please specify)
☐ supplements (please specify)
☐ specialist consultations (please specify)
☐ other (please specify)
☐ Don't Know

please specify the medication ______________________

please specify the natural/herbal therapy ______________________

please specify the supplements ______________________

please specify the specialist consultations ______________________

please specify the treatment ______________________

How is binge eating disorder treated?
(Tick all that apply)

☐ no treatment
☐ medication (please specify)
☐ natural/herbal therapy (please specify)
☐ supplements (please specify)
☐ specialist consultations (please specify)
☐ other (please specify)
☐ Don't Know

please specify the medication ______________________

please specify the natural/herbal therapy ______________________

please specify the supplements ______________________

please specify the specialist consultations ______________________

please specify the treatment ______________________

How is bulemia nervosa treated?
(Tick all that apply)

- ☐ no treatment
- ☐ medication (please specify)
- ☐ natural/herbal therapy (please specify)
- ☐ supplements (please specify)
- ☐ specialist consultations (please specify)
- ☐ other (please specify)
- ☐ Don't Know

please specify the medication ______________________

please specify the natural/herbal therapy ______________________

please specify the supplements ______________________

please specify the specialist consultations ______________________

please specify the treatment ______________________

How is [ed_others] treated?
(Tick all that apply)

- ☐ no treatment
- ☐ medication (please specify)
- ☐ natural/herbal therapy (please specify)
- ☐ supplements (please specify)
- ☐ specialist consultations (please specify)
- ☐ other (please specify)
- ☐ Don't Know

please specify the medication ______________________

please specify the natural/herbal therapy ______________________

please specify the supplements ______________________

please specify the specialist consultations ______________________

please specify the treatment ______________________

How is [ed_others_2] treated?
(Tick all that apply)

☐ no treatment
☐ medication (please specify)
☐ natural/herbal therapy (please specify)
☐ supplements (please specify)
☐ specialist consultations (please specify)
☐ other (please specify)
☐ Don't Know

please specify the medication

____

please specify the natural/herbal therapy

____

please specify the supplements

____

please specify the specialist consultations

____

please specify the treatment

____

How is [ed_others_3] treated?
(Tick all that apply)

☐ no treatment
☐ medication (please specify)
☐ natural/herbal therapy (please specify)
☐ supplements (please specify)
☐ specialist consultations (please specify)
☐ other (please specify)
☐ Don't Know

please specify the medication

____

please specify the natural/herbal therapy

____

please specify the supplements

____

please specify the specialist consultations

____

please specify the treatment

____

How is this condition treated?
(Tick all that apply)

- ☐ no treatment
- ☐ medication (please specify)
- ☐ natural/herbal therapy (please specify)
- ☐ supplements (please specify)
- ☐ specialist consultations (please specify)
- ☐ other (please specify)
- ☐ Don't Know

please specify the medication __________________________________

please specify the natural/herbal therapy __________________________________

please specify the supplements __________________________________

please specify the specialist consultations __________________________________

please specify the treatment __________________________________

Have you ever been told by a doctor (GP or specialist) or nurse that you have asthma?

- ◯ Yes
- ◯ No
- ◯ Don't know

At what age were you first told you had asthma?

○ 0
○ 1
○ 2
○ 3
○ 4
○ 5
○ 6
○ 7
○ 8
○ 9
○ 10
○ 11
○ 12
○ 13
○ 14
○ 15
○ 16
○ 17
○ 18
○ 19
○ 20
○ 21
○ 22
○ 23
○ 24
○ 25
○ 26
○ 27
○ 28
○ 29
○ 30
○ 31
○ 32
○ 33
○ 34
○ 35
○ 36
○ 37
○ 38
○ 39
○ 40
○ 41
○ 42
○ 43
○ 44
○ 45
○ 46
○ 47
○ 48
○ 49
○ 50
○ 51
○ 52
○ 53
○ 54
○ 55
○ 56
○ 57
○ 58
○ 59
○ 60
○ 61
○ 62
○ 63
○ 64
○ 65
○ 66
○ 67
○ 68

◯ 69
◯ 70
◯ 71
◯ 72
◯ 73
◯ 74
◯ 75
◯ 76
◯ 77
◯ 78
◯ 79
◯ 80
◯ 81
◯ 82
◯ 83
◯ 84
◯ 85
◯ 86
◯ 87
◯ 88
◯ 89
◯ 90
◯ 91
◯ 92
◯ 93
◯ 94
◯ 95
◯ 96
◯ 97
◯ 98
◯ 99
◯ Don't know

Do you still get asthma?

◯ Yes
◯ No
◯ Don't know

How is your asthma treated?
(Tick all that apply)

☐ no treatment ☐ diet ☐ exercise ☐ medication (please specify) ☐ natural/herbal therapy (please specify) ☐ weight management ☐ supplements (please specify) ☐ other (please specify) ☐ Don't know

---

please specify the medication

____

---

please specify the natural/herbal therapy

____

---

please specify the supplements

____

---

please specify the treatment

____

Have either of your parents, or any of brothers or sisters ever been told by a doctor (GP or specialist), or nurse that they have asthma?

◯ Yes
◯ No
◯ Don't know

Do you currently have a food allergy or intolerance?

◯ Yes
◯ No
◯ Don't know

Which food allergy/allergies or intolerance/s have been confirmed by blood test, skin prick test, biopsy, or elimination diet prescribed by a dietitian?
(Tick all that apply)

☐ None have been confirmed
☐ amine intolerance
☐ cow's milk/dairy allergy
☐ cow's milk/dairy/lactose intolerance
☐ coeliac disease (confirmed by biopsy)
☐ colour/flavour/preservative (e.g. sulphite) intolerance
☐ egg allergy
☐ fish allergy
☐ FODMAPS intolerance (e.g. fructans, fructose)
☐ glutamate intolerance
☐ gluten/wheat sensitivity (non-coeliac disease)
☐ lupin allergy
☐ peanut allergy
☐ salicylate intolerance
☐ shellfish allergy
☐ soy allergy
☐ tree nut allergy (e.g. cashew, hazelnut, walnut)
☐ wheat allergy
☐ yeast allergy
☐ Other (please specify)
☐ Don't Know

---

please specify the allergy or intolerance

____

Do you have another food allergy or intolerance need to be specified?

◯ Yes ◯ No

---

please specify the second allergy or intolerance

____

Do you have another food allergy or intolerance need to be specified?

◯ Yes ◯ No

---

please specify the third allergy or intolerance

____

At what age were you first told you had egg allergy?

- ◯ 0
- ◯ 1
- ◯ 2
- ◯ 3
- ◯ 4
- ◯ 5
- ◯ 6
- ◯ 7
- ◯ 8
- ◯ 9
- ◯ 10
- ◯ 11
- ◯ 12
- ◯ 13
- ◯ 14
- ◯ 15
- ◯ 16
- ◯ 17
- ◯ 18
- ◯ 19
- ◯ 20
- ◯ 21
- ◯ 22
- ◯ 23
- ◯ 24
- ◯ 25
- ◯ 26
- ◯ 27
- ◯ 28
- ◯ 29
- ◯ 30
- ◯ 31
- ◯ 32
- ◯ 33
- ◯ 34
- ◯ 35
- ◯ 36
- ◯ 37
- ◯ 38
- ◯ 39
- ◯ 40
- ◯ 41
- ◯ 42
- ◯ 43
- ◯ 44
- ◯ 45
- ◯ 46
- ◯ 47
- ◯ 48
- ◯ 49
- ◯ 50
- ◯ 51
- ◯ 52
- ◯ 53
- ◯ 54
- ◯ 55
- ◯ 56
- ◯ 57
- ◯ 58
- ◯ 59
- ◯ 60
- ◯ 61
- ◯ 62
- ◯ 63
- ◯ 64
- ◯ 65
- ◯ 66
- ◯ 67
- ◯ 68

◯ 69
◯ 70
◯ 71
◯ 72
◯ 73
◯ 74
◯ 75
◯ 76
◯ 77
◯ 78
◯ 79
◯ 80
◯ 81
◯ 82
◯ 83
◯ 84
◯ 85
◯ 86
◯ 87
◯ 88
◯ 89
◯ 90
◯ 91
◯ 92
◯ 93
◯ 94
◯ 95
◯ 96
◯ 97
◯ 98
◯ 99
◯ Don't know

At what age were you first told you had cow's milk/dairy allergy?

〇 0
〇 1
〇 2
〇 3
〇 4
〇 5
〇 6
〇 7
〇 8
〇 9
〇 10
〇 11
〇 12
〇 13
〇 14
〇 15
〇 16
〇 17
〇 18
〇 19
〇 20
〇 21
〇 22
〇 23
〇 24
〇 25
〇 26
〇 27
〇 28
〇 29
〇 30
〇 31
〇 32
〇 33
〇 34
〇 35
〇 36
〇 37
〇 38
〇 39
〇 40
〇 41
〇 42
〇 43
〇 44
〇 45
〇 46
〇 47
〇 48
〇 49
〇 50
〇 51
〇 52
〇 53
〇 54
〇 55
〇 56
〇 57
〇 58
〇 59
〇 60
〇 61
〇 62
〇 63
〇 64
〇 65
〇 66
〇 67
〇 68

◯ 69
◯ 70
◯ 71
◯ 72
◯ 73
◯ 74
◯ 75
◯ 76
◯ 77
◯ 78
◯ 79
◯ 80
◯ 81
◯ 82
◯ 83
◯ 84
◯ 85
◯ 86
◯ 87
◯ 88
◯ 89
◯ 90
◯ 91
◯ 92
◯ 93
◯ 94
◯ 95
◯ 96
◯ 97
◯ 98
◯ 99
◯ Don't know

At what age were you first told you had cow's milk/dairy/lactose intolerance?

- ◯ 0
- ◯ 1
- ◯ 2
- ◯ 3
- ◯ 4
- ◯ 5
- ◯ 6
- ◯ 7
- ◯ 8
- ◯ 9
- ◯ 10
- ◯ 11
- ◯ 12
- ◯ 13
- ◯ 14
- ◯ 15
- ◯ 16
- ◯ 17
- ◯ 18
- ◯ 19
- ◯ 20
- ◯ 21
- ◯ 22
- ◯ 23
- ◯ 24
- ◯ 25
- ◯ 26
- ◯ 27
- ◯ 28
- ◯ 29
- ◯ 30
- ◯ 31
- ◯ 32
- ◯ 33
- ◯ 34
- ◯ 35
- ◯ 36
- ◯ 37
- ◯ 38
- ◯ 39
- ◯ 40
- ◯ 41
- ◯ 42
- ◯ 43
- ◯ 44
- ◯ 45
- ◯ 46
- ◯ 47
- ◯ 48
- ◯ 49
- ◯ 50
- ◯ 51
- ◯ 52
- ◯ 53
- ◯ 54
- ◯ 55
- ◯ 56
- ◯ 57
- ◯ 58
- ◯ 59
- ◯ 60
- ◯ 61
- ◯ 62
- ◯ 63
- ◯ 64
- ◯ 65
- ◯ 66
- ◯ 67
- ◯ 68

- ◯ 69
- ◯ 70
- ◯ 71
- ◯ 72
- ◯ 73
- ◯ 74
- ◯ 75
- ◯ 76
- ◯ 77
- ◯ 78
- ◯ 79
- ◯ 80
- ◯ 81
- ◯ 82
- ◯ 83
- ◯ 84
- ◯ 85
- ◯ 86
- ◯ 87
- ◯ 88
- ◯ 89
- ◯ 90
- ◯ 91
- ◯ 92
- ◯ 93
- ◯ 94
- ◯ 95
- ◯ 96
- ◯ 97
- ◯ 98
- ◯ 99
- ◯ Don't know

At what age were you first told you had peanut allergy?

◯ 0
◯ 1
◯ 2
◯ 3
◯ 4
◯ 5
◯ 6
◯ 7
◯ 8
◯ 9
◯ 10
◯ 11
◯ 12
◯ 13
◯ 14
◯ 15
◯ 16
◯ 17
◯ 18
◯ 19
◯ 20
◯ 21
◯ 22
◯ 23
◯ 24
◯ 25
◯ 26
◯ 27
◯ 28
◯ 29
◯ 30
◯ 31
◯ 32
◯ 33
◯ 34
◯ 35
◯ 36
◯ 37
◯ 38
◯ 39
◯ 40
◯ 41
◯ 42
◯ 43
◯ 44
◯ 45
◯ 46
◯ 47
◯ 48
◯ 49
◯ 50
◯ 51
◯ 52
◯ 53
◯ 54
◯ 55
◯ 56
◯ 57
◯ 58
◯ 59
◯ 60
◯ 61
◯ 62
◯ 63
◯ 64
◯ 65
◯ 66
◯ 67
◯ 68

- ◯ 69
- ◯ 70
- ◯ 71
- ◯ 72
- ◯ 73
- ◯ 74
- ◯ 75
- ◯ 76
- ◯ 77
- ◯ 78
- ◯ 79
- ◯ 80
- ◯ 81
- ◯ 82
- ◯ 83
- ◯ 84
- ◯ 85
- ◯ 86
- ◯ 87
- ◯ 88
- ◯ 89
- ◯ 90
- ◯ 91
- ◯ 92
- ◯ 93
- ◯ 94
- ◯ 95
- ◯ 96
- ◯ 97
- ◯ 98
- ◯ 99
- ◯ Don't know

At what age were you first told you had tree nut allergy (e.g. cashew, hazelnut, walnut)?

- ◯ 0
- ◯ 1
- ◯ 2
- ◯ 3
- ◯ 4
- ◯ 5
- ◯ 6
- ◯ 7
- ◯ 8
- ◯ 9
- ◯ 10
- ◯ 11
- ◯ 12
- ◯ 13
- ◯ 14
- ◯ 15
- ◯ 16
- ◯ 17
- ◯ 18
- ◯ 19
- ◯ 20
- ◯ 21
- ◯ 22
- ◯ 23
- ◯ 24
- ◯ 25
- ◯ 26
- ◯ 27
- ◯ 28
- ◯ 29
- ◯ 30
- ◯ 31
- ◯ 32
- ◯ 33
- ◯ 34
- ◯ 35
- ◯ 36
- ◯ 37
- ◯ 38
- ◯ 39
- ◯ 40
- ◯ 41
- ◯ 42
- ◯ 43
- ◯ 44
- ◯ 45
- ◯ 46
- ◯ 47
- ◯ 48
- ◯ 49
- ◯ 50
- ◯ 51
- ◯ 52
- ◯ 53
- ◯ 54
- ◯ 55
- ◯ 56
- ◯ 57
- ◯ 58
- ◯ 59
- ◯ 60
- ◯ 61
- ◯ 62
- ◯ 63
- ◯ 64
- ◯ 65
- ◯ 66
- ◯ 67
- ◯ 68

◯ 69
◯ 70
◯ 71
◯ 72
◯ 73
◯ 74
◯ 75
◯ 76
◯ 77
◯ 78
◯ 79
◯ 80
◯ 81
◯ 82
◯ 83
◯ 84
◯ 85
◯ 86
◯ 87
◯ 88
◯ 89
◯ 90
◯ 91
◯ 92
◯ 93
◯ 94
◯ 95
◯ 96
◯ 97
◯ 98
◯ 99
◯ Don't know

At what age were you first told you had fish allergy?

◯ 0
◯ 1
◯ 2
◯ 3
◯ 4
◯ 5
◯ 6
◯ 7
◯ 8
◯ 9
◯ 10
◯ 11
◯ 12
◯ 13
◯ 14
◯ 15
◯ 16
◯ 17
◯ 18
◯ 19
◯ 20
◯ 21
◯ 22
◯ 23
◯ 24
◯ 25
◯ 26
◯ 27
◯ 28
◯ 29
◯ 30
◯ 31
◯ 32
◯ 33
◯ 34
◯ 35
◯ 36
◯ 37
◯ 38
◯ 39
◯ 40
◯ 41
◯ 42
◯ 43
◯ 44
◯ 45
◯ 46
◯ 47
◯ 48
◯ 49
◯ 50
◯ 51
◯ 52
◯ 53
◯ 54
◯ 55
◯ 56
◯ 57
◯ 58
◯ 59
◯ 60
◯ 61
◯ 62
◯ 63
◯ 64
◯ 65
◯ 66
◯ 67
◯ 68

- ◯ 69
- ◯ 70
- ◯ 71
- ◯ 72
- ◯ 73
- ◯ 74
- ◯ 75
- ◯ 76
- ◯ 77
- ◯ 78
- ◯ 79
- ◯ 80
- ◯ 81
- ◯ 82
- ◯ 83
- ◯ 84
- ◯ 85
- ◯ 86
- ◯ 87
- ◯ 88
- ◯ 89
- ◯ 90
- ◯ 91
- ◯ 92
- ◯ 93
- ◯ 94
- ◯ 95
- ◯ 96
- ◯ 97
- ◯ 98
- ◯ 99
- ◯ Don't know

At what age were you first told you had shellfish allergy?

◯ 0
◯ 1
◯ 2
◯ 3
◯ 4
◯ 5
◯ 6
◯ 7
◯ 8
◯ 9
◯ 10
◯ 11
◯ 12
◯ 13
◯ 14
◯ 15
◯ 16
◯ 17
◯ 18
◯ 19
◯ 20
◯ 21
◯ 22
◯ 23
◯ 24
◯ 25
◯ 26
◯ 27
◯ 28
◯ 29
◯ 30
◯ 31
◯ 32
◯ 33
◯ 34
◯ 35
◯ 36
◯ 37
◯ 38
◯ 39
◯ 40
◯ 41
◯ 42
◯ 43
◯ 44
◯ 45
◯ 46
◯ 47
◯ 48
◯ 49
◯ 50
◯ 51
◯ 52
◯ 53
◯ 54
◯ 55
◯ 56
◯ 57
◯ 58
◯ 59
◯ 60
◯ 61
◯ 62
◯ 63
◯ 64
◯ 65
◯ 66
◯ 67
◯ 68

- ◯ 69
- ◯ 70
- ◯ 71
- ◯ 72
- ◯ 73
- ◯ 74
- ◯ 75
- ◯ 76
- ◯ 77
- ◯ 78
- ◯ 79
- ◯ 80
- ◯ 81
- ◯ 82
- ◯ 83
- ◯ 84
- ◯ 85
- ◯ 86
- ◯ 87
- ◯ 88
- ◯ 89
- ◯ 90
- ◯ 91
- ◯ 92
- ◯ 93
- ◯ 94
- ◯ 95
- ◯ 96
- ◯ 97
- ◯ 98
- ◯ 99
- ◯ Don't know

At what age were you first told you had soy allergy?

- 0
- 1
- 2
- 3
- 4
- 5
- 6
- 7
- 8
- 9
- 10
- 11
- 12
- 13
- 14
- 15
- 16
- 17
- 18
- 19
- 20
- 21
- 22
- 23
- 24
- 25
- 26
- 27
- 28
- 29
- 30
- 31
- 32
- 33
- 34
- 35
- 36
- 37
- 38
- 39
- 40
- 41
- 42
- 43
- 44
- 45
- 46
- 47
- 48
- 49
- 50
- 51
- 52
- 53
- 54
- 55
- 56
- 57
- 58
- 59
- 60
- 61
- 62
- 63
- 64
- 65
- 66
- 67
- 68

- ◯ 69
- ◯ 70
- ◯ 71
- ◯ 72
- ◯ 73
- ◯ 74
- ◯ 75
- ◯ 76
- ◯ 77
- ◯ 78
- ◯ 79
- ◯ 80
- ◯ 81
- ◯ 82
- ◯ 83
- ◯ 84
- ◯ 85
- ◯ 86
- ◯ 87
- ◯ 88
- ◯ 89
- ◯ 90
- ◯ 91
- ◯ 92
- ◯ 93
- ◯ 94
- ◯ 95
- ◯ 96
- ◯ 97
- ◯ 98
- ◯ 99
- ◯ Don't know

At what age were you first told you had lupin allergy?

- ◯ 0
- ◯ 1
- ◯ 2
- ◯ 3
- ◯ 4
- ◯ 5
- ◯ 6
- ◯ 7
- ◯ 8
- ◯ 9
- ◯ 10
- ◯ 11
- ◯ 12
- ◯ 13
- ◯ 14
- ◯ 15
- ◯ 16
- ◯ 17
- ◯ 18
- ◯ 19
- ◯ 20
- ◯ 21
- ◯ 22
- ◯ 23
- ◯ 24
- ◯ 25
- ◯ 26
- ◯ 27
- ◯ 28
- ◯ 29
- ◯ 30
- ◯ 31
- ◯ 32
- ◯ 33
- ◯ 34
- ◯ 35
- ◯ 36
- ◯ 37
- ◯ 38
- ◯ 39
- ◯ 40
- ◯ 41
- ◯ 42
- ◯ 43
- ◯ 44
- ◯ 45
- ◯ 46
- ◯ 47
- ◯ 48
- ◯ 49
- ◯ 50
- ◯ 51
- ◯ 52
- ◯ 53
- ◯ 54
- ◯ 55
- ◯ 56
- ◯ 57
- ◯ 58
- ◯ 59
- ◯ 60
- ◯ 61
- ◯ 62
- ◯ 63
- ◯ 64
- ◯ 65
- ◯ 66
- ◯ 67
- ◯ 68

- ◯ 69
- ◯ 70
- ◯ 71
- ◯ 72
- ◯ 73
- ◯ 74
- ◯ 75
- ◯ 76
- ◯ 77
- ◯ 78
- ◯ 79
- ◯ 80
- ◯ 81
- ◯ 82
- ◯ 83
- ◯ 84
- ◯ 85
- ◯ 86
- ◯ 87
- ◯ 88
- ◯ 89
- ◯ 90
- ◯ 91
- ◯ 92
- ◯ 93
- ◯ 94
- ◯ 95
- ◯ 96
- ◯ 97
- ◯ 98
- ◯ 99
- ◯ Don't know

At what age were you first told you had coeliac disease (confirmed by biopsy)?

◯ 0
◯ 1
◯ 2
◯ 3
◯ 4
◯ 5
◯ 6
◯ 7
◯ 8
◯ 9
◯ 10
◯ 11
◯ 12
◯ 13
◯ 14
◯ 15
◯ 16
◯ 17
◯ 18
◯ 19
◯ 20
◯ 21
◯ 22
◯ 23
◯ 24
◯ 25
◯ 26
◯ 27
◯ 28
◯ 29
◯ 30
◯ 31
◯ 32
◯ 33
◯ 34
◯ 35
◯ 36
◯ 37
◯ 38
◯ 39
◯ 40
◯ 41
◯ 42
◯ 43
◯ 44
◯ 45
◯ 46
◯ 47
◯ 48
◯ 49
◯ 50
◯ 51
◯ 52
◯ 53
◯ 54
◯ 55
◯ 56
◯ 57
◯ 58
◯ 59
◯ 60
◯ 61
◯ 62
◯ 63
◯ 64
◯ 65
◯ 66
◯ 67
◯ 68

- ◯ 69
- ◯ 70
- ◯ 71
- ◯ 72
- ◯ 73
- ◯ 74
- ◯ 75
- ◯ 76
- ◯ 77
- ◯ 78
- ◯ 79
- ◯ 80
- ◯ 81
- ◯ 82
- ◯ 83
- ◯ 84
- ◯ 85
- ◯ 86
- ◯ 87
- ◯ 88
- ◯ 89
- ◯ 90
- ◯ 91
- ◯ 92
- ◯ 93
- ◯ 94
- ◯ 95
- ◯ 96
- ◯ 97
- ◯ 98
- ◯ 99
- ◯ Don't know

At what age were you first told you had gluten/wheat sensitivity (non-coeliac disease)?

- 0
- 1
- 2
- 3
- 4
- 5
- 6
- 7
- 8
- 9
- 10
- 11
- 12
- 13
- 14
- 15
- 16
- 17
- 18
- 19
- 20
- 21
- 22
- 23
- 24
- 25
- 26
- 27
- 28
- 29
- 30
- 31
- 32
- 33
- 34
- 35
- 36
- 37
- 38
- 39
- 40
- 41
- 42
- 43
- 44
- 45
- 46
- 47
- 48
- 49
- 50
- 51
- 52
- 53
- 54
- 55
- 56
- 57
- 58
- 59
- 60
- 61
- 62
- 63
- 64
- 65
- 66
- 67
- 68

- ◯ 69
- ◯ 70
- ◯ 71
- ◯ 72
- ◯ 73
- ◯ 74
- ◯ 75
- ◯ 76
- ◯ 77
- ◯ 78
- ◯ 79
- ◯ 80
- ◯ 81
- ◯ 82
- ◯ 83
- ◯ 84
- ◯ 85
- ◯ 86
- ◯ 87
- ◯ 88
- ◯ 89
- ◯ 90
- ◯ 91
- ◯ 92
- ◯ 93
- ◯ 94
- ◯ 95
- ◯ 96
- ◯ 97
- ◯ 98
- ◯ 99
- ◯ Don't know

At what age were you first told you had wheat allergy?

◯ 0
◯ 1
◯ 2
◯ 3
◯ 4
◯ 5
◯ 6
◯ 7
◯ 8
◯ 9
◯ 10
◯ 11
◯ 12
◯ 13
◯ 14
◯ 15
◯ 16
◯ 17
◯ 18
◯ 19
◯ 20
◯ 21
◯ 22
◯ 23
◯ 24
◯ 25
◯ 26
◯ 27
◯ 28
◯ 29
◯ 30
◯ 31
◯ 32
◯ 33
◯ 34
◯ 35
◯ 36
◯ 37
◯ 38
◯ 39
◯ 40
◯ 41
◯ 42
◯ 43
◯ 44
◯ 45
◯ 46
◯ 47
◯ 48
◯ 49
◯ 50
◯ 51
◯ 52
◯ 53
◯ 54
◯ 55
◯ 56
◯ 57
◯ 58
◯ 59
◯ 60
◯ 61
◯ 62
◯ 63
◯ 64
◯ 65
◯ 66
◯ 67
◯ 68

- ◯ 69
- ◯ 70
- ◯ 71
- ◯ 72
- ◯ 73
- ◯ 74
- ◯ 75
- ◯ 76
- ◯ 77
- ◯ 78
- ◯ 79
- ◯ 80
- ◯ 81
- ◯ 82
- ◯ 83
- ◯ 84
- ◯ 85
- ◯ 86
- ◯ 87
- ◯ 88
- ◯ 89
- ◯ 90
- ◯ 91
- ◯ 92
- ◯ 93
- ◯ 94
- ◯ 95
- ◯ 96
- ◯ 97
- ◯ 98
- ◯ 99
- ◯ Don't know

At what age were you first told you had yeast allergy?

◯ 0
◯ 1
◯ 2
◯ 3
◯ 4
◯ 5
◯ 6
◯ 7
◯ 8
◯ 9
◯ 10
◯ 11
◯ 12
◯ 13
◯ 14
◯ 15
◯ 16
◯ 17
◯ 18
◯ 19
◯ 20
◯ 21
◯ 22
◯ 23
◯ 24
◯ 25
◯ 26
◯ 27
◯ 28
◯ 29
◯ 30
◯ 31
◯ 32
◯ 33
◯ 34
◯ 35
◯ 36
◯ 37
◯ 38
◯ 39
◯ 40
◯ 41
◯ 42
◯ 43
◯ 44
◯ 45
◯ 46
◯ 47
◯ 48
◯ 49
◯ 50
◯ 51
◯ 52
◯ 53
◯ 54
◯ 55
◯ 56
◯ 57
◯ 58
◯ 59
◯ 60
◯ 61
◯ 62
◯ 63
◯ 64
◯ 65
◯ 66
◯ 67
◯ 68

◯ 69
◯ 70
◯ 71
◯ 72
◯ 73
◯ 74
◯ 75
◯ 76
◯ 77
◯ 78
◯ 79
◯ 80
◯ 81
◯ 82
◯ 83
◯ 84
◯ 85
◯ 86
◯ 87
◯ 88
◯ 89
◯ 90
◯ 91
◯ 92
◯ 93
◯ 94
◯ 95
◯ 96
◯ 97
◯ 98
◯ 99
◯ Don't know

At what age were you first told you had salicylate intolerance?

- 0
- 1
- 2
- 3
- 4
- 5
- 6
- 7
- 8
- 9
- 10
- 11
- 12
- 13
- 14
- 15
- 16
- 17
- 18
- 19
- 20
- 21
- 22
- 23
- 24
- 25
- 26
- 27
- 28
- 29
- 30
- 31
- 32
- 33
- 34
- 35
- 36
- 37
- 38
- 39
- 40
- 41
- 42
- 43
- 44
- 45
- 46
- 47
- 48
- 49
- 50
- 51
- 52
- 53
- 54
- 55
- 56
- 57
- 58
- 59
- 60
- 61
- 62
- 63
- 64
- 65
- 66
- 67
- 68

◯ 69
◯ 70
◯ 71
◯ 72
◯ 73
◯ 74
◯ 75
◯ 76
◯ 77
◯ 78
◯ 79
◯ 80
◯ 81
◯ 82
◯ 83
◯ 84
◯ 85
◯ 86
◯ 87
◯ 88
◯ 89
◯ 90
◯ 91
◯ 92
◯ 93
◯ 94
◯ 95
◯ 96
◯ 97
◯ 98
◯ 99
◯ Don't know

At what age were you first told you had amine intolerance?

- ◯ 0
- ◯ 1
- ◯ 2
- ◯ 3
- ◯ 4
- ◯ 5
- ◯ 6
- ◯ 7
- ◯ 8
- ◯ 9
- ◯ 10
- ◯ 11
- ◯ 12
- ◯ 13
- ◯ 14
- ◯ 15
- ◯ 16
- ◯ 17
- ◯ 18
- ◯ 19
- ◯ 20
- ◯ 21
- ◯ 22
- ◯ 23
- ◯ 24
- ◯ 25
- ◯ 26
- ◯ 27
- ◯ 28
- ◯ 29
- ◯ 30
- ◯ 31
- ◯ 32
- ◯ 33
- ◯ 34
- ◯ 35
- ◯ 36
- ◯ 37
- ◯ 38
- ◯ 39
- ◯ 40
- ◯ 41
- ◯ 42
- ◯ 43
- ◯ 44
- ◯ 45
- ◯ 46
- ◯ 47
- ◯ 48
- ◯ 49
- ◯ 50
- ◯ 51
- ◯ 52
- ◯ 53
- ◯ 54
- ◯ 55
- ◯ 56
- ◯ 57
- ◯ 58
- ◯ 59
- ◯ 60
- ◯ 61
- ◯ 62
- ◯ 63
- ◯ 64
- ◯ 65
- ◯ 66
- ◯ 67
- ◯ 68

◯ 69
◯ 70
◯ 71
◯ 72
◯ 73
◯ 74
◯ 75
◯ 76
◯ 77
◯ 78
◯ 79
◯ 80
◯ 81
◯ 82
◯ 83
◯ 84
◯ 85
◯ 86
◯ 87
◯ 88
◯ 89
◯ 90
◯ 91
◯ 92
◯ 93
◯ 94
◯ 95
◯ 96
◯ 97
◯ 98
◯ 99
◯ Don't know

At what age were you first told you had glutamate intolerance?

- ◯ 0
- ◯ 1
- ◯ 2
- ◯ 3
- ◯ 4
- ◯ 5
- ◯ 6
- ◯ 7
- ◯ 8
- ◯ 9
- ◯ 10
- ◯ 11
- ◯ 12
- ◯ 13
- ◯ 14
- ◯ 15
- ◯ 16
- ◯ 17
- ◯ 18
- ◯ 19
- ◯ 20
- ◯ 21
- ◯ 22
- ◯ 23
- ◯ 24
- ◯ 25
- ◯ 26
- ◯ 27
- ◯ 28
- ◯ 29
- ◯ 30
- ◯ 31
- ◯ 32
- ◯ 33
- ◯ 34
- ◯ 35
- ◯ 36
- ◯ 37
- ◯ 38
- ◯ 39
- ◯ 40
- ◯ 41
- ◯ 42
- ◯ 43
- ◯ 44
- ◯ 45
- ◯ 46
- ◯ 47
- ◯ 48
- ◯ 49
- ◯ 50
- ◯ 51
- ◯ 52
- ◯ 53
- ◯ 54
- ◯ 55
- ◯ 56
- ◯ 57
- ◯ 58
- ◯ 59
- ◯ 60
- ◯ 61
- ◯ 62
- ◯ 63
- ◯ 64
- ◯ 65
- ◯ 66
- ◯ 67
- ◯ 68

- ◯ 69
- ◯ 70
- ◯ 71
- ◯ 72
- ◯ 73
- ◯ 74
- ◯ 75
- ◯ 76
- ◯ 77
- ◯ 78
- ◯ 79
- ◯ 80
- ◯ 81
- ◯ 82
- ◯ 83
- ◯ 84
- ◯ 85
- ◯ 86
- ◯ 87
- ◯ 88
- ◯ 89
- ◯ 90
- ◯ 91
- ◯ 92
- ◯ 93
- ◯ 94
- ◯ 95
- ◯ 96
- ◯ 97
- ◯ 98
- ◯ 99
- ◯ Don't know

At what age were you first told you had colour/flavour/preservative (e.g. sulphite) intolerance?

- ◯ 0
- ◯ 1
- ◯ 2
- ◯ 3
- ◯ 4
- ◯ 5
- ◯ 6
- ◯ 7
- ◯ 8
- ◯ 9
- ◯ 10
- ◯ 11
- ◯ 12
- ◯ 13
- ◯ 14
- ◯ 15
- ◯ 16
- ◯ 17
- ◯ 18
- ◯ 19
- ◯ 20
- ◯ 21
- ◯ 22
- ◯ 23
- ◯ 24
- ◯ 25
- ◯ 26
- ◯ 27
- ◯ 28
- ◯ 29
- ◯ 30
- ◯ 31
- ◯ 32
- ◯ 33
- ◯ 34
- ◯ 35
- ◯ 36
- ◯ 37
- ◯ 38
- ◯ 39
- ◯ 40
- ◯ 41
- ◯ 42
- ◯ 43
- ◯ 44
- ◯ 45
- ◯ 46
- ◯ 47
- ◯ 48
- ◯ 49
- ◯ 50
- ◯ 51
- ◯ 52
- ◯ 53
- ◯ 54
- ◯ 55
- ◯ 56
- ◯ 57
- ◯ 58
- ◯ 59
- ◯ 60
- ◯ 61
- ◯ 62
- ◯ 63
- ◯ 64
- ◯ 65
- ◯ 66
- ◯ 67
- ◯ 68

- ◯ 69
- ◯ 70
- ◯ 71
- ◯ 72
- ◯ 73
- ◯ 74
- ◯ 75
- ◯ 76
- ◯ 77
- ◯ 78
- ◯ 79
- ◯ 80
- ◯ 81
- ◯ 82
- ◯ 83
- ◯ 84
- ◯ 85
- ◯ 86
- ◯ 87
- ◯ 88
- ◯ 89
- ◯ 90
- ◯ 91
- ◯ 92
- ◯ 93
- ◯ 94
- ◯ 95
- ◯ 96
- ◯ 97
- ◯ 98
- ◯ 99
- ◯ Don't know

At what age were you first told you had FODMAPS intolerance (e.g. fructans, fructose)?

◯ 0
◯ 1
◯ 2
◯ 3
◯ 4
◯ 5
◯ 6
◯ 7
◯ 8
◯ 9
◯ 10
◯ 11
◯ 12
◯ 13
◯ 14
◯ 15
◯ 16
◯ 17
◯ 18
◯ 19
◯ 20
◯ 21
◯ 22
◯ 23
◯ 24
◯ 25
◯ 26
◯ 27
◯ 28
◯ 29
◯ 30
◯ 31
◯ 32
◯ 33
◯ 34
◯ 35
◯ 36
◯ 37
◯ 38
◯ 39
◯ 40
◯ 41
◯ 42
◯ 43
◯ 44
◯ 45
◯ 46
◯ 47
◯ 48
◯ 49
◯ 50
◯ 51
◯ 52
◯ 53
◯ 54
◯ 55
◯ 56
◯ 57
◯ 58
◯ 59
◯ 60
◯ 61
◯ 62
◯ 63
◯ 64
◯ 65
◯ 66
◯ 67
◯ 68

◯ 69
◯ 70
◯ 71
◯ 72
◯ 73
◯ 74
◯ 75
◯ 76
◯ 77
◯ 78
◯ 79
◯ 80
◯ 81
◯ 82
◯ 83
◯ 84
◯ 85
◯ 86
◯ 87
◯ 88
◯ 89
◯ 90
◯ 91
◯ 92
◯ 93
◯ 94
◯ 95
◯ 96
◯ 97
◯ 98
◯ 99
◯ Don't know

At what age were you first told you had [allergy_others]?

- ◯ 0
- ◯ 1
- ◯ 2
- ◯ 3
- ◯ 4
- ◯ 5
- ◯ 6
- ◯ 7
- ◯ 8
- ◯ 9
- ◯ 10
- ◯ 11
- ◯ 12
- ◯ 13
- ◯ 14
- ◯ 15
- ◯ 16
- ◯ 17
- ◯ 18
- ◯ 19
- ◯ 20
- ◯ 21
- ◯ 22
- ◯ 23
- ◯ 24
- ◯ 25
- ◯ 26
- ◯ 27
- ◯ 28
- ◯ 29
- ◯ 30
- ◯ 31
- ◯ 32
- ◯ 33
- ◯ 34
- ◯ 35
- ◯ 36
- ◯ 37
- ◯ 38
- ◯ 39
- ◯ 40
- ◯ 41
- ◯ 42
- ◯ 43
- ◯ 44
- ◯ 45
- ◯ 46
- ◯ 47
- ◯ 48
- ◯ 49
- ◯ 50
- ◯ 51
- ◯ 52
- ◯ 53
- ◯ 54
- ◯ 55
- ◯ 56
- ◯ 57
- ◯ 58
- ◯ 59
- ◯ 60
- ◯ 61
- ◯ 62
- ◯ 63
- ◯ 64
- ◯ 65
- ◯ 66
- ◯ 67
- ◯ 68

◯ 69
◯ 70
◯ 71
◯ 72
◯ 73
◯ 74
◯ 75
◯ 76
◯ 77
◯ 78
◯ 79
◯ 80
◯ 81
◯ 82
◯ 83
◯ 84
◯ 85
◯ 86
◯ 87
◯ 88
◯ 89
◯ 90
◯ 91
◯ 92
◯ 93
◯ 94
◯ 95
◯ 96
◯ 97
◯ 98
◯ 99
◯ Don't know

At what age were you first told you had [allergy_others_2]?

◯ 0
◯ 1
◯ 2
◯ 3
◯ 4
◯ 5
◯ 6
◯ 7
◯ 8
◯ 9
◯ 10
◯ 11
◯ 12
◯ 13
◯ 14
◯ 15
◯ 16
◯ 17
◯ 18
◯ 19
◯ 20
◯ 21
◯ 22
◯ 23
◯ 24
◯ 25
◯ 26
◯ 27
◯ 28
◯ 29
◯ 30
◯ 31
◯ 32
◯ 33
◯ 34
◯ 35
◯ 36
◯ 37
◯ 38
◯ 39
◯ 40
◯ 41
◯ 42
◯ 43
◯ 44
◯ 45
◯ 46
◯ 47
◯ 48
◯ 49
◯ 50
◯ 51
◯ 52
◯ 53
◯ 54
◯ 55
◯ 56
◯ 57
◯ 58
◯ 59
◯ 60
◯ 61
◯ 62
◯ 63
◯ 64
◯ 65
◯ 66
◯ 67
◯ 68

- ◯ 69
- ◯ 70
- ◯ 71
- ◯ 72
- ◯ 73
- ◯ 74
- ◯ 75
- ◯ 76
- ◯ 77
- ◯ 78
- ◯ 79
- ◯ 80
- ◯ 81
- ◯ 82
- ◯ 83
- ◯ 84
- ◯ 85
- ◯ 86
- ◯ 87
- ◯ 88
- ◯ 89
- ◯ 90
- ◯ 91
- ◯ 92
- ◯ 93
- ◯ 94
- ◯ 95
- ◯ 96
- ◯ 97
- ◯ 98
- ◯ 99
- ◯ Don't know

At what age were you first told you had [allergy_others_3]?

- ◯ 0
- ◯ 1
- ◯ 2
- ◯ 3
- ◯ 4
- ◯ 5
- ◯ 6
- ◯ 7
- ◯ 8
- ◯ 9
- ◯ 10
- ◯ 11
- ◯ 12
- ◯ 13
- ◯ 14
- ◯ 15
- ◯ 16
- ◯ 17
- ◯ 18
- ◯ 19
- ◯ 20
- ◯ 21
- ◯ 22
- ◯ 23
- ◯ 24
- ◯ 25
- ◯ 26
- ◯ 27
- ◯ 28
- ◯ 29
- ◯ 30
- ◯ 31
- ◯ 32
- ◯ 33
- ◯ 34
- ◯ 35
- ◯ 36
- ◯ 37
- ◯ 38
- ◯ 39
- ◯ 40
- ◯ 41
- ◯ 42
- ◯ 43
- ◯ 44
- ◯ 45
- ◯ 46
- ◯ 47
- ◯ 48
- ◯ 49
- ◯ 50
- ◯ 51
- ◯ 52
- ◯ 53
- ◯ 54
- ◯ 55
- ◯ 56
- ◯ 57
- ◯ 58
- ◯ 59
- ◯ 60
- ◯ 61
- ◯ 62
- ◯ 63
- ◯ 64
- ◯ 65
- ◯ 66
- ◯ 67
- ◯ 68

◯ 69
◯ 70
◯ 71
◯ 72
◯ 73
◯ 74
◯ 75
◯ 76
◯ 77
◯ 78
◯ 79
◯ 80
◯ 81
◯ 82
◯ 83
◯ 84
◯ 85
◯ 86
◯ 87
◯ 88
◯ 89
◯ 90
◯ 91
◯ 92
◯ 93
◯ 94
◯ 95
◯ 96
◯ 97
◯ 98
◯ 99
◯ Don't know

At what age were you first told you had this condition?

- 0
- 1
- 2
- 3
- 4
- 5
- 6
- 7
- 8
- 9
- 10
- 11
- 12
- 13
- 14
- 15
- 16
- 17
- 18
- 19
- 20
- 21
- 22
- 23
- 24
- 25
- 26
- 27
- 28
- 29
- 30
- 31
- 32
- 33
- 34
- 35
- 36
- 37
- 38
- 39
- 40
- 41
- 42
- 43
- 44
- 45
- 46
- 47
- 48
- 49
- 50
- 51
- 52
- 53
- 54
- 55
- 56
- 57
- 58
- 59
- 60
- 61
- 62
- 63
- 64
- 65
- 66
- 67
- 68

	◯ 69<br>◯ 70<br>◯ 71<br>◯ 72<br>◯ 73<br>◯ 74<br>◯ 75<br>◯ 76<br>◯ 77<br>◯ 78<br>◯ 79<br>◯ 80<br>◯ 81<br>◯ 82<br>◯ 83<br>◯ 84<br>◯ 85<br>◯ 86<br>◯ 87<br>◯ 88<br>◯ 89<br>◯ 90<br>◯ 91<br>◯ 92<br>◯ 93<br>◯ 94<br>◯ 95<br>◯ 96<br>◯ 97<br>◯ 98<br>◯ 99<br>◯ Don't know
Do you still have egg allergy?	◯ Yes<br>◯ No<br>◯ Don't know
Do you still have milk/dairy allergy?	◯ Yes<br>◯ No<br>◯ Don't know
Do you still have milk/dairy/lactose intolerance?	◯ Yes<br>◯ No<br>◯ Don't know
Do you still have peanut allergy?	◯ Yes<br>◯ No<br>◯ Don't know
Do you still have nut allergy (e.g. cashew, hazelnut, walnut)?	◯ Yes<br>◯ No<br>◯ Don't know
Do you still have fish allergy?	◯ Yes<br>◯ No<br>◯ Don't know
Do you still have shellfish allergy?	◯ Yes<br>◯ No<br>◯ Don't know

Do you still have soy allergy?	◯ Yes<br>◯ No<br>◯ Don't know
Do you still have lupin allergy?	◯ Yes<br>◯ No<br>◯ Don't know
Do you still have coeliac disease?	◯ Yes<br>◯ No<br>◯ Don't know
Do you still have gluten/wheat sensitivity (non-coeliac disease)?	◯ Yes<br>◯ No<br>◯ Don't know
Do you still have wheat allergy?	◯ Yes<br>◯ No<br>◯ Don't know
Do you still have yeast allergy?	◯ Yes<br>◯ No<br>◯ Don't know
Do you still have salicylate intolerance?	◯ Yes<br>◯ No<br>◯ Don't know
Do you still have amine intolerance?	◯ Yes<br>◯ No<br>◯ Don't know
Do you still have glutamate intolerance?	◯ Yes<br>◯ No<br>◯ Don't know
Do you still have colour/flavour/preservative (e.g. sulphite) intolerance?	◯ Yes<br>◯ No<br>◯ Don't know
Do you still have FODMAPS intolerance (e.g. fructans, fructose)?	◯ Yes<br>◯ No<br>◯ Don't know
Do you still have [allergy_others]?	◯ Yes<br>◯ No<br>◯ Don't know
Do you still have [allergy_others_2]?	◯ Yes<br>◯ No<br>◯ Don't know
Do you still have [allergy_others_3]?	◯ Yes<br>◯ No<br>◯ Don't know

Do you still have this condition?
◯ Yes
◯ No
◯ Don't know

How is egg allergy treated?
(Tick all that apply)

☐ no treatment ☐ diet ☐ exercise ☐ medication (please specify) ☐ natural/herbal therapy (please specify) ☐ weight management ☐ supplements (please specify) ☐ other (please specify)
☐ Don't know

please specify the medication ______________________________

please specify the natural/herbal therapy ______________________________

please specify the supplements ______________________________

please specify the treatment ______________________________

How is cow's milk/dairy allergy treated?
(Tick all that apply)

☐ no treatment ☐ diet ☐ exercise ☐ medication (please specify) ☐ natural/herbal therapy (please specify) ☐ weight management ☐ supplements (please specify) ☐ other (please specify)
☐ Don't know

please specify the medication ______________________________

please specify the natural/herbal therapy ______________________________

please specify the supplements ______________________________

please specify the treatment ______________________________

How is cow's milk/dairy/lactose intolerance treated?
(Tick all that apply)

☐ no treatment ☐ diet ☐ exercise ☐ medication (please specify) ☐ natural/herbal therapy (please specify) ☐ weight management ☐ supplements (please specify) ☐ other (please specify)
☐ Don't know

please specify the medication ______________________________

please specify the natural/herbal therapy ______________________________

please specify the supplements ______________________________

please specify the treatment

____

How is peanut allergy treated?
(Tick all that apply)

☐ no treatment ☐ diet ☐ exercise ☐ medication (please specify) ☐ natural/herbal therapy (please specify) ☐ weight management ☐ supplements (please specify) ☐ other (please specify)
☐ Don't know

please specify the medication

____

please specify the natural/herbal therapy

____

please specify the supplements

____

please specify the treatment

____

How is tree nut allergy (e.g. cashew, hazelnut, walnut) treated?
(Tick all that apply)

☐ no treatment ☐ diet ☐ exercise ☐ medication (please specify) ☐ natural/herbal therapy (please specify) ☐ weight management ☐ supplements (please specify) ☐ other (please specify)
☐ Don't know

please specify the medication

____

please specify the natural/herbal therapy

____

please specify the supplements

____

please specify the treatment

____

How is fish allergy treated?
(Tick all that apply)

☐ no treatment ☐ diet ☐ exercise ☐ medication (please specify) ☐ natural/herbal therapy (please specify) ☐ weight management ☐ supplements (please specify) ☐ other (please specify)
☐ Don't know

please specify the medication

____

please specify the natural/herbal therapy

____

please specify the supplements

____

please specify the treatment

____

How is shellfish allergy treated?

☐ no treatment ☐ diet ☐ exercise ☐ medication (please specify) ☐ natural/herbal therapy (please specify) ☐ weight management ☐ supplements (please specify) ☐ other (please specify) ☐ Don't know

please specify the medication

____

please specify the natural/herbal therapy

____

please specify the supplements

____

please specify the treatment

____

How is soy allergy treated?
(Tick all that apply)

☐ no treatment ☐ diet ☐ exercise ☐ medication (please specify) ☐ natural/herbal therapy (please specify) ☐ weight management ☐ supplements (please specify) ☐ other (please specify) ☐ Don't know

please specify the medication

____

please specify the natural/herbal therapy

____

please specify the supplements

____

please specify the treatment

____

How is lupin allergy treated?
(Tick all that apply)

☐ no treatment ☐ diet ☐ exercise ☐ medication (please specify) ☐ natural/herbal therapy (please specify) ☐ weight management ☐ supplements (please specify) ☐ other (please specify) ☐ Don't know

please specify the medication

____

please specify the natural/herbal therapy

____

please specify the supplements

____

please specify the treatment

____

How is coeliac disease (confirmed by biopsy) treated?
(Tick all that apply)

☐ no treatment ☐ diet ☐ exercise ☐ medication (please specify) ☐ natural/herbal therapy (please specify) ☐ weight management ☐ supplements (please specify) ☐ other (please specify)
☐ Don't know

please specify the medication

____

please specify the natural/herbal therapy

____

please specify the supplements

____

please specify the treatment

____

How is gluten/wheat sensitivity (non-coeliac disease) treated?
(Tick all that apply)

☐ no treatment ☐ diet ☐ exercise ☐ medication (please specify) ☐ natural/herbal therapy (please specify) ☐ weight management ☐ supplements (please specify) ☐ other (please specify)
☐ Don't know

please specify the medication

____

please specify the natural/herbal therapy

____

please specify the supplements

____

please specify the treatment

____

How is wheat allergy treated?
(Tick all that apply)

☐ no treatment ☐ diet ☐ exercise ☐ medication (please specify) ☐ natural/herbal therapy (please specify) ☐ weight management ☐ supplements (please specify) ☐ other (please specify)
☐ Don't know

please specify the medication

____

please specify the natural/herbal therapy

____

please specify the supplements

____

please specify the treatment

____

How is yeast allergy treated?
(Tick all that apply)

☐ no treatment ☐ diet ☐ exercise ☐ medication (please specify) ☐ natural/herbal therapy (please specify) ☐ weight management ☐ supplements (please specify) ☐ other (please specify) ☐ Don't know

please specify the medication

____

please specify the natural/herbal therapy

____

please specify the supplements

____

please specify the treatment

____

How is salicylate intolerance treated?
(Tick all that apply)

☐ no treatment ☐ diet ☐ exercise ☐ medication (please specify) ☐ natural/herbal therapy (please specify) ☐ weight management ☐ supplements (please specify) ☐ other (please specify) ☐ Don't know

please specify the medication

____

please specify the natural/herbal therapy

____

please specify the supplements

____

please specify the treatment

____

How is amine intolerance treated?
(Tick all that apply)

☐ no treatment ☐ diet ☐ exercise ☐ medication (please specify) ☐ natural/herbal therapy (please specify) ☐ weight management ☐ supplements (please specify) ☐ other (please specify) ☐ Don't know

please specify the medication

____

please specify the natural/herbal therapy

____

please specify the supplements

____

please specify the treatment

____

How is glutamate intolerance treated?
(Tick all that apply)

☐ no treatment ☐ diet ☐ exercise ☐ medication (please specify) ☐ natural/herbal therapy (please specify) ☐ weight management ☐ supplements (please specify) ☐ other (please specify) ☐ Don't know

please specify the medication

____

please specify the natural/herbal therapy

____

please specify the supplements

____

please specify the treatment

____

How is colour/flavour/preservative (e.g. sulphite) intolerance treated?
(Tick all that apply)

☐ no treatment ☐ diet ☐ exercise ☐ medication (please specify) ☐ natural/herbal therapy (please specify) ☐ weight management ☐ supplements (please specify) ☐ other (please specify) ☐ Don't know

please specify the medication

____

please specify the natural/herbal therapy

____

please specify the supplements

____

please specify the treatment

____

How is FODMAPS intolerance (e.g. fructans, fructose) treated?
(Tick all that apply)

☐ no treatment ☐ diet ☐ exercise ☐ medication (please specify) ☐ natural/herbal therapy (please specify) ☐ weight management ☐ supplements (please specify) ☐ other (please specify) ☐ Don't know

please specify the medication

____

please specify the natural/herbal therapy

____

please specify the supplements

____

please specify the treatment

____

How is [allergy_others] treated?
(Tick all that apply)

☐ no treatment ☐ diet ☐ exercise ☐ medication (please specify) ☐ natural/herbal therapy (please specify) ☐ weight management ☐ supplements (please specify) ☐ other (please specify)
☐ Don't know

please specify the medication

____

please specify the natural/herbal therapy

____

please specify the supplements

____

please specify the treatment

____

How is [allergy_others_2] treated?
(Tick all that apply)

☐ no treatment ☐ diet ☐ exercise ☐ medication (please specify) ☐ natural/herbal therapy (please specify) ☐ weight management ☐ supplements (please specify) ☐ other (please specify)
☐ Don't know

please specify the medication

____

please specify the natural/herbal therapy

____

please specify the supplements

____

please specify the treatment

____

How is [allergy_others_3] treated?
(Tick all that apply)

☐ no treatment ☐ diet ☐ exercise ☐ medication (please specify) ☐ natural/herbal therapy (please specify) ☐ weight management ☐ supplements (please specify) ☐ other (please specify)
☐ Don't know

please specify the medication

____

please specify the natural/herbal therapy

____

please specify the supplements

____

please specify the treatment

____

How is this condition treated?
(Tick all that apply)

☐ no treatment ☐ diet ☐ exercise ☐ medication (please specify) ☐ natural/herbal therapy (please specify) ☐ weight management ☐ supplements (please specify) ☐ other (please specify)
☐ Don't know

please specify the medication

____

please specify the natural/herbal therapy

____

please specify the supplements

____

please specify the treatment

____

Have either of your parents, or any of brothers or sisters ever been told by a doctor (GP or specialist), or dietitian that they any food allergy or intolerance?

◯ Yes
◯ No
◯ Don't know

This question is about conditions that you may experience specifically related to training or competing in sport. Please tick any conditions that you regularly experience in training or competition.
(Tick all that apply)

☐ Cramps
☐ Dehydration/hypohydration
☐ Diahrroea
☐ Nausea/vomiting
☐ Stitches
☐ Other (please specify)
☐ Don't Know
☐ I don't experience any conditions related to training or competition

please specify the condition

____

Do you have another condition need to be specified?

◯ Yes ◯ No

please specify the second condition

____

Do you have another condition need to be specified?

◯ Yes ◯ No

please specify the third condition

____

Do you experience any vitamin or mineral deficiencies diagnosed by a blood test?

◯ Yes
◯ No
◯ Don't know

Which vitamin or mineral deficiencies have been diagnosed by a blood test? (Tick all that apply)

☐ Calcium deficiency
☐ Iron deficiency (e.g. low ferritin, low iron, low haemaglobin)
☐ Vitamin D deficiency
☐ Other (please specify)
☐ Don't Know

How is the calcium deficiency treated?

☐ no treatment ☐ diet ☐ exercise ☐ natural/herbal therapy (please specify) ☐ supplements (please specify) ☐ other (please specify) ☐ Don't know

please specify the natural/herbal therapy

____

please specify the supplements

____

please specify the treatment

____

How is the iron deficiency (e.g. low ferritin, low iron, low haemaglobin) treated?

☐ no treatment ☐ diet ☐ exercise ☐ natural/herbal therapy (please specify) ☐ supplements (please specify) ☐ other (please specify) ☐ Don't know

please specify the natural/herbal therapy

____

please specify the supplements

____

please specify the treatment

____

How is the vitamin D deficiency treated?

☐ no treatment ☐ diet ☐ exercise ☐ natural/herbal therapy (please specify) ☐ supplements (please specify) ☐ other (please specify) ☐ Don't know

please specify the natural/herbal therapy

____

please specify the supplements

____

please specify the treatment

____

please specify the vitamin or mineral deficiency

____

How is the [sport_deficiency_other_1] deficiency treated?

☐ no treatment ☐ diet ☐ exercise ☐ natural/herbal therapy (please specify) ☐ supplements (please specify) ☐ other (please specify) ☐ Don't know

please specify the natural/herbal therapy __________________________________

please specify the supplements __________________________________

please specify the treatment __________________________________

Do you have another deficiency that needs to be specified?

◯ Yes ◯ No

please specify the second vitamin or mineral deficiency __________________________________

How is the [sport_deficiency_other_2] deficiency treated?

☐ no treatment ☐ diet ☐ exercise ☐ natural/herbal therapy (please specify) ☐ supplements (please specify) ☐ other (please specify) ☐ Don't know

please specify the natural/herbal therapy __________________________________

please specify the supplements __________________________________

please specify the treatment __________________________________

Do you have another deficiency that needs to be specified?

◯ Yes ◯ No

please specify the third vitamin or mineral deficiency __________________________________

How is the [sport_deficiency_other_3] deficiency treated?

☐ no treatment ☐ diet ☐ exercise ☐ natural/herbal therapy (please specify) ☐ supplements (please specify) ☐ other (please specify) ☐ Don't know

please specify the natural/herbal therapy __________________________________

please specify the supplements __________________________________

please specify the treatment __________________________________

Do you take *sports foods or dietary supplements?

- ◯ Yes
- ◯ No
- ◯ Don't know

*For example, sports drink, sports gel, sports bars, sports confectionary, liquid meals, electrolyte replacement (salt tablets), whey protein, creatine, carnitine etc..

Which sports foods or dietary supplements do you take?
(Tick all that apply)

- ☐ beta-alanine
- ☐ bicarbonate
- ☐ beetroot juice
- ☐ caffeine supplements (e.g. strips or tablets)
- ☐ caffeine containing foods taken to assist performance (e.g. coffee, chocolate milk)
- ☐ calcium supplement
- ☐ carnitine
- ☐ chondroitin sulphate
- ☐ creatine
- ☐ curcumin
- ☐ electrolyte replacement (e.g. salt tablets)
- ☐ exotic berries (e.g. acai, goji)
- ☐ fish oil capsules
- ☐ glucosamine
- ☐ glutamine
- ☐ HMB
- ☐ iron supplement
- ☐ liquid meals
- ☐ multivitamin/mineral supplement
- ☐ probiotics
- ☐ quercetin
- ☐ sports bars
- ☐ sports confectionery (e.g. chews)
- ☐ sports drink
- ☐ sports gel
- ☐ tart (Montomorency) cherry
- ☐ vitamin C supplement
- ☐ vitamin D supplement
- ☐ vitamin E supplement
- ☐ whey protein
- ☐ other anti-oxidants (e.g. vitamin A, selenium)
- ☐ don't know

Please select the most important reason you use the dietary supplement/s you have selected. (Please select only one (1) reason for each supplement)

	sport performance	health/medical reason (please specify)	other reason (please specify)
beta-alanine	◯	◯	◯
bicarbonate	◯	◯	◯
beetroot juice	◯	◯	◯
caffeine supplements (e.g. strips or tablets)	◯	◯	◯
caffeine containing foods (e.g. coffee, chocolate milk)	◯	◯	◯
calcium supplement	◯	◯	◯
carnitine	◯	◯	◯
chondroitin sulphate	◯	◯	◯
creatine	◯	◯	◯
curcumin	◯	◯	◯
electrolyte replacement (e.g. salt tablets)	◯	◯	◯
exotic berries (e.g. acai, goji)	◯	◯	◯
fish oil capsules	◯	◯	◯
glucosamine	◯	◯	◯
glutamine	◯	◯	◯
HMB	◯	◯	◯
iron supplement	◯	◯	◯
liquid meals	◯	◯	◯
multivitamin/mineral supplement	◯	◯	◯
probiotics	◯	◯	◯
quercetin	◯	◯	◯
sports bars	◯	◯	◯
sports confectionary (e.g. chews)	◯	◯	◯
sports drink	◯	◯	◯
sports gel	◯	◯	◯
tart (Montomorency) cherry	◯	◯	◯
vitamin C	◯	◯	◯
vitamin D	◯	◯	◯
vitamin E	◯	◯	◯
whey protein	◯	◯	◯
other anti-oxidants (e.g. vitamin A, selenium)	◯	◯	◯

please specify the reason for taking beta-alanine

____

please specify the reason for taking bicarbonate

____

please specify the reason for taking beetroot juice ____________________

please specify the reason for taking caffeine supplements (e.g. strips or tablets) ____________________

please specify the reason for taking caffeine foods (e.g.coffee, chocolate milk) ____________________

please specify the reason for taking the calcium supplement ____________________

please specify the reason for taking carnitine ____________________

please specify the reason for taking chondroitin sulphate ____________________

please specify the reason for taking creatine ____________________

please specify the reason for taking curcumin ____________________

please specify the reason for taking the electrolyte replacement (e.g. salt tablets) ____________________

please specify the reason for taking exotic berries (e.g. acai, goji) ____________________

please specify the reason for taking fish oil capsules ____________________

please specify the reason for taking glucosamine ____________________

please specify the reason for taking glutamine ____________________

please specify the reason for taking HMB ____________________

please specify the reason for taking the iron supplement ____________________

please specify the reason for taking the liquid meals ____________________

please specify the reason for taking the multivitamin/mineral supplement ____________________

please specify the reason for taking probiotics ____________________

please specify the reason for taking quercitin

____

please specify the reason for using sports bars

____

please specify the reason for using sports confectionery

____

please specify the reason for using sports drink

____

please specify the reason for using sports gel

____

please specify the reason for taking tart (Montomorency) cherry

____

please specify the reason for taking vitamin C

____

please specify the reason for taking vitamin D

____

please specify the reason for taking vitamin E

____

please specify the reason for taking whey protein

____

please specify the reason for taking other antioxidants (e.g. vitamin A, selenium)

____

How do you ensure that your supplements are safe (that is that they are not contaminated or banned)? (Tick all that apply)

- ☐ I check the ASADA/WADA prohibited substances list
- ☐ I check that third party testing has been done (e.g. by Informed Sport, HASTA, etc)
- ☐ I take the recommendation of a health or sport professional
- ☐ I take the recommendation of the supplement company (e.g. on the packet or website)
- ☐ I take the recommendation of other athlete/s
- ☐ I use an app (please specify)
- ☐ I don't take any measures to ensure they are safe.
- ☐ Don't Know

please specify the app you are using

____

Do you have any other comments about the testing of your supplements?

____

Which health or sport professional/s make recommendations to you about the safety of your supplements?
(Tick all that apply)

☐ General practitioner (GP)
☐ Medical specialist
☐ Nurse
☐ Dietitian/Nutritionist (general)
☐ Sports Dietitian/Nutritionist
☐ Naturopath
☐ Exercise Physiologist
☐ Physiotherapist
☐ Personal trainer
☐ Coach
☐ Other (please specify)
☐ Don't Know

please specify the health or sport professional

____

This is a list of sources of nutrition information.
Please list in order of importance where you look for nutrition information (1 is the most important and 5 is the least important). Up to 5 options can be selected.

	1	2	3	4	5
General practitioner (GP)	○	○	○	○	○
Medical specialist	○	○	○	○	○
Nurse	○	○	○	○	○
Dietitian/Nutritionist (general)	○	○	○	○	○
Sports Dietitian/Nutritionist	○	○	○	○	○
Naturopath	○	○	○	○	○
Exercise Physiologist	○	○	○	○	○
Physiotherapist	○	○	○	○	○
Personal trainer	○	○	○	○	○
Coach	○	○	○	○	○
Local health food/supplement store	○	○	○	○	○
Internet	○	○	○	○	○
Magazines	○	○	○	○	○
Other	○	○	○	○	○
Don't know	○	○	○	○	○

please specify the source ______________________________

Are you interested in receiving education in nutrition, health, and performance?

○ Yes
○ No
○ Don't know

This is a list of sources of nutrition, health and performance education sources. Please list in order of importance where you look for nutrition information (1 is the most important and 5 is the least important). Up to 5 options can be selected.

	1	2	3	4	5
Consultation with a dietitian	○	○	○	○	○
Group education session	○	○	○	○	○
Webinar	○	○	○	○	○
Face-to-face nutrition course	○	○	○	○	○
Online nutrition course	○	○	○	○	○
Written materials	○	○	○	○	○
Scientific journals	○	○	○	○	○
Website information	○	○	○	○	○
Nutrition Blog	○	○	○	○	○
Other	○	○	○	○	○

please specify the source

____
